# Combining bulk and single-cell RNA-sequencing data to develop an NK cell-related prognostic signature for hepatocellular carcinoma based on an integrated machine learning framework

**DOI:** 10.1186/s40001-023-01300-6

**Published:** 2023-08-30

**Authors:** Qian Feng, Zhihao Huang, Lei Song, Le Wang, Hongcheng Lu, Linquan Wu

**Affiliations:** 1 Department of Emergency, The Second Affiliated Hospital of Nanchang University, Nanchang, 330000 China; 2 Department of General Surgery, The Second Affiliated Hospital of Nanchang University, 1st min de Road, Nanchang, 330000 China; 3 Department of General Practice, The Second Affiliated Hospital of Nanchang University, Nanchang, 330000 China; 4 Department of Blood Transfusion, The Second Affiliated Hospital of Nanchang University, Nanchang, 330000 China

**Keywords:** Hepatocellular carcinoma, Prognosis, Immunotherapy, Single-cell sequencing, Machine learning

## Abstract

**Background:**

The application of molecular targeting therapy and immunotherapy has notably prolonged the survival of patients with hepatocellular carcinoma (HCC). However, multidrug resistance and high molecular heterogeneity of HCC still prevent the further improvement of clinical benefits. Dysfunction of tumor-infiltrating natural killer (NK) cells was strongly related to HCC progression and survival benefits of HCC patients. Hence, an NK cell-related prognostic signature was built up to predict HCC patients’ prognosis and immunotherapeutic response.

**Methods:**

NK cell markers were selected from scRNA-Seq data obtained from GSE162616 data set. A consensus machine learning framework including a total of 77 algorithms was developed to establish the gene signature in TCGA–LIHC data set, GSE14520 data set, GSE76427 data set and ICGC–LIRI–JP data set. Moreover, the predictive efficacy on ICI response was externally validated by GSE91061 data set and PRJEB23709 data set.

**Results:**

With the highest C-index among 77 algorithms, a 11-gene signature was established by the combination of LASSO and CoxBoost algorithm, which classified patients into high- and low-risk group. The prognostic signature displayed a good predictive performance for overall survival rate, moderate to high predictive accuracy and was an independent risk factor for HCC patients’ prognosis in TCGA, GEO and ICGC cohorts. Compared with high-risk group, low-risk patients showed higher IPS–PD1 blocker, IPS–CTLA4 blocker, common immune checkpoints expression but lower TIDE score, which indicated low-risk patients might be prone to benefiting from ICI treatment. Moreover, a real-world cohort, PRJEB23709, also revealed better immunotherapeutic response in low-risk group.

**Conclusions:**

Overall, the present study developed a gene signature based on NK cell-related genes, which offered a novel platform for prognosis and immunotherapeutic response evaluation of HCC patients.

**Supplementary Information:**

The online version contains supplementary material available at 10.1186/s40001-023-01300-6.

## Introduction

Hepatocellular carcinoma (HCC), originating from hepatocytes, is the dominant subtype in primary liver cancer and is characterized with high incidence, delayed diagnosis and poor prognosis [1]. Although major risk factors, such as HBV infection, HCV infection, Aflatoxin B1 exposure, liver cirrhosis, non-alcoholic fatty liver disease (NAFLD) and alcohol abuse, have been ascertained and widely publicized for a long time, the incidence of HCC has been growing in many countries, including developed countries in Europe and North America [2, 3]. In addition, by 2025, an estimated incidence of over 1 million cases annually will cause a large burden of disease across the world [4]. Owing to no specific symptoms at early stages, insensitivity of traditional biomarkers and the lack of large-scale imaging screening program, only about 30% of patients are clinically diagnosed in early stages [5], which also partly contributed to the dismal overall 5-year survival rate of less than 20% [6]. In the past two decades, more optional treatment strategies are offered to patients with HCC, mainly including immunotherapy and molecular targeted therapy [7]. However, only a minority of patients can benefit from each regimen. Thus, tailored therapy concept is proposed and the related mechanisms to select right regimen are fiercely discussed by researchers. With the rapid growth in genomics research, high molecular heterogeneity of HCC is detected and is considered as one of the critical factors to affect prognosis and therapeutic effects [8]. Hence, it is of great significance to investigate novel biomarkers for the prediction of prognosis and treatment effect through high-throughput sequencing data.

Tumor immune microenvironment (TIME) consists of tumor cells, various immunocytes, stromal cells and related extracellular matrix molecules [9]. Though immune system mainly exerts functions of eliminating cancer cells, cancer cells can escape from immune killing and form an immunosuppressive microenvironment [10]. Therefore, a growing number of studies focus on the associations between tumor phenotypes and changes in TIME and confirm that TIME acts a significant role in cancer initiation and development [11, 12]. Natural killer (NK) cell is one of classical cytotoxic cells and innate immune members that can identify and eliminate damaged or stressed cells [13]. In liver tissues, NK cells account for 50% of the lymphocyte population [14]. Unlike acquired immunity, NK cells identify target cells via a cascade of germline-encoded surface receptors, and the functions of NK cells is tightly modulated by activating and inactivating signals from these receptors [15]. In tumor immunity, NK cells rapidly detect tumor cells, directly kill tumor cells and promote immune response mediated by T cells, thus inhibiting cancer occurrence and development [13]. Previous studies revealed decreased infiltrating levels of NK cells in many human cancer types, including gastric cancer, esophageal cancer, breast cancer and colorectal carcinoma [16–18]. In addition, high NK cell infiltration levels in tumor tissues are considered as biomarkers correlated to better prognosis [19–21]. In addition, high NK cell activity in peripheral blood is related to reduced risk of malignancy [22]. As for cancer immunotherapy, NK cell-based treatment has grown rapidly for decades, and its safety and efficacy are widely validated by clinical trials, therefore, becoming a vital domain of immunotherapy innovation [23]. Previous publications have systematically elucidated molecular features of NK cells in bladder cancer, low-grade glioma and neuroblastoma [24–26] and have attempted to build up NK-cell-related gene signature in lung adenocarcinoma, head and neck squamous cell carcinoma, and glioma [27–29], while related studies concerning HCC are still rare.

Single-cell sequencing technology offers an unprecedented opportunity to deepen our understanding on the transcriptomic, genomic, proteomic, epigenomic and metabolomic information of individual cells. In recent years, using single-cell RNA sequencing (scRNA-seq) data to identify immune phenotypes and novel immune cell-related functional biomarkers in tumor microenvironment becomes feasible and popular [30–32]. In the present study, we combined scRNA-seq data and bulk RNA-seq data of HCC to illuminate molecular characteristics of tumor-infiltrating NK cells and to screen out NK cell markers. Using bulk RNA-seq data and corresponding survival data from 4 independent cohorts, an NK cell marker-related prognostic signature based on 77 fundamental or combined machine learning algorithms was next developed and validated, and its associations with immune cell infiltration, immune checkpoint blockade response, chemotherapy sensitivity were further investigated. This work may provide some new ideas on prognosis evaluation and immunotherapy of patients with HCC, thereby promoting the development of individualized treatment and improving patients’ prognosis.

## Materials and methods

### Data acquisition and preparation

A total of 7 data sets, containing gene expression data and corresponding clinical data, were enrolled to perform the present study. The TCGA–LIHC data set (*n* = 370) from The Cancer Genome Atlas (TCGA) database (https://portal.gdccancer.gov/repository) was used to establish the prognostic model as the training group and to perform a series of analyses related to prognosis-related genes selection, somatic mutation, immune microenvironment, immune checkpoint inhibitor (ICI) response and chemotherapy sensitivity. GSE14520 data set (*n* = 242) and GSE76427 data set (*n* = 115) from Gene Expression Omnibus (GEO) database (https://www.ncbi.nlm.nih.gov/geo/), and ICGC–LIRI–JP data set (*n* = 260) from International Cancer Genome Consortium (ICGC) database (https://dcc.icgc.org/) were utilized to verify the predictive power of the prognostic model. The single-cell RNA-seq data were obtained from 3 HCC samples (GSM4955419, GSM4955421, and GSM4955426) in GSE162616 data set from GEO database [33]. Moreover, the two immunotherapy cohorts, GSE91061 and PRJEB23709 data set from GEO database, were also used to externally validate the predictive power of ICI response of the model.

The inclusion criteria used to perform analyses in the present study were as follows: 1. patients with a pathological diagnosis of HCC. 2. Patients with complete RNA-seq data and survival data (survival time and survival status). The exclusion criteria were: 1. duplicate samples. 2. Patients with preoperative anti-HCC therapy. 3. Samples from recurrent HCC patients. 4. Patients with other malignancy history. 5. Patients died within 1 month after surgery. After these data cleansing processes, the demographic and clinicopathological data of the 7 abovementioned data sets was summarized in Additional files 1 to 7, respectively.

### Single-cell RNA-seq data analysis

Through “Seurat” package in R, the original scRNA-seq data were transformed into Seurat format and was standardized by LogNormalize approach. To ensure the quality of scRNA-seq data analysis, we performed the quality control process through excluding the genes expressed in less than 5 single cells, cells expressed less than 50 genes, and cells with mitochondria proportion of over 5%. Next, the top 1500 highly variable genes were distinguished by “FindVariableFeatures” function in “Seurat” package. Using “RunPCA” function, principal components analysis (PCA) based on these 1500 genes was conducted to achieve the dimensional reduction of scRNA-Seq data. According to t-distributed stochastic neighbor embedding (t-SNE) algorithm, unsupervised clustering and unbiased visualization of cell populations in the top 20 PCs were shown in two-dimensional maps. Setting the threshold of |log2 (fold change)| of more than 1 and adjusted *P* value of less than 0.05, “FindAllMarkers” function selected out marker genes of each cluster. Finally, R package “SingleR” annotated cell subtypes in different clusters.

### Weighted gene co-expression network construction and module identification

Weighted correlation network analysis (WGCNA) was often used to identify clusters or modules of highly associated genes. Unlike the conventional method to screen out potential biomarkers only based on differentially expressed genes, WGCNA could also associate modules with one another and with phenotypes, and assess their correlation strength [34]. Using the expression profile and corresponding clinical data from TCGA–LIHC data set, a scale-free co-expression network was established by “WGCNA” package. The soft-threshold value was selected when the independence degree reached 0.9. Thirty was set as the minimum of module size. The correlations between marker genes in each module and survival status, and survival time were evaluated by Pearson’s correlation analysis. The optimal module was selected through comprehensively considering the coefficient and *P* value.

### Survival analysis and signature establishment via integrated machine learning framework

The prognostic performance of the gene markers in the selected module was analyzed by univariate Cox regression analysis. The genes with *P* value of less than 0.05 were defined as prognosis-related genes. Next, the expression matrices and corresponding survival data of these prognosis-related genes were brought into our integrated machine learning framework containing a total of 10 machine learning algorithms and 77 combinations to find out the optimal algorithm to build up the prognostic signature. Of these 10 machine learning algorithms, survival support vector machine (SurvivalSVM), CoxBoost, least absolute shrinkage and selection operator (LASSO), supervised principal components (SuperPC), elastic network (Enet), StepCox, Ridge regression, partial least squares regression for Cox (plsRcox), random forest (RSF) and gradient boosting machine (GBM) were classical algorithms, their statistical characteristics were described in the previous study [35]. RSF, LASSO, CoxBoost and StepCox algorithm could be utilized for feature selection. Hence, the combined algorithms included at least one of the abovementioned four classical algorithms. TCGA–LIHC data set worked as the training cohort, while GSE14520 data set, GSE76427 data set and ICGC–LIRI–JP data set worked as the validation cohorts. In this process, C-indices of corresponding algorithm in the 4 data sets was calculated. The optimal algorithm was chosen according to the highest average C-index among the 4 data sets. Based on the selected algorithm, each HCC patient in the 4 data sets was scored. Setting the median risk score as the cutoff, patients were classified into high- and low-risk groups. Afterward, the overall survival (OS) curves between these two groups in TCGA–LIHC data set and other 3 validation cohorts were compared by Kaplan–Meier method. Using R package “timeROC”, time ROC analysis and clinical ROC analysis were enrolled to evaluate the predictive accuracy of our model. Moreover, univariate and multivariate Cox regression analysis were also conducted to explore whether the risk score was an independent indicator to predict the prognosis of HCC patients.

### Nomogram construction and external comparison among signatures

The above integrated machine learning framework determined the optimal algorithm to build up the risk model. In addition, the power of the risk model in prognostic evaluation was assessed by survival curves, ROC curves and Cox regression analysis. For better correlating with clinical practice, a nomogram combining the risk score and clinical parameters, including age, gender and clinical stage, was plotted through “rms” package in R. The corresponding calibration plot was also constructed to compare the fitting degree between the ideal and the actual survival probabilities. In addition, using “DecisionCurve” package, decision curve analysis (DCA) was also conducted to evaluate predictive accuracy of the nomogram. We also focused on whether the predictive performance of our model surpassed that of published signatures. Harrell's concordance index (C-index) was widely used to describe the fitting degree between predicted value and actual value of Cox model in survival analysis. The C-indices of enrolled signatures were visualized by R package “CompareC”.

### Somatic mutation analysis and landscape of immune microenvironment

Continuous accumulation of somatic mutations induces cancer occurrence and promotes cancer development [36]. The overall somatic mutation categories and frequencies of the samples in TCGA–LIHC data set were acquired from UCSC Xena (https://xena.ucsc.edu/) and were visualized by R package “maftools”. Next, we downloaded the mutation annotation format files from TCGA database and then used “maftools” package to calculate tumor mutation burden (TMB) of each sample. TMB was defined as an applied biomarker to identify potential beneficiaries for ICI treatment, and the detailed statistical processing and calculation methods were described by Chalmers et al. [37]. Estimation of stromal and immune cells in malignant tumor tissues using expression data (ESTIMATE) algorithm was used to predict tumor purity and the infiltrating status of stromal cells and immune cells [38]. In the current study, we also calculated and compared the ImmuneScore, StromalScore, ESTIMATEScore and tumor purity in high- and low-risk group. To more precisely reflect the immune microenvironment in HCC tissues, the infiltration levels of 16 different kinds of immune cells and relative immune functional scores in high- and low-risk patients were then visualized via R package “GSVA”.

### Gene set enrichment analysis

Different from traditional gene function analysis methods, gene set enrichment analysis (GSEA) derives its power through integrating the information from the genes with similar molecular characteristics or biological function, thereby flexibly and comprehensively revealing common biological pathways of the gene group [39]. Based on “GSVA” and “GSEABase” package in R, GSEA was adopted to evaluate different molecular functions and pathways between high- and low-risk group. Furthermore, the threshold of FDR and *q* value was set as 0.05 and 0.2, respectively.

### ICI response prediction and drug sensitivity prediction

As one of the important methods of cancer immunotherapy, ICI treatment has revolutionized treatment prospects of various cancer types, prolonging the survival time of many patients and offering more therapy options for cancer patients [40]. According to the expression profile in TCGA–LIHC data set and R package “ggpubr”, the expression levels of some reported immune checkpoint genes and human leukocyte antigen (HLA)-related genes were compared between high- and low-risk groups. In previous study, the tumor immune dysfunction and exclusion (TIDE) score was considered as an effective predictor for anti-PD1 or anti-CTLA4 therapy [41]. Immunophenoscore (IPS) was calculated according to the four main components, including effector cells, immune checkpoints, immunosuppressive cells and major histocompatibility complex molecules, and a higher IPS was related to increased immunogenicity [42]. We, respectively, downloaded TIDE score and IPS from the TIDE platform (http://tide.dfci.harvard.edu) and The Cancer Immunome Atlas (TCIA) (https://tcia.at/home) and compared them between high- and low-risk patients.

Because publicly accessible cohort focusing on HCC patients’ ICI response was lacking, we could only enroll two immunotherapy cohorts comprising patients with advanced melanoma, GSE91061 data set (anti-PD1 treatment) and PRJEB23709 data set (anti-PD1 treatment or combined anti-PD1/anti-CTLA4 treatment), to externally verify the predictive power of the risk model on response of ICI therapy. GSE91061 cohort and PRJEB23709 cohort included 68 and 120 patients with advanced melanoma, respectively. Of these patients, complete follow-up records and RNA-seq data of 57 patients in GSE91061 cohort and 73 patients in PRJEB23709 cohort were provided. According to the therapeutic response defined by RECIST 1.1 criteria, patients were divided into responders (complete response (CR)/partial response (PR)) and non-responders [stable disease (SD)/progressive disease (PD)]. The similar study design was also shown in previous publications [43, 44].

Half maximal inhibitory concentration (IC50) was usually used in drug sensitivity assessment, and a higher IC50 value was correlated with lower drug sensitivity [45]. From Genomics of Drug Sensitivity in Cancer (GDSC) (https://www.cancerrxgene.org/), we collected IC50 data of several chemotherapy drug or molecular targeted drug used against HCC. Using R package “oncoPredict”, IC50 value of each drug was visualized and compared between high- and low-risk patients.

### Statistical analysis

In the present study, R software version 4.1.2 and GraphPad Prism 9.5.0 was utilized in data processing, statistical analysis and data visualization. Comparisons between two groups were assessed by Student’s *t* test or Wilcoxon test. Kruskal–Wallis test was conducted in comparisons among three groups or above. A two-sided *P* value of less than 0.05 was considered significant.

## Results

### NK cell markers identification by single-cell RNA-seq data

**Fig. 1:**
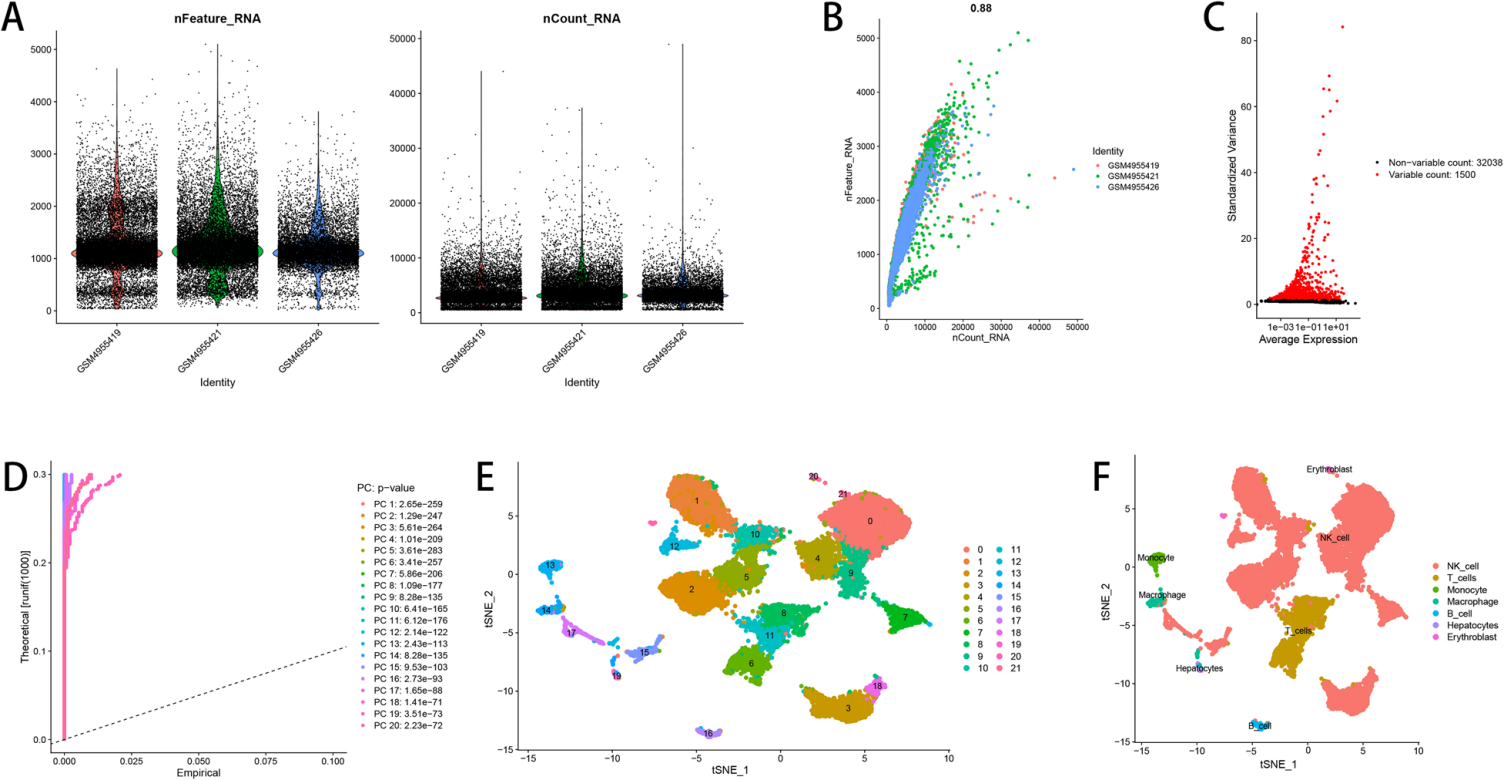
Identification of NK cell markers using scRNA-Seq data analysis. **A** Quality control process of scRNA-Seq data from 3 HCC samples. **B** Association between the detected gene numbers and the sequencing depth. **C** Identification of the top 1500 highly variable genes in scRNA-Seq data. **D** Identification of the top 20 PCs by PCA. **E** Visualization of the 22 clusters through the UMAP algorithm. **F** Cell subpopulations classified by marker genes

The flow diagram concerning study design was shown in Additional file 8. The single-cell RNA-seq data were from 3 HCC samples, GSM4955419, GSM4955421, and GSM4955426, in GSE162616 data set. Numbers of detected genes and sequencing depth in each sample were visualized in Fig. 1A, and a strongly positive association based on Pearson’s correlation test was observed between the detected gene numbers and the sequencing depth (cor = 0.88, *P* value < 0.05, Fig. 1B). Then, the quality control processes were conducted to exclude the cells with low-quality, and 24,653 cells were finally selected out to perform subsequent analyses. In these cells, the standardized gene expression matrices were compared to find out top 1500 highly variable genes (Fig. 1C). The related data were dimensionally reduced by the PCA method, and top 20 PCs were distinguished with *P* value of less than 0.05 (Fig. 1D). The 20 PCs were then classified into 22 clusters through t-SNE algorithm, which were later translated to known cell types (Fig. 1E, F), including NK cells, T cells, monocytes, macrophages, B cells, hepatocytes and erythroblasts. The corresponding markers in each cell type were summarized in Additional file 9, which included 404 NK cell markers.

### Weighted gene co-expression network analysis and module identification

**Fig. 2:**
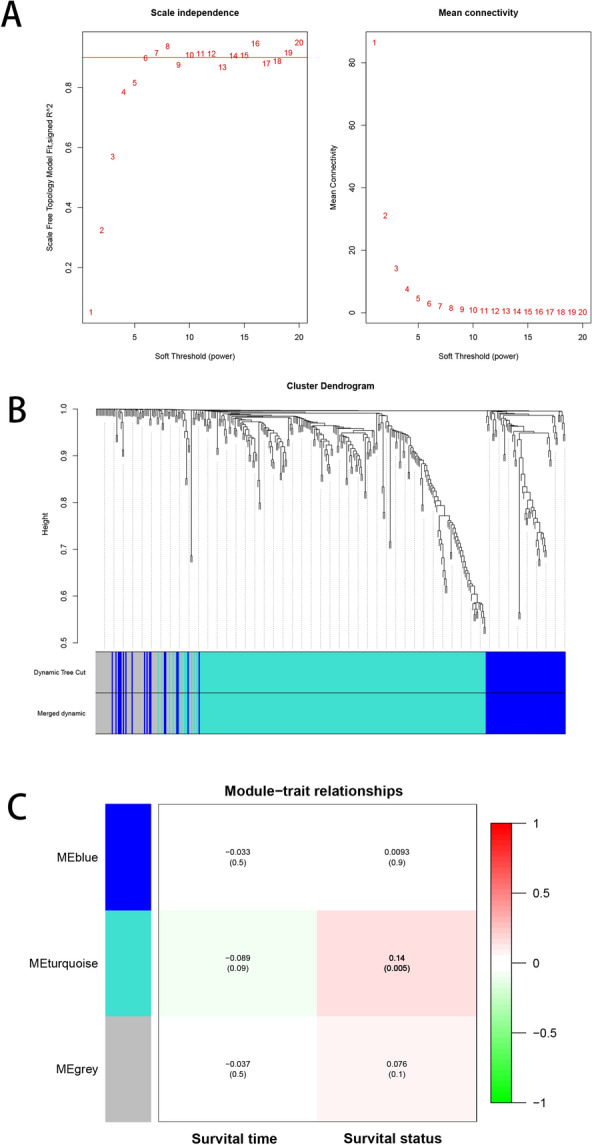
Weighted correlation network analysis to identify the NK cell markers correlated with survival time or survival status. **A** Optimal soft threshold power selection. **B** Clustering dendrogram of the 3 modules with different colors. **C** The relationship between each module and survival time, and survival status

Using the genomic data from TCGA–LIHC cohort, the expression matrices of the 404 NK cell markers were obtained. According to the above data, weighted gene co-expression network analysis was performed to screen out the survival-related markers form the 404 NK cell-related genes. The index of scale-free topologies (R^2^) of 0.9 corresponded to a lowest soft threshold value of 6 (Fig. 2A). The 404 genes were then classified into different modules and eventually formed the blue module, the turquoise module and the grey module (Fig. 2B). The survival-related module was selected using Pearson’s correlation test, and corresponding correlation coefficient and *P* value of each module were visualized in Fig. 2C. Of these 3 modules, the turquoise module was positively correlated with survival status (cor = 0.14, *P* value = 0.005), and a total of 245 genes were included in the turquoise module (Additional file 10).

### Survival analysis and prognostic signature establishment

**Fig. 3:**
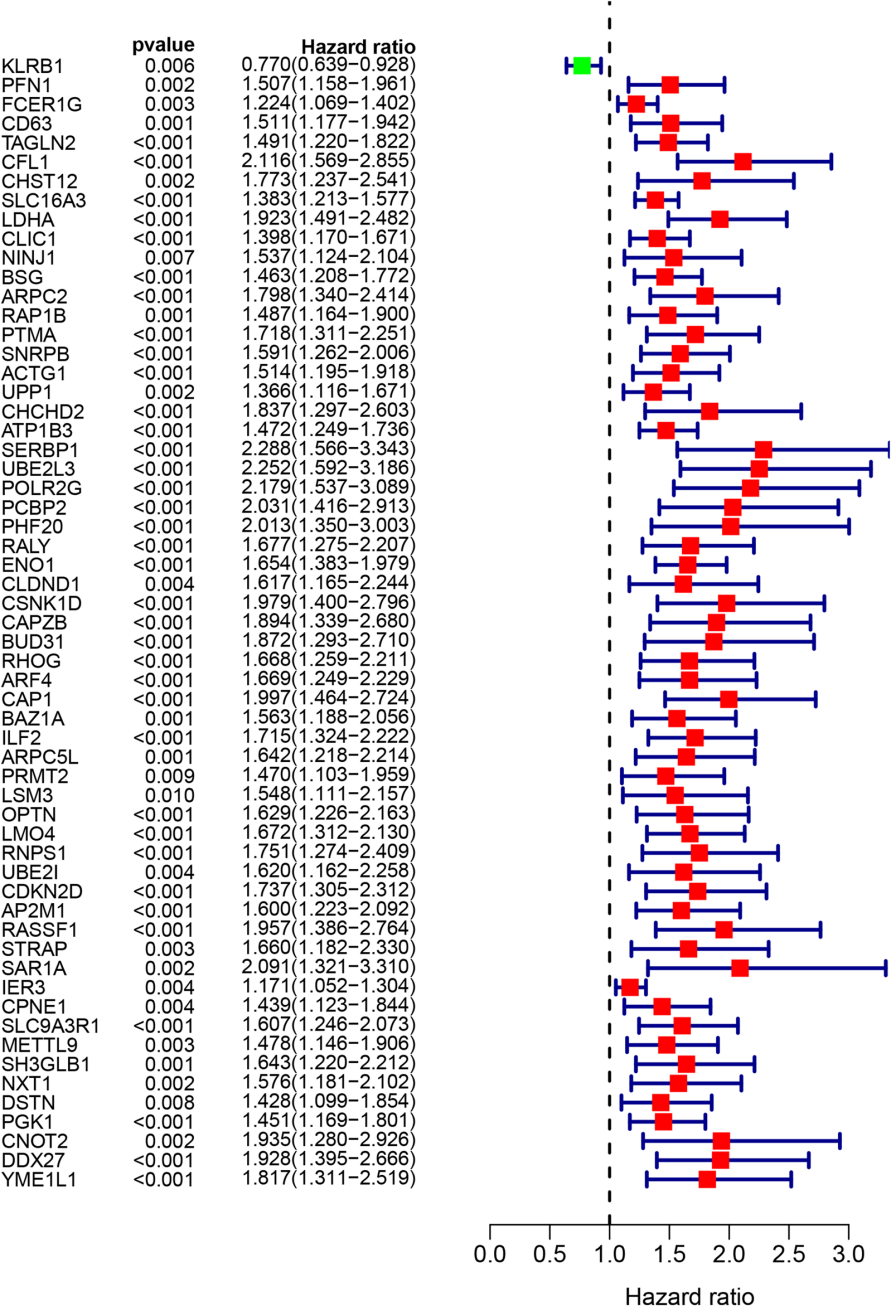
Univariate Cox regression analysis identified 59 prognosis-related markers from the 245 NK cell markers selected by WGCNA

**Fig. 4:**
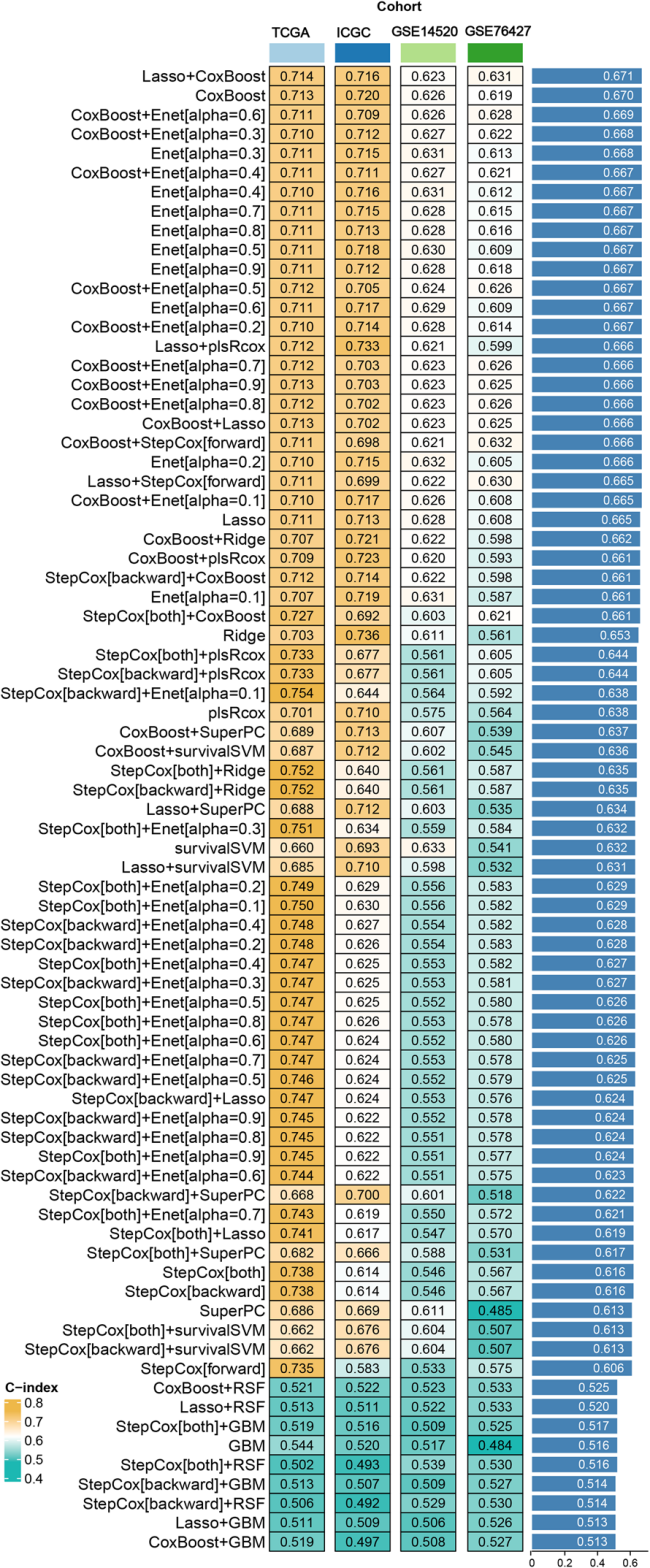
Generation of the NK cell-related signature through consensus machine learning framework, including a total of 77 independent or combined machine learning algorithms

The prognostic performance of the 245 NK cell markers associated with survival status of HCC patients was assessed by univariate Cox regression analysis, and 59 markers were finally identified as prognosis-related markers using TCGA–LIHC data set (all *P* value < 0.05, Fig. 3). Integrated framework including 77 fundamental or combined machine learning algorithms was used to develop a consensus prognostic model based on the 59 prognosis-related markers. The C-index of each model was calculated in TCGA–LIHC cohort, GSE14520 cohort, GSE76427 cohort and ICGC–LIRI–JP cohort. With a leading average C-index of 0.671 among the 4 cohorts, a combination of LASSO and CoxBoost algorithm was considered as the optimal algorithm to construct prognostic model (Fig. 4). The 59 markers were first enrolled to perform LASSO regression analysis to selected out with the minimum partial likelihood deviance and markers with nonzero LASSO coefficients. These markers were next analyzed by CoxBoost proportional hazards regression, and 11 markers, including KLRB1, CFL1, LDHA, BSG, ATP1B3, SERBP1, UBE2L3, PCBP2, ENO1, OPTN and LMO4, were identified to construct prognostic model. The risk score of each patient was calculated according to the expression level weighted by corresponding regression coefficients.

**Fig. 5:**
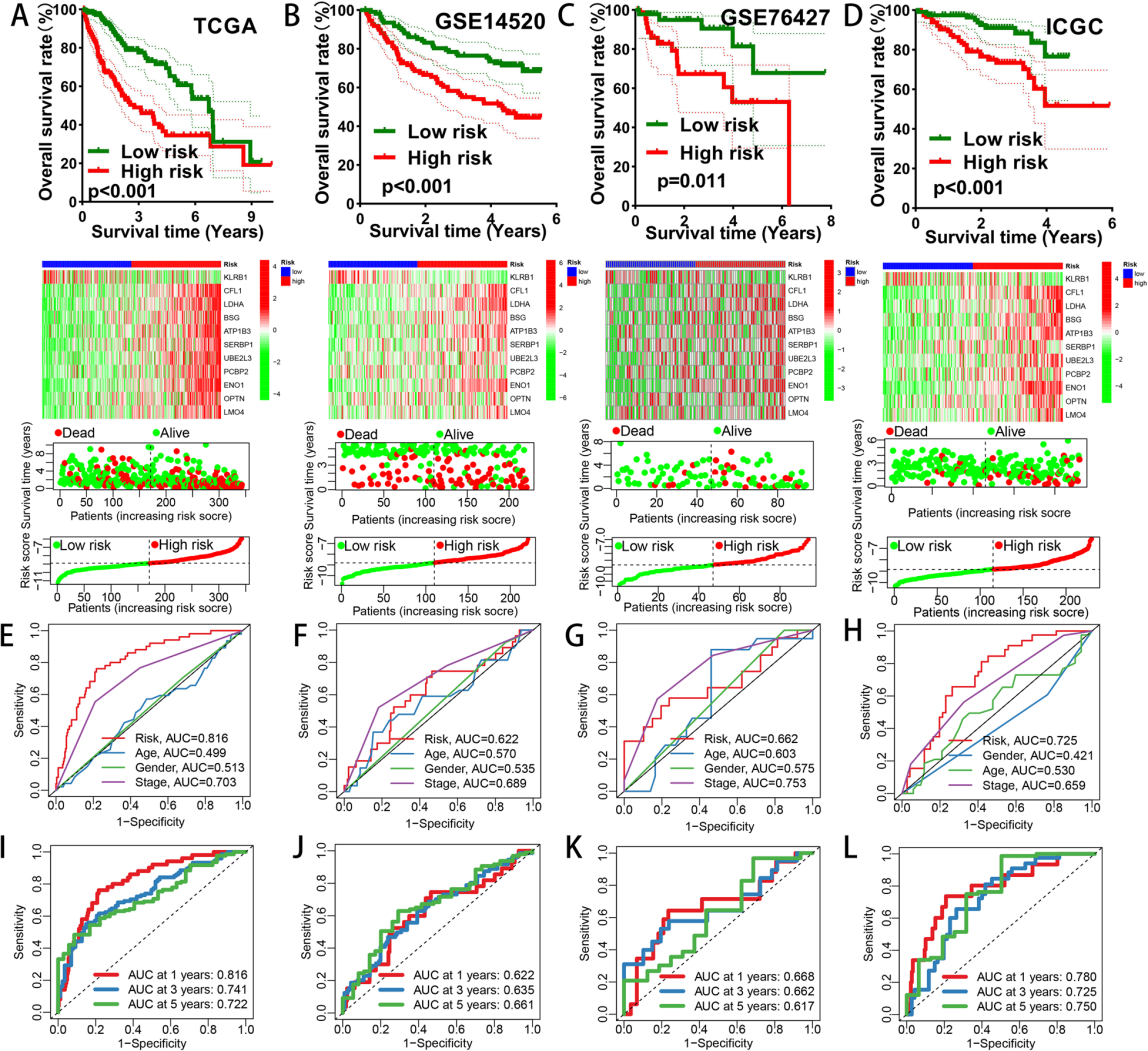
Prognostic model construction and validation. Gene expression status, patients’ survival status, risk score distribution and OS plots of high-risk group and low-risk group in TCGA–LIHC data set (**A**), GSE14520 data set (**B**), GSE76427 data set (**C**) and ICGC–LIRI–JP data set (**D**). Clinical ROC curves for predicting survival rate in TCGA–LIHC data set (**E**), GSE14520 data set (**F**), GSE76427 data set (**G**) and ICGC–LIRI–JP data set (**H**). Time ROC curves to predict 1-year, 3-year and 5-year survival rate in TCGA–LIHC data set (**I**), GSE14520 data set (**J**), GSE76427 data set (**K**) and ICGC–LIRI–JP data set (**L**)

**Fig. 6:**
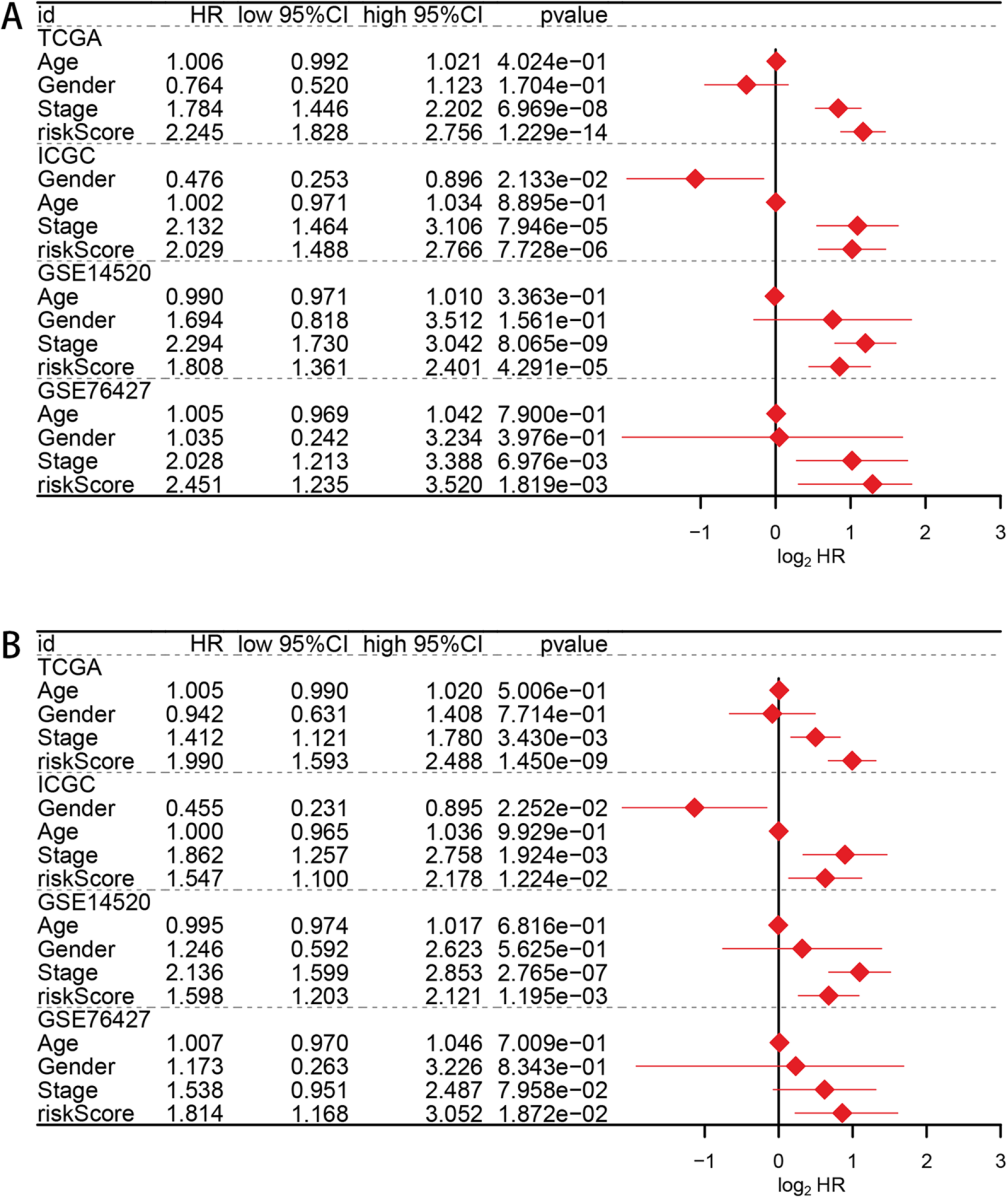
Cox regression analysis considering age, gender, clinical stage and risk score in TCGA–LIHC data set, GSE14520 data set, GSE76427 data set and ICGC–LIRI–JP data set. Univariate method (**A**) and multivariate method (**B**)

In TCGA–LIHC cohort, HCC patients in high-risk group showed a worse prognosis than low-risk patients (*P* value < 0.001, Fig. 5A). Similar survival outcomes were also detected in GSE14520 cohort (*P* value < 0.001, Fig. 5B), GSE76427 cohort (*P* value = 0.011, Fig. 5C) and ICGC–LIRI–JP cohort (*P* value < 0.001, Fig. 5D). The risk score classification, survival status and gene expression of single patient in the above 4 cohorts were also shown. Next, ROC method was utilized to evaluate predictive efficacy of the prognostic signature. In TCGA–LIHC cohort, the AUCs for age, gender, clinical stage and risk score were 0.499 0.513, 0.703 and 0.816, respectively (Fig. 5E). In GSE14520 cohort, the AUCs for age, gender, clinical stage and risk score were 0.570, 0.535, 0.689 and 0.622, respectively (Fig. 5F). In GSE76427 cohort, the AUCs for age, gender, clinical stage and risk score were 0.603, 0.575, 0.753 and 0.662, respectively (Fig. 5G). In ICGC–LIRI–JP cohort, the AUCs for age, gender, clinical stage and risk score were 0.530, 0.421, 0.659 and 0.725, respectively (Fig. 5H). As for time ROC analysis, the 1-year survival, 3-year survival and 5-year survival AUCs in TCGA–LIHC cohort were 0.816, 0.741 and 0.722 (Fig. 5I). The 1-year survival, 3-year survival and 5-year survival AUCs in GSE14520 cohort were 0.622, 0.635 and 0.661 (Fig. 5J). The 1-year survival, 3-year survival and 5-year survival AUCs in GSE76427 cohort were 0.668, 0.662 and 0.617 (Fig. 5K). the 1-year survival, 3-year survival and 5-year survival AUCs in ICGC–LIRI–JP cohort were 0.780, 0.725 and 0.750 (Fig. 5L). These ROC results revealed our signature had a good performance in prognostic assessment of HCC patients. To verify whether the risk score was the independent prognostic indicator for HCC patients, univariate and multivariate Cox regression analysis considering age, gender, clinical stage and risk score were performed in TCGA–LIHC cohort, GSE14520 cohort, GSE76427 cohort and ICGC–LIRI–JP cohort, respectively (Fig. 6A, B). To further expand our results, we also conducted univariate and multivariate Cox regression analysis considering age, gender, race, clinical stage, Child–pugh grade, tumor grade and risk score in TCGA–LIHC data set (Additional file 11A, B), which showed the similar results with Fig. 6A, B. These analyses suggested risk score was one of the independent risk factors for HCC patients’ survival.

### Nomogram construction and comparison with other signatures

**Fig. 7:**
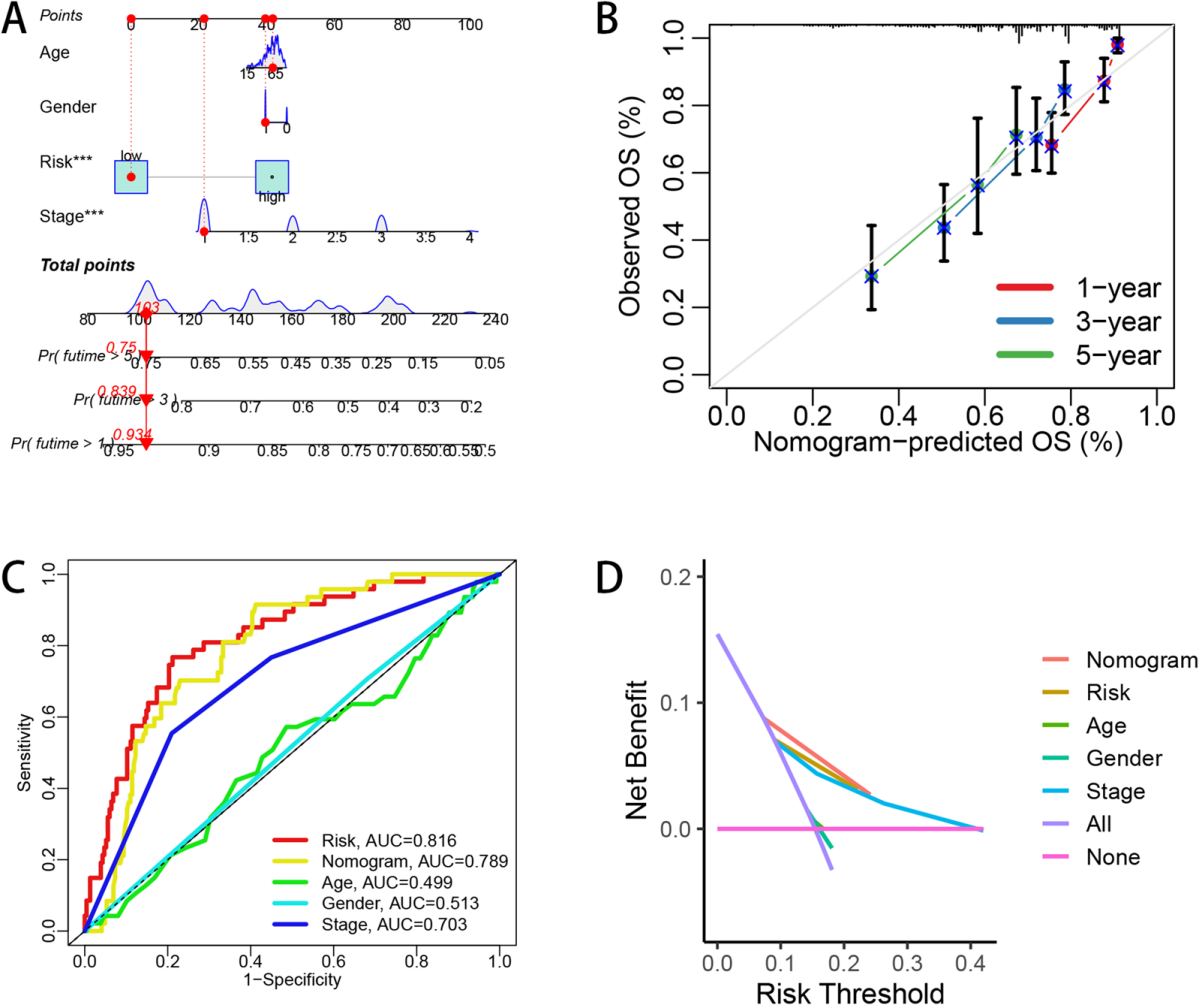
Nomogram establishment and performance assessment. **A** Nomogram considering age, gender, risk score and clinical stage to predict 1-year, 3-year and 5-year survival rate of LIHC patients. Calibration plots (**B**) and clinical ROC curves (**C**) to illustrate the predictive efficacy of the nomogram. **D** Decision curves to reveal the potential clinical application valuation of the nomogram

To offer clinicians with a more quantitative method for prognostic assessment, prognostic nomograms combining age, gender, clinical stage and risk score were established in TCGA–LIHC cohort. Each patient would get a score from corresponding clinical parameter, and a higher total score was related to a worse survival outcome (Fig. 7A). In 1-year, 3-year and 5-year calibration plots, there was a relatively good fitting between actual model and ideal model (Fig. 7B). The clinical ROC curve indicated the nomogram had a good predictive efficacy with the AUC value of 0.789 (Fig. 7C). Furthermore, the decision curves also indicated the high potential of our nomogram in clinical utility (Fig. 7D). To further enrich our study, we also built up a nomogram and corresponding 1-year, 3-year and 5-year calibration plots considering the 7 clinical parameters, including age, gender, race, clinical stage, Child–pugh grade, tumor grade and risk score in TCGA–LIHC data set (Additional file 12A and B). The AUC value of the nomogram was 0.798, which was comparatively high among these clinical factors (Additional file 12C). Furthermore, the decision curve also revealed the nomogram had a relatively good predictive power for HCC patients, prognosis (Additional file 12D).

**Fig. 8:**
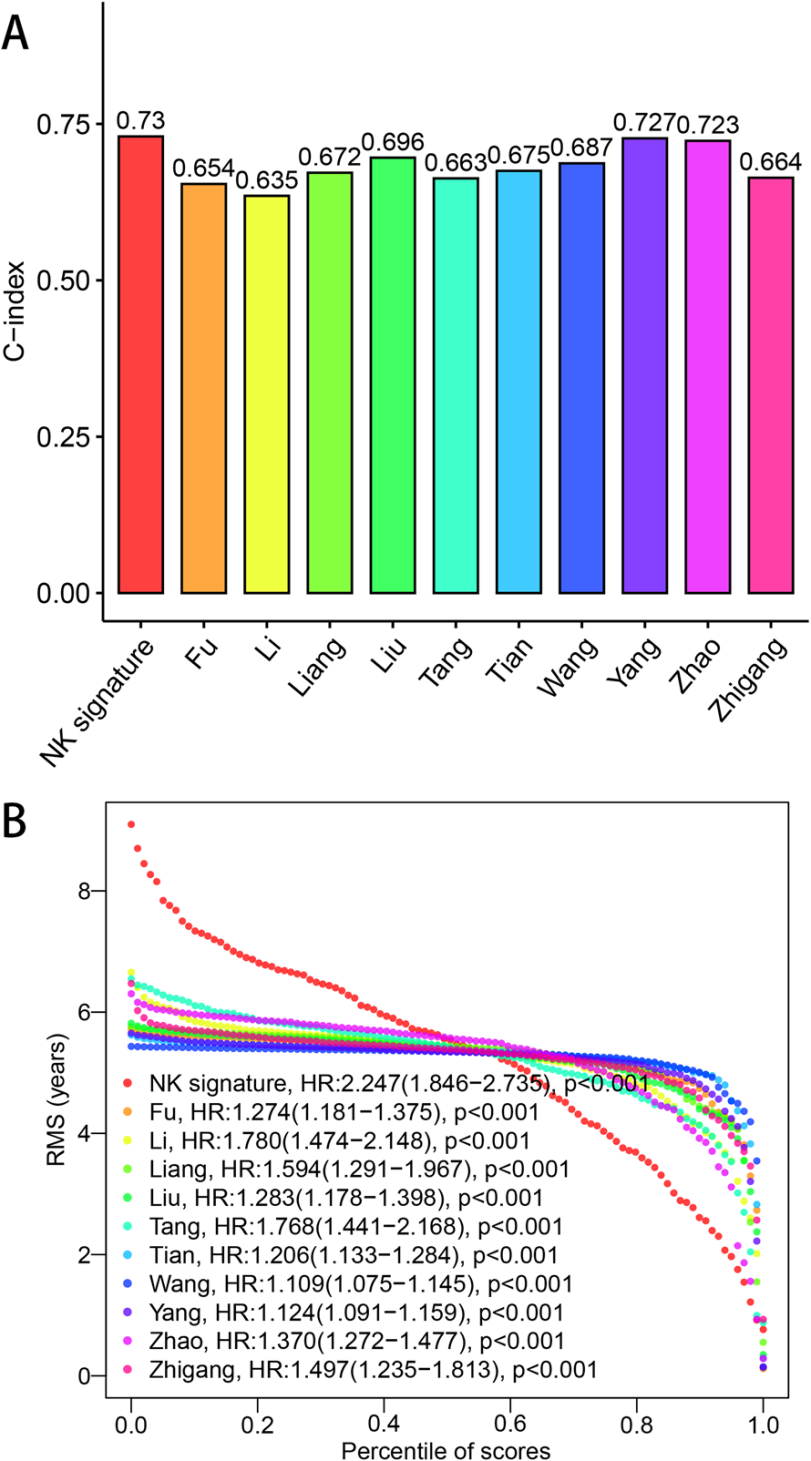
Comparisons with other 10 published signatures. **A** C-indices of the 11 signatures. **B** RMS result of the 11 signatures

To further verify the predictive efficacy of our prognostic signature, other 10 prognostic signatures were enrolled from previous publications [46–55]. The processes of target gene selection, risk score calculation and risk model establishment all obeyed the corresponding original text, and the genomic and clinical data were from TCGA–LIHC data set. Survival plots and corresponding time ROC curves were listed in Additional file 13 and Additional file 14, respectively (all *P* value < 0.05). C-indices of the 11 prognostic signatures were also compared and visualized in Fig. 8A, B. A relatively high C-index of 0.730 was obtained from our NK cell-related signature, suggesting an advanced performance.

### Somatic mutation analysis and immune cell infiltration analysis between high- and low-risk groups

**Fig. 9:**
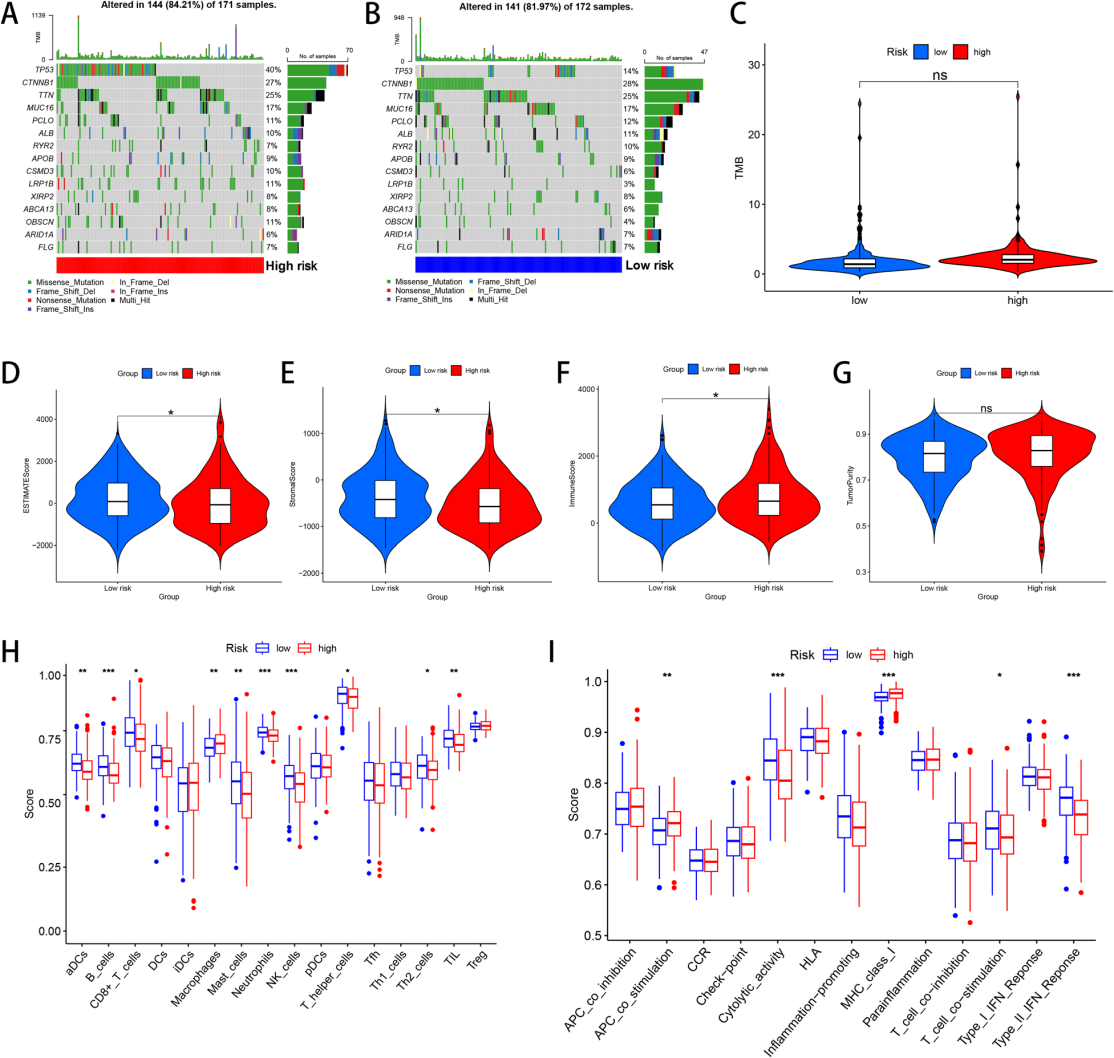
Somatic mutation landscape, immune cell infiltration status and related immune function analysis between high- and low-risk groups. Overview of somatic mutation status in high- (**A**) and low-risk groups (**B**). Tumor mutation burden (**C**), ESTIMATEScore (**D**), StromalScore (**E**), ImmuneScore (**F**), tumor purity (**G**), immune cell infiltration (**H**) and immune function (**I**) comparison between the two groups. *P < 0.05, **P < 0.01, ***P < 0.001

Mutation data from TCGA–LIHC data set was also downloaded and then visualized to reveal the overall somatic mutation status between the two groups. Both in high-risk group (Fig. 9A) and low-risk group (Fig. 9B), TP53, CTNNB1, TTN and MUC16 were identified as the most frequently mutated genes. Next, the overall TMB levels of the two groups was also calculated and compared, which showed no statistical difference between the two groups (*P* value > 0.05, Fig. 9C).

We also investigated the correlations between risk score and immune cell infiltration status in HCC. No significant difference was detected in tumor purity between these two groups (*P* value > 0.05, Fig. 9G), while high-risk patients had lower ESTIMATEScore (*P* value < 0.05, Fig. 9D) and StromalScore (*P* value < 0.05, Fig. 9E), but higher ImmuneScore (*P* value < 0.05, Fig. 9F). More precisely, we next assessed the infiltration levels of 16 kinds of immnocytes according to the ssGSEA algorithm. The infiltrating levels of aDCs, B cells, CD8 + T cells, mast cells, neutrophils, NK cells, T helper cells, Th2 cells and TIL cells in low-risk patients were higher than high-risk patients, while the infiltrating level of macrophages was increased in high-risk patients (all *P* value < 0.05, Fig. 9H). As for common immune functions, the ssGSEA score of corresponding items between these two groups was also compared. The levels of cytolytic activity, T cell co-stimulation and type II IFN response were decreased in high-risk patients, while APC co-stimulation and MHC class I level were increased in high-risk patients (all *P* value < 0.05, Fig. 9I).

### Gene set enrichment analysis between high- and low-risk groups

**Fig. 10:**
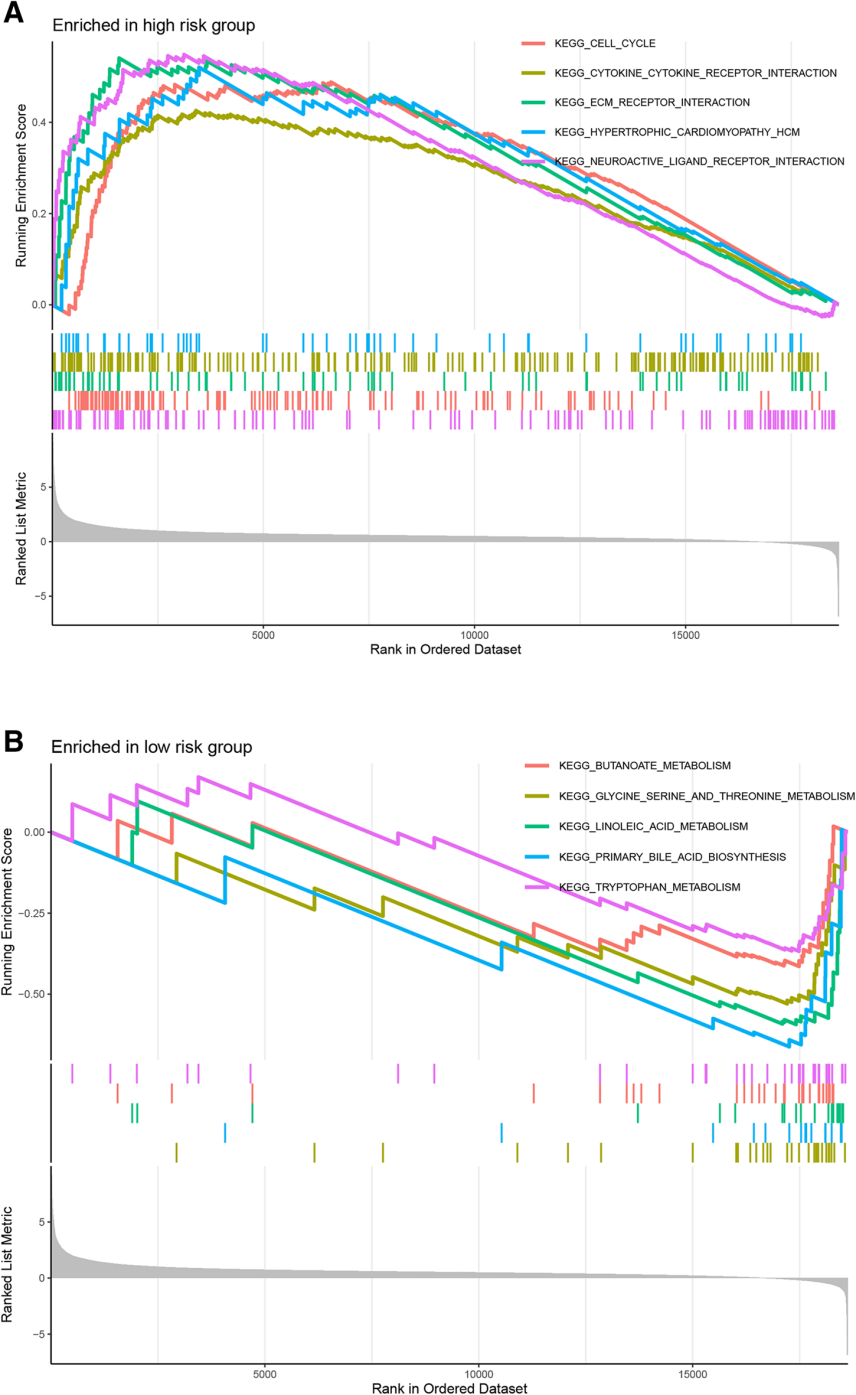
Gene set enrichment analysis. The top 5 enriched items of GSEA in high- (**A**) and low-risk groups (**B**)

GSEA was conducted to explore the potential biological functions and pathways of the prognostic model. The 5 most relevant KEGG items of the two groups were shown. High-risk group was mainly enriched in cell cycle, cytokine–cytokine receptor interaction, ECM receptor interaction, hypertrophic cardiomyopathy and neuroactive ligand receptor interaction (Fig. 10A), while low-risk group was primarily enriched in butanoate metabolism, glycine serine and threonine metabolism, linoleic acid metabolism, primary bile acid biosynthesis and tryptophan metabolism (Fig. 10B).

### Immunotherapeutic benefit prediction between high- and low-risk groups

**Fig. 11:**
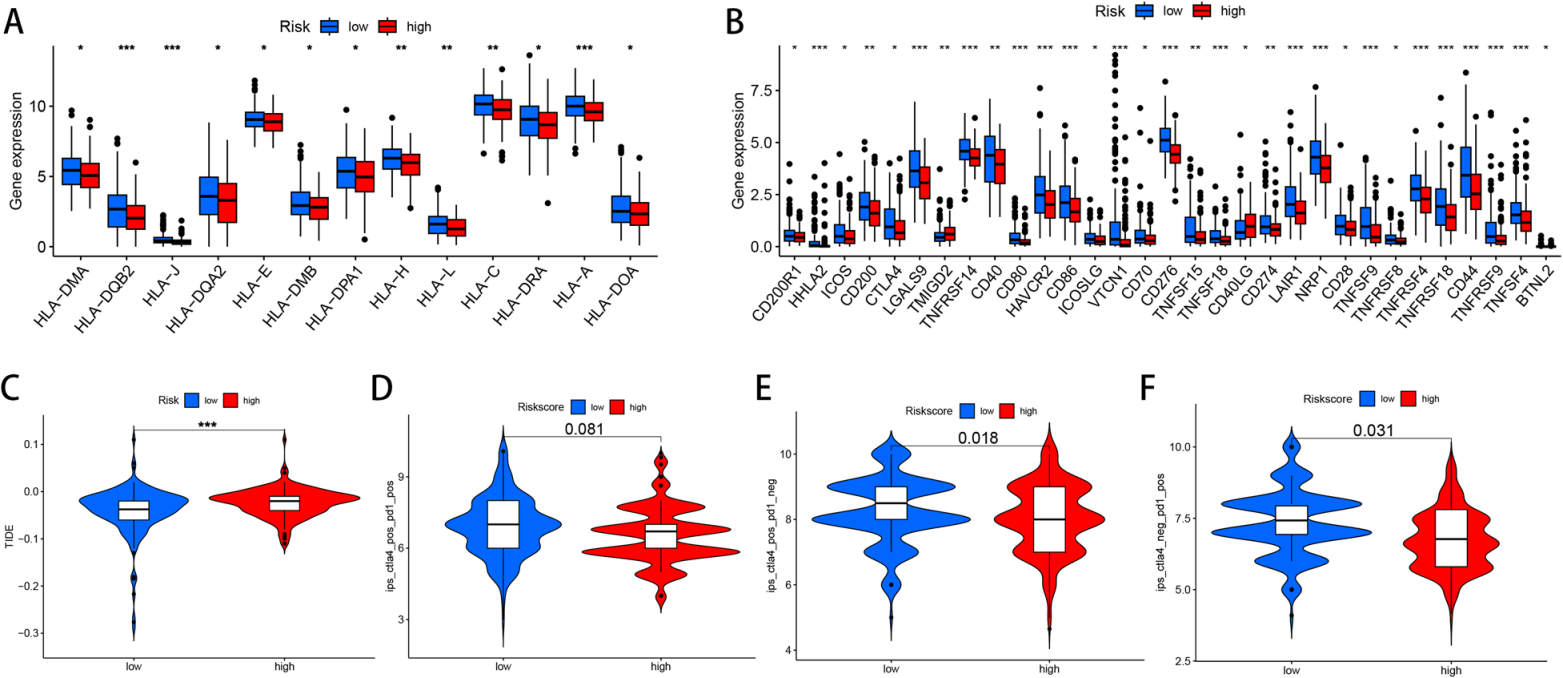
Role of our signature for immunotherapeutic benefits prediction. Differential expression levels of HLA-related genes (**A**) and immune checkpoint-related genes (**B**) between low-risk and high-risk groups. Comparison of TIDE score (**C**), IPS–PD1 + CTLA4 blocker (**D**), IPS–CTLA4 blocker (**E**) and IPS–PD1 blocker (**F**) between low- and high-risk groups. *P < 0.05, **P < 0.01, ***P < 0.001

Previous publications emphasized the major role of NK cells in anti-tumor immunity and immunotherapy [23, 56]. Thus, we investigated whether the risk score could predict ICI response of HCC patients. We first compared the expression levels of HLA-related genes in the two groups and found that the expression levels of HLA–DMA, HLA–DQB2, HLA-J, HLA–DQA2, HLA-E, HLA–DMB, HLA–DPA1, HLA-H, HLA-L, HLA-C, HLA–DRA, HLA-A and HLA–DOA were significantly higher in low-risk patients (all *P* value < 0.05, Fig. 11A). Next, the expression levels of common immune checkpoints between the two groups were also compared. As shown in Fig. 11B, the TMIGD2 expression and the CD40LG expression were increased in high-risk group, while the CD200R1, HHLA2, ICOS, CD200, CTLA4, LGALS9, TNFRSF14, CD40, CD80, HAVCR2, CD86, ICOSLG, VTCN1, CD70, CD276, TNFSF15, TNFSF18, CD274, LAIR1, NRP1, CD28, TNFSF9, TNFRSF8, TNFRSF4, TNFRSF18, CD44, TNFRSF9, TNFSF4 and BTNL2 expression were significantly elevated in low-risk group (all *P* value < 0.05, Fig. 11B). Subsequently, we also compared TIDE score in the two groups and detected that TIDE score in high-risk group was significantly higher (*P* value < 0.001, Fig. 11C). Moreover, we also analyzed IPS–CTLA4 + PD1 blocker, IPS–CTLA4 blocker and IPS–PD1 blocker in the two groups. No significant statistical difference was detected in IPS–CTLA4 + PD1 blocker (*P* value = 0.081, Fig. 11D), while IPS–CTLA4 blocker (*P* value = 0.018, Fig. 11E) and IPS–PD1 blocker (*P* value = 0.031, Fig. 11F) were higher in low-risk patients. From the above results, patients in low-risk group might be more likely to benefit from ICI treatment.

### External validation of immunotherapy responses between high- and low-risk groups

**Fig. 12:**
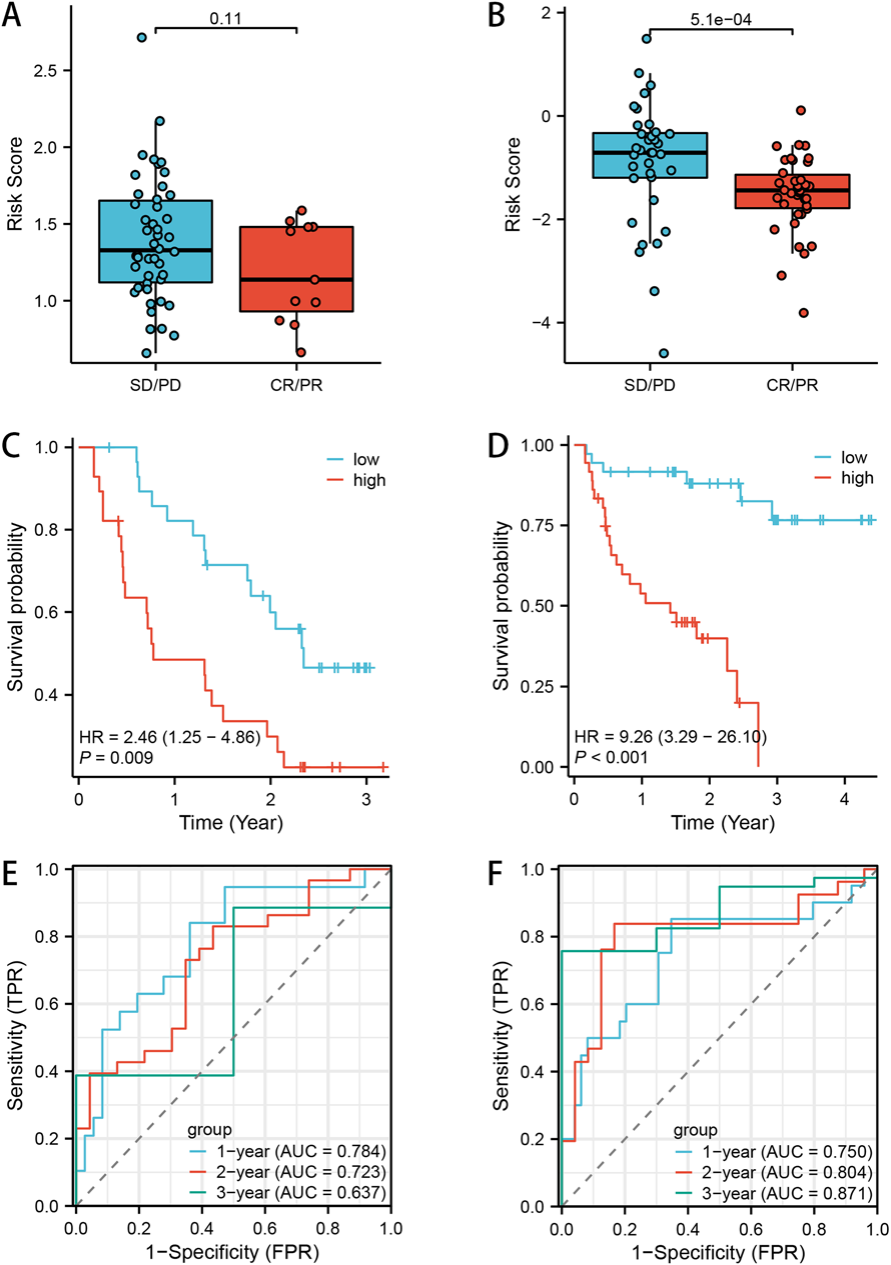
Real-world immunotherapy cohorts to externally validate our NK cell-related signature. The risk score distribution between responders and non-responders in GSE91061 data set (**A**) and PRJEB23709 data set (**B**). The OS curves of high- and low-risk groups in GSE91061 data set (**C**) and PRJEB23709 data set (**D**). Time ROC analysis to predict 1-year, 2-year and 3-year survival rate in GSE91061 data set (**E**) and PRJEB23709 data set (**F**)

In the abovementioned analyses, patients in low-risk group were detected to be more reactive to ICI treatment. Next, we also enrolled the two immunotherapy cohorts (GSE91061 cohort and PRJEB23709 cohort) to externally validate this result. Although there was no significant statistical difference of risk score between responders (CR/PR group) and non-responders (SD/PD group) in GSE91061 cohort (*P* value = 0.11, Fig. 12A), high-risk patients were still correlated with an adverse prognosis (*P* value = 0.009, Fig. 12C). The AUC values of 1-year, 2-year and 3-year survival rate were 0.784, 0.723 and 0.637, respectively (Fig. 12E). As for PRJEB23709 cohort, a significant lower risk score was detected in responders (CR/PR group) (*P* value = 5.1e−04, Fig. 12B), and high-risk patients showed a worse prognosis than those in low-risk group (*P* value < 0.001, Fig. 12D), which was similar with ours. In addition, the AUC values of 1-year, 2-year and 3-year survival rate in PRJEB23709 cohort were 0.750, 0.804 and 0.871, respectively (Fig. 12F).

### Drug sensitivity evaluation between high- and low-risk groups

**Fig. 13:**
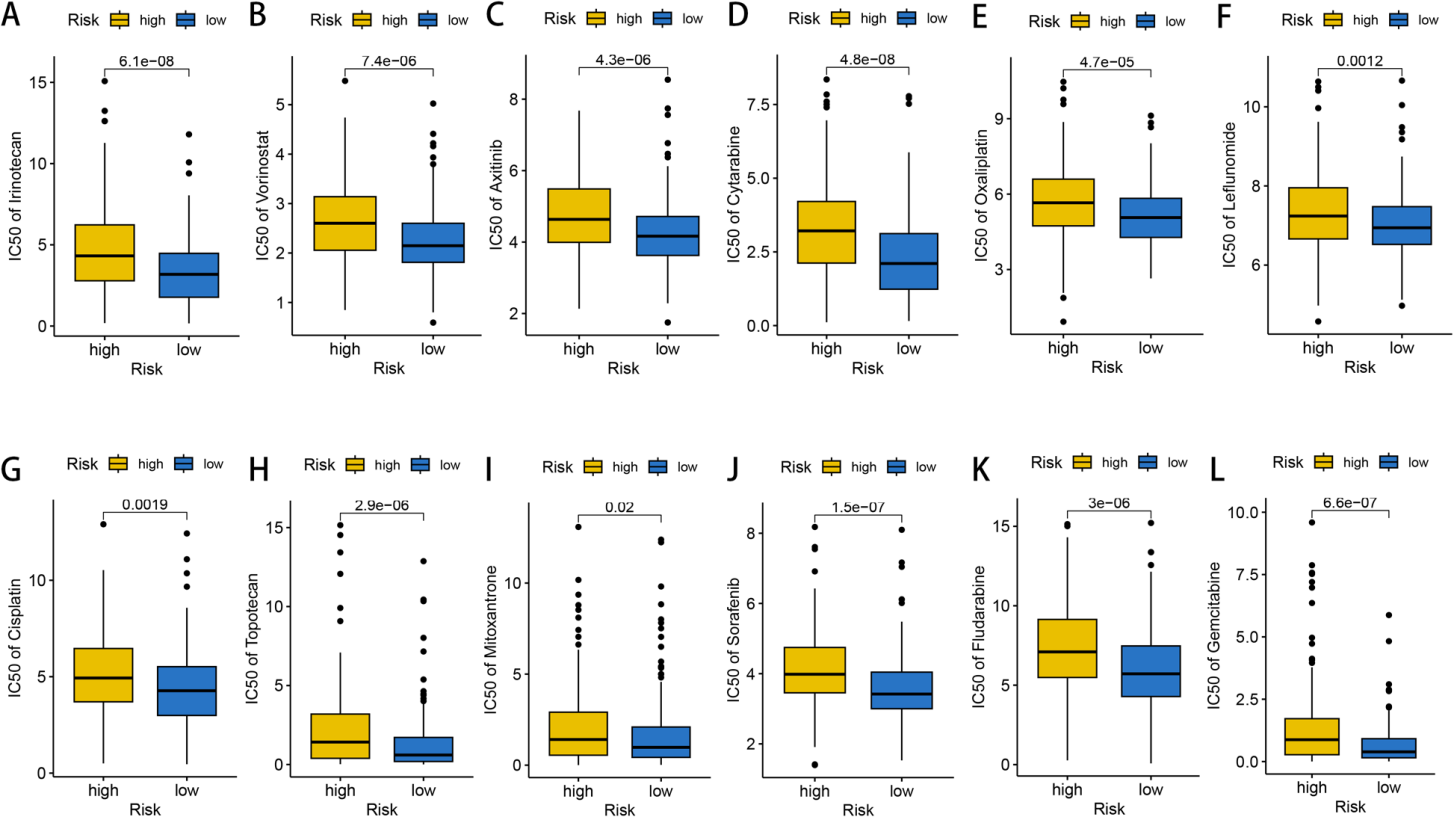
Comparison of estimated IC50 value between high- and low-risk groups. Low‐risk patients were more sensitive to irinotecan (**A**), vorinostat (**B**), axitinib (**C**), cytarabine (**D**), oxaliplatin (**E**), leflunomide (**F**), cisplatin (**G**), topotecan (**H**), mitoxantrone (**I**), sorafenib (**J**), fludarabine (**K**) and gemcitabine (**L**)

The IC50 levels of some common targeted or chemotherapy drugs used against HCC were further compared between high- and low-risk patients. As shown in Fig. 13, IC50 values of irinotecan (*P* value = 6.1e−08, Fig. 13A), vorinostat (*P* value = 7.4e−06, Fig. 13B), axitinib (*P* value = 4.3e−06, Fig. 13C), cytarabine (*P* value = 4.8e−08, Fig. 13D), oxaliplatin (*P* value = 4.7e−05, Fig. 13E), leflunomide (*P* value = 0.0012, Fig. 13F), cisplatin (*P* value = 0.0019, Fig. 13G), topotecan (*P* value = 2.9e−06, Fig. 13H), mitoxantrone (*P* value = 0.02, Fig. 13I), sorafenib (*P* value = 1.5e−07, Fig. 13J), fludarabine (*P* value = 3e−06, Fig. 13K) and gemcitabine (*P* value = 6.6e−07, Fig. 13L) in low-risk group were significantly lower than those in high-risk group, which revealed that low-risk patients might be more reactive to targeted therapy and chemotherapy.

## Discussion

scRNA-seq technology could collect and classify the genomic data from various cell subpopulations in TME, thereby deepening our understanding of tumor heterogeneity and molecular features of tumor-infiltrating immunocytes. NK cell dysfunction had been observed in HCC tissues, which mainly contained abnormal frequency and phenotype of NK cells, and functional impairment of NK cells [57]. Considering such phenomena, accumulating studies attempted to explore the features of NK cells in HCC and related immunoregulation of NK cells in TME. HCC patients with low intratumoral NK cells showed higher recurrence rate and shorter overall survival rate after surgical resection [58]. In addition, NK cell markers, such as CD96, TIM-3 and TIGIT, were strongly correlated with functional exhaustion of NK cells and poor survival outcomes in HCC [59, 60]. As for NK cell-based immunotherapy of HCC, various immunotherapeutic strategies, including antibody-dependent cell-mediated cytotoxicity (ADCC) promoter, immune checkpoint blockade, genetically modified NK cells, off target effects on NK cells, autologous NK cell transfer and allogeneic NK cell transfer, were utilized in clinical application or clinical trials, which exhibited an overall positive effect [61]. Nevertheless, there was still a lack of studies to systematically elaborate the role of NK cell markers in prognostic prediction and immunotherapy of HCC patients.

With the rapid rise of computational medicine and bioinformatics, varied machine learning approaches were utilized to develop prediction models based on gene expression data. Nevertheless, why to select a particular algorithm and how to choose the optimal algorithm were rarely discussed in the process of model construction. As a matter of fact, a significant number of researchers chose the algorithm according to personal experience and preference, which might bias the results. To avoid this issue, we combined scRNA-Seq data, bulk RNA-Seq data and the consensus machine learning framework including a total of 77 independent or combined machine learning algorithms to develop a signature based on a total of 11 NK cell marker genes, KLRB1, CFL1, LDHA, BSG, ATP1B3, SERBP1, UBE2L3, PCBP2, ENO1, OPTN and LMO4. In GSE14520 data set, GSE76427 data set, ICGC–LIRI–JP data set and TCGA–LIHC data set, high-risk patients showed a worse OS rate than those with low risk score. In addition, the risk score was identified as the independent risk factor to affect patients’ prognosis via univariate and multivariate Cox regression analysis. ROC curves based on the survival time and clinical parameters revealed moderate to high prediction accuracy of our signature. In addition, compared to other 10 risk models published in previous studies, the leading C-index of 0.730 was also presented in our signature, displaying relatively high predictive value for HCC patients’ prognosis.

In published studies, the expression level of CFL1 was significantly increased in HCC tissue, and high CFL1 expression was strongly correlated with poorer survival outcomes. Downregulation of CFL1 suppressed proliferation, invasion and epithelial–mesenchymal transformation (EMT) in HCC cell lines [62]. As the upstream regulators of LDHA, miR-383 and miR-142-3p exerted anti-tumor effects via negatively regulating LDHA expression [63, 64]. BSG, also known as CD147, acted as an oncogene in HCC and was considered as prognostic indicator for HCC patients [65]. In addition, BSG overexpression could activate ERK signaling pathway and TGF-β signaling pathway, thereby promoting HCC migration, invasion and EMT [66, 67]. In addition, chimeric antigen receptor (CAR) therapy toward CD147 could selectively kill HCC cells and avoid severe on-target/off-tumor toxicity in mouse model [68]. Silencing of ATP1B3 induced apoptosis and inhibited proliferation and migration in two HCC cell lines, HCCLM3 and Huh7 [69]. SERBP1 was also detected to promote metastasis and EMT of HCC cells, while miR-218 could repressed the pro-tumor functions of SERBP1 [70]. UBE2L3 was also identified as a pro-tumorigenic factor to stimulate HCC cell proliferation by inactivating GSK3β/p65 signaling pathway [71]. PCBP2 expression was also increased both in HCC cell lines and tissues. PCBP2 overexpression predicted unfavorable prognosis for HCC patients and induced proliferation and sorafenib resistance in HCC cells [72]. Ferroptosis of HCC cells was inhibited by ENO1–IRP1–Mfrn1 regulatory axis [73]. Furthermore, elevated OPTN expression promoted the proliferation, migration and mitophagy of HCC cells, thus modulating HCC progression [74].

We illustrated the overall somatic mutation status in high- and low-risk groups and identified some highly mutated genes in HCC. In previous study, Ke et al. analyzed the somatic mutation profiles from 22 HCC patients, which revealed that TP53, MUC16 and TTN were the genes with high mutation frequency in HCC [75]. These results were similar with ours. The correlations between immune microenvironment and risk score were also evaluated in these two groups. First, higher StromalScore and ESTIMATEScore were detected in low-risk patients. Afterward, we observed increased infiltration levels of aDCs, B cells, CD8 + T cells, mast cells, neutrophils, NK cells, T helper cells, Th2 cells and TIL cells in low-risk group, while the infiltrating level of macrophages was increased in high-risk patients. In addition, related immune function analysis revealed a decreased ssGSEA score of cytolytic activity, T cell co-stimulation and type II IFN response in high-risk patients. The anti-cancer effects of CD8 + T cells, NK cells and TIL cells in TME were widely recognized in previous study [76]. The increase of tumor-associated macrophages in TME promoted tumor growth and resistance to sorafenib in HCC cells [77]. Shankaran et al. reported that IFN could cooperate with lymphocytes to maintain the immunogenicity of tumor cells, thus inhibiting tumor progression [78]. These evidences indicated anti-tumor immune response may be relatively more active in low-risk patients. Next, we also found that the expression levels of the majority of common immunotherapeutic targets were elevated in low-risk patients. In addition, IPS–CTLA4 blocker and IPS–PD1 blocker were higher in low-risk patients, while TIDE score was higher in high-risk patients. Moreover, the similar risk score distribution and survival status were also observed in the external validation cohort, PRJEB23709. Based on the distribution of cytotoxic immune cells in TME, solid tumor was classified into “hot” and “cold” tumor, and the “hot” tumor was more sensitive to ICI therapy [79]. In the current study, the low-risk group might be identified as “hot” tumor and more likely to benefit from ICI treatment.

The curative effects of cancer targeting agents and chemotherapy drug varied greatly among individuals, which was mainly attributed to tumor heterogeneity [80]. Thus, it had become popular in recent years to utilize tumor molecular characteristics to develop tools to identify potential beneficiary group for specific regimens. In our work, we found that low‐risk group was more sensitive to irinotecan, vorinostat, axitinib, cytarabine, oxaliplatin, leflunomide, cisplatin, topotecan, mitoxantrone, sorafenib, fludarabine and gemcitabine, which provided novel insights on clinical selection of these 12 anti-tumor drugs. In follow-up work, we are willing to explore the underlying molecular mechanisms of the differences in drug sensitivity.

Some limitations in this study should be declared. First, the data used in our work was derived from transcriptome sequencing. Therefore, some of our results may not be applicable to studies with protein level. Secondly, the underlying molecular mechanisms of the prognostic signature to influence the prognosis, drug sensitivity, ICI response, and immune cell infiltration still need further investigation. Third, the genomic data used in the current study was from retrospective studies. We deeply hope our signature can be further verified by multi-center prospective projects. Fourth, due to the lack of publicly available cohorts concerning ICI treatment of HCC patients, we enrolled the immunotherapy cohorts of melanoma patients, GSE91061 and PRJEB23709 data set, to externally validate the predictive power of our signature on ICI response. Although similar study design was detected in previous publications [43, 44], we still expect future researchers can offer related data to supply our findings.

## Conclusions

In all, we developed and validated an NK cell-related gene signature to possess good performance on prognosis and ICI treatment response prediction of HCC patients based on scRNA-Seq data, bulk RNA-Seq data and multiple machine learning algorithms, which might offer a promising tool for risk stratification and immunotherapeutic guidance of HCC patients.

### Supplementary Information

**Additional file 1.** The demographic and clinicopathological data of TCGA–LIHC data set.

**Additional file 2.** The demographic and clinicopathological data of GSE14520 data set.

**Additional file 3.** The demographic and clinicopathological data of GSE76427 data set.

**Additional file 4.** The demographic and clinicopathological data of ICGC–LIRI–JP data set.

**Additional file 5.** The demographic and clinicopathological data of GSM4955419, GSM4955421, and GSM4955426 in GSE162616 data set.

**Additional file 6.** The demographic and clinicopathological data of GSE91061 data set.

**Additional file 7.** The demographic and clinicopathological data of PRJEB23709 data set.

**Additional file 8.** The workflow of the present study.

**Additional file 9.** The information of the 404 NK cell markers.

**Additional file 10.** The 245 survival-related NK cell markers selected by WGCNA.

**Additional file 11.** Cox regression analysis considering age, gender, race, clinical stage, Child–pugh grade, tumor grade and risk score in TCGA–LIHC data set. Univariate (A) and multivariate methods (B).

**Additional file 12.** Nomogram establishment and performance assessment. (A) A nomogram considering race, age, gender, tumor grade, Child–pugh grade, risk score and clinical stage to predict 1-year, 3-year and 5-year survival rate of LIHC patients. Calibration plots (B) and clinical ROC curves (C) to illustrate the predictive efficacy of the nomogram. (D) Decision curves to reveal the potential clinical application valuation of the nomogram.

**Additional file 13.** Survival curves of our signature and other 10 gene signature from previous publications. Our signature (A), Fu’s signature (B), Li’s signature (C), Liang’s signature (D), Liu’s signature (E), Tang’s signature (F), Tian’s signature (G), Wang’s signature (H), Yang’s signature (I), Zhao’s signature (J) and Zhigang’s signature (K).

**Additional file 14.** Time ROC curves of our signature and other 10 gene signature from previous publications. Our signature (A), Fu’s signature (B), Li’s signature (C), Liang’s signature (D), Liu’s signature (E), Tang’s signature (F), Tian’s signature (G), Wang’s signature (H), Yang’s signature (I), Zhao’s signature (J) and Zhigang’s signature (K).

## Data Availability

The data sets used and/or analyzed during the current study are available from the corresponding author on reasonable request.

## Abbreviations

HCC: Hepatocellular carcinoma
NK: Natural killer
NAFLD: Non-alcoholic fatty liver disease
TIME: Tumor immune microenvironment
scRNA-seq: Single-cell RNA sequencing
TCGA: The Cancer Genome Atlas
ICI: Immune checkpoint inhibitor
GEO: Gene Expression Omnibus
ICGC: International Cancer Genome Consortium
PCA: Principal components analysis
t-SNE: T-distributed stochastic neighbor embedding
WGCNA: Weighted correlation network analysis
SurvivalSVM: Survival support vector machine
LASSO: Least absolute shrinkage and selection operator
SuperPC: Supervised principal components
Enet: Elastic network
plsRcox: Partial least squares regression for Cox
RSF: Random forest
GBM: Gradient boosting machine
OS: Overall survival
DCA: Decision curve analysis
C-index: Harrell's concordance index
TMB: Tumor mutation burden
ESTIMATE: Estimation of stromal and immune cells in malignant tumor tissues using expression data
GSEA: Gene set enrichment analysis
HLA: Human leukocyte antigen
TIDE: The tumor immune dysfunction and exclusion
IPS: Immunophenoscore
TCIA: The Cancer Immunome Atlas
CR: Complete response
PR: Partial response
SD: Stable disease
PD: Progressive disease
IC50: Half maximal inhibitory concentration
GDSC: Genomics of Drug Sensitivity in Cancer
ADCC: Antibody-dependent cell-mediated cytotoxicity
EMT: Epithelial–mesenchymal transformation

## References

[1] E C, D S (2022). Emerging Therapies for Hepatocellular Carcinoma (HCC). Cancers (Basel).

[2] Laura K, Hashem E-S (2019). Epidemiology and management of hepatocellular carcinoma. Gastroenterology.

[3] Mc Glynn Katherine A, Petrick Jessica L, El-SeragHashem B (2021). Epidemiology of hepatocellular carcinoma. Hepatology.

[4] Hyuna S, Jacques F, Siegel Rebecca L (2021). Global Cancer Statistics 2020: GLOBOCAN Estimates of Incidence and Mortality Worldwide for 36 Cancers in 185 Countries. CA Cancer J Clin.

[5] An T, Oussama H, Victoria C (2018). Epidemiology of hepatocellular carcinoma: target population for surveillance and diagnosis. Abdom Radiol (NY).

[6] Jordi B (2019). Insights into the success and failure of systemic therapy for hepatocellular carcinoma. Nat Rev Gastroenterol Hepatol.

[7] Ziyu L, Yan L, Jinyan Z (2019). Molecular targeted and immune checkpoint therapy for advanced hepatocellular carcinoma. J Exp Clin Cancer Res.

[8] Chan Stephen L, Wong N, Lam W, Jacky K (2022). Personalized treatment for hepatocellular carcinoma: Current status and future perspectives. J Gastroenterol Hepatol.

[9] Bingzhe Lv, Yunpeng W, Dongjiang Ma (2022). Immunotherapy: reshape the tumor immune microenvironment. Front Immunol.

[10] Veronica H, Chiara C, Angela B (2017). Cancer acidity: An ultimate frontier of tumor immune escape and a novel target of immunomodulation. Semin Cancer Biol.

[11] Neda K, Ahad M, Amir B (2020). Immune checkpoints in tumor microenvironment and their relevance to the development of cancer stem cells. Life Sci.

[12] Chen Daniel S (2013). Mellman Ira, Oncology meets immunology: the cancer-immunity cycle. Immunity.

[13] Camille G (2020). NK Cells in the Tumor Microenvironment. Adv Exp Med Biol.

[14] Robinson Mark W (2016). Liver immunology and its role in inflammation and homeostasis. Cell Mol Immunol.

[15] Moretta A, Bottino C, Vitale M (2001). Activating receptors and coreceptors involved in human natural killer cell-mediated cytolysis. Annu Rev Immunol.

[16] Emilie M, Aude S, Marie-Laure T (2011). Human breast cancer cells enhance self tolerance by promoting evasion from NK cell antitumor immunity. J Clin Invest.

[17] Shinichirou I, Koji K, Kousaku M (2011). H_2_O_2_ production within tumor microenvironment inversely correlated with infiltration of CD56(dim) NK cells in gastric and esophageal cancer: possible mechanisms of NK cell dysfunction. Cancer Immunol Immunother.

[18] Niels H, Monika B, Christoph K (2011). Natural killer cells are scarce in colorectal carcinoma tissue despite high levels of chemokines and cytokines. Clin Cancer Res.

[19] Ishigami S, Natsugoe S, Tokuda K (2000). Prognostic value of intratumoral natural killer cells in gastric carcinoma. Cancer.

[20] Coca S, Perez-Piqueras J, Martinez D (1997). The prognostic significance of intratumoral natural killer cells in patients with colorectal carcinoma. Cancer.

[21] Versluis MAC, Marchal S, Plat A (2017). The prognostic benefit of tumour-infiltrating Natural Killer cells in endometrial cancer is dependent on concurrent overexpression of Human Leucocyte Antigen-E in the tumour microenvironment. Eur J Cancer.

[22] Imai K, Matsuyama S, Miyake S (2000). Natural cytotoxic activity of peripheral-blood lymphocytes and cancer incidence: an 11-year follow-up study of a general population. Lancet.

[23] Myers Jacob A, Miller Jeffrey S (2021). Exploring the NK cell platform for cancer immunotherapy. Nat Rev Clin Oncol.

[24] Sun Yuhan, Sedgwick Alexander James, Khan Md Abdullah-Al-Kamran, et al. A Transcriptional Signature of IL-2 Expanded Natural Killer Cells Predicts More Favorable Prognosis in Bladder Cancer. Front Immunol 2021; 12: 724107. 10.3389/fimmu.2021.724107 PMC8631443 34858395

[25] Ombretta M, Marco C, Valeria L (2020). Cellular and gene signatures of tumor-infiltrating dendritic cells and natural-killer cells predict prognosis of neuroblastoma. Nat Commun.

[26] Yuhan S, James SA, Yaseelan P (2021). A Transcriptional Signature of PDGF-DD activated natural killer cells predicts more favorable prognosis in low-grade glioma. Front Immunol.

[27] Peng S, Wenbin Li, Lei G (2022). Identification and validation of a novel signature based on NK Cell marker genes to predict prognosis and immunotherapy response in lung adenocarcinoma by integrated analysis of single-cell and bulk RNA-sequencing. Front Immunol.

[28] Chenglong Li, Fangkun L, Lunquan S (2022). Natural killer cell-related gene signature predicts malignancy of glioma and the survival of patients. BMC Cancer.

[29] Hao C, Xixi X, Yingjie Y (2022). Natural killer cell-related prognosis signature characterizes immune landscape and predicts prognosis of HNSCC. Front Immunol.

[30] Elham A, Ambrose CJ, George P (2018). Single-cell map of diverse immune phenotypes in the breast tumor microenvironment. Cell.

[31] Jin Z, Can G, Fang X (2020). Single cell RNA-seq reveals the landscape of tumor and infiltrating immune cells in nasopharyngeal carcinoma. Cancer Lett.

[32] Pan Yu, Fengchun Lu, Qinglin F (2019). Single-cell RNA sequencing reveals compartmental remodeling of tumor-infiltrating immune cells induced by anti-CD47 targeting in pancreatic cancer. J Hematol Oncol.

[33] Huan L, Ronghua Z, Rongrong Q (2022). Panoramic comparison between NK cells in healthy and cancerous liver through single-cell RNA sequencing. Cancer Biol Med.

[34] Peter L, Steve H (2008). WGCNA: an R package for weighted correlation network analysis. BMC Bioinformatics.

[35] Hui Xu, Zaoqu L, Siyuan W (2022). Artificial intelligence-driven consensus gene signatures for improving bladder cancer clinical outcomes identified by multi-center integration analysis. Mol Oncol.

[36] Fleck Julia L, Pavel Ana B, Cassandras Christos G (2016). Integrating mutation and gene expression cross-sectional data to infer cancer progression. BMC Syst Biol.

[37] Chalmers Zachary R, Connelly Caitlin F, Fabrizio D (2017). Analysis of 100,000 human cancer genomes reveals the landscape of tumor mutational burden. Genome Med.

[38] Kosuke Y, Maria S, Emmanuel M (2013). Inferring tumour purity and stromal and immune cell admixture from expression data. Nat Commun.

[39] Subramanian A, Tamayo P, Mootha VK (2005). Gene set enrichment analysis: a knowledge-based approach for interpreting genome-wide expression profiles. Proc Natl Acad Sci U S A.

[40] Sreya B, Robert Y (2021). Immune checkpoint inhibitors for the treatment of cancer: clinical impact and mechanisms of response and resistance. Annu Rev Pathol.

[41] Jiang Peng Gu, Shengqing PD (2018). Signatures of T cell dysfunction and exclusion predict cancer immunotherapy response. Nat Med.

[42] Pornpimol C, Francesca F, Mihaela A (2017). Pan-cancer immunogenomic analyses reveal genotype-immunophenotype relationships and predictors of response to checkpoint blockade. Cell Rep.

[43] Chen W, Mingkai C, Wenying D (2022). Characterization of gastric cancer stem-like molecular features, immune and pharmacogenomic landscapes. Brief Bioinform.

[44] Donghai X, Yian W, Ming Y (2020). A gene expression signature of TREM2 macrophages and γδ T cells predicts immunotherapy response. Nat Commun.

[45] Marc H, Mario N, Kartik S (2017). Designing drug-response experiments and quantifying their results. Curr Protoc Chem Biol.

[46] Xiao-Wei Fu, Chun-Qing S (2021). Identification and validation of pyroptosis-related gene signature to predict prognosis and reveal immune infiltration in hepatocellular carcinoma. Front Cell Dev Biol.

[47] Dazhi T, Yang Y, Li Z (2021). A five-gene-based prognostic signature for hepatocellular carcinoma. Front Med (Lausanne).

[48] Wang Zhangding F, Yao XA (2021). Prognostic and predictive role of a metabolic rate-limiting enzyme signature in hepatocellular carcinoma. Cell Prolif.

[49] Zhigang W, Leyu P, Deliang G (2021). A novel five-gene signature predicts overall survival of patients with hepatocellular carcinoma. Cancer Med.

[50] Lili L, Rongrong X, Guangrong L (2021). Identification of m6A methyltransferase-related lncRNA signature for predicting immunotherapy and prognosis in patients with hepatocellular carcinoma. Biosci Rep.

[51] Jiaying L, Yaofeng Z, Wenhui D (2021). Development and validation of ferroptosis-related lncRNAs signature for hepatocellular carcinoma. PeerJ.

[52] Yajuan Z, Junli Z, Shuhan W (2021). Identification and validation of a nine-gene amino acid metabolism-related risk signature in HCC. Front Cell Dev Biol.

[53] Gao-Min L, Hua-Dong Z, Cai-Yun Z (2019). Identification of a six-gene signature predicting overall survival for hepatocellular carcinoma. Cancer Cell Int.

[54] Zichang Y, Quan Z, Kang X (2021). Development of a macrophages-related 4-gene signature and nomogram for the overall survival prediction of hepatocellular carcinoma based on WGCNA and LASSO algorithm. Int Immunopharmacol.

[55] Linsong T, Rongli W, Ronggao C (2022). Establishment and validation of a cholesterol metabolism-related prognostic signature for hepatocellular carcinoma. Comput Struct Biotechnol J.

[56] Peterson Erin E, Barry Kevin C (2020). The natural killer-dendritic cell immune axis in anti-cancer immunity and immunotherapy. Front Immunol.

[57] Cheng S, Hao-yu S, Wei-hua X (2015). Natural killer cell dysfunction in hepatocellular carcinoma and NK cell-based immunotherapy. Acta Pharmacol Sin.

[58] Valerie C, Jinmiao C, Deming L (2012). Chemokine-driven lymphocyte infiltration: an early intratumoural event determining long-term survival in resectable hepatocellular carcinoma. Gut.

[59] Lihua Y, Xiaoli L, Xinhui W (2021). TIGIT TIM-3 NK cells are correlated with NK cell exhaustion and disease progression in patients with hepatitis B virus-related hepatocellular carcinoma. Oncoimmunology.

[60] Haoyu S, Qiang H, Mei H (2019). Human CD96 correlates to natural killer cell exhaustion and predicts the prognosis of human hepatocellular carcinoma. Hepatology.

[61] Min Y, Zonghai L (2017). Natural killer cells in hepatocellular carcinoma: current status and perspectives for future immunotherapeutic approaches. Front Med.

[62] Bowen Y, Yazhao Li, Tianxiang C (2021). Hypoxia-induced cofilin 1 promotes hepatocellular carcinoma progression by regulating the PLD1/AKT pathway. Clin Transl Med.

[63] Shengni H, Chengdong L, Li L (2018). miR-142-3p inhibits aerobic glycolysis and cell proliferation in hepatocellular carcinoma via targeting LDHA. Biochem Biophys Res Commun.

[64] Zhixiong F, Langqiu H, Hui J (2017). The miR-383-LDHA axis regulates cell proliferation, invasion and glycolysis in hepatocellular cancer. Iran J Basic Med Sci.

[65] Shaojun Z, Yanhong L, Yang Z (2015). Expression and clinical implications of HAb18G/CD147 in hepatocellular carcinoma. Hepatol Res.

[66] Ning-Yu R, Jiao W, Zhi-Nan C (2015). HAb18G/CD147 is involved in TGF-β-induced epithelial-mesenchymal transition and hepatocellular carcinoma invasion. Cell Biol Int.

[67] Wenjing X, Shufen Z, Fangzhen S (2017). Overexpression of CD147 is associated with poor prognosis, tumor cell migration and ERK signaling pathway activation in hepatocellular carcinoma. Exp Ther Med.

[68] Hsiang-Chi T, Wei X, Saiaditya B (2020). Efficacy of anti-CD147 chimeric antigen receptors targeting hepatocellular carcinoma. Nat Commun.

[69] Shanshan L, Shenglan C, Xiaozhen P (2021). Integrative transcriptomic, proteomic and functional analysis reveals ATP1B3 as a diagnostic and potential therapeutic target in hepatocellular carcinoma. Front Immunol.

[70] Wang Ting Xu, Ling JR (2017). MiR-218 suppresses the metastasis and EMT of HCC cells via targeting SERBP1. Acta Biochim Biophys Sin (Shanghai).

[71] Na-Na T, Zhen-Zhen Z, Ji-Hua R (2020). Overexpression of ubiquitin-conjugating enzyme E2 L3 in hepatocellular carcinoma potentiates apoptosis evasion by inhibiting the GSK3β/p65 pathway. Cancer Lett.

[72] Xiubing Z, Hua Lu, Daliang Y (2016). Overexpression of PCBP2 contributes to poor prognosis and enhanced cell growth in human hepatocellular carcinoma. Oncol Rep.

[73] Tong Z, Linchong S, Yijie H (2022). ENO1 suppresses cancer cell ferroptosis by degrading the mRNA of iron regulatory protein 1. Nat Cancer.

[74] Shoichi I, Tomoharu Y, Takeo T (2021). Suppression of optineurin impairs the progression of hepatocellular carcinoma through regulating mitophagy. Cancer Med.

[75] Lixin K, Jianming S, Jikun F (2021). Somatic mutation profiles revealed by next generation sequencing (NGS) in 39 Chinese hepatocellular carcinoma patients. Front Mol Biosci.

[76] Jingting J, Changping W, Binfeng L (2013). Cytokine-induced killer cells promote antitumor immunity. J Transl Med.

[77] Shao-Lai Z, Zheng-Jun Z, Zhi-Qiang H (2016). Tumor-associated neutrophils recruit macrophages and T-regulatory cells to promote progression of hepatocellular carcinoma and resistance to sorafenib. Gastroenterology.

[78] Shankaran V, Ikeda H, Bruce AT (2001). IFNgamma and lymphocytes prevent primary tumour development and shape tumour immunogenicity. Nature.

[79] Yuan-Tong L, Zhi-Jun S (2021). Turning cold tumors into hot tumors by improving T-cell infiltration. Theranostics.

[80] Zuan-Fu L, MaPatrick C (2019). Emerging insights of tumor heterogeneity and drug resistance mechanisms in lung cancer targeted therapy. J Hematol Oncol.

