# Supplementary material for: Combining bulk and single-cell RNA-sequencing data to develop an NK cell-related prognostic signature for hepatocellular carcinoma based on an integrated machine learning framework
Source: Eur J Med Res. 2023 Aug 30;28:306. doi: 10.1186/s40001-023-01300-6 (PMC10466881; doi:10.1186/s40001-023-01300-6)
Supplement: Supplementary file 1 — Additional file 1. The demographic and clinicopathological data of TCGA–LIHC data set. [file 40001_2023_1300_MOESM1_ESM.docx]

Additional file 1. The demographic and clinicopathological data of TCGA-LIHC dataset.

| Clinical characteristics | Number |
| --- | --- |
| **Age** |  |
| < 60 years | 157 |
| ≥ 60 years | 186 |
| **Gender** |  |
| Female | 110 |
| Male | 233 |
| **Race** |  |
| Asian | 148 |
| White | 169 |
| African american | 16 |
| Unknown | 10 |
| **T stage** |  |
| T1 | 168 |
| T2 | 84 |
| T3 | 75 |
| T4 | 13 |
| Unknown | 3 |
| **N stage** |  |
| N0 | 239 |
| N1 | 3 |
| NX | 101 |
| **M stage** |  |
| M0 | 245 |
| M1 | 3 |
| MX | 95 |
| **Clinical stage** |  |
| Stage Ⅰ | 161 |
| Stage Ⅱ | 77 |
| Stage Ⅲ | 80 |
| Stage Ⅳ | 3 |
| Unknown | 22 |
| **Tumor grade** |  |
| G1 | 53 |
| G2 | 161 |
| G3 | 112 |
| G4 | 12 |
| Unknown | 5 |
| **Child-pugh grade** |  |
| A | 204 |
| B | 20 |
| C | 1 |
| Unknown | 118 |
