# Supplementary material for: Combining bulk and single-cell RNA-sequencing data to develop an NK cell-related prognostic signature for hepatocellular carcinoma based on an integrated machine learning framework
Source: Eur J Med Res. 2023 Aug 30;28:306. doi: 10.1186/s40001-023-01300-6 (PMC10466881; doi:10.1186/s40001-023-01300-6)
Supplement: Supplementary file 2 — Additional file 2. The demographic and clinicopathological data of GSE14520 data set. [file 40001_2023_1300_MOESM2_ESM.docx]

Additional file 2. The demographic and clinicopathological data of GSE14520 dataset.

| Clinical characteristics | Number |
| --- | --- |
| **Age** |  |
| < 60 years | 177 |
| ≥ 60 years | 44 |
| **Gender** |  |
| Female | 32 |
| Male | 189 |
| **Clinical stage** |  |
| Stage Ⅰ | 95 |
| Stage Ⅱ | 77 |
| Stage Ⅲ | 49 |
