# Supplementary material for: Combining bulk and single-cell RNA-sequencing data to develop an NK cell-related prognostic signature for hepatocellular carcinoma based on an integrated machine learning framework
Source: Eur J Med Res. 2023 Aug 30;28:306. doi: 10.1186/s40001-023-01300-6 (PMC10466881; doi:10.1186/s40001-023-01300-6)
Supplement: Supplementary file 3 — Additional file 3. The demographic and clinicopathological data of GSE76427 data set. [file 40001_2023_1300_MOESM3_ESM.docx]

Additional file 3. The demographic and clinicopathological data of GSE76427 dataset.

| Clinical characteristics | Number |
| --- | --- |
| **Age** |  |
| < 60 years | 41 |
| ≥ 60 years | 53 |
| **Gender** |  |
| Female | 13 |
| Male | 81 |
| **Clinical stage** |  |
| Stage Ⅰ | 40 |
| Stage Ⅱ | 31 |
| Stage Ⅲ | 19 |
| Stage Ⅳ | 3 |
| Unknown | 1 |
