# Supplementary material for: Combining bulk and single-cell RNA-sequencing data to develop an NK cell-related prognostic signature for hepatocellular carcinoma based on an integrated machine learning framework
Source: Eur J Med Res. 2023 Aug 30;28:306. doi: 10.1186/s40001-023-01300-6 (PMC10466881; doi:10.1186/s40001-023-01300-6)
Supplement: Supplementary file 4 — Additional file 4. The demographic and clinicopathological data of ICGC–LIRI–JP data set. [file 40001_2023_1300_MOESM4_ESM.docx]

Additional file 4. The demographic and clinicopathological data of ICGC-LIRI-JP dataset.

| Clinical characteristics | Number |
| --- | --- |
| **Age** |  |
| < 60 years | 45 |
| ≥ 60 years | 185 |
| **Gender** |  |
| Female | 61 |
| Male | 169 |
| **Clinical stage** |  |
| Stage Ⅰ | 39 |
| Stage Ⅱ | 102 |
| Stage Ⅲ | 68 |
| Stage Ⅳ | 21 |
| **Tumor grade** |  |
| G1 | 20 |
| G1-G2 | 11 |
| G2 | 125 |
| G2-G3 | 32 |
| G3 | 23 |
| G4 | 1 |
| Unknown | 18 |
