# Supplementary material for: Combining bulk and single-cell RNA-sequencing data to develop an NK cell-related prognostic signature for hepatocellular carcinoma based on an integrated machine learning framework
Source: Eur J Med Res. 2023 Aug 30;28:306. doi: 10.1186/s40001-023-01300-6 (PMC10466881; doi:10.1186/s40001-023-01300-6)
Supplement: Supplementary file 5 — Additional file 5. The demographic and clinicopathological data of GSM4955419, GSM4955421, and GSM4955426 in GSE162616 data set. [file 40001_2023_1300_MOESM5_ESM.docx]

Additional file 5. The demographic and clinicopathological data of GSM4955419, GSM4955421, and GSM4955426 in GSE162616 dataset.

| Patient ID | Gender | Age | Tumor number | AFP level (ng/ml) | Tumor diameter (cm) | HBV infection | HCV infection | Clinical stage | Lymph node metastasis | Distant metastasis | Tumor grade | Tumors grow to hepatic vessels | Tumors grow to nearby organs |
| --- | --- | --- | --- | --- | --- | --- | --- | --- | --- | --- | --- | --- | --- |
| GSM4955419 | Male | 50 | 1 | 0.83 | 3 | Yes | No | Stage Ⅱ | No | No | G2-G3 | Yes | No |
| GSM4955421 | Male | 76 | Multiple | 110.3 | 3.5 | Yes | No | Stage Ⅱ | No | No | G3 | No | No |
| GSM4955426 | Female | 54 | Multiple | 56728 | 13 | Yes | No | Stage Ⅱ | No | No | G2 | Yes | No |
