# Supplementary material for: Combining bulk and single-cell RNA-sequencing data to develop an NK cell-related prognostic signature for hepatocellular carcinoma based on an integrated machine learning framework
Source: Eur J Med Res. 2023 Aug 30;28:306. doi: 10.1186/s40001-023-01300-6 (PMC10466881; doi:10.1186/s40001-023-01300-6)
Supplement: Supplementary file 6 — Additional file 6. The demographic and clinicopathological data of GSE91061 data set. [file 40001_2023_1300_MOESM6_ESM.docx]

Additional file 6. The demographic and clinicopathological data of GSE91061 dataset.

| Clinical characteristics | Number |
| --- | --- |
| **Therapeutic response** |  |
| CR/PR | 11 |
| SD/PD | 46 |
| **M stage** |  |
| M0 | 1 |
| M1A | 11 |
| M1B | 10 |
| M1C | 25 |
| Unknown | 10 |
| **Therapy regimen** |  |
| Anti-PD-1 therapy | 57 |
