# Supplementary material for: Combining bulk and single-cell RNA-sequencing data to develop an NK cell-related prognostic signature for hepatocellular carcinoma based on an integrated machine learning framework
Source: Eur J Med Res. 2023 Aug 30;28:306. doi: 10.1186/s40001-023-01300-6 (PMC10466881; doi:10.1186/s40001-023-01300-6)
Supplement: Supplementary file 7 — Additional file 7. The demographic and clinicopathological data of PRJEB23709 data set. [file 40001_2023_1300_MOESM7_ESM.docx]

Additional file 7. The demographic and clinicopathological data of PRJEB23709 dataset.

| Clinical characteristics | Number |
| --- | --- |
| **Age** |  |
| < 60 years | 35 |
| ≥ 60 years | 38 |
| **Gender** |  |
| Female | 26 |
| Male | 47 |
| **Therapeutic response** |  |
| CR/PR | 38 |
| SD/PD | 35 |
| **Therapy regimen** |  |
| Anti-PD-1 therapy | 41 |
| Anti-CTLA-4 + anti-PD-1 therapy | 32 |
