# Supplementary figures and images for: Combining bulk and single-cell RNA-sequencing data to develop an NK cell-related prognostic signature for hepatocellular carcinoma based on an integrated machine learning framework
Source: Eur J Med Res. 2023 Aug 30;28:306. doi: 10.1186/s40001-023-01300-6 (PMC10466881; doi:10.1186/s40001-023-01300-6)

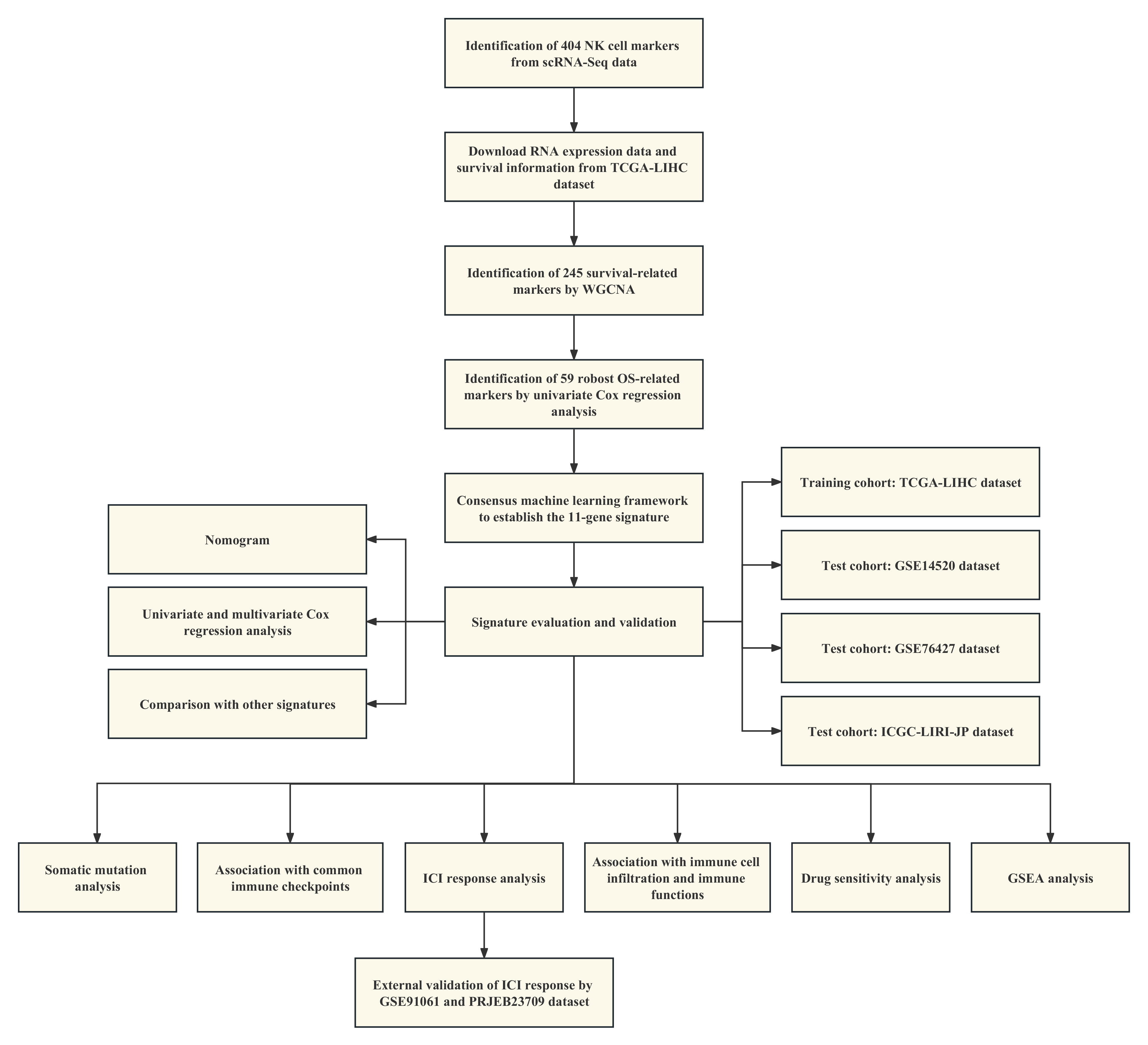

Supplement: Supplementary file 8 — Additional file 8. The workflow of the present study. [file 40001_2023_1300_MOESM8_ESM.tif]

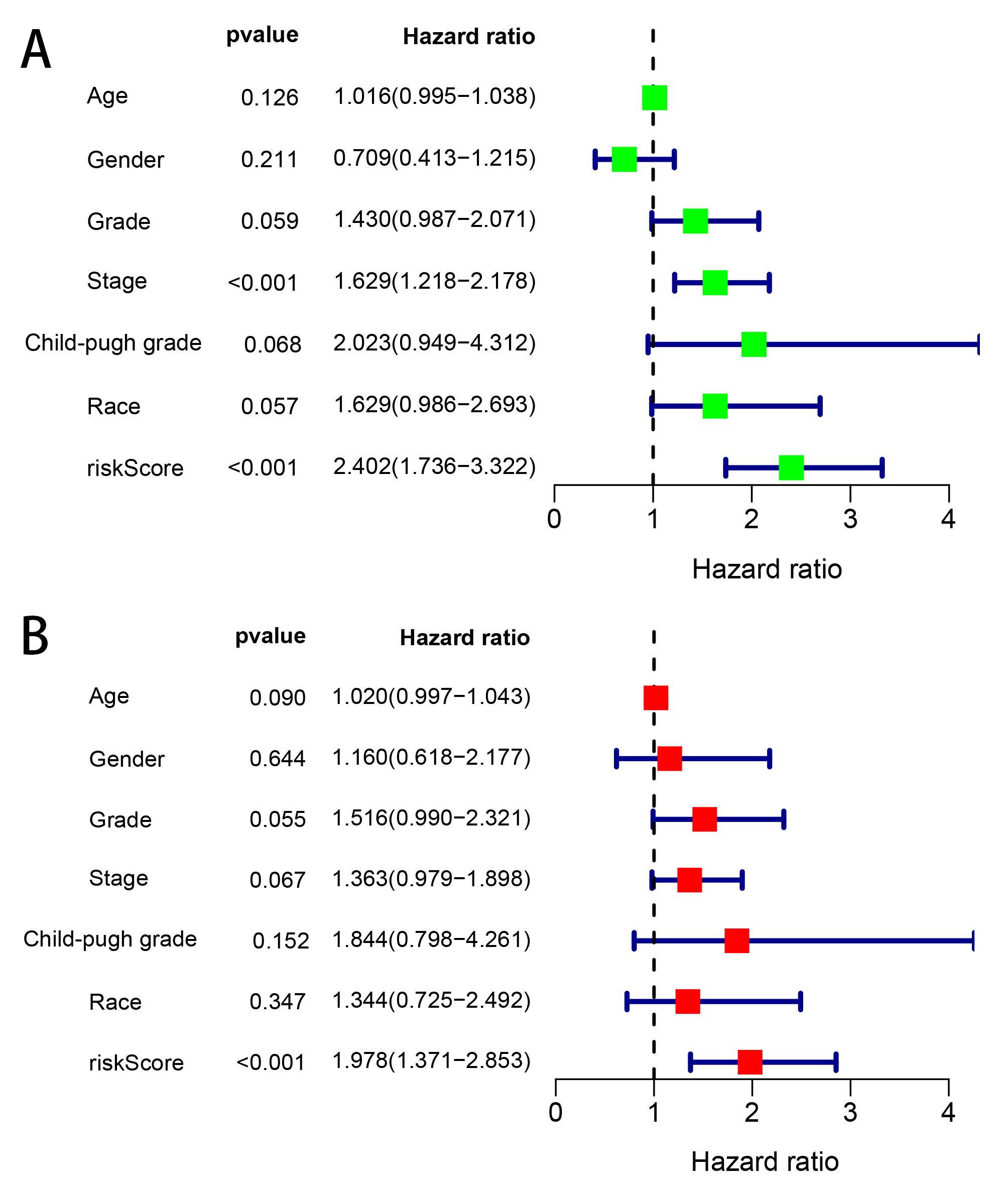

Supplement: Supplementary file 11 — Additional file 11. Cox regression analysis considering age, gender, race, clinical stage, Child–pugh grade, tumor grade and risk score in TCGA–LIHC data set. Univariate (A) and multivariate methods (B). [file 40001_2023_1300_MOESM11_ESM.tif]

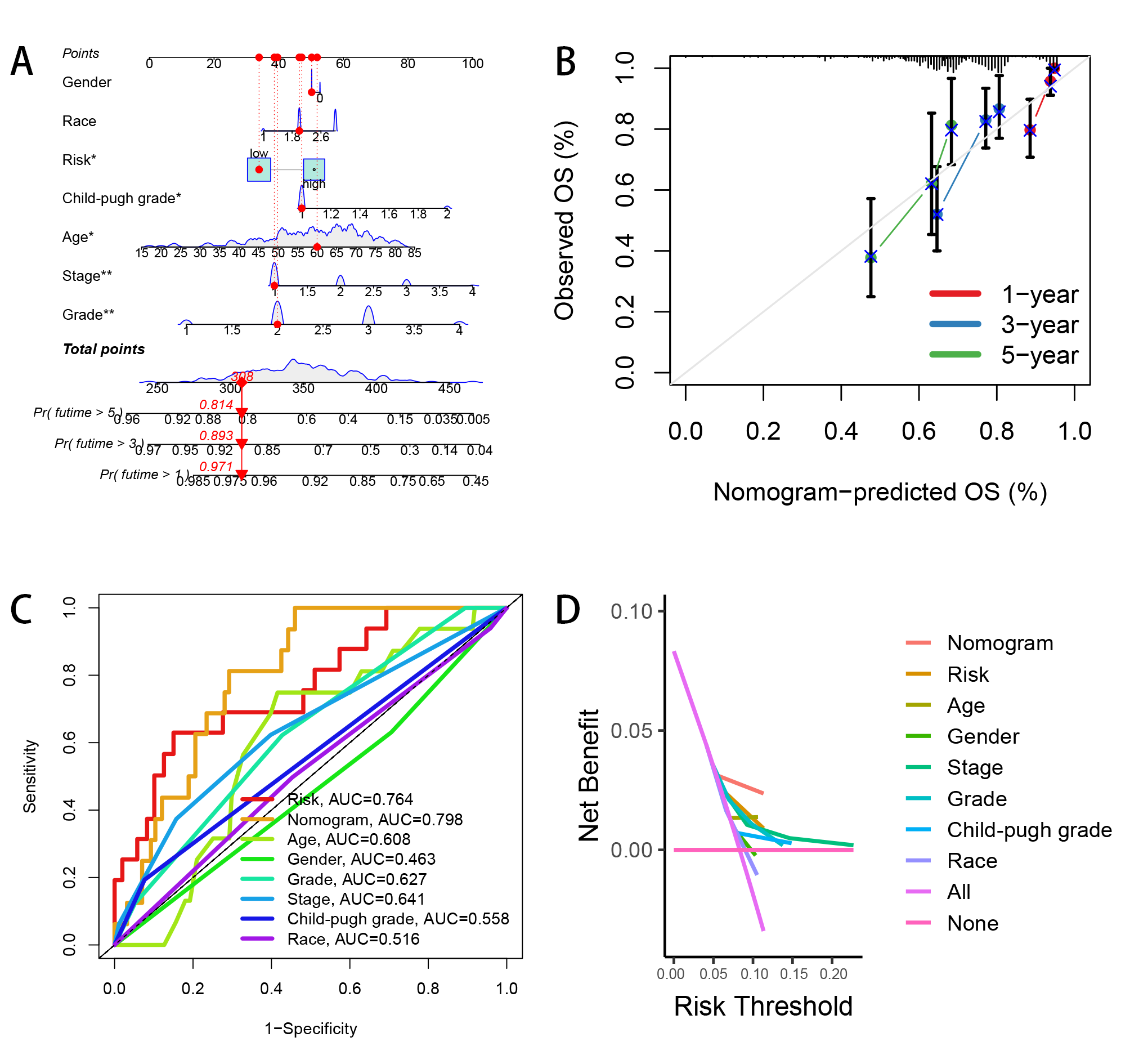

Supplement: Supplementary file 12 — Additional file 12. Nomogram establishment and performance assessment. (A) A nomogram considering race, age, gender, tumor grade, Child–pugh grade, risk score and clinical stage to predict 1-year, 3-year and 5-year survival rate of LIHC patients. Calibration plots (B) and clinical ROC curves (C) to illustrate the predictive efficacy of the nomogram. (D) Decision curves to reveal the potential clinical application valuation of the nomogram. [file 40001_2023_1300_MOESM12_ESM.tif]

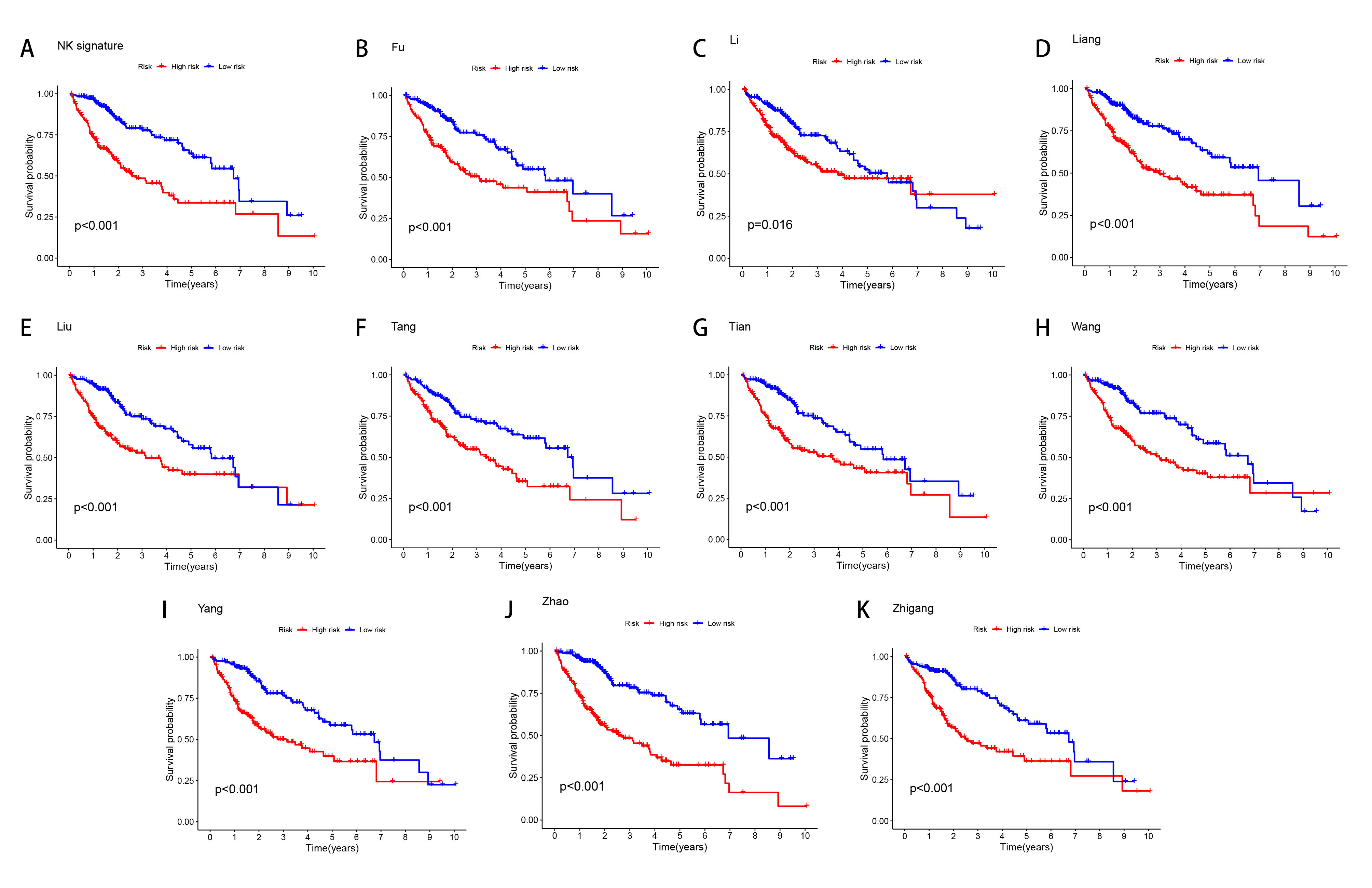

Supplement: Supplementary file 13 — Additional file 13. Survival curves of our signature and other 10 gene signature from previous publications. Our signature (A), Fu’s signature (B), Li’s signature (C), Liang’s signature (D), Liu’s signature (E), Tang’s signature (F), Tian’s signature (G), Wang’s signature (H), Yang’s signature (I), Zhao’s signature (J) and Zhigang’s signature (K). [file 40001_2023_1300_MOESM13_ESM.tif]

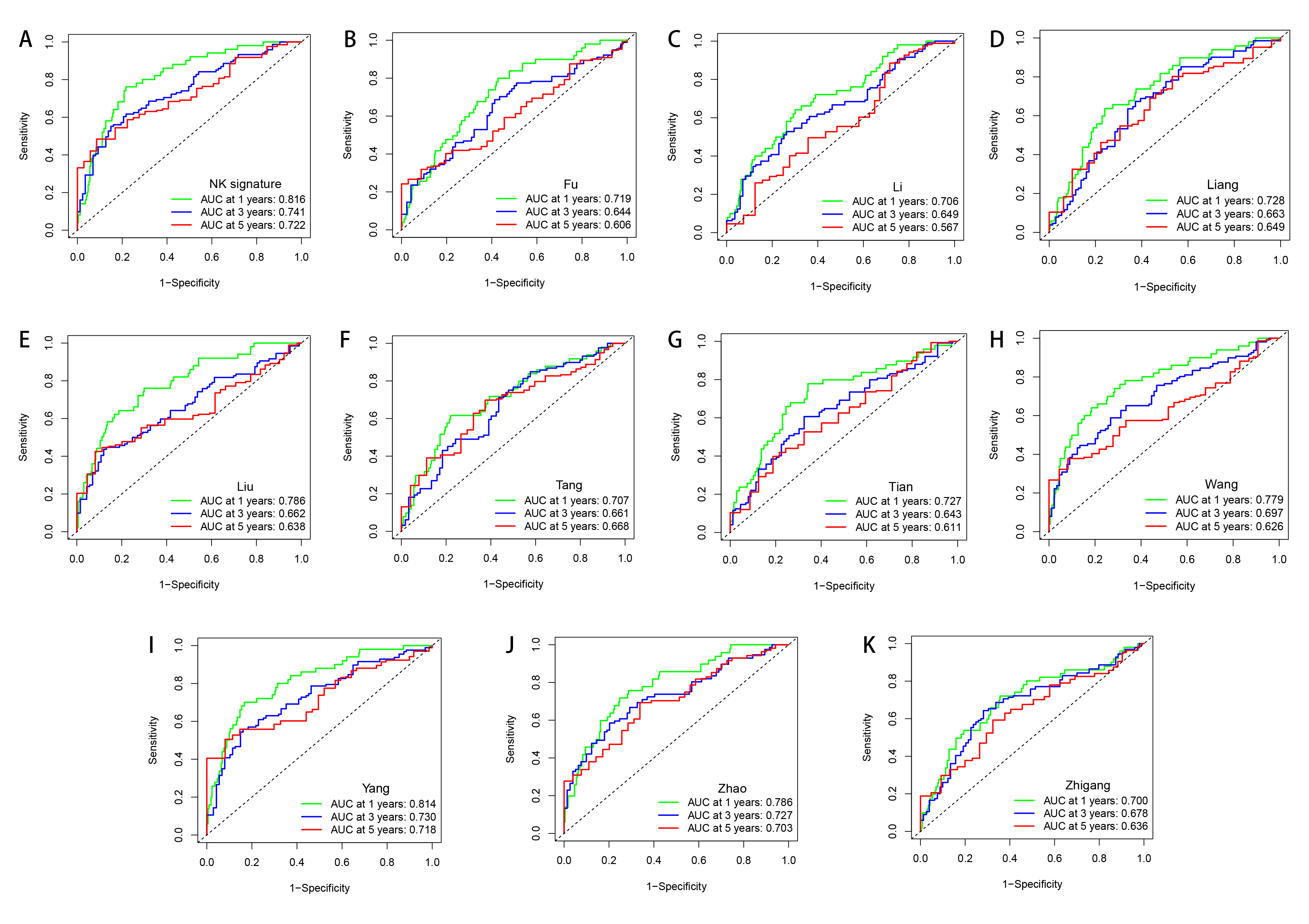

Supplement: Supplementary file 14 — Additional file 14. Time ROC curves of our signature and other 10 gene signature from previous publications. Our signature (A), Fu’s signature (B), Li’s signature (C), Liang’s signature (D), Liu’s signature (E), Tang’s signature (F), Tian’s signature (G), Wang’s signature (H), Yang’s signature (I), Zhao’s signature (J) and Zhigang’s signature (K). [file 40001_2023_1300_MOESM14_ESM.tif]
