# Supplementary material for: Combining bulk and single-cell RNA-sequencing data to develop an NK cell-related prognostic signature for hepatocellular carcinoma based on an integrated machine learning framework
Source: Eur J Med Res. 2023 Aug 30;28:306. doi: 10.1186/s40001-023-01300-6 (PMC10466881; doi:10.1186/s40001-023-01300-6)
Supplement: Supplementary file 9 — Additional file 9. The information of the 404 NK cell markers. [file 40001_2023_1300_MOESM9_ESM.docx]

Additional file 9. The information of the 404 NK cell markers.

| **Gene name** | **P-value** | **log2FC** | **pct.1** | **pct.2** | **Adjusted P-value** | **Cluster** |
| --- | --- | --- | --- | --- | --- | --- |
| GNLY | 0 | 2.71960187 | 0.627 | 0.279 | 0 | NK_cell |
| GZMB | 0 | 2.43160497 | 0.604 | 0.173 | 0 | NK_cell |
| CTSW | 0 | 2.39026235 | 0.798 | 0.172 | 0 | NK_cell |
| NKG7 | 0 | 2.14016303 | 0.904 | 0.497 | 0 | NK_cell |
| KLRB1 | 0 | 2.13744851 | 0.879 | 0.325 | 0 | NK_cell |
| KLRD1 | 0 | 2.05260471 | 0.784 | 0.227 | 0 | NK_cell |
| CCL3 | 0 | 1.93463815 | 0.748 | 0.368 | 0 | NK_cell |
| PRF1 | 0 | 1.768326 | 0.652 | 0.206 | 0 | NK_cell |
| KLRF1 | 0 | 1.76312682 | 0.574 | 0.097 | 0 | NK_cell |
| CLIC3 | 0 | 1.72587904 | 0.574 | 0.147 | 0 | NK_cell |
| XCL2 | 0 | 1.63722991 | 0.626 | 0.277 | 0 | NK_cell |
| GZMA | 0 | 1.62729261 | 0.787 | 0.362 | 0 | NK_cell |
| SPON2 | 0 | 1.61613138 | 0.569 | 0.256 | 0 | NK_cell |
| CD247 | 0 | 1.59745075 | 0.723 | 0.306 | 0 | NK_cell |
| AREG | 0 | 1.57281909 | 0.631 | 0.288 | 0 | NK_cell |
| CD160 | 0 | 1.56733158 | 0.453 | 0.092 | 0 | NK_cell |
| CMC1 | 0 | 1.54613217 | 0.65 | 0.319 | 0 | NK_cell |
| TRDC | 0 | 1.53261804 | 0.502 | 0.102 | 0 | NK_cell |
| ALOX5AP | 0 | 1.51360902 | 0.713 | 0.349 | 0 | NK_cell |
| CST7 | 0 | 1.48997724 | 0.846 | 0.531 | 0 | NK_cell |
| HOPX | 0 | 1.38463952 | 0.521 | 0.185 | 0 | NK_cell |
| CCL5 | 0 | 1.36930555 | 0.811 | 0.544 | 0 | NK_cell |
| GZMM | 0 | 1.35277941 | 0.66 | 0.29 | 0 | NK_cell |
| CCL4 | 0 | 1.34172398 | 0.859 | 0.591 | 0 | NK_cell |
| CD7 | 0 | 1.32000652 | 0.875 | 0.559 | 0 | NK_cell |
| MATK | 0 | 1.31945317 | 0.48 | 0.101 | 0 | NK_cell |
| FAM177A1 | 0 | 1.30623471 | 0.756 | 0.541 | 0 | NK_cell |
| APMAP | 0 | 1.21615297 | 0.574 | 0.235 | 0 | NK_cell |
| IFITM2 | 0 | 1.19281545 | 0.923 | 0.684 | 0 | NK_cell |
| GSTP1 | 0 | 1.16825717 | 0.764 | 0.429 | 0 | NK_cell |
| ADGRE5 | 0 | 1.12638083 | 0.78 | 0.482 | 0 | NK_cell |
| LITAF | 0 | 1.11799273 | 0.734 | 0.481 | 0 | NK_cell |
| HCST | 0 | 0.94873955 | 0.833 | 0.594 | 0 | NK_cell |
| PLAC8 | 0 | 0.94859047 | 0.525 | 0.177 | 0 | NK_cell |
| PFN1 | 0 | 0.89949068 | 0.969 | 0.865 | 0 | NK_cell |
| TYROBP | 0 | 0.88383528 | 0.853 | 0.291 | 0 | NK_cell |
| HLA-A | 0 | 0.83359374 | 0.995 | 0.95 | 0 | NK_cell |
| FCER1G | 0 | 0.58316479 | 0.765 | 0.256 | 0 | NK_cell |
| HLA-C | 0 | 0.57941029 | 0.996 | 0.95 | 0 | NK_cell |
| NFKBIA | 4.90E-305 | 9.08E-01 | 0.971 | 0.869 | 1.64E-300 | NK_cell |
| RPL31 | 5.85E-302 | 1.14E+00 | 0.896 | 0.794 | 1.96E-297 | NK_cell |
| LAT2 | 8.59E-297 | 1.09E+00 | 0.41 | 0.107 | 2.88E-292 | NK_cell |
| CDC42SE1 | 1.09E-296 | 9.17E-01 | 0.661 | 0.383 | 3.64E-292 | NK_cell |
| ITGB2 | 2.67E-294 | 9.16E-01 | 0.707 | 0.426 | 8.96E-290 | NK_cell |
| TMIGD2 | 1.96E-287 | 1.07E+00 | 0.369 | 0.07 | 6.59E-283 | NK_cell |
| CD63 | 2.77E-279 | 3.62E-01 | 0.768 | 0.493 | 9.30E-275 | NK_cell |
| TAGLN2 | 1.54E-270 | 7.24E-01 | 0.861 | 0.656 | 5.17E-266 | NK_cell |
| MAP3K8 | 1.18E-268 | 9.67E-01 | 0.62 | 0.342 | 3.96E-264 | NK_cell |
| RPS4Y1 | 2.25E-260 | 8.75E-01 | 0.525 | 0.2 | 7.54E-256 | NK_cell |
| GNG2 | 9.99E-260 | 1.02E+00 | 0.618 | 0.37 | 3.35E-255 | NK_cell |
| SRSF2 | 2.56E-259 | 7.15E-01 | 0.869 | 0.687 | 8.59E-255 | NK_cell |
| GZMH | 1.50E-256 | 1.50E+00 | 0.445 | 0.159 | 5.02E-252 | NK_cell |
| FGFBP2 | 3.02E-254 | 2.00E+00 | 0.346 | 0.076 | 1.01E-249 | NK_cell |
| CFL1 | 8.48E-252 | 5.78E-01 | 0.958 | 0.865 | 2.85E-247 | NK_cell |
| S1PR5 | 3.82E-251 | 1.10E+00 | 0.306 | 0.045 | 1.28E-246 | NK_cell |
| ARHGDIB | 3.17E-247 | 8.72E-01 | 0.887 | 0.749 | 1.06E-242 | NK_cell |
| RPL27A | 4.51E-245 | 1.13E+00 | 0.901 | 0.836 | 1.51E-240 | NK_cell |
| IL2RB | 4.96E-245 | 1.06E+00 | 0.408 | 0.137 | 1.66E-240 | NK_cell |
| PRR7 | 1.26E-244 | 1.01E+00 | 0.427 | 0.152 | 4.23E-240 | NK_cell |
| RPL23A | 5.12E-244 | 5.59E-01 | 0.989 | 0.927 | 1.72E-239 | NK_cell |
| UBB | 3.45E-242 | 5.37E-01 | 0.986 | 0.933 | 1.16E-237 | NK_cell |
| MYL6 | 9.66E-239 | 5.16E-01 | 0.962 | 0.875 | 3.24E-234 | NK_cell |
| CYBA | 2.86E-237 | 5.69E-01 | 0.93 | 0.778 | 9.60E-233 | NK_cell |
| NFKB1 | 7.32E-237 | 9.21E-01 | 0.555 | 0.295 | 2.45E-232 | NK_cell |
| RPL27 | 4.77E-234 | 6.12E-01 | 0.944 | 0.862 | 1.60E-229 | NK_cell |
| RPL3 | 1.55E-227 | 5.14E-01 | 0.989 | 0.936 | 5.21E-223 | NK_cell |
| EIF5A | 4.11E-227 | 7.95E-01 | 0.636 | 0.401 | 1.38E-222 | NK_cell |
| EFHD2 | 1.35E-223 | 9.26E-01 | 0.616 | 0.364 | 4.53E-219 | NK_cell |
| LSP1 | 4.54E-223 | 7.71E-01 | 0.662 | 0.43 | 1.52E-218 | NK_cell |
| FKBP11 | 4.83E-223 | 8.94E-01 | 0.403 | 0.139 | 1.62E-218 | NK_cell |
| KLRC1 | 1.39E-218 | 1.18E+00 | 0.326 | 0.077 | 4.65E-214 | NK_cell |
| BIN2 | 2.58E-218 | 8.13E-01 | 0.508 | 0.254 | 8.66E-214 | NK_cell |
| IL2RG | 2.88E-218 | 6.69E-01 | 0.739 | 0.509 | 9.67E-214 | NK_cell |
| CHST12 | 3.32E-216 | 8.79E-01 | 0.408 | 0.151 | 1.11E-211 | NK_cell |
| PDIA3 | 6.66E-216 | 6.65E-01 | 0.746 | 0.537 | 2.24E-211 | NK_cell |
| RPS17 | 4.78E-215 | 9.63E-01 | 0.552 | 0.318 | 1.60E-210 | NK_cell |
| RPL21 | 2.09E-214 | 4.67E-01 | 0.994 | 0.953 | 6.99E-210 | NK_cell |
| CD69 | 2.70E-213 | 7.69E-01 | 0.886 | 0.703 | 9.04E-209 | NK_cell |
| IFITM1 | 6.40E-211 | 9.34E-01 | 0.456 | 0.204 | 2.15E-206 | NK_cell |
| STAT4 | 3.41E-210 | 8.35E-01 | 0.523 | 0.27 | 1.14E-205 | NK_cell |
| SRGN | 1.47E-209 | 6.24E-01 | 0.989 | 0.922 | 4.92E-205 | NK_cell |
| CD53 | 1.66E-208 | 6.30E-01 | 0.793 | 0.607 | 5.57E-204 | NK_cell |
| TGFB1 | 4.10E-207 | 7.06E-01 | 0.656 | 0.414 | 1.37E-202 | NK_cell |
| TMSB4X | 2.90E-205 | 5.95E-01 | 0.999 | 0.978 | 9.71E-201 | NK_cell |
| ZBTB16 | 1.32E-202 | 8.32E-01 | 0.368 | 0.121 | 4.41E-198 | NK_cell |
| VPS37B | 1.03E-201 | 8.22E-01 | 0.5 | 0.25 | 3.46E-197 | NK_cell |
| TLE1 | 5.83E-198 | 9.32E-01 | 0.323 | 0.088 | 1.96E-193 | NK_cell |
| RPLP2 | 1.51E-192 | 5.02E-01 | 0.992 | 0.943 | 5.07E-188 | NK_cell |
| RPL37A | 1.28E-186 | 4.50E-01 | 0.977 | 0.911 | 4.30E-182 | NK_cell |
| HSPA5 | 2.92E-186 | 6.52E-01 | 0.892 | 0.744 | 9.81E-182 | NK_cell |
| SLC16A3 | 3.10E-184 | 9.22E-01 | 0.334 | 0.112 | 1.04E-179 | NK_cell |
| RPS27A | 4.22E-184 | 3.06E-01 | 0.998 | 0.97 | 1.41E-179 | NK_cell |
| RPL13A | 5.52E-175 | 5.72E-01 | 0.985 | 0.932 | 1.85E-170 | NK_cell |
| RPS6 | 9.35E-174 | 4.84E-01 | 0.989 | 0.934 | 3.14E-169 | NK_cell |
| RPL7 | 1.16E-173 | 6.31E-01 | 0.935 | 0.877 | 3.91E-169 | NK_cell |
| RAC2 | 4.18E-171 | 5.95E-01 | 0.756 | 0.572 | 1.40E-166 | NK_cell |
| AOAH | 2.80E-170 | 7.33E-01 | 0.311 | 0.099 | 9.38E-166 | NK_cell |
| LDHA | 7.61E-170 | 6.84E-01 | 0.829 | 0.693 | 2.55E-165 | NK_cell |
| APOBEC3G | 1.75E-169 | 7.81E-01 | 0.467 | 0.249 | 5.89E-165 | NK_cell |
| ANXA1 | 6.38E-169 | 4.97E-01 | 0.733 | 0.451 | 2.14E-164 | NK_cell |
| IER2 | 6.44E-169 | 6.95E-01 | 0.944 | 0.823 | 2.16E-164 | NK_cell |
| PAXX | 2.94E-168 | 6.44E-01 | 0.556 | 0.331 | 9.86E-164 | NK_cell |
| RPS3 | 6.14E-168 | 3.50E-01 | 0.996 | 0.947 | 2.06E-163 | NK_cell |
| IRF8 | 6.03E-167 | 8.49E-01 | 0.411 | 0.192 | 2.02E-162 | NK_cell |
| HSP90B1 | 6.19E-167 | 6.37E-01 | 0.654 | 0.45 | 2.08E-162 | NK_cell |
| REL | 3.74E-166 | 6.18E-01 | 0.812 | 0.627 | 1.26E-161 | NK_cell |
| RPS20 | 2.53E-165 | 5.98E-01 | 0.936 | 0.884 | 8.49E-161 | NK_cell |
| ID2 | 7.08E-165 | 5.44E-01 | 0.927 | 0.784 | 2.37E-160 | NK_cell |
| MAFF | 1.54E-162 | 7.55E-01 | 0.491 | 0.276 | 5.15E-158 | NK_cell |
| RARRES3 | 2.38E-158 | 6.04E-01 | 0.66 | 0.455 | 7.98E-154 | NK_cell |
| POLR2L | 2.08E-156 | 5.79E-01 | 0.657 | 0.477 | 6.98E-152 | NK_cell |
| RPL35 | 5.92E-154 | 4.53E-01 | 0.957 | 0.906 | 1.99E-149 | NK_cell |
| SLC15A4 | 7.75E-154 | 7.85E-01 | 0.251 | 0.064 | 2.60E-149 | NK_cell |
| RPL15 | 1.38E-153 | 3.47E-01 | 0.992 | 0.945 | 4.63E-149 | NK_cell |
| TXK | 2.88E-148 | 7.27E-01 | 0.285 | 0.092 | 9.67E-144 | NK_cell |
| NDUFB2 | 6.34E-148 | 5.20E-01 | 0.627 | 0.428 | 2.13E-143 | NK_cell |
| CLIC1 | 1.07E-147 | 4.66E-01 | 0.842 | 0.678 | 3.60E-143 | NK_cell |
| BHLHE40 | 6.30E-147 | 6.92E-01 | 0.59 | 0.377 | 2.11E-142 | NK_cell |
| BZW1 | 1.65E-142 | 5.49E-01 | 0.683 | 0.504 | 5.54E-138 | NK_cell |
| MAPK1 | 3.26E-142 | 7.23E-01 | 0.389 | 0.194 | 1.09E-137 | NK_cell |
| RELB | 1.02E-140 | 7.39E-01 | 0.37 | 0.176 | 3.40E-136 | NK_cell |
| NINJ1 | 2.41E-140 | 5.17E-01 | 0.529 | 0.301 | 8.09E-136 | NK_cell |
| NCR3 | 2.49E-140 | 6.57E-01 | 0.315 | 0.117 | 8.33E-136 | NK_cell |
| SYAP1 | 4.33E-139 | 6.94E-01 | 0.602 | 0.425 | 1.45E-134 | NK_cell |
| MAP2K3 | 2.51E-138 | 6.96E-01 | 0.455 | 0.264 | 8.42E-134 | NK_cell |
| BSG | 3.38E-136 | 4.88E-01 | 0.525 | 0.329 | 1.13E-131 | NK_cell |
| GZMK | 3.11E-135 | 9.91E-01 | 0.492 | 0.292 | 1.04E-130 | NK_cell |
| ZC3H12A | 1.83E-134 | 6.12E-01 | 0.568 | 0.371 | 6.14E-130 | NK_cell |
| C12orf75 | 2.96E-133 | 7.85E-01 | 0.285 | 0.103 | 9.92E-129 | NK_cell |
| RPS29 | 7.08E-132 | 4.79E-01 | 0.982 | 0.933 | 2.37E-127 | NK_cell |
| EIF3G | 7.78E-132 | 5.52E-01 | 0.642 | 0.476 | 2.61E-127 | NK_cell |
| RPS27 | 3.81E-129 | 3.91E-01 | 0.997 | 0.962 | 1.28E-124 | NK_cell |
| CALR | 1.08E-127 | 4.91E-01 | 0.786 | 0.653 | 3.63E-123 | NK_cell |
| XCL1 | 2.36E-126 | 1.17E+00 | 0.42 | 0.219 | 7.90E-122 | NK_cell |
| TMA7 | 5.80E-126 | 4.19E-01 | 0.846 | 0.712 | 1.94E-121 | NK_cell |
| OSTF1 | 4.10E-125 | 5.42E-01 | 0.503 | 0.315 | 1.38E-120 | NK_cell |
| GPR65 | 3.74E-124 | 6.10E-01 | 0.383 | 0.196 | 1.25E-119 | NK_cell |
| AC020916.1 | 8.63E-124 | 7.22E-01 | 0.497 | 0.305 | 2.89E-119 | NK_cell |
| IFNGR1 | 1.15E-123 | 5.99E-01 | 0.412 | 0.224 | 3.85E-119 | NK_cell |
| HNRNPA1 | 1.20E-123 | 4.35E-01 | 0.885 | 0.793 | 4.03E-119 | NK_cell |
| SYTL3 | 2.77E-122 | 5.58E-01 | 0.525 | 0.323 | 9.28E-118 | NK_cell |
| GYPC | 1.13E-121 | 5.22E-01 | 0.662 | 0.498 | 3.78E-117 | NK_cell |
| FOSL2 | 3.60E-121 | 6.52E-01 | 0.481 | 0.302 | 1.21E-116 | NK_cell |
| SLA2 | 3.43E-120 | 6.64E-01 | 0.255 | 0.087 | 1.15E-115 | NK_cell |
| TAPBP | 2.05E-119 | 5.21E-01 | 0.574 | 0.4 | 6.88E-115 | NK_cell |
| HLA-E | 9.50E-119 | 3.36E-01 | 0.983 | 0.906 | 3.19E-114 | NK_cell |
| RBM39 | 1.43E-118 | 3.93E-01 | 0.909 | 0.79 | 4.81E-114 | NK_cell |
| AKNA | 8.20E-118 | 6.34E-01 | 0.393 | 0.212 | 2.75E-113 | NK_cell |
| PIK3R1 | 1.51E-116 | 5.69E-01 | 0.535 | 0.341 | 5.08E-112 | NK_cell |
| EEF2 | 5.59E-116 | 4.52E-01 | 0.809 | 0.693 | 1.87E-111 | NK_cell |
| CORO1A | 2.05E-114 | 5.15E-01 | 0.714 | 0.551 | 6.89E-110 | NK_cell |
| ARPC2 | 7.59E-112 | 3.92E-01 | 0.869 | 0.74 | 2.55E-107 | NK_cell |
| RAP1B | 1.30E-111 | 4.33E-01 | 0.723 | 0.542 | 4.37E-107 | NK_cell |
| PTMA | 3.06E-110 | 2.62E-01 | 0.995 | 0.966 | 1.03E-105 | NK_cell |
| CCDC107 | 4.30E-110 | 5.92E-01 | 0.476 | 0.297 | 1.44E-105 | NK_cell |
| PSME1 | 1.64E-107 | 3.97E-01 | 0.828 | 0.698 | 5.48E-103 | NK_cell |
| DDX3Y | 3.29E-107 | 6.43E-01 | 0.268 | 0.105 | 1.10E-102 | NK_cell |
| WIPF1 | 6.06E-107 | 5.30E-01 | 0.529 | 0.366 | 2.03E-102 | NK_cell |
| YPEL5 | 4.59E-106 | 4.33E-01 | 0.87 | 0.754 | 1.54E-101 | NK_cell |
| LCP1 | 2.20E-105 | 4.14E-01 | 0.613 | 0.432 | 7.36E-101 | NK_cell |
| METRNL | 1.34E-104 | 5.36E-01 | 0.56 | 0.37 | 4.51E-100 | NK_cell |
| SNRPB | 5.45E-104 | 4.62E-01 | 0.636 | 0.481 | 1.83E-99 | NK_cell |
| EVL | 1.62E-101 | 4.63E-01 | 0.673 | 0.523 | 5.43E-97 | NK_cell |
| 7-Sep | 2.13E-100 | 4.55E-01 | 0.69 | 0.55 | 7.14E-96 | NK_cell |
| SQSTM1 | 3.42E-100 | 5.10E-01 | 0.694 | 0.546 | 1.15E-95 | NK_cell |
| SH2D2A | 4.33E-100 | 5.69E-01 | 0.396 | 0.225 | 1.45E-95 | NK_cell |
| CEBPD | 3.79E-99 | 2.94E-01 | 0.395 | 0.213 | 1.27E-94 | NK_cell |
| ITGB7 | 7.19E-99 | 5.37E-01 | 0.251 | 0.098 | 2.41E-94 | NK_cell |
| CRIP1 | 2.41E-98 | 7.29E-01 | 0.35 | 0.193 | 8.08E-94 | NK_cell |
| RAB5IF | 3.57E-98 | 5.50E-01 | 0.454 | 0.294 | 1.20E-93 | NK_cell |
| ANKRD28 | 5.68E-98 | 5.44E-01 | 0.331 | 0.165 | 1.90E-93 | NK_cell |
| CD99 | 3.40E-97 | 4.72E-01 | 0.729 | 0.566 | 1.14E-92 | NK_cell |
| PPP1CA | 1.36E-94 | 5.01E-01 | 0.488 | 0.331 | 4.55E-90 | NK_cell |
| RPS18 | 1.87E-94 | 3.52E-01 | 0.995 | 0.96 | 6.26E-90 | NK_cell |
| FCGR3A | 4.33E-93 | 3.39E-01 | 0.39 | 0.199 | 1.45E-88 | NK_cell |
| TSEN54 | 8.62E-93 | 4.92E-01 | 0.256 | 0.107 | 2.89E-88 | NK_cell |
| DBI | 2.10E-92 | 3.43E-01 | 0.676 | 0.532 | 7.05E-88 | NK_cell |
| ACTG1 | 3.85E-92 | 4.11E-01 | 0.965 | 0.891 | 1.29E-87 | NK_cell |
| GLIPR2 | 2.82E-90 | 5.04E-01 | 0.284 | 0.137 | 9.47E-86 | NK_cell |
| HMGN3 | 3.29E-90 | 4.43E-01 | 0.379 | 0.224 | 1.10E-85 | NK_cell |
| HNRNPC | 9.98E-88 | 4.21E-01 | 0.746 | 0.632 | 3.35E-83 | NK_cell |
| GUK1 | 4.09E-86 | 3.51E-01 | 0.73 | 0.604 | 1.37E-81 | NK_cell |
| SKP1 | 4.43E-86 | 3.53E-01 | 0.815 | 0.715 | 1.49E-81 | NK_cell |
| RHOF | 5.55E-86 | 5.10E-01 | 0.433 | 0.28 | 1.86E-81 | NK_cell |
| PRDX5 | 2.46E-85 | 3.79E-01 | 0.516 | 0.362 | 8.24E-81 | NK_cell |
| CCL3L1 | 2.78E-85 | 7.68E-01 | 0.476 | 0.3 | 9.31E-81 | NK_cell |
| ENSA | 5.17E-85 | 4.16E-01 | 0.574 | 0.431 | 1.73E-80 | NK_cell |
| ABHD17A | 1.32E-84 | 6.08E-01 | 0.443 | 0.302 | 4.42E-80 | NK_cell |
| AES | 3.10E-84 | 5.01E-01 | 0.596 | 0.461 | 1.04E-79 | NK_cell |
| TWISTNB | 5.91E-84 | 5.96E-01 | 0.327 | 0.182 | 1.98E-79 | NK_cell |
| TNFSF14 | 1.15E-83 | 5.04E-01 | 0.329 | 0.172 | 3.84E-79 | NK_cell |
| UPP1 | 2.36E-83 | 5.19E-01 | 0.401 | 0.253 | 7.91E-79 | NK_cell |
| CCDC12 | 2.06E-82 | 4.53E-01 | 0.429 | 0.282 | 6.90E-78 | NK_cell |
| HNRNPK | 1.47E-81 | 3.68E-01 | 0.716 | 0.593 | 4.94E-77 | NK_cell |
| MYDGF | 3.23E-81 | 3.72E-01 | 0.4 | 0.252 | 1.08E-76 | NK_cell |
| CHCHD2 | 4.11E-81 | 3.25E-01 | 0.835 | 0.734 | 1.38E-76 | NK_cell |
| MANF | 6.05E-81 | 4.77E-01 | 0.304 | 0.161 | 2.03E-76 | NK_cell |
| POLR2K | 2.12E-80 | 4.62E-01 | 0.494 | 0.352 | 7.12E-76 | NK_cell |
| IGKC | 8.93E-80 | 2.83E+00 | 0.289 | 0.429 | 3.00E-75 | NK_cell |
| MYADM | 1.52E-79 | 3.91E-01 | 0.611 | 0.448 | 5.10E-75 | NK_cell |
| RHOC | 2.53E-78 | 4.57E-01 | 0.516 | 0.369 | 8.48E-74 | NK_cell |
| BIRC2 | 2.62E-78 | 4.72E-01 | 0.396 | 0.256 | 8.78E-74 | NK_cell |
| PABPC1 | 7.49E-77 | 3.64E-01 | 0.903 | 0.814 | 2.51E-72 | NK_cell |
| ATP1B3 | 1.25E-75 | 6.79E-01 | 0.644 | 0.542 | 4.18E-71 | NK_cell |
| IRF1 | 1.57E-75 | 3.96E-01 | 0.846 | 0.702 | 5.26E-71 | NK_cell |
| THAP2 | 3.97E-75 | 6.17E-01 | 0.346 | 0.212 | 1.33E-70 | NK_cell |
| DHRS7 | 1.31E-73 | 4.15E-01 | 0.487 | 0.342 | 4.39E-69 | NK_cell |
| SELENOS | 1.40E-73 | 4.78E-01 | 0.377 | 0.242 | 4.70E-69 | NK_cell |
| ZFP36 | 1.63E-72 | 3.43E-01 | 0.96 | 0.865 | 5.46E-68 | NK_cell |
| RNF125 | 2.73E-72 | 4.20E-01 | 0.345 | 0.198 | 9.17E-68 | NK_cell |
| AC245297.3 | 3.14E-72 | 4.45E-01 | 0.369 | 0.231 | 1.05E-67 | NK_cell |
| RAB8B | 5.53E-72 | 4.72E-01 | 0.404 | 0.266 | 1.85E-67 | NK_cell |
| KMT2E | 5.78E-71 | 3.96E-01 | 0.623 | 0.492 | 1.94E-66 | NK_cell |
| UBE2F | 7.13E-71 | 4.98E-01 | 0.283 | 0.157 | 2.39E-66 | NK_cell |
| RBM3 | 1.69E-70 | 3.67E-01 | 0.676 | 0.549 | 5.66E-66 | NK_cell |
| ICAM1 | 2.99E-70 | 4.16E-01 | 0.281 | 0.147 | 1.00E-65 | NK_cell |
| ARHGAP9 | 1.55E-69 | 4.20E-01 | 0.447 | 0.309 | 5.19E-65 | NK_cell |
| SERBP1 | 3.22E-69 | 3.66E-01 | 0.582 | 0.462 | 1.08E-64 | NK_cell |
| SIRT2 | 4.96E-69 | 4.53E-01 | 0.289 | 0.161 | 1.66E-64 | NK_cell |
| PSME2 | 5.69E-68 | 2.90E-01 | 0.67 | 0.548 | 1.91E-63 | NK_cell |
| SSR4 | 7.76E-68 | 4.14E-01 | 0.74 | 0.638 | 2.60E-63 | NK_cell |
| SELENOK | 1.49E-67 | 3.32E-01 | 0.783 | 0.665 | 4.99E-63 | NK_cell |
| UBE2L3 | 6.23E-67 | 3.79E-01 | 0.43 | 0.299 | 2.09E-62 | NK_cell |
| FMNL1 | 1.63E-66 | 4.41E-01 | 0.441 | 0.311 | 5.48E-62 | NK_cell |
| TPST2 | 2.23E-66 | 4.65E-01 | 0.278 | 0.157 | 7.49E-62 | NK_cell |
| POLR2G | 6.61E-65 | 4.28E-01 | 0.337 | 0.213 | 2.22E-60 | NK_cell |
| C4orf3 | 6.97E-65 | 3.22E-01 | 0.528 | 0.404 | 2.34E-60 | NK_cell |
| FBL | 1.53E-64 | 4.06E-01 | 0.301 | 0.174 | 5.14E-60 | NK_cell |
| LPIN1 | 1.94E-64 | 4.42E-01 | 0.318 | 0.189 | 6.51E-60 | NK_cell |
| ISG20 | 2.75E-64 | 5.03E-01 | 0.752 | 0.668 | 9.22E-60 | NK_cell |
| INPP5D | 6.03E-64 | 4.48E-01 | 0.27 | 0.149 | 2.02E-59 | NK_cell |
| SNRPD2 | 6.52E-64 | 3.54E-01 | 0.644 | 0.536 | 2.19E-59 | NK_cell |
| STARD3NL | 1.28E-63 | 4.69E-01 | 0.267 | 0.149 | 4.30E-59 | NK_cell |
| RPS9 | 3.94E-62 | 2.67E-01 | 0.977 | 0.934 | 1.32E-57 | NK_cell |
| C1orf56 | 6.13E-62 | 5.36E-01 | 0.277 | 0.159 | 2.06E-57 | NK_cell |
| PCBP2 | 5.02E-61 | 3.46E-01 | 0.627 | 0.517 | 1.68E-56 | NK_cell |
| CMTM3 | 6.05E-61 | 4.04E-01 | 0.278 | 0.159 | 2.03E-56 | NK_cell |
| PPIB | 7.09E-61 | 2.78E-01 | 0.669 | 0.563 | 2.38E-56 | NK_cell |
| PHF20 | 7.93E-61 | 3.55E-01 | 0.583 | 0.462 | 2.66E-56 | NK_cell |
| SUN2 | 9.21E-61 | 4.69E-01 | 0.323 | 0.207 | 3.09E-56 | NK_cell |
| TES | 2.11E-60 | 4.26E-01 | 0.285 | 0.169 | 7.08E-56 | NK_cell |
| HSH2D | 2.43E-60 | 4.66E-01 | 0.25 | 0.138 | 8.14E-56 | NK_cell |
| MSN | 3.40E-60 | 3.39E-01 | 0.521 | 0.387 | 1.14E-55 | NK_cell |
| P4HB | 4.18E-60 | 3.22E-01 | 0.421 | 0.293 | 1.40E-55 | NK_cell |
| RALY | 4.64E-60 | 3.99E-01 | 0.41 | 0.293 | 1.55E-55 | NK_cell |
| TPM4 | 5.59E-60 | 4.14E-01 | 0.366 | 0.243 | 1.88E-55 | NK_cell |
| MIF | 6.84E-60 | 4.20E-01 | 0.406 | 0.285 | 2.29E-55 | NK_cell |
| NFKB2 | 1.10E-59 | 4.23E-01 | 0.379 | 0.258 | 3.70E-55 | NK_cell |
| LAMP1 | 1.14E-59 | 3.69E-01 | 0.409 | 0.287 | 3.84E-55 | NK_cell |
| NR4A2 | 1.16E-59 | 4.04E-01 | 0.747 | 0.627 | 3.88E-55 | NK_cell |
| SEC61B | 6.37E-59 | 2.75E-01 | 0.613 | 0.497 | 2.14E-54 | NK_cell |
| EMD | 1.06E-58 | 4.02E-01 | 0.394 | 0.271 | 3.56E-54 | NK_cell |
| CDK2AP2 | 6.78E-58 | 3.92E-01 | 0.363 | 0.243 | 2.27E-53 | NK_cell |
| GPSM3 | 1.10E-57 | 3.61E-01 | 0.612 | 0.492 | 3.70E-53 | NK_cell |
| NFKBIZ | 9.73E-57 | 3.22E-01 | 0.497 | 0.354 | 3.26E-52 | NK_cell |
| ANXA6 | 2.29E-56 | 3.77E-01 | 0.389 | 0.266 | 7.67E-52 | NK_cell |
| PLA2G16 | 4.65E-56 | 3.31E-01 | 0.26 | 0.144 | 1.56E-51 | NK_cell |
| AC004687.1 | 5.58E-56 | 4.34E-01 | 0.315 | 0.198 | 1.87E-51 | NK_cell |
| MYO1F | 5.63E-56 | 4.03E-01 | 0.267 | 0.157 | 1.89E-51 | NK_cell |
| ENO1 | 7.56E-56 | 3.55E-01 | 0.589 | 0.487 | 2.54E-51 | NK_cell |
| CCND2 | 1.06E-55 | 3.71E-01 | 0.358 | 0.231 | 3.54E-51 | NK_cell |
| CLDND1 | 1.18E-55 | 4.52E-01 | 0.313 | 0.199 | 3.97E-51 | NK_cell |
| ARHGDIA | 2.09E-55 | 3.81E-01 | 0.516 | 0.406 | 7.01E-51 | NK_cell |
| JAK1 | 2.45E-55 | 3.50E-01 | 0.59 | 0.476 | 8.20E-51 | NK_cell |
| BRAF | 2.74E-55 | 4.53E-01 | 0.282 | 0.171 | 9.20E-51 | NK_cell |
| UBE2D3 | 3.14E-55 | 2.81E-01 | 0.769 | 0.661 | 1.05E-50 | NK_cell |
| CD164 | 4.04E-55 | 3.34E-01 | 0.475 | 0.364 | 1.35E-50 | NK_cell |
| RPL36AL | 4.27E-55 | 2.58E-01 | 0.907 | 0.833 | 1.43E-50 | NK_cell |
| DUSP10 | 2.57E-54 | 3.58E-01 | 0.35 | 0.226 | 8.61E-50 | NK_cell |
| CHMP1B | 3.31E-54 | 3.45E-01 | 0.577 | 0.451 | 1.11E-49 | NK_cell |
| PRELID1 | 3.39E-54 | 2.80E-01 | 0.479 | 0.352 | 1.14E-49 | NK_cell |
| JMJD6 | 6.04E-54 | 3.89E-01 | 0.418 | 0.3 | 2.03E-49 | NK_cell |
| ARL4A | 6.79E-54 | 4.10E-01 | 0.475 | 0.358 | 2.28E-49 | NK_cell |
| SKIL | 7.68E-54 | 3.98E-01 | 0.338 | 0.224 | 2.58E-49 | NK_cell |
| RPL4 | 1.06E-53 | 2.96E-01 | 0.884 | 0.826 | 3.55E-49 | NK_cell |
| GADD45B | 1.12E-53 | 4.20E-01 | 0.929 | 0.839 | 3.76E-49 | NK_cell |
| ZAP70 | 1.56E-53 | 4.03E-01 | 0.276 | 0.165 | 5.22E-49 | NK_cell |
| XBP1 | 2.47E-53 | 3.47E-01 | 0.517 | 0.393 | 8.30E-49 | NK_cell |
| CSNK1D | 4.63E-53 | 3.83E-01 | 0.4 | 0.289 | 1.55E-48 | NK_cell |
| OFD1 | 1.92E-52 | 4.56E-01 | 0.43 | 0.312 | 6.43E-48 | NK_cell |
| PTPN7 | 2.08E-52 | 3.70E-01 | 0.426 | 0.299 | 6.96E-48 | NK_cell |
| CD96 | 3.47E-52 | 3.45E-01 | 0.515 | 0.386 | 1.16E-47 | NK_cell |
| CAPZB | 1.18E-51 | 3.03E-01 | 0.576 | 0.464 | 3.95E-47 | NK_cell |
| CYSTM1 | 1.19E-51 | 4.30E-01 | 0.257 | 0.152 | 3.99E-47 | NK_cell |
| TAP1 | 8.49E-51 | 3.56E-01 | 0.423 | 0.312 | 2.85E-46 | NK_cell |
| TRAPPC1 | 2.69E-50 | 3.60E-01 | 0.42 | 0.312 | 9.01E-46 | NK_cell |
| CHMP4A | 3.00E-50 | 3.54E-01 | 0.411 | 0.303 | 1.01E-45 | NK_cell |
| NFE2L2 | 4.98E-50 | 3.67E-01 | 0.413 | 0.298 | 1.67E-45 | NK_cell |
| ICAM3 | 2.15E-49 | 3.29E-01 | 0.526 | 0.415 | 7.22E-45 | NK_cell |
| NFKBIB | 8.06E-49 | 4.03E-01 | 0.278 | 0.176 | 2.70E-44 | NK_cell |
| OTULIN | 3.71E-48 | 5.16E-01 | 0.346 | 0.244 | 1.24E-43 | NK_cell |
| IFNG | 6.51E-48 | 7.74E-01 | 0.464 | 0.348 | 2.18E-43 | NK_cell |
| COPS9 | 8.74E-48 | 2.81E-01 | 0.455 | 0.345 | 2.93E-43 | NK_cell |
| EIF3I | 2.21E-47 | 3.36E-01 | 0.413 | 0.31 | 7.40E-43 | NK_cell |
| BUD31 | 4.00E-47 | 3.05E-01 | 0.49 | 0.385 | 1.34E-42 | NK_cell |
| SERTAD1 | 4.19E-47 | 2.87E-01 | 0.745 | 0.638 | 1.40E-42 | NK_cell |
| ARID4B | 1.01E-46 | 3.05E-01 | 0.614 | 0.509 | 3.38E-42 | NK_cell |
| RPL17 | 1.45E-46 | 3.45E-01 | 0.5 | 0.4 | 4.85E-42 | NK_cell |
| HNRNPM | 1.93E-46 | 3.35E-01 | 0.457 | 0.355 | 6.48E-42 | NK_cell |
| KDM6B | 2.77E-46 | 3.75E-01 | 0.549 | 0.437 | 9.28E-42 | NK_cell |
| RASSF5 | 4.41E-46 | 3.73E-01 | 0.34 | 0.236 | 1.48E-41 | NK_cell |
| KDELR2 | 4.42E-46 | 3.20E-01 | 0.311 | 0.208 | 1.48E-41 | NK_cell |
| RHOG | 1.18E-45 | 2.65E-01 | 0.473 | 0.354 | 3.94E-41 | NK_cell |
| ARPC4 | 1.19E-45 | 3.31E-01 | 0.478 | 0.372 | 3.98E-41 | NK_cell |
| TNIP1 | 1.38E-45 | 3.80E-01 | 0.304 | 0.204 | 4.64E-41 | NK_cell |
| ARF4 | 2.69E-44 | 3.22E-01 | 0.443 | 0.342 | 9.02E-40 | NK_cell |
| ELOVL5 | 4.39E-44 | 3.67E-01 | 0.366 | 0.263 | 1.47E-39 | NK_cell |
| MYL12A | 4.67E-44 | 3.34E-01 | 0.787 | 0.707 | 1.56E-39 | NK_cell |
| YWHAZ | 6.05E-44 | 2.68E-01 | 0.779 | 0.674 | 2.03E-39 | NK_cell |
| CAP1 | 6.84E-44 | 2.90E-01 | 0.493 | 0.386 | 2.30E-39 | NK_cell |
| CDC37 | 8.42E-44 | 3.06E-01 | 0.443 | 0.343 | 2.83E-39 | NK_cell |
| SPPL2A | 9.79E-44 | 3.13E-01 | 0.28 | 0.18 | 3.28E-39 | NK_cell |
| ICAM2 | 1.13E-43 | 4.23E-01 | 0.278 | 0.182 | 3.78E-39 | NK_cell |
| PRKAR1A | 4.10E-43 | 3.63E-01 | 0.357 | 0.262 | 1.37E-38 | NK_cell |
| BAZ1A | 8.55E-43 | 3.34E-01 | 0.496 | 0.392 | 2.87E-38 | NK_cell |
| ILF2 | 1.77E-42 | 3.07E-01 | 0.396 | 0.294 | 5.94E-38 | NK_cell |
| ARPC5L | 1.16E-41 | 3.86E-01 | 0.352 | 0.257 | 3.89E-37 | NK_cell |
| PRMT2 | 1.71E-41 | 3.44E-01 | 0.308 | 0.213 | 5.74E-37 | NK_cell |
| FAM118A | 1.81E-41 | 3.54E-01 | 0.304 | 0.208 | 6.07E-37 | NK_cell |
| LSM3 | 3.78E-41 | 2.59E-01 | 0.347 | 0.25 | 1.27E-36 | NK_cell |
| JTB | 5.26E-41 | 2.66E-01 | 0.544 | 0.449 | 1.76E-36 | NK_cell |
| OPTN | 1.52E-40 | 3.08E-01 | 0.331 | 0.229 | 5.09E-36 | NK_cell |
| TMED10 | 1.80E-40 | 2.88E-01 | 0.377 | 0.279 | 6.04E-36 | NK_cell |
| TMED2 | 3.10E-40 | 2.78E-01 | 0.328 | 0.229 | 1.04E-35 | NK_cell |
| LMNA | 6.68E-40 | 4.10E-01 | 0.567 | 0.469 | 2.24E-35 | NK_cell |
| TMED9 | 7.83E-40 | 2.61E-01 | 0.382 | 0.283 | 2.63E-35 | NK_cell |
| SCP2 | 9.91E-40 | 2.71E-01 | 0.351 | 0.257 | 3.32E-35 | NK_cell |
| LINC00623 | 2.17E-39 | 3.11E-01 | 0.258 | 0.163 | 7.27E-35 | NK_cell |
| GRASP | 2.62E-39 | 6.83E-01 | 0.351 | 0.263 | 8.79E-35 | NK_cell |
| RSRP1 | 4.63E-39 | 3.24E-01 | 0.649 | 0.568 | 1.55E-34 | NK_cell |
| LMO4 | 1.34E-38 | 3.44E-01 | 0.257 | 0.167 | 4.49E-34 | NK_cell |
| FLNA | 2.40E-38 | 3.53E-01 | 0.314 | 0.221 | 8.04E-34 | NK_cell |
| RNPS1 | 2.68E-38 | 2.90E-01 | 0.426 | 0.331 | 8.99E-34 | NK_cell |
| RNF115 | 5.97E-38 | 3.06E-01 | 0.35 | 0.251 | 2.00E-33 | NK_cell |
| NDUFV1 | 9.43E-38 | 3.17E-01 | 0.271 | 0.182 | 3.16E-33 | NK_cell |
| MAF1 | 1.01E-37 | 2.54E-01 | 0.332 | 0.234 | 3.38E-33 | NK_cell |
| PIP4K2A | 2.78E-37 | 2.78E-01 | 0.415 | 0.316 | 9.33E-33 | NK_cell |
| UBE2I | 7.68E-37 | 2.73E-01 | 0.438 | 0.351 | 2.58E-32 | NK_cell |
| CDKN2D | 1.11E-36 | 3.99E-01 | 0.311 | 0.225 | 3.74E-32 | NK_cell |
| SURF4 | 1.29E-36 | 3.45E-01 | 0.321 | 0.231 | 4.34E-32 | NK_cell |
| PDCD4 | 1.68E-36 | 3.09E-01 | 0.46 | 0.373 | 5.64E-32 | NK_cell |
| PTP4A1 | 3.94E-36 | 2.90E-01 | 0.503 | 0.415 | 1.32E-31 | NK_cell |
| ZEB2 | 5.68E-36 | 3.01E-01 | 0.452 | 0.341 | 1.90E-31 | NK_cell |
| HIST1H4C | 7.91E-36 | 3.71E-01 | 0.36 | 0.261 | 2.65E-31 | NK_cell |
| RNASEH2C | 1.95E-35 | 2.79E-01 | 0.284 | 0.197 | 6.54E-31 | NK_cell |
| AP2M1 | 2.04E-35 | 2.57E-01 | 0.377 | 0.284 | 6.86E-31 | NK_cell |
| ARHGAP45 | 2.30E-35 | 2.94E-01 | 0.259 | 0.172 | 7.72E-31 | NK_cell |
| RORA | 2.95E-35 | 3.07E-01 | 0.499 | 0.388 | 9.89E-31 | NK_cell |
| HES4 | 5.88E-35 | 3.64E-01 | 0.25 | 0.157 | 1.97E-30 | NK_cell |
| MAZ | 6.50E-35 | 2.90E-01 | 0.307 | 0.218 | 2.18E-30 | NK_cell |
| RASSF1 | 7.18E-35 | 2.97E-01 | 0.286 | 0.198 | 2.41E-30 | NK_cell |
| SPN | 8.46E-35 | 3.10E-01 | 0.269 | 0.185 | 2.84E-30 | NK_cell |
| FAM49B | 8.48E-35 | 2.77E-01 | 0.413 | 0.324 | 2.84E-30 | NK_cell |
| N4BP2L1 | 8.75E-35 | 3.10E-01 | 0.262 | 0.175 | 2.93E-30 | NK_cell |
| SIGIRR | 1.63E-34 | 3.06E-01 | 0.31 | 0.222 | 5.48E-30 | NK_cell |
| ACTN4 | 3.99E-34 | 2.91E-01 | 0.381 | 0.294 | 1.34E-29 | NK_cell |
| SELENOT | 4.65E-34 | 2.55E-01 | 0.418 | 0.33 | 1.56E-29 | NK_cell |
| STRAP | 5.69E-34 | 2.79E-01 | 0.359 | 0.271 | 1.91E-29 | NK_cell |
| SAR1A | 7.72E-34 | 2.78E-01 | 0.291 | 0.205 | 2.59E-29 | NK_cell |
| MBP | 1.91E-33 | 3.38E-01 | 0.307 | 0.226 | 6.40E-29 | NK_cell |
| RNF126 | 6.34E-33 | 3.10E-01 | 0.259 | 0.177 | 2.13E-28 | NK_cell |
| PPP1R18 | 8.71E-33 | 2.97E-01 | 0.31 | 0.226 | 2.92E-28 | NK_cell |
| IER3 | 3.27E-32 | 3.75E-01 | 0.335 | 0.244 | 1.10E-27 | NK_cell |
| TLN1 | 3.36E-32 | 2.73E-01 | 0.341 | 0.257 | 1.13E-27 | NK_cell |
| EBP | 4.33E-32 | 2.90E-01 | 0.291 | 0.204 | 1.45E-27 | NK_cell |
| FNIP1 | 4.54E-32 | 3.07E-01 | 0.293 | 0.212 | 1.52E-27 | NK_cell |
| CPNE1 | 8.47E-32 | 2.88E-01 | 0.256 | 0.176 | 2.84E-27 | NK_cell |
| PIM2 | 1.41E-31 | 2.89E-01 | 0.406 | 0.317 | 4.73E-27 | NK_cell |
| RALGAPA1 | 1.45E-31 | 2.98E-01 | 0.261 | 0.178 | 4.88E-27 | NK_cell |
| MED10 | 1.56E-31 | 2.64E-01 | 0.426 | 0.345 | 5.24E-27 | NK_cell |
| EIF4H | 2.23E-31 | 2.72E-01 | 0.38 | 0.296 | 7.47E-27 | NK_cell |
| SLC9A3R1 | 2.32E-31 | 2.86E-01 | 0.292 | 0.208 | 7.78E-27 | NK_cell |
| METTL9 | 3.95E-31 | 2.88E-01 | 0.37 | 0.284 | 1.32E-26 | NK_cell |
| KLF3 | 4.19E-31 | 2.82E-01 | 0.271 | 0.187 | 1.40E-26 | NK_cell |
| PSMC4 | 8.55E-31 | 2.69E-01 | 0.27 | 0.192 | 2.87E-26 | NK_cell |
| CASP4 | 1.03E-30 | 2.82E-01 | 0.392 | 0.314 | 3.45E-26 | NK_cell |
| CXCR4 | 2.37E-30 | 3.09E-01 | 0.795 | 0.773 | 7.95E-26 | NK_cell |
| HNRNPUL1 | 2.85E-30 | 2.71E-01 | 0.422 | 0.334 | 9.55E-26 | NK_cell |
| RNF166 | 2.87E-30 | 3.00E-01 | 0.294 | 0.216 | 9.63E-26 | NK_cell |
| GHITM | 4.87E-30 | 2.52E-01 | 0.431 | 0.353 | 1.63E-25 | NK_cell |
| GATA3 | 6.12E-30 | 3.41E-01 | 0.355 | 0.267 | 2.05E-25 | NK_cell |
| LMAN2 | 1.11E-29 | 2.56E-01 | 0.387 | 0.311 | 3.73E-25 | NK_cell |
| SDF4 | 1.39E-29 | 2.66E-01 | 0.308 | 0.226 | 4.65E-25 | NK_cell |
| SH3GLB1 | 7.15E-29 | 2.66E-01 | 0.333 | 0.253 | 2.40E-24 | NK_cell |
| NXT1 | 1.51E-28 | 2.67E-01 | 0.346 | 0.264 | 5.06E-24 | NK_cell |
| RNF168 | 2.26E-28 | 2.74E-01 | 0.271 | 0.192 | 7.57E-24 | NK_cell |
| DSTN | 2.41E-28 | 3.15E-01 | 0.314 | 0.239 | 8.09E-24 | NK_cell |
| APOL6 | 2.78E-28 | 2.68E-01 | 0.286 | 0.21 | 9.31E-24 | NK_cell |
| SEC14L1 | 4.00E-28 | 2.71E-01 | 0.278 | 0.203 | 1.34E-23 | NK_cell |
| H2AFJ | 7.34E-28 | 2.58E-01 | 0.409 | 0.326 | 2.46E-23 | NK_cell |
| PGK1 | 9.11E-28 | 2.52E-01 | 0.66 | 0.586 | 3.06E-23 | NK_cell |
| CHD4 | 1.82E-27 | 2.82E-01 | 0.311 | 0.234 | 6.11E-23 | NK_cell |
| SDF2L1 | 1.93E-27 | 2.60E-01 | 0.294 | 0.216 | 6.49E-23 | NK_cell |
| BIN1 | 9.38E-27 | 2.62E-01 | 0.3 | 0.222 | 3.15E-22 | NK_cell |
| GGNBP2 | 1.18E-26 | 2.66E-01 | 0.396 | 0.32 | 3.94E-22 | NK_cell |
| CNOT2 | 2.53E-26 | 2.75E-01 | 0.34 | 0.263 | 8.48E-22 | NK_cell |
| HIPK1 | 4.02E-25 | 2.57E-01 | 0.305 | 0.229 | 1.35E-20 | NK_cell |
| PITPNC1 | 1.33E-24 | 2.71E-01 | 0.258 | 0.188 | 4.47E-20 | NK_cell |
| DDX27 | 9.04E-24 | 2.72E-01 | 0.269 | 0.2 | 3.03E-19 | NK_cell |
| ILF3-DT | 1.44E-23 | 3.47E-01 | 0.297 | 0.227 | 4.84E-19 | NK_cell |
| YME1L1 | 1.52E-23 | 2.78E-01 | 0.393 | 0.321 | 5.09E-19 | NK_cell |
| TENT5A | 6.30E-21 | 2.67E-01 | 0.264 | 0.199 | 2.11E-16 | NK_cell |
| MAPRE2 | 2.09E-18 | 2.68E-01 | 0.286 | 0.23 | 7.02E-14 | NK_cell |
| PPP1R15A | 2.31E-12 | 3.02E-01 | 0.88 | 0.821 | 7.76E-08 | NK_cell |
| CD3D | 0 | 2.31356939 | 0.829 | 0.101 | 0 | T_cells |
| TRAC | 0 | 2.29752094 | 0.758 | 0.132 | 0 | T_cells |
| CTLA4 | 0 | 2.23991126 | 0.488 | 0.02 | 0 | T_cells |
| MT1E | 0 | 2.18997203 | 0.446 | 0.096 | 0 | T_cells |
| DUSP4 | 0 | 2.14314764 | 0.663 | 0.158 | 0 | T_cells |
| MT1X | 0 | 2.01819142 | 0.606 | 0.272 | 0 | T_cells |
| CD27 | 0 | 1.99925329 | 0.547 | 0.05 | 0 | T_cells |
| CD3G | 0 | 1.72681007 | 0.582 | 0.046 | 0 | T_cells |
| ICA1 | 0 | 1.67272479 | 0.35 | 0.022 | 0 | T_cells |
| ARID5B | 0 | 1.62514026 | 0.615 | 0.192 | 0 | T_cells |
| CD2 | 0 | 1.53108467 | 0.702 | 0.245 | 0 | T_cells |
| TRBC2 | 0 | 1.52455646 | 0.769 | 0.484 | 0 | T_cells |
| HSPA1B | 0 | 1.50198373 | 0.717 | 0.338 | 0 | T_cells |
| IER5L | 0 | 1.49649872 | 0.582 | 0.179 | 0 | T_cells |
| BATF | 0 | 1.46391411 | 0.477 | 0.138 | 0 | T_cells |
| SOD1 | 0 | 1.44990664 | 0.854 | 0.6 | 0 | T_cells |
| MAGEH1 | 0 | 1.39102454 | 0.272 | 0.058 | 0 | T_cells |
| CLEC2D | 0 | 1.38584527 | 0.543 | 0.149 | 0 | T_cells |
| CD8A | 0 | 1.29171435 | 0.336 | 0.088 | 0 | T_cells |
| DNAJB1 | 0 | 1.27968541 | 0.921 | 0.639 | 0 | T_cells |
| ID3 | 0 | 1.27868056 | 0.351 | 0.096 | 0 | T_cells |
| CD8B | 0 | 1.27051841 | 0.271 | 0.018 | 0 | T_cells |
| HSPB1 | 0 | 1.26864941 | 0.781 | 0.423 | 0 | T_cells |
| HSPA1A | 0 | 1.24871836 | 0.88 | 0.528 | 0 | T_cells |
| BAG3 | 0 | 1.22983447 | 0.408 | 0.123 | 0 | T_cells |
| IL7R | 0 | 1.22123054 | 0.619 | 0.252 | 0 | T_cells |
| CACYBP | 0 | 1.18186496 | 0.78 | 0.453 | 0 | T_cells |
| ICOS | 0 | 1.15912126 | 0.428 | 0.107 | 0 | T_cells |
| CD3E | 0 | 1.15746658 | 0.855 | 0.434 | 0 | T_cells |
| XIST | 0 | 1.15528138 | 0.617 | 0.232 | 0 | T_cells |
| TBC1D4 | 0 | 1.11643058 | 0.297 | 0.015 | 0 | T_cells |
| HSP90AA1 | 0 | 1.04481693 | 0.989 | 0.959 | 0 | T_cells |
| PLK3 | 0 | 1.01450874 | 0.481 | 0.178 | 0 | T_cells |
| SRSF7 | 0 | 0.95959136 | 0.938 | 0.827 | 0 | T_cells |
| HSP90AB1 | 0 | 0.95264517 | 0.973 | 0.928 | 0 | T_cells |
| GNAS | 0 | 0.93600563 | 0.867 | 0.624 | 0 | T_cells |
| UBC | 0 | 0.93231604 | 0.991 | 0.98 | 0 | T_cells |
| HSPH1 | 0 | 0.89547482 | 0.798 | 0.456 | 0 | T_cells |
| RGS10 | 0 | 0.86933318 | 0.442 | 0.151 | 0 | T_cells |
| CD28 | 0 | 0.85966943 | 0.256 | 0.021 | 0 | T_cells |
| IL6ST | 0 | 0.83961406 | 0.301 | 0.075 | 0 | T_cells |
| RPL28 | 2.82E-301 | 4.45E-01 | 0.998 | 0.993 | 9.47E-297 | T_cells |
| ITM2A | 5.32E-297 | 1.28E+00 | 0.647 | 0.361 | 1.79E-292 | T_cells |
| PRDX1 | 1.45E-296 | 1.05E+00 | 0.607 | 0.307 | 4.85E-292 | T_cells |
| HSPE1 | 5.16E-293 | 1.12E+00 | 0.919 | 0.797 | 1.73E-288 | T_cells |
| BCL2 | 2.88E-292 | 8.88E-01 | 0.405 | 0.139 | 9.67E-288 | T_cells |
| GADD45G | 1.30E-284 | 1.15E+00 | 0.65 | 0.345 | 4.35E-280 | T_cells |
| CORO1B | 1.72E-270 | 8.81E-01 | 0.32 | 0.096 | 5.77E-266 | T_cells |
| HSPA8 | 1.44E-265 | 7.83E-01 | 0.964 | 0.906 | 4.84E-261 | T_cells |
| BTG1 | 1.54E-261 | 5.84E-01 | 0.993 | 0.97 | 5.17E-257 | T_cells |
| SAP30 | 2.99E-260 | 9.16E-01 | 0.298 | 0.086 | 1.00E-255 | T_cells |
| SNX9 | 7.21E-259 | 7.96E-01 | 0.296 | 0.087 | 2.42E-254 | T_cells |
| IFI6 | 1.23E-250 | 1.01E+00 | 0.611 | 0.326 | 4.12E-246 | T_cells |
| PTGES3 | 1.41E-249 | 1.01E+00 | 0.765 | 0.526 | 4.73E-245 | T_cells |
| JUNB | 2.45E-249 | 8.59E-01 | 0.967 | 0.916 | 8.21E-245 | T_cells |
| SERPINH1 | 7.18E-247 | 1.12E+00 | 0.304 | 0.089 | 2.41E-242 | T_cells |
| MAF | 3.26E-245 | 8.96E-01 | 0.368 | 0.132 | 1.09E-240 | T_cells |
| IL32 | 1.78E-240 | 7.42E-01 | 0.873 | 0.5 | 5.95E-236 | T_cells |
| FOXO1 | 2.29E-240 | 7.50E-01 | 0.299 | 0.092 | 7.68E-236 | T_cells |
| AC058791.1 | 4.98E-230 | 9.32E-01 | 0.493 | 0.226 | 1.67E-225 | T_cells |
| JUND | 2.80E-228 | 6.94E-01 | 0.971 | 0.874 | 9.39E-224 | T_cells |
| TSPYL2 | 2.13E-225 | 9.73E-01 | 0.725 | 0.477 | 7.14E-221 | T_cells |
| C12orf57 | 1.90E-223 | 8.42E-01 | 0.714 | 0.47 | 6.37E-219 | T_cells |
| TNFRSF9 | 8.41E-223 | 9.31E-01 | 0.417 | 0.17 | 2.82E-218 | T_cells |
| BTG3 | 3.67E-220 | 1.00E+00 | 0.498 | 0.245 | 1.23E-215 | T_cells |
| TXNIP | 7.95E-220 | 6.12E-01 | 0.82 | 0.554 | 2.66E-215 | T_cells |
| HSPD1 | 9.94E-220 | 8.29E-01 | 0.844 | 0.665 | 3.33E-215 | T_cells |
| FABP5 | 2.56E-216 | 1.11E+00 | 0.446 | 0.201 | 8.58E-212 | T_cells |
| PTTG1 | 3.54E-214 | 8.03E-01 | 0.305 | 0.102 | 1.19E-209 | T_cells |
| DNAJA1 | 5.36E-214 | 8.40E-01 | 0.898 | 0.766 | 1.80E-209 | T_cells |
| RNF19A | 5.89E-207 | 8.86E-01 | 0.609 | 0.354 | 1.98E-202 | T_cells |
| TOB1 | 6.00E-203 | 8.30E-01 | 0.472 | 0.224 | 2.01E-198 | T_cells |
| PHACTR2 | 6.36E-198 | 6.60E-01 | 0.252 | 0.077 | 2.13E-193 | T_cells |
| GADD45A | 7.05E-197 | 1.01E+00 | 0.556 | 0.308 | 2.37E-192 | T_cells |
| PELI1 | 1.19E-190 | 6.76E-01 | 0.26 | 0.083 | 3.99E-186 | T_cells |
| HLA-DRB1 | 7.29E-187 | 4.41E-01 | 0.684 | 0.428 | 2.44E-182 | T_cells |
| UBE2S | 5.76E-185 | 7.53E-01 | 0.76 | 0.538 | 1.93E-180 | T_cells |
| NCK2 | 2.43E-184 | 6.63E-01 | 0.297 | 0.107 | 8.14E-180 | T_cells |
| SGK1 | 2.22E-182 | 8.17E-01 | 0.329 | 0.129 | 7.45E-178 | T_cells |
| MT-CYB | 3.06E-181 | 4.73E-01 | 0.982 | 0.978 | 1.03E-176 | T_cells |
| MT-ATP6 | 2.38E-178 | 4.52E-01 | 0.986 | 0.98 | 7.97E-174 | T_cells |
| WNK1 | 4.89E-178 | 7.53E-01 | 0.365 | 0.157 | 1.64E-173 | T_cells |
| IPCEF1 | 1.40E-174 | 6.20E-01 | 0.273 | 0.094 | 4.68E-170 | T_cells |
| ZBTB38 | 2.37E-160 | 6.07E-01 | 0.275 | 0.1 | 7.95E-156 | T_cells |
| SPATS2L | 7.96E-160 | 6.14E-01 | 0.27 | 0.099 | 2.67E-155 | T_cells |
| TNFSF9 | 5.35E-159 | 1.27E+00 | 0.325 | 0.139 | 1.79E-154 | T_cells |
| MT-ND3 | 5.18E-158 | 4.42E-01 | 0.975 | 0.962 | 1.74E-153 | T_cells |
| KLF6 | 1.76E-157 | 6.41E-01 | 0.942 | 0.881 | 5.91E-153 | T_cells |
| HSPA6 | 9.46E-157 | 6.69E-01 | 0.508 | 0.257 | 3.17E-152 | T_cells |
| LIMS1 | 1.29E-156 | 7.23E-01 | 0.381 | 0.178 | 4.32E-152 | T_cells |
| NR3C1 | 2.05E-150 | 8.83E-01 | 0.544 | 0.331 | 6.87E-146 | T_cells |
| HLA-DQB1 | 3.03E-149 | 3.21E-01 | 0.31 | 0.123 | 1.02E-144 | T_cells |
| RGCC | 2.74E-145 | 8.11E-01 | 0.798 | 0.652 | 9.18E-141 | T_cells |
| MSI2 | 1.24E-143 | 5.92E-01 | 0.278 | 0.11 | 4.17E-139 | T_cells |
| RPL30 | 7.89E-143 | 3.28E-01 | 0.992 | 0.991 | 2.65E-138 | T_cells |
| GSPT1 | 2.75E-141 | 7.63E-01 | 0.416 | 0.219 | 9.22E-137 | T_cells |
| STAT3 | 7.22E-141 | 7.34E-01 | 0.589 | 0.381 | 2.42E-136 | T_cells |
| RPL12 | 2.51E-137 | 3.51E-01 | 0.988 | 0.985 | 8.41E-133 | T_cells |
| JUN | 1.61E-136 | 4.84E-01 | 0.954 | 0.84 | 5.40E-132 | T_cells |
| SMAP2 | 2.05E-136 | 6.94E-01 | 0.678 | 0.491 | 6.86E-132 | T_cells |
| RPS12 | 4.03E-134 | 3.26E-01 | 0.994 | 0.994 | 1.35E-129 | T_cells |
| TNFAIP8 | 3.40E-133 | 6.91E-01 | 0.35 | 0.165 | 1.14E-128 | T_cells |
| NDUFV2 | 9.15E-133 | 7.22E-01 | 0.465 | 0.269 | 3.07E-128 | T_cells |
| MT2A | 2.23E-132 | 1.19E+00 | 0.866 | 0.745 | 7.48E-128 | T_cells |
| AHSA1 | 2.99E-132 | 7.02E-01 | 0.575 | 0.371 | 1.00E-127 | T_cells |
| TUBB4B | 1.28E-131 | 5.85E-01 | 0.893 | 0.807 | 4.31E-127 | T_cells |
| LINC00513 | 8.19E-130 | 5.89E-01 | 0.31 | 0.137 | 2.75E-125 | T_cells |
| DEDD2 | 5.31E-128 | 6.45E-01 | 0.499 | 0.293 | 1.78E-123 | T_cells |
| ANKRD12 | 3.32E-125 | 6.93E-01 | 0.614 | 0.418 | 1.11E-120 | T_cells |
| PHLDA1 | 3.80E-125 | 7.57E-01 | 0.557 | 0.345 | 1.27E-120 | T_cells |
| COTL1 | 1.57E-124 | 3.82E-01 | 0.627 | 0.378 | 5.28E-120 | T_cells |
| FKBP4 | 2.68E-123 | 6.29E-01 | 0.426 | 0.225 | 8.99E-119 | T_cells |
| SPOCK2 | 5.47E-123 | 6.33E-01 | 0.561 | 0.348 | 1.83E-118 | T_cells |
| PPP1R2 | 3.20E-122 | 7.19E-01 | 0.671 | 0.502 | 1.07E-117 | T_cells |
| AC016831.5 | 5.95E-122 | 6.68E-01 | 0.461 | 0.265 | 2.00E-117 | T_cells |
| HSPA4 | 4.67E-121 | 6.38E-01 | 0.389 | 0.203 | 1.57E-116 | T_cells |
| IFI44L | 5.46E-117 | 4.42E-01 | 0.458 | 0.236 | 1.83E-112 | T_cells |
| PRDM1 | 2.54E-116 | 6.93E-01 | 0.342 | 0.168 | 8.51E-112 | T_cells |
| CYTIP | 9.74E-115 | 6.38E-01 | 0.682 | 0.506 | 3.27E-110 | T_cells |
| MDFIC | 1.13E-113 | 5.21E-01 | 0.29 | 0.132 | 3.80E-109 | T_cells |
| SPTAN1 | 6.32E-112 | 6.05E-01 | 0.459 | 0.266 | 2.12E-107 | T_cells |
| FUS | 9.80E-111 | 4.84E-01 | 0.894 | 0.809 | 3.29E-106 | T_cells |
| RGS2 | 1.62E-110 | 9.63E-01 | 0.642 | 0.483 | 5.43E-106 | T_cells |
| CHORDC1 | 2.90E-109 | 5.63E-01 | 0.507 | 0.311 | 9.72E-105 | T_cells |
| ATXN1 | 3.02E-108 | 5.43E-01 | 0.277 | 0.125 | 1.01E-103 | T_cells |
| SNHG12 | 3.78E-107 | 6.48E-01 | 0.477 | 0.294 | 1.27E-102 | T_cells |
| SOCS3 | 4.23E-106 | 5.77E-01 | 0.384 | 0.207 | 1.42E-101 | T_cells |
| DYNLL1 | 1.92E-104 | 5.34E-01 | 0.722 | 0.568 | 6.45E-100 | T_cells |
| MALAT1 | 8.27E-104 | 2.70E-01 | 1 | 0.999 | 2.77E-99 | T_cells |
| FAS | 1.50E-103 | 5.34E-01 | 0.256 | 0.113 | 5.02E-99 | T_cells |
| NSD3 | 3.65E-102 | 6.10E-01 | 0.462 | 0.281 | 1.22E-97 | T_cells |
| PMAIP1 | 6.76E-101 | 8.03E-01 | 0.71 | 0.601 | 2.27E-96 | T_cells |
| JMY | 2.59E-100 | 6.64E-01 | 0.338 | 0.177 | 8.69E-96 | T_cells |
| DNAJA4 | 7.97E-99 | 5.06E-01 | 0.308 | 0.149 | 2.67E-94 | T_cells |
| RPL11 | 1.53E-98 | 2.55E-01 | 0.993 | 0.991 | 5.12E-94 | T_cells |
| SLBP | 6.92E-97 | 6.05E-01 | 0.461 | 0.287 | 2.32E-92 | T_cells |
| STK17B | 5.28E-93 | 5.74E-01 | 0.667 | 0.52 | 1.77E-88 | T_cells |
| ZFP36L2 | 4.20E-92 | 4.58E-01 | 0.938 | 0.893 | 1.41E-87 | T_cells |
| KIAA1551 | 5.29E-92 | 6.06E-01 | 0.381 | 0.218 | 1.77E-87 | T_cells |
| H2AFZ | 1.28E-90 | 6.51E-01 | 0.774 | 0.671 | 4.28E-86 | T_cells |
| MRPL18 | 3.37E-89 | 6.24E-01 | 0.445 | 0.276 | 1.13E-84 | T_cells |
| VMP1 | 7.17E-89 | 6.26E-01 | 0.405 | 0.245 | 2.40E-84 | T_cells |
| H1FX | 9.31E-89 | 4.09E-01 | 0.618 | 0.425 | 3.12E-84 | T_cells |
| IFI44 | 3.97E-88 | 4.38E-01 | 0.262 | 0.125 | 1.33E-83 | T_cells |
| NPM1 | 2.09E-87 | 4.01E-01 | 0.896 | 0.837 | 7.00E-83 | T_cells |
| FKBP5 | 4.39E-87 | 5.92E-01 | 0.483 | 0.314 | 1.47E-82 | T_cells |
| CDKN1B | 6.73E-87 | 4.85E-01 | 0.278 | 0.138 | 2.26E-82 | T_cells |
| TIGIT | 2.75E-86 | 6.15E-01 | 0.372 | 0.213 | 9.24E-82 | T_cells |
| GLS | 7.42E-85 | 4.98E-01 | 0.439 | 0.272 | 2.49E-80 | T_cells |
| TMEM173 | 1.28E-84 | 4.82E-01 | 0.312 | 0.166 | 4.28E-80 | T_cells |
| NOP58 | 1.58E-84 | 5.89E-01 | 0.455 | 0.297 | 5.30E-80 | T_cells |
| DOK2 | 2.31E-84 | 6.16E-01 | 0.654 | 0.518 | 7.74E-80 | T_cells |
| RHBDD2 | 2.43E-84 | 6.11E-01 | 0.332 | 0.186 | 8.15E-80 | T_cells |
| ELF1 | 1.18E-83 | 5.48E-01 | 0.732 | 0.603 | 3.96E-79 | T_cells |
| ZFAND2A | 5.20E-83 | 6.42E-01 | 0.481 | 0.318 | 1.74E-78 | T_cells |
| CDV3 | 3.50E-82 | 5.56E-01 | 0.524 | 0.362 | 1.17E-77 | T_cells |
| AC016831.7 | 6.77E-82 | 5.14E-01 | 0.304 | 0.161 | 2.27E-77 | T_cells |
| TUBA1C | 7.41E-82 | 5.58E-01 | 0.417 | 0.259 | 2.48E-77 | T_cells |
| JMJD1C | 5.02E-81 | 5.76E-01 | 0.483 | 0.326 | 1.68E-76 | T_cells |
| RBPJ | 3.20E-79 | 5.93E-01 | 0.316 | 0.177 | 1.07E-74 | T_cells |
| FAM107B | 1.91E-78 | 4.50E-01 | 0.568 | 0.394 | 6.41E-74 | T_cells |
| EPSTI1 | 5.67E-78 | 4.65E-01 | 0.365 | 0.211 | 1.90E-73 | T_cells |
| ZFP36L1 | 3.02E-77 | 4.98E-01 | 0.65 | 0.491 | 1.01E-72 | T_cells |
| STIP1 | 9.20E-76 | 5.07E-01 | 0.411 | 0.258 | 3.09E-71 | T_cells |
| ZFAND5 | 5.98E-75 | 5.52E-01 | 0.656 | 0.517 | 2.00E-70 | T_cells |
| LTB | 4.11E-74 | 7.51E-01 | 0.436 | 0.285 | 1.38E-69 | T_cells |
| PTPRC | 9.21E-74 | 4.24E-01 | 0.85 | 0.755 | 3.09E-69 | T_cells |
| TNFRSF4 | 1.78E-70 | 8.87E-01 | 0.319 | 0.191 | 5.98E-66 | T_cells |
| CITED2 | 3.67E-70 | 5.77E-01 | 0.631 | 0.497 | 1.23E-65 | T_cells |
| H3F3B | 1.16E-69 | 2.73E-01 | 0.992 | 0.984 | 3.89E-65 | T_cells |
| ODC1 | 1.40E-69 | 6.10E-01 | 0.445 | 0.306 | 4.71E-65 | T_cells |
| EIF2AK2 | 3.44E-69 | 4.12E-01 | 0.35 | 0.204 | 1.15E-64 | T_cells |
| STK17A | 1.95E-66 | 5.33E-01 | 0.665 | 0.551 | 6.53E-62 | T_cells |
| H2AFX | 4.53E-66 | 4.74E-01 | 0.611 | 0.47 | 1.52E-61 | T_cells |
| RSRC2 | 1.02E-65 | 4.75E-01 | 0.6 | 0.465 | 3.43E-61 | T_cells |
| DUSP5 | 4.36E-64 | 4.48E-01 | 0.417 | 0.268 | 1.46E-59 | T_cells |
| CD52 | 1.08E-63 | 3.17E-01 | 0.706 | 0.502 | 3.62E-59 | T_cells |
| KCNQ1OT1 | 1.34E-63 | 6.90E-01 | 0.299 | 0.172 | 4.49E-59 | T_cells |
| SOCS1 | 1.43E-63 | 4.20E-01 | 0.63 | 0.486 | 4.81E-59 | T_cells |
| EIF4A2 | 2.31E-63 | 3.56E-01 | 0.804 | 0.706 | 7.73E-59 | T_cells |
| HLA-DMA | 6.00E-63 | 2.94E-01 | 0.252 | 0.133 | 2.01E-58 | T_cells |
| RNASET2 | 2.40E-62 | 3.70E-01 | 0.412 | 0.266 | 8.05E-58 | T_cells |
| HERPUD1 | 5.01E-62 | 3.87E-01 | 0.747 | 0.634 | 1.68E-57 | T_cells |
| B3GNT2 | 3.52E-61 | 4.19E-01 | 0.264 | 0.146 | 1.18E-56 | T_cells |
| AMD1 | 7.10E-61 | 4.39E-01 | 0.578 | 0.436 | 2.38E-56 | T_cells |
| XAF1 | 8.82E-61 | 4.56E-01 | 0.425 | 0.279 | 2.96E-56 | T_cells |
| ZC3HAV1 | 1.54E-60 | 4.70E-01 | 0.547 | 0.406 | 5.15E-56 | T_cells |
| TAF1D | 1.74E-60 | 4.61E-01 | 0.552 | 0.412 | 5.82E-56 | T_cells |
| PTPN1 | 2.11E-60 | 4.47E-01 | 0.341 | 0.211 | 7.09E-56 | T_cells |
| SYNE2 | 3.40E-60 | 4.29E-01 | 0.446 | 0.293 | 1.14E-55 | T_cells |
| FYB1 | 4.48E-60 | 5.05E-01 | 0.561 | 0.43 | 1.50E-55 | T_cells |
| TYMP | 4.70E-60 | 5.19E-01 | 0.362 | 0.23 | 1.58E-55 | T_cells |
| ANP32B | 2.92E-59 | 4.11E-01 | 0.545 | 0.404 | 9.81E-55 | T_cells |
| ITK | 3.61E-59 | 4.31E-01 | 0.266 | 0.149 | 1.21E-54 | T_cells |
| DDX24 | 1.21E-58 | 3.94E-01 | 0.78 | 0.69 | 4.07E-54 | T_cells |
| RHOB | 1.94E-58 | 6.26E-01 | 0.458 | 0.326 | 6.52E-54 | T_cells |
| CLPP | 8.50E-58 | 4.19E-01 | 0.306 | 0.185 | 2.85E-53 | T_cells |
| NSMCE3 | 2.62E-57 | 4.57E-01 | 0.389 | 0.254 | 8.79E-53 | T_cells |
| LBH | 3.79E-57 | 4.62E-01 | 0.467 | 0.325 | 1.27E-52 | T_cells |
| SRSF3 | 5.74E-57 | 3.80E-01 | 0.804 | 0.719 | 1.93E-52 | T_cells |
| HNRNPH3 | 1.66E-56 | 4.25E-01 | 0.506 | 0.372 | 5.58E-52 | T_cells |
| NUFIP2 | 1.67E-55 | 4.12E-01 | 0.422 | 0.285 | 5.60E-51 | T_cells |
| SARAF | 2.35E-55 | 3.75E-01 | 0.913 | 0.868 | 7.88E-51 | T_cells |
| DYNLL2 | 2.81E-55 | 4.38E-01 | 0.297 | 0.177 | 9.42E-51 | T_cells |
| PER1 | 4.53E-55 | 3.96E-01 | 0.438 | 0.299 | 1.52E-50 | T_cells |
| LCK | 6.79E-55 | 4.01E-01 | 0.4 | 0.262 | 2.28E-50 | T_cells |
| UBE2B | 1.84E-54 | 4.76E-01 | 0.567 | 0.452 | 6.17E-50 | T_cells |
| PRDM2 | 6.19E-54 | 4.13E-01 | 0.339 | 0.213 | 2.08E-49 | T_cells |
| BCAS2 | 9.22E-54 | 5.55E-01 | 0.548 | 0.431 | 3.09E-49 | T_cells |
| ETS1 | 1.48E-53 | 4.34E-01 | 0.557 | 0.426 | 4.96E-49 | T_cells |
| ZNF331 | 3.65E-53 | 5.16E-01 | 0.667 | 0.566 | 1.22E-48 | T_cells |
| UBALD2 | 3.99E-53 | 4.31E-01 | 0.57 | 0.44 | 1.34E-48 | T_cells |
| PEBP1 | 5.54E-53 | 4.06E-01 | 0.471 | 0.342 | 1.86E-48 | T_cells |
| CCT4 | 1.32E-52 | 4.59E-01 | 0.549 | 0.436 | 4.41E-48 | T_cells |
| SBDS | 2.97E-52 | 4.09E-01 | 0.521 | 0.389 | 9.95E-48 | T_cells |
| SF1 | 8.77E-52 | 3.99E-01 | 0.648 | 0.535 | 2.94E-47 | T_cells |
| STAT1 | 1.30E-51 | 4.06E-01 | 0.304 | 0.188 | 4.35E-47 | T_cells |
| NEU1 | 1.60E-51 | 5.12E-01 | 0.507 | 0.385 | 5.36E-47 | T_cells |
| TGIF1 | 1.11E-49 | 4.26E-01 | 0.268 | 0.16 | 3.71E-45 | T_cells |
| TNF | 1.34E-49 | 4.17E-01 | 0.283 | 0.168 | 4.50E-45 | T_cells |
| TUBB2A | 1.51E-47 | 3.72E-01 | 0.268 | 0.16 | 5.05E-43 | T_cells |
| CKS2 | 5.98E-47 | 4.80E-01 | 0.328 | 0.214 | 2.01E-42 | T_cells |
| OASL | 1.80E-46 | 4.07E-01 | 0.253 | 0.149 | 6.03E-42 | T_cells |
| CYTOR | 2.67E-46 | 5.07E-01 | 0.457 | 0.342 | 8.97E-42 | T_cells |
| CDC42EP3 | 1.45E-45 | 4.10E-01 | 0.319 | 0.206 | 4.87E-41 | T_cells |
| WSB1 | 2.71E-45 | 4.04E-01 | 0.542 | 0.425 | 9.08E-41 | T_cells |
| SLC3A2 | 1.78E-44 | 4.16E-01 | 0.583 | 0.473 | 5.98E-40 | T_cells |
| N4BP1 | 2.86E-44 | 3.58E-01 | 0.255 | 0.154 | 9.59E-40 | T_cells |
| BIRC3 | 6.00E-44 | 4.11E-01 | 0.454 | 0.332 | 2.01E-39 | T_cells |
| SMCHD1 | 1.32E-43 | 4.58E-01 | 0.498 | 0.386 | 4.41E-39 | T_cells |
| SLC38A2 | 1.75E-43 | 4.04E-01 | 0.615 | 0.51 | 5.86E-39 | T_cells |
| RBM8A | 3.95E-43 | 3.48E-01 | 0.728 | 0.645 | 1.32E-38 | T_cells |
| CNIH1 | 2.15E-42 | 3.96E-01 | 0.275 | 0.175 | 7.22E-38 | T_cells |
| CARD16 | 5.30E-42 | 6.10E-01 | 0.514 | 0.415 | 1.78E-37 | T_cells |
| NDFIP1 | 5.63E-42 | 3.64E-01 | 0.423 | 0.306 | 1.89E-37 | T_cells |
| RGS16 | 2.03E-41 | 4.42E-01 | 0.257 | 0.157 | 6.80E-37 | T_cells |
| SAT1 | 2.78E-41 | 4.21E-01 | 0.86 | 0.834 | 9.32E-37 | T_cells |
| RAN | 3.24E-41 | 3.37E-01 | 0.66 | 0.569 | 1.09E-36 | T_cells |
| RGS1 | 4.58E-41 | 4.27E-01 | 0.805 | 0.725 | 1.54E-36 | T_cells |
| CBX4 | 5.36E-41 | 2.99E-01 | 0.271 | 0.168 | 1.80E-36 | T_cells |
| SERPINB9 | 2.98E-40 | 3.52E-01 | 0.323 | 0.213 | 1.00E-35 | T_cells |
| TOMM20 | 6.73E-40 | 3.47E-01 | 0.524 | 0.416 | 2.26E-35 | T_cells |
| MAP3K2 | 1.17E-39 | 3.77E-01 | 0.297 | 0.197 | 3.93E-35 | T_cells |
| AC243960.1 | 2.14E-39 | 3.50E-01 | 0.253 | 0.155 | 7.18E-35 | T_cells |
| PIK3IP1 | 6.15E-39 | 3.22E-01 | 0.684 | 0.581 | 2.06E-34 | T_cells |
| HMGB2 | 7.91E-39 | 3.96E-01 | 0.653 | 0.554 | 2.65E-34 | T_cells |
| ODF2L | 1.05E-38 | 4.23E-01 | 0.38 | 0.273 | 3.53E-34 | T_cells |
| NUDC | 5.01E-38 | 3.96E-01 | 0.483 | 0.376 | 1.68E-33 | T_cells |
| TSPYL1 | 6.04E-38 | 3.27E-01 | 0.266 | 0.17 | 2.03E-33 | T_cells |
| LDHB | 8.29E-37 | 3.38E-01 | 0.547 | 0.435 | 2.78E-32 | T_cells |
| UGP2 | 9.09E-37 | 5.08E-01 | 0.379 | 0.284 | 3.05E-32 | T_cells |
| PTP4A2 | 1.53E-36 | 3.42E-01 | 0.542 | 0.432 | 5.14E-32 | T_cells |
| GBP2 | 6.19E-36 | 3.31E-01 | 0.38 | 0.27 | 2.08E-31 | T_cells |
| IKZF3 | 8.34E-36 | 3.71E-01 | 0.265 | 0.17 | 2.80E-31 | T_cells |
| KMT2A | 1.67E-35 | 3.44E-01 | 0.255 | 0.162 | 5.61E-31 | T_cells |
| SIAH2 | 2.38E-35 | 3.46E-01 | 0.365 | 0.261 | 8.00E-31 | T_cells |
| CCT3 | 2.44E-35 | 3.43E-01 | 0.423 | 0.318 | 8.17E-31 | T_cells |
| PDCL3 | 3.27E-35 | 4.59E-01 | 0.306 | 0.211 | 1.10E-30 | T_cells |
| RSBN1 | 5.89E-35 | 3.47E-01 | 0.274 | 0.181 | 1.97E-30 | T_cells |
| EVI2B | 6.60E-35 | 3.60E-01 | 0.357 | 0.256 | 2.21E-30 | T_cells |
| ISCU | 2.51E-34 | 3.42E-01 | 0.393 | 0.289 | 8.41E-30 | T_cells |
| JPT1 | 2.56E-34 | 3.89E-01 | 0.462 | 0.362 | 8.58E-30 | T_cells |
| LAT | 7.22E-34 | 3.24E-01 | 0.311 | 0.212 | 2.42E-29 | T_cells |
| RILPL2 | 1.40E-33 | 3.35E-01 | 0.384 | 0.281 | 4.69E-29 | T_cells |
| EIF1AX | 2.45E-33 | 3.43E-01 | 0.517 | 0.42 | 8.23E-29 | T_cells |
| CELF2 | 7.68E-33 | 3.56E-01 | 0.511 | 0.412 | 2.58E-28 | T_cells |
| SP100 | 7.90E-33 | 3.39E-01 | 0.478 | 0.374 | 2.65E-28 | T_cells |
| CHST11 | 2.93E-32 | 3.03E-01 | 0.356 | 0.255 | 9.82E-28 | T_cells |
| SMARCA2 | 1.35E-30 | 3.23E-01 | 0.306 | 0.214 | 4.51E-26 | T_cells |
| CNOT6L | 2.69E-30 | 3.69E-01 | 0.569 | 0.476 | 9.04E-26 | T_cells |
| NEDD9 | 6.33E-30 | 3.29E-01 | 0.266 | 0.18 | 2.12E-25 | T_cells |
| SNHG8 | 2.31E-29 | 3.17E-01 | 0.611 | 0.527 | 7.74E-25 | T_cells |
| TUBA4A | 2.32E-29 | 3.68E-01 | 0.764 | 0.69 | 7.77E-25 | T_cells |
| TANK | 3.78E-29 | 3.45E-01 | 0.393 | 0.3 | 1.27E-24 | T_cells |
| GAPDH | 7.08E-29 | 2.60E-01 | 0.94 | 0.929 | 2.38E-24 | T_cells |
| NASP | 1.10E-28 | 3.27E-01 | 0.536 | 0.446 | 3.68E-24 | T_cells |
| EIF5 | 1.16E-28 | 2.67E-01 | 0.741 | 0.674 | 3.90E-24 | T_cells |
| TOPORS | 3.73E-28 | 3.16E-01 | 0.277 | 0.192 | 1.25E-23 | T_cells |
| RAP1A | 4.59E-28 | 2.90E-01 | 0.482 | 0.388 | 1.54E-23 | T_cells |
| CBLB | 8.84E-28 | 2.93E-01 | 0.344 | 0.248 | 2.97E-23 | T_cells |
| CDKN1A | 1.09E-27 | 2.82E-01 | 0.278 | 0.192 | 3.64E-23 | T_cells |
| BCL2L11 | 1.25E-27 | 3.86E-01 | 0.306 | 0.223 | 4.20E-23 | T_cells |
| HNRNPU | 1.76E-27 | 3.04E-01 | 0.696 | 0.624 | 5.90E-23 | T_cells |
| CLK1 | 3.45E-27 | 3.36E-01 | 0.638 | 0.586 | 1.16E-22 | T_cells |
| EZR | 5.07E-27 | 2.96E-01 | 0.747 | 0.666 | 1.70E-22 | T_cells |
| SAMSN1 | 1.10E-26 | 4.30E-01 | 0.5 | 0.428 | 3.70E-22 | T_cells |
| RHOH | 1.19E-26 | 2.90E-01 | 0.505 | 0.404 | 3.98E-22 | T_cells |
| FAM133B | 5.55E-26 | 3.07E-01 | 0.549 | 0.46 | 1.86E-21 | T_cells |
| MZT2A | 1.38E-25 | 3.08E-01 | 0.439 | 0.347 | 4.61E-21 | T_cells |
| TOP1 | 1.43E-25 | 2.94E-01 | 0.38 | 0.291 | 4.81E-21 | T_cells |
| TENT5C | 1.89E-25 | 3.30E-01 | 0.41 | 0.324 | 6.35E-21 | T_cells |
| S100A4 | 2.20E-25 | 3.45E-01 | 0.805 | 0.734 | 7.39E-21 | T_cells |
| GTF2B | 3.75E-25 | 2.91E-01 | 0.272 | 0.193 | 1.26E-20 | T_cells |
| CRYBG1 | 4.14E-25 | 3.35E-01 | 0.37 | 0.284 | 1.39E-20 | T_cells |
| NKTR | 4.24E-25 | 3.39E-01 | 0.436 | 0.345 | 1.42E-20 | T_cells |
| HBP1 | 5.86E-25 | 2.85E-01 | 0.291 | 0.211 | 1.97E-20 | T_cells |
| MYLIP | 1.12E-24 | 3.07E-01 | 0.382 | 0.295 | 3.75E-20 | T_cells |
| ATF3 | 1.39E-24 | 3.69E-01 | 0.506 | 0.425 | 4.65E-20 | T_cells |
| TRBC1 | 5.05E-24 | 7.05E-01 | 0.585 | 0.563 | 1.69E-19 | T_cells |
| ARIH1 | 2.79E-23 | 3.03E-01 | 0.292 | 0.215 | 9.34E-19 | T_cells |
| PPP2R5C | 3.22E-23 | 2.56E-01 | 0.626 | 0.544 | 1.08E-18 | T_cells |
| IFI16 | 5.09E-23 | 3.22E-01 | 0.475 | 0.391 | 1.71E-18 | T_cells |
| ABHD5 | 5.81E-23 | 3.04E-01 | 0.287 | 0.21 | 1.95E-18 | T_cells |
| PBXIP1 | 8.62E-23 | 3.00E-01 | 0.365 | 0.282 | 2.89E-18 | T_cells |
| NME3 | 8.91E-23 | 2.62E-01 | 0.332 | 0.25 | 2.99E-18 | T_cells |
| BUB3 | 1.84E-22 | 2.78E-01 | 0.382 | 0.296 | 6.16E-18 | T_cells |
| AZIN1 | 2.91E-22 | 2.70E-01 | 0.287 | 0.21 | 9.77E-18 | T_cells |
| KDM2A | 5.07E-22 | 2.85E-01 | 0.327 | 0.247 | 1.70E-17 | T_cells |
| TUBA1A | 6.89E-22 | 2.87E-01 | 0.622 | 0.542 | 2.31E-17 | T_cells |
| CMC2 | 7.28E-22 | 2.70E-01 | 0.329 | 0.25 | 2.44E-17 | T_cells |
| GLRX | 1.21E-21 | 2.75E-01 | 0.408 | 0.323 | 4.07E-17 | T_cells |
| SP110 | 1.23E-21 | 2.94E-01 | 0.287 | 0.212 | 4.12E-17 | T_cells |
| NOL7 | 1.34E-21 | 2.74E-01 | 0.294 | 0.218 | 4.50E-17 | T_cells |
| HEXIM1 | 1.82E-21 | 2.98E-01 | 0.379 | 0.299 | 6.12E-17 | T_cells |
| SELENOW | 3.18E-21 | 2.96E-01 | 0.549 | 0.479 | 1.07E-16 | T_cells |
| DDX3X | 5.99E-21 | 2.68E-01 | 0.73 | 0.671 | 2.01E-16 | T_cells |
| BOD1L1 | 9.92E-21 | 2.68E-01 | 0.292 | 0.216 | 3.33E-16 | T_cells |
| RPS27L | 1.21E-20 | 3.13E-01 | 0.409 | 0.334 | 4.04E-16 | T_cells |
| NAP1L4 | 2.01E-20 | 3.00E-01 | 0.356 | 0.279 | 6.74E-16 | T_cells |
| YWHAH | 3.06E-20 | 2.87E-01 | 0.274 | 0.203 | 1.03E-15 | T_cells |
| KPNA2 | 6.80E-20 | 2.75E-01 | 0.336 | 0.262 | 2.28E-15 | T_cells |
| RAB9A | 7.01E-20 | 3.69E-01 | 0.326 | 0.254 | 2.35E-15 | T_cells |
| EID1 | 9.50E-20 | 2.87E-01 | 0.481 | 0.411 | 3.19E-15 | T_cells |
| SNRPN | 1.01E-19 | 2.58E-01 | 0.373 | 0.294 | 3.37E-15 | T_cells |
| SLA | 1.47E-19 | 3.28E-01 | 0.46 | 0.383 | 4.93E-15 | T_cells |
| FXR1 | 1.68E-19 | 2.50E-01 | 0.324 | 0.25 | 5.63E-15 | T_cells |
| SLC2A3 | 5.65E-19 | 3.68E-01 | 0.524 | 0.457 | 1.90E-14 | T_cells |
| ST13 | 1.24E-18 | 2.65E-01 | 0.487 | 0.423 | 4.16E-14 | T_cells |
| TCP1 | 1.84E-18 | 2.61E-01 | 0.408 | 0.333 | 6.18E-14 | T_cells |
| PKM | 3.06E-17 | 3.22E-01 | 0.383 | 0.314 | 1.03E-12 | T_cells |
| ANKRD11 | 4.23E-17 | 2.61E-01 | 0.39 | 0.318 | 1.42E-12 | T_cells |
| TERF2IP | 5.43E-17 | 2.59E-01 | 0.433 | 0.365 | 1.82E-12 | T_cells |
| FAM129A | 9.29E-17 | 2.69E-01 | 0.259 | 0.194 | 3.12E-12 | T_cells |
| RAB11FIP1 | 4.73E-16 | 2.55E-01 | 0.387 | 0.32 | 1.59E-11 | T_cells |
| DYNC1H1 | 5.10E-16 | 2.76E-01 | 0.333 | 0.267 | 1.71E-11 | T_cells |
| MBNL1 | 9.82E-15 | 2.53E-01 | 0.487 | 0.422 | 3.29E-10 | T_cells |
| TAGAP | 2.67E-11 | 2.86E-01 | 0.458 | 0.406 | 8.95E-07 | T_cells |
| CEBPB | 7.84E-11 | 2.69E-01 | 0.445 | 0.395 | 2.63E-06 | T_cells |
| TNFRSF18 | 1.23E-07 | 4.37E-01 | 0.339 | 0.309 | 0.004114609 | T_cells |
| SAP18 | 2.81E-07 | 2.63E-01 | 0.687 | 0.671 | 0.009437565 | T_cells |
| IL1B | 0 | 5.40634095 | 0.918 | 0.014 | 0 | Monocyte |
| AIF1 | 0 | 4.39050005 | 0.991 | 0.034 | 0 | Monocyte |
| CXCL8 | 0 | 4.26875606 | 0.852 | 0.025 | 0 | Monocyte |
| LST1 | 0 | 3.92731697 | 0.991 | 0.132 | 0 | Monocyte |
| PLAUR | 0 | 3.87560479 | 0.988 | 0.088 | 0 | Monocyte |
| SOD2 | 0 | 3.62178052 | 0.988 | 0.155 | 0 | Monocyte |
| G0S2 | 0 | 3.59168385 | 0.689 | 0.018 | 0 | Monocyte |
| TIMP1 | 0 | 3.51659373 | 0.967 | 0.18 | 0 | Monocyte |
| CXCL2 | 0 | 3.39606496 | 0.562 | 0.023 | 0 | Monocyte |
| MARCKS | 0 | 3.26733954 | 0.894 | 0.015 | 0 | Monocyte |
| MS4A7 | 0 | 3.19984008 | 0.979 | 0.013 | 0 | Monocyte |
| CST3 | 0 | 3.15412618 | 0.994 | 0.107 | 0 | Monocyte |
| FCN1 | 0 | 3.11735677 | 0.861 | 0.002 | 0 | Monocyte |
| MAFB | 0 | 2.84363969 | 0.897 | 0.023 | 0 | Monocyte |
| C5AR1 | 0 | 2.83094849 | 0.94 | 0.004 | 0 | Monocyte |
| CFD | 0 | 2.82043107 | 0.931 | 0.014 | 0 | Monocyte |
| SERPINA1 | 0 | 2.81335446 | 0.967 | 0.031 | 0 | Monocyte |
| ATP2B1-AS1 | 0 | 2.72694077 | 0.949 | 0.209 | 0 | Monocyte |
| LYZ | 0 | 2.64823876 | 0.915 | 0.02 | 0 | Monocyte |
| LYPD2 | 0 | 2.59154585 | 0.456 | 0.001 | 0 | Monocyte |
| OTUD1 | 0 | 2.58263126 | 0.882 | 0.076 | 0 | Monocyte |
| CD68 | 0 | 2.56676909 | 0.97 | 0.021 | 0 | Monocyte |
| SPI1 | 0 | 2.49888548 | 0.912 | 0.013 | 0 | Monocyte |
| CXCL3 | 0 | 2.46657356 | 0.332 | 0.009 | 0 | Monocyte |
| S100A9 | 0 | 2.44106537 | 0.704 | 0.007 | 0 | Monocyte |
| EREG | 0 | 2.39564411 | 0.465 | 0.009 | 0 | Monocyte |
| WARS | 0 | 2.33168166 | 0.903 | 0.034 | 0 | Monocyte |
| LYN | 0 | 2.31255467 | 0.943 | 0.18 | 0 | Monocyte |
| PILRA | 0 | 2.27551167 | 0.918 | 0.006 | 0 | Monocyte |
| CLEC7A | 0 | 2.27381606 | 0.918 | 0.02 | 0 | Monocyte |
| SMIM25 | 0 | 2.26491486 | 0.855 | 0.003 | 0 | Monocyte |
| KLF4 | 0 | 2.25803952 | 0.9 | 0.067 | 0 | Monocyte |
| ASAH1 | 0 | 2.22000091 | 0.967 | 0.227 | 0 | Monocyte |
| LILRB2 | 0 | 2.18900731 | 0.888 | 0.004 | 0 | Monocyte |
| BRI3 | 0 | 2.12543166 | 0.94 | 0.183 | 0 | Monocyte |
| POU2F2 | 0 | 2.06713114 | 0.864 | 0.048 | 0 | Monocyte |
| STXBP2 | 0 | 2.04634539 | 0.915 | 0.175 | 0 | Monocyte |
| IFI30 | 0 | 2.02609648 | 0.755 | 0.027 | 0 | Monocyte |
| ANXA5 | 0 | 1.99252244 | 0.946 | 0.156 | 0 | Monocyte |
| PHACTR1 | 0 | 1.92654275 | 0.659 | 0.017 | 0 | Monocyte |
| CYBB | 0 | 1.90510172 | 0.804 | 0.009 | 0 | Monocyte |
| CXCL16 | 0 | 1.89443007 | 0.807 | 0.026 | 0 | Monocyte |
| APOBEC3A | 0 | 1.89350066 | 0.48 | 0.002 | 0 | Monocyte |
| LILRA5 | 0 | 1.81324059 | 0.743 | 0.003 | 0 | Monocyte |
| CSF1R | 0 | 1.78835031 | 0.764 | 0.005 | 0 | Monocyte |
| TNFAIP2 | 0 | 1.77483612 | 0.65 | 0.006 | 0 | Monocyte |
| TCF7L2 | 0 | 1.73869584 | 0.773 | 0.016 | 0 | Monocyte |
| FGL2 | 0 | 1.73643857 | 0.867 | 0.133 | 0 | Monocyte |
| HMOX1 | 0 | 1.71442277 | 0.61 | 0.045 | 0 | Monocyte |
| CDKN1C | 0 | 1.69258228 | 0.689 | 0.06 | 0 | Monocyte |
| LRRC25 | 0 | 1.67937994 | 0.776 | 0.004 | 0 | Monocyte |
| CFP | 0 | 1.64359749 | 0.773 | 0.009 | 0 | Monocyte |
| CTSZ | 0 | 1.62518612 | 0.882 | 0.141 | 0 | Monocyte |
| SLC31A2 | 0 | 1.61903849 | 0.764 | 0.049 | 0 | Monocyte |
| RNF144B | 0 | 1.5766592 | 0.653 | 0.009 | 0 | Monocyte |
| ITGAX | 0 | 1.5254069 | 0.807 | 0.08 | 0 | Monocyte |
| LGALS3 | 0 | 1.51774159 | 0.837 | 0.112 | 0 | Monocyte |
| HCK | 0 | 1.51674923 | 0.704 | 0.007 | 0 | Monocyte |
| CD300E | 0 | 1.47818392 | 0.571 | 0.001 | 0 | Monocyte |
| NCF2 | 0 | 1.47148549 | 0.698 | 0.004 | 0 | Monocyte |
| CTSB | 0 | 1.45579339 | 0.816 | 0.083 | 0 | Monocyte |
| SLC2A6 | 0 | 1.44458683 | 0.656 | 0.012 | 0 | Monocyte |
| C19orf38 | 0 | 1.43106014 | 0.698 | 0.009 | 0 | Monocyte |
| PECAM1 | 0 | 1.4173649 | 0.713 | 0.051 | 0 | Monocyte |
| BASP1 | 0 | 1.39906044 | 0.628 | 0.061 | 0 | Monocyte |
| SLC43A2 | 0 | 1.3901915 | 0.689 | 0.027 | 0 | Monocyte |
| SLC11A1 | 0 | 1.37190953 | 0.662 | 0.009 | 0 | Monocyte |
| TNFSF13B | 0 | 1.37126097 | 0.707 | 0.046 | 0 | Monocyte |
| IGSF6 | 0 | 1.36666658 | 0.628 | 0.014 | 0 | Monocyte |
| SLC7A7 | 0 | 1.35909051 | 0.686 | 0.004 | 0 | Monocyte |
| FAM49A | 0 | 1.3442049 | 0.737 | 0.066 | 0 | Monocyte |
| S100A8 | 0 | 1.32592966 | 0.302 | 0.003 | 0 | Monocyte |
| MNDA | 0 | 1.32533617 | 0.574 | 0.011 | 0 | Monocyte |
| CSTA | 0 | 1.31940517 | 0.568 | 0.002 | 0 | Monocyte |
| FPR1 | 0 | 1.2717368 | 0.574 | 0.002 | 0 | Monocyte |
| CKB | 0 | 1.25225868 | 0.583 | 0.021 | 0 | Monocyte |
| RRAS | 0 | 1.23183627 | 0.631 | 0.035 | 0 | Monocyte |
| CD86 | 0 | 1.2112363 | 0.577 | 0.008 | 0 | Monocyte |
| ZNF703 | 0 | 1.20888852 | 0.523 | 0.014 | 0 | Monocyte |
| LMO2 | 0 | 1.17939537 | 0.61 | 0.004 | 0 | Monocyte |
| CTSL | 0 | 1.17343186 | 0.598 | 0.012 | 0 | Monocyte |
| IRAK3 | 0 | 1.14926049 | 0.637 | 0.05 | 0 | Monocyte |
| IFNGR2 | 0 | 1.13173268 | 0.601 | 0.041 | 0 | Monocyte |
| TIMP2 | 0 | 1.12903746 | 0.644 | 0.031 | 0 | Monocyte |
| ETS2 | 0 | 1.12504305 | 0.526 | 0.03 | 0 | Monocyte |
| FCGRT | 0 | 1.11042906 | 0.698 | 0.054 | 0 | Monocyte |
| KYNU | 0 | 1.10219572 | 0.505 | 0.007 | 0 | Monocyte |
| SNX18 | 0 | 1.10100624 | 0.65 | 0.05 | 0 | Monocyte |
| C9orf72 | 0 | 1.08194338 | 0.628 | 0.048 | 0 | Monocyte |
| SNN | 0 | 1.08061423 | 0.598 | 0.031 | 0 | Monocyte |
| FFAR2 | 0 | 1.07458327 | 0.438 | 0.005 | 0 | Monocyte |
| FCGR2A | 0 | 1.04071309 | 0.541 | 0.011 | 0 | Monocyte |
| LILRB3 | 0 | 1.01572043 | 0.55 | 0.007 | 0 | Monocyte |
| RAB31 | 0 | 1.01016409 | 0.508 | 0.009 | 0 | Monocyte |
| RXRA | 0 | 1.00954853 | 0.61 | 0.062 | 0 | Monocyte |
| VMO1 | 0 | 1.0059869 | 0.317 | 0.006 | 0 | Monocyte |
| SIGLEC10 | 0 | 1.00521446 | 0.502 | 0.006 | 0 | Monocyte |
| MPEG1 | 0 | 0.99227547 | 0.505 | 0.008 | 0 | Monocyte |
| RAB32 | 0 | 0.98785506 | 0.565 | 0.024 | 0 | Monocyte |
| C20orf27 | 0 | 0.97526353 | 0.589 | 0.055 | 0 | Monocyte |
| EMILIN2 | 0 | 0.97205685 | 0.544 | 0.012 | 0 | Monocyte |
| CLEC12A | 0 | 0.96900882 | 0.486 | 0.022 | 0 | Monocyte |
| CCRL2 | 0 | 0.96728489 | 0.432 | 0.028 | 0 | Monocyte |
| HCAR3 | 0 | 0.96109136 | 0.372 | 0.001 | 0 | Monocyte |
| PAPSS2 | 0 | 0.94620379 | 0.456 | 0.001 | 0 | Monocyte |
| ADGRE2 | 0 | 0.89891187 | 0.459 | 0.014 | 0 | Monocyte |
| SECTM1 | 0 | 0.89393366 | 0.453 | 0.007 | 0 | Monocyte |
| LILRB1 | 0 | 0.8920288 | 0.459 | 0.015 | 0 | Monocyte |
| CPVL | 0 | 0.86068808 | 0.541 | 0.014 | 0 | Monocyte |
| AQP9 | 0 | 0.85972826 | 0.338 | 0.002 | 0 | Monocyte |
| LY96 | 0 | 0.8545226 | 0.55 | 0.026 | 0 | Monocyte |
| SLC8A1 | 0 | 0.85245456 | 0.444 | 0.007 | 0 | Monocyte |
| MEF2C | 0 | 0.84982583 | 0.432 | 0.029 | 0 | Monocyte |
| ABCA1 | 0 | 0.84887212 | 0.366 | 0.014 | 0 | Monocyte |
| SMPDL3A | 0 | 0.8468239 | 0.432 | 0.007 | 0 | Monocyte |
| NRGN | 0 | 0.84664258 | 0.369 | 0.002 | 0 | Monocyte |
| TREM1 | 0 | 0.84540618 | 0.347 | 0.001 | 0 | Monocyte |
| CPPED1 | 0 | 0.84239047 | 0.453 | 0.024 | 0 | Monocyte |
| DMXL2 | 0 | 0.81028553 | 0.426 | 0.004 | 0 | Monocyte |
| SIDT2 | 0 | 0.80288195 | 0.465 | 0.023 | 0 | Monocyte |
| LYL1 | 0 | 0.79057593 | 0.441 | 0.023 | 0 | Monocyte |
| CD4 | 0 | 0.7667998 | 0.465 | 0.023 | 0 | Monocyte |
| MYD88 | 0 | 0.74810086 | 0.489 | 0.04 | 0 | Monocyte |
| SCIMP | 0 | 0.74651543 | 0.432 | 0.007 | 0 | Monocyte |
| CD302 | 0 | 0.72940392 | 0.393 | 0.007 | 0 | Monocyte |
| C1QA | 0 | 0.72331449 | 0.417 | 0.019 | 0 | Monocyte |
| AC245128.3 | 0 | 0.72132858 | 0.381 | 0.001 | 0 | Monocyte |
| FGD4 | 0 | 0.71537398 | 0.399 | 0.004 | 0 | Monocyte |
| BTK | 0 | 0.70624615 | 0.375 | 0.005 | 0 | Monocyte |
| LINC02432 | 0 | 0.70543098 | 0.36 | 0 | 0 | Monocyte |
| RBM47 | 0 | 0.69024333 | 0.347 | 0.008 | 0 | Monocyte |
| CALHM6 | 0 | 0.68695728 | 0.375 | 0.013 | 0 | Monocyte |
| SMCO4 | 0 | 0.68284505 | 0.411 | 0.024 | 0 | Monocyte |
| DENND5A | 0 | 0.67524968 | 0.408 | 0.021 | 0 | Monocyte |
| PRAM1 | 0 | 0.67131787 | 0.39 | 0.007 | 0 | Monocyte |
| ZNF385A | 0 | 0.66422465 | 0.341 | 0.014 | 0 | Monocyte |
| TGFBI | 0 | 0.65760217 | 0.396 | 0.015 | 0 | Monocyte |
| AC064805.1 | 0 | 0.63939922 | 0.344 | 0.001 | 0 | Monocyte |
| CLEC4A | 0 | 0.62840479 | 0.332 | 0.006 | 0 | Monocyte |
| HK3 | 0 | 0.62562129 | 0.323 | 0.002 | 0 | Monocyte |
| MEFV | 0 | 0.62545969 | 0.323 | 0 | 0 | Monocyte |
| SLC15A3 | 0 | 0.62022923 | 0.363 | 0.004 | 0 | Monocyte |
| PLXNB2 | 0 | 0.6187072 | 0.329 | 0.004 | 0 | Monocyte |
| TLR2 | 0 | 0.61426094 | 0.326 | 0.003 | 0 | Monocyte |
| LINC00877 | 0 | 0.60853742 | 0.299 | 0.004 | 0 | Monocyte |
| CD14 | 0 | 0.60375612 | 0.26 | 0.007 | 0 | Monocyte |
| RAB20 | 0 | 0.60244708 | 0.314 | 0.006 | 0 | Monocyte |
| LILRA1 | 0 | 0.59787533 | 0.335 | 0.001 | 0 | Monocyte |
| LY86 | 0 | 0.59625821 | 0.332 | 0.015 | 0 | Monocyte |
| ANPEP | 0 | 0.59459225 | 0.266 | 0.004 | 0 | Monocyte |
| BATF3 | 0 | 0.59395484 | 0.305 | 0.01 | 0 | Monocyte |
| PLD4 | 0 | 0.58415456 | 0.32 | 0.006 | 0 | Monocyte |
| JDP2 | 0 | 0.57239334 | 0.305 | 0.007 | 0 | Monocyte |
| RGS18 | 0 | 0.57109136 | 0.344 | 0.014 | 0 | Monocyte |
| GPBAR1 | 0 | 0.56316997 | 0.287 | 0.002 | 0 | Monocyte |
| TMEM176B | 0 | 0.56236495 | 0.266 | 0.009 | 0 | Monocyte |
| CHST15 | 0 | 0.56090082 | 0.275 | 0.002 | 0 | Monocyte |
| IL10RB-DT | 0 | 0.55229415 | 0.287 | 0.008 | 0 | Monocyte |
| ADAP2 | 0 | 0.54913408 | 0.311 | 0.005 | 0 | Monocyte |
| ZDHHC1 | 0 | 0.54702304 | 0.269 | 0.001 | 0 | Monocyte |
| CD300LF | 0 | 0.5391304 | 0.29 | 0.009 | 0 | Monocyte |
| LILRA2 | 0 | 0.539066 | 0.311 | 0.003 | 0 | Monocyte |
| MCTP1 | 0 | 0.52835276 | 0.308 | 0.006 | 0 | Monocyte |
| LPCAT2 | 0 | 0.52697184 | 0.311 | 0.013 | 0 | Monocyte |
| SLC24A4 | 0 | 0.52191067 | 0.281 | 0.002 | 0 | Monocyte |
| ADGRE1 | 0 | 0.51363334 | 0.272 | 0.002 | 0 | Monocyte |
| ALDH3B1 | 0 | 0.51271276 | 0.293 | 0.002 | 0 | Monocyte |
| ICAM4 | 0 | 0.5083416 | 0.26 | 0.006 | 0 | Monocyte |
| LRRK2 | 0 | 0.49180931 | 0.263 | 0.002 | 0 | Monocyte |
| P2RX1 | 0 | 0.49086163 | 0.272 | 0.008 | 0 | Monocyte |
| ALOX5 | 0 | 0.4896609 | 0.257 | 0.004 | 0 | Monocyte |
| FGD2 | 0 | 0.48766345 | 0.287 | 0.008 | 0 | Monocyte |
| EPB41L3 | 0 | 0.48114845 | 0.263 | 0.002 | 0 | Monocyte |
| 1-Mar | 0-Jan | 0.47887794 | 0.257 | 0.004 | 0 | Monocyte |
| AC096667.1 | 0 | 0.47806365 | 0.275 | 0 | 0 | Monocyte |
| CDA | 0 | 0.45699389 | 0.26 | 0.001 | 0 | Monocyte |
| HNMT | 0 | 0.45445638 | 0.257 | 0.005 | 0 | Monocyte |
| TTYH2 | 0 | 0.44444535 | 0.254 | 0.007 | 0 | Monocyte |
| KDM1B | 0 | 0.44088876 | 0.266 | 0.011 | 0 | Monocyte |
| NPL | 0 | 0.42025502 | 0.254 | 0.008 | 0 | Monocyte |
| TBXAS1 | 3.37E-304 | 9.64E-01 | 0.622 | 0.067 | 1.13E-299 | Monocyte |
| HES1 | 4.64E-304 | 9.04E-01 | 0.372 | 0.024 | 1.56E-299 | Monocyte |
| SULT1A1 | 1.87E-300 | 4.25E-01 | 0.269 | 0.012 | 6.26E-296 | Monocyte |
| CTSH | 1.12E-297 | 8.26E-01 | 0.495 | 0.044 | 3.75E-293 | Monocyte |
| RNF13 | 2.33E-290 | 1.11E+00 | 0.68 | 0.084 | 7.81E-286 | Monocyte |
| CUX1 | 3.53E-290 | 9.49E-01 | 0.583 | 0.062 | 1.18E-285 | Monocyte |
| SESTD1 | 6.60E-289 | 5.15E-01 | 0.299 | 0.016 | 2.21E-284 | Monocyte |
| RNF130 | 2.76E-282 | 1.19E+00 | 0.77 | 0.112 | 9.25E-278 | Monocyte |
| NAMPT | 2.88E-282 | 2.86E+00 | 0.982 | 0.303 | 9.65E-278 | Monocyte |
| SH3BP2 | 1.63E-281 | 9.94E-01 | 0.613 | 0.07 | 5.48E-277 | Monocyte |
| AP1S2 | 4.05E-281 | 1.33E+00 | 0.798 | 0.122 | 1.36E-276 | Monocyte |
| MAPKAPK3 | 4.43E-281 | 9.24E-01 | 0.604 | 0.068 | 1.49E-276 | Monocyte |
| TYMP | 7.56E-281 | 2.26E+00 | 0.952 | 0.235 | 2.53E-276 | Monocyte |
| BCL2A1 | 1.53E-278 | 2.57E+00 | 0.973 | 0.258 | 5.12E-274 | Monocyte |
| YBX3 | 7.23E-278 | 1.37E+00 | 0.716 | 0.099 | 2.42E-273 | Monocyte |
| PPM1F | 8.07E-277 | 5.48E-01 | 0.332 | 0.021 | 2.71E-272 | Monocyte |
| UNC119 | 1.37E-275 | 1.19E+00 | 0.701 | 0.093 | 4.60E-271 | Monocyte |
| PLXDC2 | 6.43E-271 | 5.08E-01 | 0.29 | 0.016 | 2.16E-266 | Monocyte |
| MTSS1 | 1.08E-270 | 1.26E+00 | 0.749 | 0.11 | 3.61E-266 | Monocyte |
| NLRP3 | 1.30E-268 | 1.00E+00 | 0.45 | 0.041 | 4.37E-264 | Monocyte |
| AC007032.1 | 5.75E-268 | 5.07E-01 | 0.314 | 0.019 | 1.93E-263 | Monocyte |
| TKT | 7.92E-264 | 2.01E+00 | 0.955 | 0.255 | 2.66E-259 | Monocyte |
| CTSS | 2.35E-261 | 3.15E+00 | 0.976 | 0.353 | 7.87E-257 | Monocyte |
| HLA-DRA | 2.61E-261 | 2.39E+00 | 0.985 | 0.283 | 8.77E-257 | Monocyte |
| NAAA | 4.26E-261 | 8.49E-01 | 0.52 | 0.054 | 1.43E-256 | Monocyte |
| TICAM1 | 7.29E-260 | 8.46E-01 | 0.483 | 0.047 | 2.44E-255 | Monocyte |
| GRINA | 3.68E-259 | 1.01E+00 | 0.604 | 0.075 | 1.23E-254 | Monocyte |
| MPP1 | 3.77E-259 | 5.38E-01 | 0.375 | 0.028 | 1.26E-254 | Monocyte |
| MFSD1 | 1.84E-258 | 7.18E-01 | 0.447 | 0.041 | 6.16E-254 | Monocyte |
| PPM1N | 1.58E-257 | 6.36E-01 | 0.341 | 0.024 | 5.29E-253 | Monocyte |
| PYCARD | 4.25E-255 | 1.66E+00 | 0.888 | 0.188 | 1.43E-250 | Monocyte |
| GRN | 1.89E-254 | 8.29E-01 | 0.58 | 0.068 | 6.34E-250 | Monocyte |
| PSAP | 6.35E-253 | 2.69E+00 | 0.991 | 0.382 | 2.13E-248 | Monocyte |
| PTPN6 | 7.76E-253 | 1.36E+00 | 0.728 | 0.113 | 2.60E-248 | Monocyte |
| ATP6V0A1 | 2.49E-252 | 4.12E-01 | 0.251 | 0.013 | 8.35E-248 | Monocyte |
| COTL1 | 8.80E-250 | 2.90E+00 | 0.997 | 0.398 | 2.95E-245 | Monocyte |
| SNX10 | 2.80E-249 | 1.11E+00 | 0.616 | 0.081 | 9.39E-245 | Monocyte |
| HBEGF | 2.76E-248 | 4.91E-01 | 0.26 | 0.014 | 9.24E-244 | Monocyte |
| NPC2 | 7.67E-247 | 1.92E+00 | 0.961 | 0.27 | 2.57E-242 | Monocyte |
| NOTCH2 | 1.54E-243 | 6.87E-01 | 0.423 | 0.039 | 5.16E-239 | Monocyte |
| ADAM17 | 6.62E-237 | 8.78E-01 | 0.559 | 0.068 | 2.22E-232 | Monocyte |
| NAGA | 1.04E-236 | 4.67E-01 | 0.326 | 0.024 | 3.49E-232 | Monocyte |
| ATP11A | 1.75E-234 | 5.08E-01 | 0.278 | 0.017 | 5.87E-230 | Monocyte |
| KCNMA1 | 4.93E-233 | 5.97E-01 | 0.366 | 0.03 | 1.65E-228 | Monocyte |
| RASGEF1B | 1.04E-232 | 1.63E+00 | 0.906 | 0.204 | 3.49E-228 | Monocyte |
| DPEP2 | 4.75E-231 | 6.64E-01 | 0.396 | 0.035 | 1.59E-226 | Monocyte |
| GNS | 1.01E-230 | 6.22E-01 | 0.426 | 0.041 | 3.40E-226 | Monocyte |
| PLEK | 2.96E-230 | 1.99E+00 | 0.94 | 0.243 | 9.91E-226 | Monocyte |
| ATP2B1 | 9.66E-229 | 1.82E+00 | 0.897 | 0.218 | 3.24E-224 | Monocyte |
| ATP6V1B2 | 1.47E-228 | 7.92E-01 | 0.511 | 0.059 | 4.94E-224 | Monocyte |
| AC015912.3 | 5.13E-228 | 8.07E-01 | 0.402 | 0.038 | 1.72E-223 | Monocyte |
| ZFHX3 | 3.96E-225 | 5.34E-01 | 0.311 | 0.023 | 1.33E-220 | Monocyte |
| KIAA0930 | 2.66E-222 | 5.65E-01 | 0.372 | 0.033 | 8.91E-218 | Monocyte |
| MIR29B2CHG | 5.21E-222 | 5.88E-01 | 0.347 | 0.029 | 1.75E-217 | Monocyte |
| BID | 5.08E-221 | 1.54E+00 | 0.849 | 0.186 | 1.71E-216 | Monocyte |
| RNF141 | 1.14E-219 | 5.94E-01 | 0.344 | 0.029 | 3.82E-215 | Monocyte |
| FEM1C | 4.90E-217 | 7.31E-01 | 0.48 | 0.054 | 1.64E-212 | Monocyte |
| MCOLN1 | 1.64E-211 | 5.30E-01 | 0.341 | 0.029 | 5.50E-207 | Monocyte |
| LCP2 | 1.29E-209 | 1.11E+00 | 0.749 | 0.134 | 4.33E-205 | Monocyte |
| GBP1 | 2.10E-208 | 1.22E+00 | 0.58 | 0.084 | 7.04E-204 | Monocyte |
| S100A11 | 5.10E-208 | 2.57E+00 | 0.988 | 0.548 | 1.71E-203 | Monocyte |
| FTH1 | 1.48E-206 | 2.81E+00 | 1 | 0.993 | 4.98E-202 | Monocyte |
| VPS35 | 3.54E-205 | 7.80E-01 | 0.55 | 0.074 | 1.19E-200 | Monocyte |
| MFSD12 | 4.46E-204 | 6.74E-01 | 0.399 | 0.041 | 1.50E-199 | Monocyte |
| IQSEC1 | 1.81E-203 | 7.56E-01 | 0.477 | 0.057 | 6.07E-199 | Monocyte |
| EHBP1L1 | 3.43E-203 | 7.73E-01 | 0.556 | 0.076 | 1.15E-198 | Monocyte |
| ADA2 | 1.48E-201 | 7.60E-01 | 0.462 | 0.055 | 4.96E-197 | Monocyte |
| SAT1 | 9.12E-201 | 2.75E+00 | 1 | 0.835 | 3.06E-196 | Monocyte |
| HSBP1 | 1.96E-198 | 1.06E+00 | 0.734 | 0.135 | 6.56E-194 | Monocyte |
| MTHFR | 9.00E-198 | 5.40E-01 | 0.314 | 0.026 | 3.02E-193 | Monocyte |
| FTL | 7.35E-197 | 2.68E+00 | 1 | 0.962 | 2.47E-192 | Monocyte |
| NUP214 | 3.11E-196 | 6.00E-01 | 0.414 | 0.045 | 1.04E-191 | Monocyte |
| C1orf162 | 3.39E-196 | 1.27E+00 | 0.858 | 0.189 | 1.14E-191 | Monocyte |
| TMEM154 | 9.99E-196 | 4.26E-01 | 0.272 | 0.02 | 3.35E-191 | Monocyte |
| CAMK1 | 1.96E-193 | 5.63E-01 | 0.417 | 0.046 | 6.57E-189 | Monocyte |
| MAP3K1 | 5.67E-193 | 6.27E-01 | 0.366 | 0.036 | 1.90E-188 | Monocyte |
| CHCHD10 | 1.79E-192 | 1.20E+00 | 0.776 | 0.159 | 6.01E-188 | Monocyte |
| FAM214B | 3.41E-191 | 4.15E-01 | 0.269 | 0.02 | 1.14E-186 | Monocyte |
| APH1B | 3.19E-190 | 6.12E-01 | 0.384 | 0.04 | 1.07E-185 | Monocyte |
| ARRB1 | 7.57E-190 | 4.74E-01 | 0.344 | 0.032 | 2.54E-185 | Monocyte |
| MBD2 | 3.57E-188 | 8.56E-01 | 0.61 | 0.097 | 1.20E-183 | Monocyte |
| HLA-DQB1 | 5.48E-188 | 9.69E-01 | 0.728 | 0.136 | 1.84E-183 | Monocyte |
| PTGS2 | 8.38E-188 | 1.08E+00 | 0.32 | 0.03 | 2.81E-183 | Monocyte |
| NEAT1 | 2.59E-186 | 2.40E+00 | 0.994 | 0.835 | 8.69E-182 | Monocyte |
| GCA | 4.15E-186 | 4.88E-01 | 0.323 | 0.029 | 1.39E-181 | Monocyte |
| CCDC88A | 4.28E-186 | 6.52E-01 | 0.396 | 0.044 | 1.43E-181 | Monocyte |
| NUMB | 6.02E-186 | 7.00E-01 | 0.471 | 0.06 | 2.02E-181 | Monocyte |
| PSEN1 | 9.28E-186 | 8.11E-01 | 0.565 | 0.085 | 3.11E-181 | Monocyte |
| UNC93B1 | 1.07E-184 | 6.81E-01 | 0.453 | 0.057 | 3.61E-180 | Monocyte |
| ACOT9 | 4.72E-184 | 6.94E-01 | 0.456 | 0.057 | 1.58E-179 | Monocyte |
| HLA-DPA1 | 1.13E-182 | 1.68E+00 | 0.973 | 0.405 | 3.78E-178 | Monocyte |
| ANXA2 | 5.45E-181 | 1.31E+00 | 0.864 | 0.204 | 1.83E-176 | Monocyte |
| TRIB1 | 5.14E-180 | 7.46E-01 | 0.396 | 0.046 | 1.72E-175 | Monocyte |
| ACSL1 | 2.45E-175 | 6.27E-01 | 0.341 | 0.035 | 8.21E-171 | Monocyte |
| FCER1G | 9.45E-174 | 1.88E+00 | 0.991 | 0.684 | 3.17E-169 | Monocyte |
| HES4 | 1.42E-173 | 1.67E+00 | 0.852 | 0.227 | 4.76E-169 | Monocyte |
| FGR | 1.06E-172 | 1.09E+00 | 0.731 | 0.148 | 3.55E-168 | Monocyte |
| RNASET2 | 2.20E-172 | 1.39E+00 | 0.906 | 0.274 | 7.38E-168 | Monocyte |
| PRKACA | 7.79E-171 | 5.29E-01 | 0.363 | 0.04 | 2.61E-166 | Monocyte |
| HLA-DQA1 | 8.50E-171 | 8.78E-01 | 0.565 | 0.092 | 2.85E-166 | Monocyte |
| SVIL | 4.90E-170 | 4.35E-01 | 0.302 | 0.028 | 1.64E-165 | Monocyte |
| FCGR3A | 9.37E-170 | 1.75E+00 | 0.964 | 0.353 | 3.14E-165 | Monocyte |
| PAG1 | 2.13E-169 | 9.07E-01 | 0.622 | 0.11 | 7.15E-165 | Monocyte |
| NAP1L1 | 3.78E-169 | 1.83E+00 | 0.979 | 0.557 | 1.27E-164 | Monocyte |
| UBE2R2 | 3.99E-169 | 1.11E+00 | 0.804 | 0.184 | 1.34E-164 | Monocyte |
| CD83 | 2.40E-168 | 1.92E+00 | 0.961 | 0.396 | 8.03E-164 | Monocyte |
| IPMK | 7.11E-168 | 3.97E-01 | 0.26 | 0.021 | 2.38E-163 | Monocyte |
| PDLIM5 | 7.67E-168 | 7.16E-01 | 0.514 | 0.077 | 2.57E-163 | Monocyte |
| UBE2D1 | 1.63E-165 | 1.06E+00 | 0.665 | 0.128 | 5.47E-161 | Monocyte |
| SGK1 | 4.94E-165 | 1.49E+00 | 0.683 | 0.144 | 1.66E-160 | Monocyte |
| ARRB2 | 1.66E-164 | 1.09E+00 | 0.761 | 0.169 | 5.56E-160 | Monocyte |
| RRP12 | 5.29E-164 | 5.67E-01 | 0.317 | 0.032 | 1.77E-159 | Monocyte |
| GSTO1 | 5.44E-162 | 1.20E+00 | 0.834 | 0.209 | 1.82E-157 | Monocyte |
| LACTB | 2.63E-160 | 7.47E-01 | 0.568 | 0.095 | 8.82E-156 | Monocyte |
| MTPN | 4.44E-160 | 1.26E+00 | 0.876 | 0.246 | 1.49E-155 | Monocyte |
| S100A6 | 4.97E-160 | 2.15E+00 | 0.988 | 0.643 | 1.67E-155 | Monocyte |
| RNPEP | 2.91E-159 | 6.30E-01 | 0.45 | 0.063 | 9.75E-155 | Monocyte |
| THEMIS2 | 8.05E-159 | 8.71E-01 | 0.647 | 0.121 | 2.70E-154 | Monocyte |
| BACH1 | 7.95E-158 | 8.82E-01 | 0.547 | 0.093 | 2.66E-153 | Monocyte |
| S100A4 | 1.02E-157 | 2.03E+00 | 0.985 | 0.738 | 3.43E-153 | Monocyte |
| GNAI2 | 1.05E-156 | 1.56E+00 | 0.955 | 0.454 | 3.52E-152 | Monocyte |
| STX11 | 6.98E-156 | 1.42E+00 | 0.915 | 0.31 | 2.34E-151 | Monocyte |
| GAA | 7.05E-154 | 4.31E-01 | 0.323 | 0.035 | 2.37E-149 | Monocyte |
| TET2 | 8.57E-154 | 6.56E-01 | 0.444 | 0.062 | 2.87E-149 | Monocyte |
| AGPAT3 | 7.32E-153 | 4.07E-01 | 0.284 | 0.027 | 2.45E-148 | Monocyte |
| COL4A3BP | 3.28E-152 | 8.25E-01 | 0.586 | 0.106 | 1.10E-147 | Monocyte |
| PELI1 | 8.54E-151 | 1.18E+00 | 0.538 | 0.097 | 2.86E-146 | Monocyte |
| PPCDC | 1.15E-149 | 5.67E-01 | 0.375 | 0.047 | 3.86E-145 | Monocyte |
| RP2 | 7.31E-148 | 5.01E-01 | 0.338 | 0.039 | 2.45E-143 | Monocyte |
| C3AR1 | 2.32E-147 | 4.85E-01 | 0.332 | 0.038 | 7.77E-143 | Monocyte |
| FNIP2 | 5.65E-147 | 6.78E-01 | 0.39 | 0.052 | 1.89E-142 | Monocyte |
| TNF | 1.04E-146 | 2.06E+00 | 0.707 | 0.173 | 3.47E-142 | Monocyte |
| NFKBIZ | 1.04E-145 | 1.61E+00 | 0.976 | 0.468 | 3.48E-141 | Monocyte |
| SYNGR2 | 1.31E-145 | 9.32E-01 | 0.713 | 0.158 | 4.41E-141 | Monocyte |
| CMTM6 | 3.15E-144 | 1.38E+00 | 0.906 | 0.323 | 1.06E-139 | Monocyte |
| PAK1 | 3.37E-144 | 6.04E-01 | 0.423 | 0.061 | 1.13E-139 | Monocyte |
| GK | 3.47E-144 | 4.66E-01 | 0.263 | 0.025 | 1.16E-139 | Monocyte |
| TMEM127 | 5.43E-143 | 4.42E-01 | 0.326 | 0.038 | 1.82E-138 | Monocyte |
| SCPEP1 | 9.23E-143 | 5.75E-01 | 0.462 | 0.071 | 3.10E-138 | Monocyte |
| ZFYVE16 | 3.55E-142 | 5.79E-01 | 0.393 | 0.054 | 1.19E-137 | Monocyte |
| AMPD2 | 4.15E-142 | 5.21E-01 | 0.387 | 0.052 | 1.39E-137 | Monocyte |
| PLAGL2 | 2.07E-141 | 3.90E-01 | 0.269 | 0.027 | 6.94E-137 | Monocyte |
| EMP3 | 5.80E-139 | 1.60E+00 | 0.979 | 0.586 | 1.95E-134 | Monocyte |
| LEPROT | 2.41E-138 | 6.36E-01 | 0.526 | 0.092 | 8.09E-134 | Monocyte |
| NRARP | 3.38E-138 | 7.43E-01 | 0.387 | 0.055 | 1.13E-133 | Monocyte |
| IMPDH1 | 6.47E-138 | 4.46E-01 | 0.344 | 0.043 | 2.17E-133 | Monocyte |
| HLA-DRB1 | 1.33E-137 | 1.50E+00 | 0.976 | 0.449 | 4.48E-133 | Monocyte |
| VAMP3 | 2.87E-137 | 5.07E-01 | 0.438 | 0.066 | 9.63E-133 | Monocyte |
| ZYX | 3.43E-137 | 7.22E-01 | 0.589 | 0.113 | 1.15E-132 | Monocyte |
| RNF19B | 4.54E-137 | 8.31E-01 | 0.498 | 0.087 | 1.52E-132 | Monocyte |
| ODF3B | 4.81E-136 | 5.63E-01 | 0.423 | 0.063 | 1.61E-131 | Monocyte |
| ACTB | 1.81E-135 | 1.20E+00 | 1 | 0.989 | 6.07E-131 | Monocyte |
| ARAP1 | 2.18E-135 | 5.21E-01 | 0.363 | 0.048 | 7.32E-131 | Monocyte |
| ARPC3 | 1.05E-134 | 1.26E+00 | 0.982 | 0.794 | 3.51E-130 | Monocyte |
| CEBPA | 1.00E-133 | 4.62E-01 | 0.296 | 0.034 | 3.36E-129 | Monocyte |
| PQLC1 | 1.43E-133 | 5.55E-01 | 0.45 | 0.072 | 4.78E-129 | Monocyte |
| ATG3 | 9.83E-133 | 8.49E-01 | 0.637 | 0.137 | 3.30E-128 | Monocyte |
| ZEB2 | 1.05E-132 | 1.47E+00 | 0.955 | 0.428 | 3.52E-128 | Monocyte |
| CSTB | 1.68E-132 | 1.33E+00 | 0.906 | 0.331 | 5.65E-128 | Monocyte |
| H3F3A | 3.00E-132 | 1.10E+00 | 0.997 | 0.941 | 1.01E-127 | Monocyte |
| ADM | 5.20E-131 | 5.68E-01 | 0.257 | 0.026 | 1.74E-126 | Monocyte |
| FKBP15 | 1.37E-129 | 3.63E-01 | 0.269 | 0.029 | 4.60E-125 | Monocyte |
| MYO1G | 2.74E-129 | 1.02E+00 | 0.776 | 0.203 | 9.18E-125 | Monocyte |
| FAM110A | 8.50E-128 | 8.58E-01 | 0.492 | 0.09 | 2.85E-123 | Monocyte |
| RIPK2 | 9.23E-127 | 9.03E-01 | 0.74 | 0.183 | 3.10E-122 | Monocyte |
| PTP4A2 | 3.10E-126 | 1.35E+00 | 0.927 | 0.437 | 1.04E-121 | Monocyte |
| EVI2B | 1.66E-125 | 1.07E+00 | 0.855 | 0.259 | 5.58E-121 | Monocyte |
| NCOR2 | 1.59E-124 | 4.91E-01 | 0.381 | 0.056 | 5.34E-120 | Monocyte |
| RGS19 | 6.60E-124 | 8.64E-01 | 0.665 | 0.155 | 2.21E-119 | Monocyte |
| TOM1 | 2.39E-123 | 5.34E-01 | 0.402 | 0.062 | 8.02E-119 | Monocyte |
| ZNF267 | 4.28E-123 | 7.68E-01 | 0.544 | 0.109 | 1.44E-118 | Monocyte |
| MED13L | 1.37E-122 | 6.02E-01 | 0.468 | 0.083 | 4.60E-118 | Monocyte |
| CORO1B | 4.68E-122 | 7.13E-01 | 0.562 | 0.115 | 1.57E-117 | Monocyte |
| NCOA4 | 1.01E-120 | 6.23E-01 | 0.505 | 0.095 | 3.37E-116 | Monocyte |
| RASSF2 | 3.71E-120 | 4.20E-01 | 0.272 | 0.032 | 1.24E-115 | Monocyte |
| TALDO1 | 6.64E-120 | 9.43E-01 | 0.767 | 0.213 | 2.23E-115 | Monocyte |
| FAM45A | 9.26E-120 | 7.21E-01 | 0.511 | 0.099 | 3.11E-115 | Monocyte |
| ARPC5 | 1.02E-118 | 1.09E+00 | 0.894 | 0.314 | 3.42E-114 | Monocyte |
| TYROBP | 3.81E-118 | 1.13E+00 | 0.994 | 0.764 | 1.28E-113 | Monocyte |
| PPT1 | 5.63E-117 | 6.67E-01 | 0.526 | 0.105 | 1.89E-112 | Monocyte |
| SERPINB9 | 6.56E-117 | 1.10E+00 | 0.755 | 0.218 | 2.20E-112 | Monocyte |
| STX7 | 1.42E-116 | 4.16E-01 | 0.344 | 0.049 | 4.76E-112 | Monocyte |
| DOCK5 | 1.52E-116 | 4.68E-01 | 0.314 | 0.042 | 5.09E-112 | Monocyte |
| GCH1 | 2.33E-116 | 8.76E-01 | 0.716 | 0.182 | 7.82E-112 | Monocyte |
| IFITM3 | 3.95E-116 | 1.73E+00 | 0.961 | 0.495 | 1.32E-111 | Monocyte |
| CDKN1A | 8.99E-116 | 1.07E+00 | 0.707 | 0.195 | 3.01E-111 | Monocyte |
| RNH1 | 2.00E-115 | 9.34E-01 | 0.798 | 0.229 | 6.72E-111 | Monocyte |
| GDI2 | 2.12E-115 | 1.07E+00 | 0.864 | 0.29 | 7.12E-111 | Monocyte |
| GPR137B | 2.17E-115 | 3.99E-01 | 0.284 | 0.035 | 7.29E-111 | Monocyte |
| RALB | 8.32E-115 | 4.34E-01 | 0.363 | 0.055 | 2.79E-110 | Monocyte |
| CLIP4 | 8.70E-115 | 4.52E-01 | 0.29 | 0.037 | 2.92E-110 | Monocyte |
| MGAT1 | 1.12E-114 | 9.13E-01 | 0.758 | 0.207 | 3.75E-110 | Monocyte |
| BLVRA | 1.14E-114 | 5.18E-01 | 0.438 | 0.076 | 3.83E-110 | Monocyte |
| SPOPL | 2.34E-113 | 3.67E-01 | 0.272 | 0.033 | 7.84E-109 | Monocyte |
| SOCS3 | 3.74E-113 | 1.11E+00 | 0.746 | 0.22 | 1.25E-108 | Monocyte |
| OAS1 | 5.21E-113 | 6.34E-01 | 0.429 | 0.076 | 1.75E-108 | Monocyte |
| EPN1 | 5.95E-113 | 5.32E-01 | 0.408 | 0.069 | 1.99E-108 | Monocyte |
| RASSF4 | 1.16E-112 | 4.13E-01 | 0.335 | 0.048 | 3.90E-108 | Monocyte |
| TSPO | 1.20E-112 | 1.22E+00 | 0.931 | 0.409 | 4.01E-108 | Monocyte |
| ZBTB7A | 1.25E-112 | 8.40E-01 | 0.695 | 0.178 | 4.18E-108 | Monocyte |
| SH2B3 | 2.65E-112 | 5.65E-01 | 0.387 | 0.063 | 8.90E-108 | Monocyte |
| CAPNS1 | 2.97E-112 | 9.32E-01 | 0.798 | 0.235 | 9.97E-108 | Monocyte |
| NQO2 | 6.10E-112 | 4.06E-01 | 0.338 | 0.049 | 2.05E-107 | Monocyte |
| MANBA | 4.19E-111 | 4.39E-01 | 0.347 | 0.052 | 1.40E-106 | Monocyte |
| PTPRE | 9.21E-110 | 1.05E+00 | 0.701 | 0.194 | 3.09E-105 | Monocyte |
| UBAC1 | 1.13E-108 | 4.16E-01 | 0.323 | 0.047 | 3.78E-104 | Monocyte |
| FKBP1A | 4.16E-108 | 1.03E+00 | 0.852 | 0.292 | 1.39E-103 | Monocyte |
| B4GALT5 | 1.40E-107 | 6.88E-01 | 0.498 | 0.102 | 4.70E-103 | Monocyte |
| CASP1 | 8.92E-107 | 8.31E-01 | 0.728 | 0.197 | 2.99E-102 | Monocyte |
| WASHC4 | 4.01E-106 | 4.74E-01 | 0.347 | 0.054 | 1.35E-101 | Monocyte |
| TESC | 5.30E-106 | 7.86E-01 | 0.577 | 0.134 | 1.78E-101 | Monocyte |
| PGLS | 6.55E-106 | 9.69E-01 | 0.825 | 0.264 | 2.20E-101 | Monocyte |
| WDR11 | 6.58E-106 | 4.53E-01 | 0.323 | 0.048 | 2.21E-101 | Monocyte |
| NDUFB5 | 5.45E-105 | 8.09E-01 | 0.68 | 0.177 | 1.83E-100 | Monocyte |
| VSIR | 6.26E-105 | 1.17E+00 | 0.876 | 0.335 | 2.10E-100 | Monocyte |
| RAB10 | 1.52E-104 | 6.48E-01 | 0.508 | 0.107 | 5.09E-100 | Monocyte |
| ERICH1 | 3.23E-104 | 6.53E-01 | 0.568 | 0.127 | 1.08E-99 | Monocyte |
| RGS10 | 1.72E-103 | 7.17E-01 | 0.683 | 0.177 | 5.78E-99 | Monocyte |
| QKI | 1.95E-103 | 7.36E-01 | 0.61 | 0.146 | 6.55E-99 | Monocyte |
| NBPF14 | 2.94E-103 | 4.81E-01 | 0.329 | 0.05 | 9.85E-99 | Monocyte |
| NACA | 6.83E-103 | 8.41E-01 | 0.997 | 0.963 | 2.29E-98 | Monocyte |
| CHP1 | 1.12E-102 | 5.06E-01 | 0.462 | 0.09 | 3.75E-98 | Monocyte |
| APLP2 | 1.23E-102 | 8.51E-01 | 0.701 | 0.193 | 4.14E-98 | Monocyte |
| HEBP1 | 1.68E-102 | 4.05E-01 | 0.272 | 0.036 | 5.64E-98 | Monocyte |
| PIK3AP1 | 3.89E-102 | 6.52E-01 | 0.495 | 0.103 | 1.31E-97 | Monocyte |
| PRKCB | 5.00E-102 | 6.73E-01 | 0.598 | 0.142 | 1.68E-97 | Monocyte |
| P2RX4 | 6.10E-102 | 5.13E-01 | 0.405 | 0.073 | 2.05E-97 | Monocyte |
| PRCP | 1.09E-101 | 5.39E-01 | 0.429 | 0.081 | 3.66E-97 | Monocyte |
| HLA-DPB1 | 1.45E-101 | 1.02E+00 | 0.915 | 0.401 | 4.85E-97 | Monocyte |
| CACUL1 | 1.47E-101 | 4.93E-01 | 0.417 | 0.077 | 4.93E-97 | Monocyte |
| GNG5 | 2.20E-101 | 1.05E+00 | 0.943 | 0.437 | 7.37E-97 | Monocyte |
| CSK | 1.03E-100 | 8.46E-01 | 0.743 | 0.212 | 3.45E-96 | Monocyte |
| MAPK6 | 1.04E-100 | 4.54E-01 | 0.372 | 0.063 | 3.50E-96 | Monocyte |
| OGFRL1 | 3.52E-100 | 5.53E-01 | 0.432 | 0.083 | 1.18E-95 | Monocyte |
| PICALM | 9.08E-100 | 4.38E-01 | 0.399 | 0.071 | 3.05E-95 | Monocyte |
| ICAM1 | 2.46E-99 | 1.29E+00 | 0.752 | 0.254 | 8.26E-95 | Monocyte |
| MT-CO2 | 8.51E-99 | 7.18E-01 | 0.994 | 0.989 | 2.85E-94 | Monocyte |
| ACAA1 | 5.05E-98 | 7.25E-01 | 0.604 | 0.152 | 1.69E-93 | Monocyte |
| LYSMD2 | 3.62E-97 | 5.65E-01 | 0.489 | 0.103 | 1.21E-92 | Monocyte |
| ACSL4 | 1.29E-96 | 3.84E-01 | 0.281 | 0.04 | 4.31E-92 | Monocyte |
| IER3 | 2.38E-96 | 1.47E+00 | 0.825 | 0.314 | 7.98E-92 | Monocyte |
| DOCK2 | 4.56E-96 | 5.94E-01 | 0.538 | 0.122 | 1.53E-91 | Monocyte |
| SSFA2 | 8.69E-96 | 3.48E-01 | 0.26 | 0.035 | 2.91E-91 | Monocyte |
| AP2A1 | 2.48E-95 | 4.63E-01 | 0.405 | 0.076 | 8.31E-91 | Monocyte |
| PFDN5 | 2.55E-95 | 8.33E-01 | 0.985 | 0.913 | 8.55E-91 | Monocyte |
| NAGK | 5.84E-95 | 5.84E-01 | 0.441 | 0.09 | 1.96E-90 | Monocyte |
| BCL6 | 6.60E-95 | 6.23E-01 | 0.414 | 0.081 | 2.21E-90 | Monocyte |
| KLF7 | 1.77E-94 | 3.59E-01 | 0.269 | 0.038 | 5.92E-90 | Monocyte |
| SNX2 | 6.83E-94 | 5.88E-01 | 0.444 | 0.092 | 2.29E-89 | Monocyte |
| NINJ1 | 8.60E-94 | 1.25E+00 | 0.934 | 0.488 | 2.88E-89 | Monocyte |
| ARPC1B | 2.50E-93 | 1.04E+00 | 0.921 | 0.41 | 8.38E-89 | Monocyte |
| ATP6V0B | 3.29E-93 | 9.89E-01 | 0.882 | 0.357 | 1.10E-88 | Monocyte |
| YPEL2 | 3.85E-93 | 4.83E-01 | 0.311 | 0.05 | 1.29E-88 | Monocyte |
| NAPRT | 3.85E-93 | 3.82E-01 | 0.275 | 0.04 | 1.29E-88 | Monocyte |
| ATP6V0D1 | 4.19E-93 | 8.34E-01 | 0.764 | 0.239 | 1.40E-88 | Monocyte |
| LGALS9 | 5.81E-93 | 5.59E-01 | 0.492 | 0.109 | 1.95E-88 | Monocyte |
| GNAQ | 5.95E-93 | 4.41E-01 | 0.353 | 0.062 | 2.00E-88 | Monocyte |
| OAZ2 | 8.32E-92 | 6.43E-01 | 0.483 | 0.108 | 2.79E-87 | Monocyte |
| YBX1 | 1.39E-91 | 9.57E-01 | 0.985 | 0.835 | 4.66E-87 | Monocyte |
| CHST7 | 5.40E-91 | 5.26E-01 | 0.39 | 0.074 | 1.81E-86 | Monocyte |
| OAZ1 | 7.40E-91 | 8.86E-01 | 0.988 | 0.888 | 2.48E-86 | Monocyte |
| MIR22HG | 3.25E-90 | 6.67E-01 | 0.532 | 0.128 | 1.09E-85 | Monocyte |
| EGR1 | 7.82E-90 | 1.46E+00 | 0.402 | 0.088 | 2.62E-85 | Monocyte |
| CSGALNACT2 | 8.73E-90 | 4.49E-01 | 0.456 | 0.096 | 2.93E-85 | Monocyte |
| GBP2 | 1.56E-89 | 8.99E-01 | 0.804 | 0.275 | 5.25E-85 | Monocyte |
| VMP1 | 2.45E-89 | 8.35E-01 | 0.785 | 0.255 | 8.23E-85 | Monocyte |
| NADK | 4.44E-89 | 3.58E-01 | 0.305 | 0.049 | 1.49E-84 | Monocyte |
| SH3BGRL3 | 1.43E-88 | 8.76E-01 | 0.997 | 0.875 | 4.80E-84 | Monocyte |
| LGALS1 | 1.59E-88 | 1.03E+00 | 0.976 | 0.525 | 5.32E-84 | Monocyte |
| THAP9-AS1 | 2.32E-88 | 6.03E-01 | 0.529 | 0.127 | 7.76E-84 | Monocyte |
| HIPK3 | 2.58E-88 | 4.44E-01 | 0.341 | 0.06 | 8.65E-84 | Monocyte |
| SWAP70 | 7.50E-88 | 3.72E-01 | 0.284 | 0.044 | 2.52E-83 | Monocyte |
| CNIH4 | 1.82E-87 | 5.43E-01 | 0.45 | 0.097 | 6.11E-83 | Monocyte |
| POLK | 4.16E-87 | 4.16E-01 | 0.311 | 0.052 | 1.40E-82 | Monocyte |
| SNX27 | 4.29E-87 | 3.56E-01 | 0.254 | 0.036 | 1.44E-82 | Monocyte |
| SKAP2 | 6.99E-87 | 5.57E-01 | 0.547 | 0.133 | 2.34E-82 | Monocyte |
| VASP | 1.84E-86 | 9.60E-01 | 0.879 | 0.367 | 6.17E-82 | Monocyte |
| H2AFY | 2.26E-86 | 8.19E-01 | 0.795 | 0.266 | 7.56E-82 | Monocyte |
| PRKCD | 2.38E-86 | 3.10E-01 | 0.254 | 0.036 | 7.98E-82 | Monocyte |
| CTBS | 2.70E-86 | 4.41E-01 | 0.462 | 0.1 | 9.05E-82 | Monocyte |
| SMIM14 | 6.64E-86 | 3.67E-01 | 0.369 | 0.069 | 2.23E-81 | Monocyte |
| STX10 | 1.02E-85 | 5.75E-01 | 0.532 | 0.129 | 3.41E-81 | Monocyte |
| ASAP1 | 1.65E-85 | 5.03E-01 | 0.42 | 0.088 | 5.54E-81 | Monocyte |
| PLIN3 | 7.16E-85 | 4.39E-01 | 0.332 | 0.059 | 2.40E-80 | Monocyte |
| GNB2 | 7.89E-85 | 8.47E-01 | 0.825 | 0.292 | 2.65E-80 | Monocyte |
| SAMHD1 | 1.19E-84 | 6.12E-01 | 0.607 | 0.162 | 3.98E-80 | Monocyte |
| PPIF | 1.38E-84 | 6.46E-01 | 0.498 | 0.119 | 4.64E-80 | Monocyte |
| CTNNA1 | 2.50E-84 | 3.36E-01 | 0.287 | 0.046 | 8.39E-80 | Monocyte |
| WASF2 | 2.82E-84 | 8.66E-01 | 0.81 | 0.289 | 9.44E-80 | Monocyte |
| TMPO | 3.82E-84 | 4.91E-01 | 0.48 | 0.11 | 1.28E-79 | Monocyte |
| RILPL2 | 5.05E-84 | 9.93E-01 | 0.795 | 0.285 | 1.69E-79 | Monocyte |
| LPCAT3 | 5.93E-84 | 3.15E-01 | 0.251 | 0.036 | 1.99E-79 | Monocyte |
| PTPN18 | 6.96E-84 | 5.50E-01 | 0.535 | 0.131 | 2.33E-79 | Monocyte |
| GRK2 | 5.70E-83 | 7.22E-01 | 0.686 | 0.204 | 1.91E-78 | Monocyte |
| PTPRJ | 5.89E-83 | 4.90E-01 | 0.423 | 0.09 | 1.98E-78 | Monocyte |
| HAGH | 1.09E-82 | 4.31E-01 | 0.447 | 0.098 | 3.64E-78 | Monocyte |
| BLOC1S1 | 1.66E-82 | 9.16E-01 | 0.831 | 0.314 | 5.57E-78 | Monocyte |
| GPCPD1 | 4.79E-82 | 6.58E-01 | 0.628 | 0.174 | 1.61E-77 | Monocyte |
| 2-Mar | 1.33E-81 | 4.04E-01 | 0.344 | 0.065 | 4.46E-77 | Monocyte |
| FAM120A | 2.88E-81 | 4.99E-01 | 0.429 | 0.095 | 9.65E-77 | Monocyte |
| HCCS | 3.35E-81 | 3.46E-01 | 0.266 | 0.042 | 1.12E-76 | Monocyte |
| JAML | 8.75E-81 | 5.00E-01 | 0.387 | 0.08 | 2.94E-76 | Monocyte |
| DIAPH2 | 9.37E-81 | 3.36E-01 | 0.254 | 0.038 | 3.14E-76 | Monocyte |
| CEBPB | 4.45E-80 | 1.09E+00 | 0.897 | 0.394 | 1.49E-75 | Monocyte |
| PFKL | 5.62E-80 | 4.97E-01 | 0.438 | 0.099 | 1.89E-75 | Monocyte |
| MAP4K4 | 3.07E-79 | 4.57E-01 | 0.393 | 0.083 | 1.03E-74 | Monocyte |
| SELPLG | 4.23E-79 | 6.21E-01 | 0.634 | 0.181 | 1.42E-74 | Monocyte |
| NR4A1 | 4.59E-79 | 9.17E-01 | 0.888 | 0.37 | 1.54E-74 | Monocyte |
| LFNG | 6.03E-79 | 4.45E-01 | 0.387 | 0.08 | 2.02E-74 | Monocyte |
| C4orf48 | 8.10E-79 | 5.26E-01 | 0.535 | 0.137 | 2.72E-74 | Monocyte |
| PLEKHO2 | 9.13E-79 | 3.68E-01 | 0.275 | 0.045 | 3.06E-74 | Monocyte |
| ZNF106 | 1.14E-78 | 5.58E-01 | 0.474 | 0.115 | 3.82E-74 | Monocyte |
| DICER1 | 1.35E-78 | 3.36E-01 | 0.29 | 0.049 | 4.54E-74 | Monocyte |
| RCOR1 | 4.48E-78 | 4.45E-01 | 0.393 | 0.083 | 1.50E-73 | Monocyte |
| DUSP1 | 5.62E-78 | 8.59E-01 | 0.994 | 0.944 | 1.89E-73 | Monocyte |
| CD52 | 6.79E-78 | 1.00E+00 | 0.961 | 0.518 | 2.28E-73 | Monocyte |
| EIF4E2 | 7.01E-78 | 4.86E-01 | 0.498 | 0.122 | 2.35E-73 | Monocyte |
| LTA4H | 2.18E-77 | 5.08E-01 | 0.423 | 0.095 | 7.30E-73 | Monocyte |
| AP2S1 | 2.53E-77 | 7.34E-01 | 0.761 | 0.257 | 8.47E-73 | Monocyte |
| SERPINB1 | 2.87E-77 | 8.34E-01 | 0.819 | 0.301 | 9.62E-73 | Monocyte |
| PLEC | 8.38E-77 | 4.45E-01 | 0.335 | 0.065 | 2.81E-72 | Monocyte |
| SLC25A6 | 1.75E-76 | 9.01E-01 | 0.979 | 0.742 | 5.85E-72 | Monocyte |
| MYO9B | 1.92E-76 | 5.67E-01 | 0.498 | 0.125 | 6.44E-72 | Monocyte |
| JPT1 | 2.51E-76 | 9.47E-01 | 0.861 | 0.366 | 8.41E-72 | Monocyte |
| TSC22D2 | 2.64E-76 | 6.09E-01 | 0.514 | 0.134 | 8.86E-72 | Monocyte |
| SDCBP | 2.99E-76 | 8.94E-01 | 0.946 | 0.469 | 1.00E-71 | Monocyte |
| TPP1 | 5.54E-76 | 4.75E-01 | 0.495 | 0.123 | 1.86E-71 | Monocyte |
| C15orf39 | 8.18E-76 | 4.18E-01 | 0.284 | 0.05 | 2.74E-71 | Monocyte |
| PRDX3 | 1.73E-75 | 4.15E-01 | 0.329 | 0.064 | 5.80E-71 | Monocyte |
| ATG16L2 | 2.28E-75 | 3.97E-01 | 0.35 | 0.07 | 7.65E-71 | Monocyte |
| SNX9 | 3.21E-75 | 4.33E-01 | 0.447 | 0.105 | 1.07E-70 | Monocyte |
| NBPF19 | 3.83E-75 | 3.00E-01 | 0.284 | 0.049 | 1.29E-70 | Monocyte |
| CX3CR1 | 4.07E-75 | 3.31E-01 | 0.251 | 0.04 | 1.37E-70 | Monocyte |
| HIVEP1 | 7.24E-75 | 3.66E-01 | 0.278 | 0.048 | 2.43E-70 | Monocyte |
| MX2 | 8.08E-75 | 5.86E-01 | 0.429 | 0.101 | 2.71E-70 | Monocyte |
| CD300A | 2.23E-74 | 5.05E-01 | 0.65 | 0.186 | 7.46E-70 | Monocyte |
| CD74 | 4.39E-74 | 7.85E-01 | 0.994 | 0.772 | 1.47E-69 | Monocyte |
| SLCO3A1 | 4.90E-74 | 4.72E-01 | 0.411 | 0.093 | 1.64E-69 | Monocyte |
| LSM6 | 1.17E-73 | 4.39E-01 | 0.429 | 0.1 | 3.94E-69 | Monocyte |
| COMMD9 | 2.59E-73 | 3.40E-01 | 0.329 | 0.064 | 8.68E-69 | Monocyte |
| RHOQ | 4.02E-73 | 3.84E-01 | 0.366 | 0.077 | 1.35E-68 | Monocyte |
| MICU1 | 5.04E-73 | 2.82E-01 | 0.272 | 0.046 | 1.69E-68 | Monocyte |
| STAT6 | 5.67E-73 | 4.19E-01 | 0.384 | 0.083 | 1.90E-68 | Monocyte |
| CHURC1 | 7.27E-73 | 5.38E-01 | 0.601 | 0.171 | 2.44E-68 | Monocyte |
| GLUL | 1.75E-72 | 8.94E-01 | 0.801 | 0.316 | 5.87E-68 | Monocyte |
| LAMP2 | 2.90E-72 | 4.15E-01 | 0.405 | 0.091 | 9.71E-68 | Monocyte |
| RAPGEF1 | 3.65E-72 | 4.23E-01 | 0.387 | 0.085 | 1.23E-67 | Monocyte |
| RHOG | 9.02E-72 | 8.80E-01 | 0.931 | 0.448 | 3.03E-67 | Monocyte |
| CNPY3 | 1.90E-71 | 6.62E-01 | 0.689 | 0.22 | 6.36E-67 | Monocyte |
| CDC42EP3 | 2.09E-71 | 6.94E-01 | 0.659 | 0.212 | 7.00E-67 | Monocyte |
| SH3BGRL | 2.77E-71 | 8.81E-01 | 0.906 | 0.432 | 9.28E-67 | Monocyte |
| TUBGCP2 | 2.91E-71 | 3.66E-01 | 0.347 | 0.072 | 9.76E-67 | Monocyte |
| PHF19 | 4.73E-71 | 4.90E-01 | 0.369 | 0.081 | 1.59E-66 | Monocyte |
| PKN1 | 5.29E-71 | 5.96E-01 | 0.692 | 0.218 | 1.78E-66 | Monocyte |
| ACTR2 | 8.58E-71 | 8.49E-01 | 0.876 | 0.385 | 2.88E-66 | Monocyte |
| LIMS1 | 1.53E-70 | 6.78E-01 | 0.631 | 0.195 | 5.14E-66 | Monocyte |
| MXD1 | 1.79E-70 | 6.06E-01 | 0.574 | 0.168 | 5.99E-66 | Monocyte |
| GRB2 | 1.92E-70 | 7.75E-01 | 0.773 | 0.286 | 6.45E-66 | Monocyte |
| STAT2 | 2.61E-70 | 3.72E-01 | 0.293 | 0.055 | 8.76E-66 | Monocyte |
| C11orf21 | 4.57E-70 | 4.10E-01 | 0.36 | 0.077 | 1.53E-65 | Monocyte |
| OSER1 | 5.05E-70 | 6.07E-01 | 0.598 | 0.178 | 1.69E-65 | Monocyte |
| HLA-DMB | 5.27E-70 | 3.83E-01 | 0.293 | 0.055 | 1.77E-65 | Monocyte |
| PLK3 | 1.04E-69 | 5.96E-01 | 0.659 | 0.205 | 3.48E-65 | Monocyte |
| TPM3 | 1.54E-69 | 8.55E-01 | 0.97 | 0.671 | 5.17E-65 | Monocyte |
| TAF10 | 2.59E-69 | 7.43E-01 | 0.77 | 0.287 | 8.70E-65 | Monocyte |
| LYPLA1 | 2.93E-69 | 4.52E-01 | 0.471 | 0.119 | 9.82E-65 | Monocyte |
| SFMBT2 | 5.08E-69 | 3.88E-01 | 0.366 | 0.08 | 1.70E-64 | Monocyte |
| ARID3A | 7.67E-69 | 2.66E-01 | 0.251 | 0.042 | 2.57E-64 | Monocyte |
| CD79B | 8.48E-69 | 5.01E-01 | 0.465 | 0.118 | 2.84E-64 | Monocyte |
| MT-CO1 | 8.61E-69 | 4.46E-01 | 0.997 | 0.993 | 2.89E-64 | Monocyte |
| WSB1 | 1.14E-68 | 9.01E-01 | 0.885 | 0.431 | 3.81E-64 | Monocyte |
| BLOC1S6 | 1.99E-68 | 5.28E-01 | 0.459 | 0.118 | 6.66E-64 | Monocyte |
| CTNNB1 | 4.76E-68 | 7.51E-01 | 0.698 | 0.241 | 1.60E-63 | Monocyte |
| UBE2J1 | 6.09E-68 | 7.04E-01 | 0.671 | 0.227 | 2.04E-63 | Monocyte |
| BLVRB | 7.52E-68 | 4.46E-01 | 0.556 | 0.157 | 2.52E-63 | Monocyte |
| TNFRSF1B | 7.61E-68 | 8.97E-01 | 0.931 | 0.464 | 2.55E-63 | Monocyte |
| CTSC | 1.05E-67 | 9.37E-01 | 0.921 | 0.471 | 3.52E-63 | Monocyte |
| STX12 | 2.73E-67 | 3.92E-01 | 0.338 | 0.072 | 9.14E-63 | Monocyte |
| CD37 | 1.04E-66 | 8.03E-01 | 0.97 | 0.689 | 3.50E-62 | Monocyte |
| SIPA1L1 | 1.70E-66 | 4.49E-01 | 0.396 | 0.094 | 5.71E-62 | Monocyte |
| POLE4 | 1.80E-66 | 5.57E-01 | 0.58 | 0.171 | 6.05E-62 | Monocyte |
| RELT | 4.12E-66 | 5.10E-01 | 0.489 | 0.133 | 1.38E-61 | Monocyte |
| OXSR1 | 4.66E-66 | 4.48E-01 | 0.396 | 0.095 | 1.56E-61 | Monocyte |
| MPC1 | 5.02E-66 | 4.46E-01 | 0.495 | 0.133 | 1.68E-61 | Monocyte |
| TCIRG1 | 7.02E-66 | 6.20E-01 | 0.628 | 0.197 | 2.35E-61 | Monocyte |
| FOXO3 | 9.76E-66 | 6.12E-01 | 0.532 | 0.153 | 3.27E-61 | Monocyte |
| BNIP3L | 2.81E-65 | 5.87E-01 | 0.55 | 0.161 | 9.42E-61 | Monocyte |
| PIK3CG | 3.25E-65 | 3.06E-01 | 0.278 | 0.053 | 1.09E-60 | Monocyte |
| ETV6 | 8.03E-65 | 4.39E-01 | 0.287 | 0.056 | 2.69E-60 | Monocyte |
| ARL5B | 1.18E-64 | 4.28E-01 | 0.429 | 0.108 | 3.94E-60 | Monocyte |
| IRF1 | 1.64E-64 | 1.14E+00 | 0.967 | 0.822 | 5.50E-60 | Monocyte |
| HIGD2A | 5.65E-64 | 8.17E-01 | 0.912 | 0.479 | 1.90E-59 | Monocyte |
| LRRFIP1 | 9.09E-64 | 8.92E-01 | 0.961 | 0.613 | 3.05E-59 | Monocyte |
| OSBPL8 | 9.87E-64 | 5.00E-01 | 0.598 | 0.182 | 3.31E-59 | Monocyte |
| CD55 | 1.12E-63 | 8.18E-01 | 0.882 | 0.394 | 3.75E-59 | Monocyte |
| AHNAK | 1.51E-63 | 6.88E-01 | 0.801 | 0.308 | 5.07E-59 | Monocyte |
| ATP13A3 | 2.35E-63 | 3.99E-01 | 0.311 | 0.066 | 7.87E-59 | Monocyte |
| SERP1 | 6.72E-63 | 8.15E-01 | 0.973 | 0.691 | 2.25E-58 | Monocyte |
| PABPC4 | 9.75E-63 | 7.74E-01 | 0.746 | 0.28 | 3.27E-58 | Monocyte |
| ZBTB43 | 1.07E-62 | 6.27E-01 | 0.492 | 0.143 | 3.58E-58 | Monocyte |
| DRAM1 | 1.31E-62 | 3.55E-01 | 0.257 | 0.048 | 4.40E-58 | Monocyte |
| MID1IP1 | 1.98E-62 | 4.98E-01 | 0.36 | 0.085 | 6.64E-58 | Monocyte |
| DCP2 | 2.15E-62 | 4.10E-01 | 0.495 | 0.136 | 7.22E-58 | Monocyte |
| VAMP5 | 2.17E-62 | 7.88E-01 | 0.725 | 0.282 | 7.29E-58 | Monocyte |
| NFATC1 | 3.47E-62 | 3.70E-01 | 0.378 | 0.09 | 1.16E-57 | Monocyte |
| ADPGK | 3.72E-62 | 4.05E-01 | 0.502 | 0.138 | 1.25E-57 | Monocyte |
| AHCYL1 | 9.96E-62 | 3.36E-01 | 0.293 | 0.06 | 3.34E-57 | Monocyte |
| LAMTOR2 | 2.33E-61 | 4.77E-01 | 0.559 | 0.166 | 7.82E-57 | Monocyte |
| RHOA | 5.28E-61 | 7.64E-01 | 0.973 | 0.687 | 1.77E-56 | Monocyte |
| AKIRIN2 | 7.60E-61 | 5.95E-01 | 0.68 | 0.233 | 2.55E-56 | Monocyte |
| BORCS7 | 1.65E-60 | 3.99E-01 | 0.514 | 0.146 | 5.53E-56 | Monocyte |
| RBX1 | 2.09E-60 | 5.76E-01 | 0.695 | 0.246 | 6.99E-56 | Monocyte |
| LYST | 3.32E-60 | 7.10E-01 | 0.785 | 0.315 | 1.11E-55 | Monocyte |
| FLII | 5.90E-60 | 3.05E-01 | 0.347 | 0.079 | 1.98E-55 | Monocyte |
| PQLC3 | 1.06E-59 | 3.82E-01 | 0.432 | 0.113 | 3.55E-55 | Monocyte |
| HOOK3 | 1.22E-59 | 4.10E-01 | 0.384 | 0.095 | 4.10E-55 | Monocyte |
| TRIM38 | 2.06E-59 | 4.61E-01 | 0.486 | 0.138 | 6.92E-55 | Monocyte |
| RIOK3 | 2.41E-59 | 5.66E-01 | 0.592 | 0.192 | 8.08E-55 | Monocyte |
| TUT7 | 2.55E-59 | 4.17E-01 | 0.396 | 0.1 | 8.57E-55 | Monocyte |
| TAOK3 | 3.28E-59 | 5.36E-01 | 0.625 | 0.203 | 1.10E-54 | Monocyte |
| CDC42 | 5.82E-59 | 7.25E-01 | 0.967 | 0.748 | 1.95E-54 | Monocyte |
| PSTPIP2 | 6.20E-59 | 3.31E-01 | 0.305 | 0.066 | 2.08E-54 | Monocyte |
| HK1 | 1.32E-58 | 3.45E-01 | 0.311 | 0.068 | 4.44E-54 | Monocyte |
| SBF2 | 1.47E-58 | 3.49E-01 | 0.332 | 0.076 | 4.92E-54 | Monocyte |
| DOCK8 | 1.84E-58 | 4.69E-01 | 0.607 | 0.193 | 6.16E-54 | Monocyte |
| MSRA | 2.37E-58 | 3.24E-01 | 0.302 | 0.065 | 7.95E-54 | Monocyte |
| HCLS1 | 2.50E-58 | 6.37E-01 | 0.843 | 0.342 | 8.37E-54 | Monocyte |
| TMBIM4 | 3.93E-58 | 6.26E-01 | 0.749 | 0.286 | 1.32E-53 | Monocyte |
| RNF149 | 4.28E-58 | 6.34E-01 | 0.631 | 0.214 | 1.43E-53 | Monocyte |
| SAMSN1 | 9.30E-58 | 7.73E-01 | 0.888 | 0.43 | 3.12E-53 | Monocyte |
| ARSA | 1.06E-57 | 2.57E-01 | 0.269 | 0.054 | 3.54E-53 | Monocyte |
| PRELID1 | 2.38E-57 | 7.95E-01 | 0.903 | 0.454 | 7.97E-53 | Monocyte |
| FAM96A | 4.25E-57 | 3.70E-01 | 0.353 | 0.086 | 1.42E-52 | Monocyte |
| RPL8 | 4.97E-57 | 5.13E-01 | 0.994 | 0.982 | 1.67E-52 | Monocyte |
| CYB561A3 | 6.08E-57 | 2.67E-01 | 0.278 | 0.058 | 2.04E-52 | Monocyte |
| MGST3 | 9.49E-57 | 4.73E-01 | 0.583 | 0.187 | 3.18E-52 | Monocyte |
| TFRC | 2.75E-56 | 4.50E-01 | 0.36 | 0.091 | 9.22E-52 | Monocyte |
| SUPT4H1 | 3.06E-56 | 6.60E-01 | 0.767 | 0.301 | 1.03E-51 | Monocyte |
| HLA-DMA | 5.27E-56 | 5.29E-01 | 0.474 | 0.142 | 1.77E-51 | Monocyte |
| KLF3 | 5.87E-56 | 6.20E-01 | 0.689 | 0.252 | 1.97E-51 | Monocyte |
| PTBP3 | 8.14E-56 | 4.46E-01 | 0.492 | 0.146 | 2.73E-51 | Monocyte |
| NUDT16 | 1.07E-55 | 3.61E-01 | 0.317 | 0.074 | 3.58E-51 | Monocyte |
| ARL8B | 1.84E-55 | 3.75E-01 | 0.393 | 0.103 | 6.16E-51 | Monocyte |
| PDCD6IP | 2.04E-55 | 4.23E-01 | 0.441 | 0.124 | 6.84E-51 | Monocyte |
| PHTF2 | 2.13E-55 | 3.17E-01 | 0.459 | 0.128 | 7.15E-51 | Monocyte |
| PRDX1 | 7.44E-55 | 5.75E-01 | 0.795 | 0.334 | 2.49E-50 | Monocyte |
| MMP24OS | 1.28E-54 | 4.65E-01 | 0.589 | 0.191 | 4.28E-50 | Monocyte |
| NCKAP1L | 2.00E-54 | 4.17E-01 | 0.514 | 0.155 | 6.71E-50 | Monocyte |
| HECA | 2.50E-54 | 3.77E-01 | 0.468 | 0.135 | 8.39E-50 | Monocyte |
| S100A10 | 2.82E-54 | 9.05E-01 | 0.961 | 0.736 | 9.45E-50 | Monocyte |
| FLNA | 3.93E-54 | 6.55E-01 | 0.746 | 0.293 | 1.32E-49 | Monocyte |
| STK10 | 5.22E-53 | 4.91E-01 | 0.574 | 0.189 | 1.75E-48 | Monocyte |
| SFT2D2 | 5.41E-53 | 4.52E-01 | 0.42 | 0.118 | 1.82E-48 | Monocyte |
| RYBP | 6.02E-53 | 4.78E-01 | 0.42 | 0.12 | 2.02E-48 | Monocyte |
| RPS6KA1 | 6.02E-53 | 3.27E-01 | 0.384 | 0.1 | 2.02E-48 | Monocyte |
| COX6B1 | 8.67E-53 | 7.33E-01 | 0.946 | 0.601 | 2.91E-48 | Monocyte |
| CLTC | 1.61E-52 | 2.67E-01 | 0.254 | 0.053 | 5.39E-48 | Monocyte |
| KLF6 | 5.76E-52 | 8.06E-01 | 0.991 | 0.886 | 1.93E-47 | Monocyte |
| CD48 | 5.78E-52 | 7.49E-01 | 0.931 | 0.623 | 1.94E-47 | Monocyte |
| AFTPH | 7.34E-52 | 2.87E-01 | 0.287 | 0.065 | 2.46E-47 | Monocyte |
| XIAP | 2.32E-51 | 2.79E-01 | 0.32 | 0.077 | 7.78E-47 | Monocyte |
| MT-CO3 | 1.27E-50 | 4.36E-01 | 0.994 | 0.989 | 4.24E-46 | Monocyte |
| BCL3 | 2.37E-50 | 4.46E-01 | 0.48 | 0.149 | 7.96E-46 | Monocyte |
| CCDC115 | 2.55E-50 | 3.54E-01 | 0.372 | 0.099 | 8.57E-46 | Monocyte |
| TAOK1 | 3.43E-50 | 2.60E-01 | 0.302 | 0.071 | 1.15E-45 | Monocyte |
| LAIR1 | 1.01E-49 | 3.70E-01 | 0.39 | 0.108 | 3.38E-45 | Monocyte |
| NIPSNAP2 | 1.36E-49 | 2.56E-01 | 0.257 | 0.056 | 4.54E-45 | Monocyte |
| CAT | 1.64E-49 | 3.80E-01 | 0.42 | 0.122 | 5.49E-45 | Monocyte |
| ZDHHC20 | 1.83E-49 | 3.29E-01 | 0.329 | 0.083 | 6.13E-45 | Monocyte |
| SLAMF7 | 2.34E-49 | 5.63E-01 | 0.468 | 0.148 | 7.84E-45 | Monocyte |
| DRAP1 | 2.69E-49 | 7.25E-01 | 0.927 | 0.541 | 9.03E-45 | Monocyte |
| EP300 | 3.96E-49 | 3.39E-01 | 0.42 | 0.12 | 1.33E-44 | Monocyte |
| IRF2 | 6.76E-49 | 3.65E-01 | 0.39 | 0.109 | 2.27E-44 | Monocyte |
| MLX | 1.14E-48 | 2.97E-01 | 0.302 | 0.073 | 3.83E-44 | Monocyte |
| SLC25A37 | 2.07E-48 | 3.46E-01 | 0.263 | 0.06 | 6.95E-44 | Monocyte |
| LAMTOR4 | 2.28E-48 | 6.62E-01 | 0.87 | 0.447 | 7.65E-44 | Monocyte |
| SPATA13 | 3.17E-48 | 3.95E-01 | 0.42 | 0.123 | 1.06E-43 | Monocyte |
| NFYC | 3.73E-48 | 3.52E-01 | 0.296 | 0.071 | 1.25E-43 | Monocyte |
| RLIM | 4.54E-48 | 2.56E-01 | 0.266 | 0.06 | 1.52E-43 | Monocyte |
| RBBP8 | 4.87E-48 | 3.30E-01 | 0.29 | 0.07 | 1.63E-43 | Monocyte |
| AKR1A1 | 5.51E-48 | 4.31E-01 | 0.441 | 0.134 | 1.85E-43 | Monocyte |
| MTMR14 | 6.09E-48 | 2.70E-01 | 0.326 | 0.083 | 2.04E-43 | Monocyte |
| UBE2Z | 8.65E-48 | 3.53E-01 | 0.356 | 0.097 | 2.90E-43 | Monocyte |
| IL10RA | 9.09E-48 | 4.39E-01 | 0.707 | 0.267 | 3.05E-43 | Monocyte |
| BORCS8 | 1.12E-47 | 2.73E-01 | 0.296 | 0.072 | 3.76E-43 | Monocyte |
| ACTR3 | 1.58E-47 | 6.43E-01 | 0.867 | 0.43 | 5.29E-43 | Monocyte |
| ADAM10 | 1.93E-47 | 4.82E-01 | 0.511 | 0.169 | 6.46E-43 | Monocyte |
| KMT2C | 2.06E-47 | 3.63E-01 | 0.474 | 0.148 | 6.92E-43 | Monocyte |
| RTN4 | 2.29E-47 | 6.32E-01 | 0.864 | 0.417 | 7.69E-43 | Monocyte |
| CAPZA2 | 3.58E-47 | 5.10E-01 | 0.713 | 0.28 | 1.20E-42 | Monocyte |
| RAP2C | 6.31E-47 | 4.11E-01 | 0.444 | 0.137 | 2.12E-42 | Monocyte |
| PNPLA8 | 7.15E-47 | 4.85E-01 | 0.628 | 0.231 | 2.40E-42 | Monocyte |
| SPG21 | 7.98E-47 | 3.13E-01 | 0.366 | 0.101 | 2.68E-42 | Monocyte |
| CD44 | 8.77E-47 | 7.35E-01 | 0.964 | 0.661 | 2.94E-42 | Monocyte |
| CNDP2 | 9.23E-47 | 3.55E-01 | 0.423 | 0.127 | 3.10E-42 | Monocyte |
| VIM | 1.57E-46 | 7.00E-01 | 0.991 | 0.845 | 5.28E-42 | Monocyte |
| SP110 | 3.61E-46 | 4.57E-01 | 0.598 | 0.215 | 1.21E-41 | Monocyte |
| VAV1 | 4.09E-46 | 2.88E-01 | 0.32 | 0.083 | 1.37E-41 | Monocyte |
| LAP3 | 4.10E-46 | 4.36E-01 | 0.532 | 0.181 | 1.37E-41 | Monocyte |
| MT-ND1 | 4.84E-46 | 5.12E-01 | 0.988 | 0.941 | 1.62E-41 | Monocyte |
| NDUFB3 | 6.85E-46 | 3.39E-01 | 0.492 | 0.159 | 2.30E-41 | Monocyte |
| MYO1F | 1.12E-45 | 4.20E-01 | 0.668 | 0.244 | 3.75E-41 | Monocyte |
| NCOA2 | 1.13E-45 | 2.59E-01 | 0.275 | 0.066 | 3.79E-41 | Monocyte |
| ZFAND6 | 1.19E-45 | 3.30E-01 | 0.502 | 0.161 | 3.98E-41 | Monocyte |
| HIF1A | 1.61E-45 | 4.42E-01 | 0.526 | 0.178 | 5.40E-41 | Monocyte |
| WNK1 | 2.55E-45 | 3.43E-01 | 0.532 | 0.175 | 8.55E-41 | Monocyte |
| USP3 | 2.92E-45 | 3.41E-01 | 0.408 | 0.122 | 9.80E-41 | Monocyte |
| ADA | 3.20E-45 | 3.17E-01 | 0.426 | 0.128 | 1.07E-40 | Monocyte |
| NOP10 | 3.48E-45 | 6.71E-01 | 0.858 | 0.436 | 1.17E-40 | Monocyte |
| NBN | 3.54E-45 | 3.29E-01 | 0.344 | 0.095 | 1.19E-40 | Monocyte |
| MDM2 | 3.75E-45 | 3.16E-01 | 0.308 | 0.079 | 1.26E-40 | Monocyte |
| RAP1B | 4.36E-45 | 7.21E-01 | 0.943 | 0.692 | 1.46E-40 | Monocyte |
| FRAT2 | 7.34E-45 | 3.08E-01 | 0.344 | 0.094 | 2.46E-40 | Monocyte |
| ZFY | 1.07E-44 | 2.62E-01 | 0.266 | 0.062 | 3.59E-40 | Monocyte |
| SNAP29 | 1.17E-44 | 3.04E-01 | 0.317 | 0.083 | 3.92E-40 | Monocyte |
| SHKBP1 | 1.37E-44 | 3.15E-01 | 0.435 | 0.133 | 4.59E-40 | Monocyte |
| CAP1 | 1.50E-44 | 6.55E-01 | 0.894 | 0.471 | 5.02E-40 | Monocyte |
| MT-ND3 | 1.72E-44 | 4.97E-01 | 0.994 | 0.963 | 5.78E-40 | Monocyte |
| GNB1 | 2.28E-44 | 4.50E-01 | 0.662 | 0.25 | 7.63E-40 | Monocyte |
| CCNI | 4.16E-44 | 6.24E-01 | 0.961 | 0.705 | 1.40E-39 | Monocyte |
| RPS26 | 4.70E-44 | 4.98E-01 | 0.985 | 0.88 | 1.58E-39 | Monocyte |
| TSPAN14 | 6.29E-44 | 3.13E-01 | 0.438 | 0.136 | 2.11E-39 | Monocyte |
| SNAP23 | 1.04E-43 | 4.28E-01 | 0.492 | 0.166 | 3.49E-39 | Monocyte |
| SSBP4 | 1.33E-43 | 3.96E-01 | 0.529 | 0.179 | 4.46E-39 | Monocyte |
| ZNF706 | 1.43E-43 | 5.14E-01 | 0.755 | 0.318 | 4.80E-39 | Monocyte |
| PDPK1 | 1.70E-43 | 2.74E-01 | 0.287 | 0.073 | 5.69E-39 | Monocyte |
| METRNL | 2.53E-43 | 5.85E-01 | 0.918 | 0.526 | 8.49E-39 | Monocyte |
| LAPTM5 | 3.03E-43 | 6.20E-01 | 0.991 | 0.706 | 1.02E-38 | Monocyte |
| AL139246.5 | 3.09E-43 | 4.71E-01 | 0.505 | 0.174 | 1.04E-38 | Monocyte |
| NIN | 3.94E-43 | 3.02E-01 | 0.335 | 0.093 | 1.32E-38 | Monocyte |
| EAF1 | 4.18E-43 | 3.75E-01 | 0.272 | 0.068 | 1.40E-38 | Monocyte |
| CALCOCO2 | 4.79E-43 | 3.48E-01 | 0.498 | 0.165 | 1.61E-38 | Monocyte |
| PDE4B | 4.95E-43 | 6.08E-01 | 0.698 | 0.3 | 1.66E-38 | Monocyte |
| GBP5 | 5.71E-43 | 5.20E-01 | 0.408 | 0.131 | 1.91E-38 | Monocyte |
| SERINC1 | 1.22E-42 | 3.65E-01 | 0.544 | 0.189 | 4.09E-38 | Monocyte |
| PPP1R15A | 1.23E-42 | 5.90E-01 | 0.988 | 0.869 | 4.12E-38 | Monocyte |
| ARRDC1 | 1.36E-42 | 2.62E-01 | 0.353 | 0.1 | 4.57E-38 | Monocyte |
| CHMP4B | 3.75E-42 | 3.22E-01 | 0.526 | 0.179 | 1.26E-37 | Monocyte |
| MICAL1 | 3.93E-42 | 2.61E-01 | 0.257 | 0.062 | 1.32E-37 | Monocyte |
| NDUFV3 | 5.01E-42 | 2.73E-01 | 0.254 | 0.061 | 1.68E-37 | Monocyte |
| NCOA3 | 5.10E-42 | 2.82E-01 | 0.302 | 0.08 | 1.71E-37 | Monocyte |
| PURB | 6.23E-42 | 2.93E-01 | 0.305 | 0.082 | 2.09E-37 | Monocyte |
| UQCRC1 | 1.02E-41 | 3.43E-01 | 0.435 | 0.139 | 3.44E-37 | Monocyte |
| SEC11A | 1.03E-41 | 6.73E-01 | 0.852 | 0.444 | 3.44E-37 | Monocyte |
| KLF11 | 1.23E-41 | 2.53E-01 | 0.263 | 0.065 | 4.13E-37 | Monocyte |
| M6PR | 1.38E-41 | 3.80E-01 | 0.607 | 0.223 | 4.63E-37 | Monocyte |
| MT-ATP6 | 1.51E-41 | 4.93E-01 | 0.994 | 0.981 | 5.07E-37 | Monocyte |
| BNIP2 | 1.74E-41 | 3.58E-01 | 0.474 | 0.159 | 5.82E-37 | Monocyte |
| RHOC | 1.83E-41 | 7.18E-01 | 0.867 | 0.489 | 6.12E-37 | Monocyte |
| IFIT2 | 1.89E-41 | 1.02E+00 | 0.338 | 0.102 | 6.34E-37 | Monocyte |
| CYB5R4 | 3.05E-41 | 2.73E-01 | 0.278 | 0.071 | 1.02E-36 | Monocyte |
| RIN3 | 4.47E-41 | 4.29E-01 | 0.592 | 0.225 | 1.50E-36 | Monocyte |
| HACD4 | 5.36E-41 | 3.29E-01 | 0.341 | 0.099 | 1.80E-36 | Monocyte |
| CAPZA1 | 5.62E-41 | 4.28E-01 | 0.683 | 0.268 | 1.89E-36 | Monocyte |
| MOB1A | 6.33E-41 | 5.39E-01 | 0.776 | 0.339 | 2.12E-36 | Monocyte |
| CYBA | 7.39E-41 | 5.10E-01 | 0.985 | 0.906 | 2.48E-36 | Monocyte |
| PTEN | 9.46E-41 | 3.67E-01 | 0.414 | 0.133 | 3.17E-36 | Monocyte |
| TMOD3 | 1.02E-40 | 3.69E-01 | 0.474 | 0.161 | 3.43E-36 | Monocyte |
| TMEM167A | 1.11E-40 | 3.37E-01 | 0.52 | 0.181 | 3.72E-36 | Monocyte |
| RHOB | 1.37E-40 | 7.93E-01 | 0.752 | 0.335 | 4.58E-36 | Monocyte |
| ITGAL | 1.61E-40 | 3.72E-01 | 0.574 | 0.209 | 5.38E-36 | Monocyte |
| PKM | 1.63E-40 | 4.36E-01 | 0.746 | 0.316 | 5.48E-36 | Monocyte |
| BFAR | 2.49E-40 | 3.13E-01 | 0.453 | 0.149 | 8.36E-36 | Monocyte |
| PBX2 | 2.63E-40 | 2.66E-01 | 0.257 | 0.064 | 8.82E-36 | Monocyte |
| HEG1 | 3.47E-40 | 2.51E-01 | 0.251 | 0.062 | 1.16E-35 | Monocyte |
| PTPN1 | 4.63E-40 | 4.83E-01 | 0.568 | 0.22 | 1.55E-35 | Monocyte |
| DBNL | 6.32E-40 | 4.13E-01 | 0.574 | 0.216 | 2.12E-35 | Monocyte |
| SERPINB6 | 8.84E-40 | 2.86E-01 | 0.483 | 0.164 | 2.97E-35 | Monocyte |
| ACAP2 | 9.44E-40 | 3.24E-01 | 0.429 | 0.139 | 3.17E-35 | Monocyte |
| ATP6V1F | 1.15E-39 | 5.86E-01 | 0.782 | 0.37 | 3.85E-35 | Monocyte |
| CYTIP | 1.63E-39 | 6.15E-01 | 0.879 | 0.521 | 5.45E-35 | Monocyte |
| FBXL5 | 2.01E-39 | 2.53E-01 | 0.287 | 0.077 | 6.74E-35 | Monocyte |
| FAM89B | 2.09E-39 | 3.94E-01 | 0.453 | 0.155 | 6.99E-35 | Monocyte |
| DUSP6 | 2.97E-39 | 5.11E-01 | 0.505 | 0.185 | 9.97E-35 | Monocyte |
| RASSF3 | 6.37E-39 | 2.52E-01 | 0.263 | 0.068 | 2.14E-34 | Monocyte |
| CELF1 | 7.79E-39 | 3.54E-01 | 0.444 | 0.149 | 2.61E-34 | Monocyte |
| PCGF5 | 8.23E-39 | 3.81E-01 | 0.607 | 0.231 | 2.76E-34 | Monocyte |
| HMGN2 | 8.33E-39 | 5.39E-01 | 0.918 | 0.579 | 2.80E-34 | Monocyte |
| STMP1 | 8.74E-39 | 3.87E-01 | 0.628 | 0.241 | 2.93E-34 | Monocyte |
| MTHFD2 | 1.31E-38 | 3.30E-01 | 0.411 | 0.135 | 4.38E-34 | Monocyte |
| TUBB | 2.22E-38 | 4.03E-01 | 0.764 | 0.33 | 7.45E-34 | Monocyte |
| ANKRD13A | 3.16E-38 | 2.70E-01 | 0.266 | 0.069 | 1.06E-33 | Monocyte |
| IRF9 | 4.27E-38 | 4.52E-01 | 0.595 | 0.236 | 1.43E-33 | Monocyte |
| DDX21 | 4.29E-38 | 5.06E-01 | 0.767 | 0.346 | 1.44E-33 | Monocyte |
| TRIM8 | 7.14E-38 | 2.53E-01 | 0.381 | 0.118 | 2.39E-33 | Monocyte |
| GMFG | 7.33E-38 | 6.20E-01 | 0.918 | 0.591 | 2.46E-33 | Monocyte |
| PARVG | 9.82E-38 | 3.11E-01 | 0.432 | 0.144 | 3.29E-33 | Monocyte |
| ZFAND5 | 1.99E-37 | 5.64E-01 | 0.918 | 0.527 | 6.69E-33 | Monocyte |
| DRAM2 | 2.02E-37 | 2.79E-01 | 0.375 | 0.118 | 6.76E-33 | Monocyte |
| TGOLN2 | 3.08E-37 | 3.99E-01 | 0.647 | 0.258 | 1.03E-32 | Monocyte |
| SH3BP1 | 5.62E-37 | 2.77E-01 | 0.39 | 0.124 | 1.88E-32 | Monocyte |
| TBC1D1 | 6.38E-37 | 2.76E-01 | 0.366 | 0.114 | 2.14E-32 | Monocyte |
| ELF2 | 7.66E-37 | 3.60E-01 | 0.483 | 0.174 | 2.57E-32 | Monocyte |
| WAC | 7.72E-37 | 4.28E-01 | 0.595 | 0.24 | 2.59E-32 | Monocyte |
| ARPC2 | 8.13E-37 | 5.00E-01 | 0.985 | 0.847 | 2.73E-32 | Monocyte |
| ZFP36 | 8.33E-37 | 5.68E-01 | 0.994 | 0.945 | 2.79E-32 | Monocyte |
| PIM3 | 8.53E-37 | 5.95E-01 | 0.819 | 0.44 | 2.86E-32 | Monocyte |
| ATF3 | 1.59E-36 | 5.18E-01 | 0.84 | 0.429 | 5.32E-32 | Monocyte |
| VPS29 | 1.88E-36 | 4.20E-01 | 0.665 | 0.277 | 6.32E-32 | Monocyte |
| GSTK1 | 2.94E-36 | 5.37E-01 | 0.867 | 0.456 | 9.86E-32 | Monocyte |
| C6orf62 | 3.02E-36 | 3.26E-01 | 0.535 | 0.199 | 1.01E-31 | Monocyte |
| RAB8A | 3.44E-36 | 3.56E-01 | 0.592 | 0.23 | 1.15E-31 | Monocyte |
| EIF4E | 3.67E-36 | 7.86E-01 | 0.483 | 0.185 | 1.23E-31 | Monocyte |
| EIF4EBP1 | 4.13E-36 | 2.58E-01 | 0.326 | 0.098 | 1.38E-31 | Monocyte |
| MEF2D | 4.46E-36 | 2.92E-01 | 0.408 | 0.136 | 1.50E-31 | Monocyte |
| TMEM167B | 4.58E-36 | 2.83E-01 | 0.39 | 0.127 | 1.54E-31 | Monocyte |
| FBXW7 | 6.85E-36 | 2.65E-01 | 0.375 | 0.12 | 2.30E-31 | Monocyte |
| LAPTM4A | 6.97E-36 | 4.71E-01 | 0.782 | 0.358 | 2.34E-31 | Monocyte |
| CHMP5 | 7.36E-36 | 2.59E-01 | 0.477 | 0.169 | 2.47E-31 | Monocyte |
| CARD16 | 8.16E-36 | 4.20E-01 | 0.867 | 0.42 | 2.74E-31 | Monocyte |
| BCL10 | 8.54E-36 | 2.65E-01 | 0.311 | 0.092 | 2.86E-31 | Monocyte |
| AGTRAP | 8.74E-36 | 2.58E-01 | 0.532 | 0.192 | 2.93E-31 | Monocyte |
| LAMTOR3 | 1.97E-35 | 2.67E-01 | 0.29 | 0.084 | 6.61E-31 | Monocyte |
| AIMP1 | 3.11E-35 | 2.63E-01 | 0.399 | 0.133 | 1.04E-30 | Monocyte |
| WAS | 3.37E-35 | 4.57E-01 | 0.656 | 0.282 | 1.13E-30 | Monocyte |
| CHMP2B | 3.41E-35 | 2.77E-01 | 0.474 | 0.168 | 1.14E-30 | Monocyte |
| EIF2S3 | 3.49E-35 | 3.06E-01 | 0.577 | 0.219 | 1.17E-30 | Monocyte |
| SLC44A2 | 3.64E-35 | 2.71E-01 | 0.363 | 0.115 | 1.22E-30 | Monocyte |
| NDUFS2 | 3.68E-35 | 2.69E-01 | 0.366 | 0.117 | 1.23E-30 | Monocyte |
| MIS18BP1 | 4.66E-35 | 3.80E-01 | 0.48 | 0.178 | 1.56E-30 | Monocyte |
| SHOC2 | 4.66E-35 | 2.65E-01 | 0.369 | 0.118 | 1.56E-30 | Monocyte |
| DDX3Y | 5.69E-35 | 3.84E-01 | 0.598 | 0.238 | 1.91E-30 | Monocyte |
| NDUFS7 | 6.57E-35 | 3.74E-01 | 0.634 | 0.256 | 2.20E-30 | Monocyte |
| PRKAG2 | 7.72E-35 | 2.59E-01 | 0.302 | 0.089 | 2.59E-30 | Monocyte |
| GBP4 | 3.03E-34 | 3.14E-01 | 0.396 | 0.133 | 1.01E-29 | Monocyte |
| MSN | 3.55E-34 | 5.29E-01 | 0.882 | 0.496 | 1.19E-29 | Monocyte |
| CREBRF | 4.42E-34 | 3.37E-01 | 0.547 | 0.213 | 1.48E-29 | Monocyte |
| TMEM134 | 5.09E-34 | 3.09E-01 | 0.432 | 0.152 | 1.71E-29 | Monocyte |
| TMEM179B | 6.07E-34 | 2.96E-01 | 0.42 | 0.145 | 2.03E-29 | Monocyte |
| ARHGAP30 | 6.69E-34 | 3.37E-01 | 0.477 | 0.176 | 2.24E-29 | Monocyte |
| CAST | 9.71E-34 | 3.62E-01 | 0.764 | 0.339 | 3.26E-29 | Monocyte |
| PFKFB3 | 9.98E-34 | 4.10E-01 | 0.517 | 0.199 | 3.35E-29 | Monocyte |
| HSD17B11 | 1.01E-33 | 2.91E-01 | 0.562 | 0.214 | 3.39E-29 | Monocyte |
| ARHGAP4 | 1.25E-33 | 2.99E-01 | 0.486 | 0.18 | 4.19E-29 | Monocyte |
| HM13 | 1.40E-33 | 3.29E-01 | 0.541 | 0.211 | 4.69E-29 | Monocyte |
| HDLBP | 1.77E-33 | 2.61E-01 | 0.29 | 0.086 | 5.95E-29 | Monocyte |
| VMA21 | 3.62E-33 | 2.60E-01 | 0.384 | 0.13 | 1.21E-28 | Monocyte |
| MAP3K2 | 4.20E-33 | 3.68E-01 | 0.52 | 0.204 | 1.41E-28 | Monocyte |
| PRR13 | 6.62E-33 | 5.17E-01 | 0.876 | 0.469 | 2.22E-28 | Monocyte |
| PLSCR1 | 9.11E-33 | 3.95E-01 | 0.438 | 0.163 | 3.06E-28 | Monocyte |
| DYNC1LI1 | 9.96E-33 | 2.73E-01 | 0.387 | 0.131 | 3.34E-28 | Monocyte |
| HADHA | 1.31E-32 | 3.42E-01 | 0.547 | 0.217 | 4.41E-28 | Monocyte |
| AC007952.4 | 1.68E-32 | 4.14E-01 | 0.48 | 0.184 | 5.63E-28 | Monocyte |
| DUSP5 | 1.79E-32 | 3.84E-01 | 0.656 | 0.279 | 6.01E-28 | Monocyte |
| HOTAIRM1 | 2.27E-32 | 3.45E-01 | 0.486 | 0.186 | 7.63E-28 | Monocyte |
| ERO1A | 2.33E-32 | 2.84E-01 | 0.275 | 0.082 | 7.80E-28 | Monocyte |
| ATP1A1 | 3.11E-32 | 3.62E-01 | 0.644 | 0.267 | 1.04E-27 | Monocyte |
| TNFSF10 | 3.42E-32 | 4.58E-01 | 0.492 | 0.197 | 1.15E-27 | Monocyte |
| CDKN2D | 3.59E-32 | 3.07E-01 | 0.695 | 0.293 | 1.20E-27 | Monocyte |
| STAG2 | 3.74E-32 | 2.88E-01 | 0.489 | 0.185 | 1.25E-27 | Monocyte |
| PTPRC | 4.55E-32 | 4.98E-01 | 0.967 | 0.763 | 1.53E-27 | Monocyte |
| PNRC2 | 7.36E-32 | 4.43E-01 | 0.619 | 0.269 | 2.47E-27 | Monocyte |
| SNX17 | 8.31E-32 | 3.51E-01 | 0.453 | 0.169 | 2.79E-27 | Monocyte |
| YWHAB | 9.52E-32 | 5.30E-01 | 0.906 | 0.583 | 3.19E-27 | Monocyte |
| ESD | 1.48E-31 | 2.62E-01 | 0.332 | 0.108 | 4.96E-27 | Monocyte |
| ANP32B | 1.60E-31 | 4.35E-01 | 0.837 | 0.414 | 5.38E-27 | Monocyte |
| NANS | 1.82E-31 | 3.08E-01 | 0.52 | 0.204 | 6.10E-27 | Monocyte |
| CYTH1 | 2.65E-31 | 3.36E-01 | 0.628 | 0.261 | 8.88E-27 | Monocyte |
| DECR1 | 2.87E-31 | 2.71E-01 | 0.435 | 0.158 | 9.62E-27 | Monocyte |
| KRAS | 3.63E-31 | 3.18E-01 | 0.508 | 0.196 | 1.22E-26 | Monocyte |
| CCT5 | 4.22E-31 | 3.09E-01 | 0.489 | 0.188 | 1.42E-26 | Monocyte |
| ITM2B | 5.17E-31 | 4.95E-01 | 0.976 | 0.837 | 1.73E-26 | Monocyte |
| GLIPR1 | 9.95E-31 | 3.37E-01 | 0.68 | 0.291 | 3.34E-26 | Monocyte |
| IDH3G | 1.55E-30 | 2.99E-01 | 0.48 | 0.184 | 5.19E-26 | Monocyte |
| SNX3 | 1.78E-30 | 3.86E-01 | 0.801 | 0.386 | 5.98E-26 | Monocyte |
| PGAM1 | 3.57E-30 | 3.96E-01 | 0.74 | 0.343 | 1.20E-25 | Monocyte |
| STAT1 | 4.20E-30 | 2.88E-01 | 0.498 | 0.197 | 1.41E-25 | Monocyte |
| LIMD2 | 4.35E-30 | 4.70E-01 | 0.867 | 0.479 | 1.46E-25 | Monocyte |
| ZSWIM6 | 4.58E-30 | 2.51E-01 | 0.254 | 0.075 | 1.54E-25 | Monocyte |
| RAB5C | 5.13E-30 | 3.74E-01 | 0.698 | 0.312 | 1.72E-25 | Monocyte |
| TACC1 | 9.87E-30 | 3.50E-01 | 0.625 | 0.265 | 3.31E-25 | Monocyte |
| UCP2 | 2.08E-29 | 4.40E-01 | 0.801 | 0.411 | 6.97E-25 | Monocyte |
| CHMP2A | 2.10E-29 | 3.34E-01 | 0.586 | 0.244 | 7.04E-25 | Monocyte |
| SEC14L1 | 2.69E-29 | 3.09E-01 | 0.61 | 0.262 | 9.03E-25 | Monocyte |
| PHIP | 3.06E-29 | 3.40E-01 | 0.462 | 0.181 | 1.03E-24 | Monocyte |
| ICAM2 | 3.28E-29 | 3.17E-01 | 0.613 | 0.259 | 1.10E-24 | Monocyte |
| ARPC4 | 4.26E-29 | 4.46E-01 | 0.84 | 0.457 | 1.43E-24 | Monocyte |
| APBB1IP | 4.42E-29 | 2.54E-01 | 0.483 | 0.188 | 1.48E-24 | Monocyte |
| ATP5MC2 | 5.86E-29 | 4.75E-01 | 0.967 | 0.75 | 1.97E-24 | Monocyte |
| TBCA | 8.90E-29 | 3.95E-01 | 0.807 | 0.395 | 2.99E-24 | Monocyte |
| PAK2 | 1.14E-28 | 3.81E-01 | 0.631 | 0.28 | 3.81E-24 | Monocyte |
| ERGIC1 | 1.23E-28 | 2.81E-01 | 0.483 | 0.19 | 4.12E-24 | Monocyte |
| TNFRSF14 | 1.26E-28 | 3.63E-01 | 0.662 | 0.296 | 4.23E-24 | Monocyte |
| SLC25A5 | 1.28E-28 | 5.18E-01 | 0.931 | 0.652 | 4.30E-24 | Monocyte |
| COX5B | 1.30E-28 | 4.74E-01 | 0.915 | 0.559 | 4.38E-24 | Monocyte |
| CAPZB | 1.48E-28 | 4.85E-01 | 0.927 | 0.554 | 4.98E-24 | Monocyte |
| CORO1A | 1.92E-28 | 4.57E-01 | 0.955 | 0.685 | 6.44E-24 | Monocyte |
| RASSF5 | 1.97E-28 | 3.58E-01 | 0.701 | 0.319 | 6.61E-24 | Monocyte |
| ATP6V1G1 | 3.05E-28 | 4.40E-01 | 0.921 | 0.54 | 1.02E-23 | Monocyte |
| PNP | 5.39E-28 | 3.37E-01 | 0.508 | 0.211 | 1.81E-23 | Monocyte |
| AZIN1 | 9.94E-28 | 2.96E-01 | 0.523 | 0.215 | 3.33E-23 | Monocyte |
| PLEKHB2 | 1.05E-27 | 3.15E-01 | 0.535 | 0.222 | 3.51E-23 | Monocyte |
| ABRACL | 1.10E-27 | 4.19E-01 | 0.737 | 0.361 | 3.69E-23 | Monocyte |
| JMJD1C | 1.39E-27 | 4.97E-01 | 0.698 | 0.339 | 4.66E-23 | Monocyte |
| COX17 | 2.47E-27 | 2.90E-01 | 0.637 | 0.28 | 8.28E-23 | Monocyte |
| PSMD4 | 3.25E-27 | 2.61E-01 | 0.502 | 0.203 | 1.09E-22 | Monocyte |
| DAZAP2 | 4.06E-27 | 4.84E-01 | 0.876 | 0.508 | 1.36E-22 | Monocyte |
| EIF1B | 4.90E-27 | 3.97E-01 | 0.68 | 0.319 | 1.64E-22 | Monocyte |
| TLN1 | 9.73E-27 | 3.48E-01 | 0.689 | 0.324 | 3.26E-22 | Monocyte |
| MT-ND2 | 2.37E-26 | 3.29E-01 | 0.991 | 0.952 | 7.96E-22 | Monocyte |
| WDR1 | 2.68E-26 | 3.18E-01 | 0.607 | 0.272 | 9.00E-22 | Monocyte |
| ATP5PO | 2.87E-26 | 3.60E-01 | 0.834 | 0.409 | 9.62E-22 | Monocyte |
| EIF3A | 6.57E-26 | 3.79E-01 | 0.634 | 0.295 | 2.20E-21 | Monocyte |
| 9-Sep | 8.15E-26 | 3.61E-01 | 0.677 | 0.319 | 2.73E-21 | Monocyte |
| RER1 | 1.78E-25 | 2.61E-01 | 0.508 | 0.213 | 5.98E-21 | Monocyte |
| LAMTOR1 | 2.49E-25 | 2.63E-01 | 0.619 | 0.271 | 8.35E-21 | Monocyte |
| PHB2 | 2.68E-25 | 2.90E-01 | 0.58 | 0.254 | 8.99E-21 | Monocyte |
| RAB1A | 3.54E-25 | 2.95E-01 | 0.592 | 0.262 | 1.19E-20 | Monocyte |
| RIPOR2 | 4.22E-25 | 2.66E-01 | 0.429 | 0.174 | 1.42E-20 | Monocyte |
| ATP5PD | 4.22E-25 | 3.17E-01 | 0.695 | 0.323 | 1.42E-20 | Monocyte |
| ATP6AP2 | 5.40E-25 | 3.30E-01 | 0.683 | 0.322 | 1.81E-20 | Monocyte |
| TWF2 | 6.60E-25 | 2.77E-01 | 0.402 | 0.16 | 2.21E-20 | Monocyte |
| SPN | 1.79E-24 | 3.00E-01 | 0.571 | 0.252 | 6.00E-20 | Monocyte |
| IFIT3 | 2.19E-24 | 5.51E-01 | 0.287 | 0.104 | 7.35E-20 | Monocyte |
| PFDN2 | 2.32E-24 | 2.62E-01 | 0.628 | 0.281 | 7.78E-20 | Monocyte |
| GSTP1 | 2.44E-24 | 3.78E-01 | 0.973 | 0.71 | 8.19E-20 | Monocyte |
| MINOS1 | 2.51E-24 | 2.80E-01 | 0.704 | 0.328 | 8.43E-20 | Monocyte |
| ENY2 | 3.58E-24 | 4.06E-01 | 0.837 | 0.456 | 1.20E-19 | Monocyte |
| UQCRB | 1.76E-23 | 4.36E-01 | 0.976 | 0.792 | 5.90E-19 | Monocyte |
| CDV3 | 5.14E-23 | 3.14E-01 | 0.74 | 0.375 | 1.72E-18 | Monocyte |
| IQGAP1 | 6.26E-23 | 3.61E-01 | 0.861 | 0.481 | 2.10E-18 | Monocyte |
| LCP1 | 6.81E-23 | 4.34E-01 | 0.9 | 0.581 | 2.28E-18 | Monocyte |
| CHD1 | 7.83E-23 | 3.73E-01 | 0.755 | 0.393 | 2.63E-18 | Monocyte |
| KLF2 | 7.85E-23 | 3.62E-01 | 0.97 | 0.656 | 2.63E-18 | Monocyte |
| FYB1 | 8.20E-23 | 3.44E-01 | 0.816 | 0.44 | 2.75E-18 | Monocyte |
| SFT2D1 | 9.10E-23 | 2.55E-01 | 0.502 | 0.213 | 3.05E-18 | Monocyte |
| ZFAS1 | 1.20E-22 | 4.17E-01 | 0.97 | 0.662 | 4.01E-18 | Monocyte |
| EFHD2 | 1.21E-22 | 2.84E-01 | 0.918 | 0.573 | 4.06E-18 | Monocyte |
| MRPS21 | 1.73E-22 | 2.56E-01 | 0.644 | 0.297 | 5.82E-18 | Monocyte |
| CD47 | 2.45E-22 | 3.02E-01 | 0.689 | 0.338 | 8.21E-18 | Monocyte |
| EIF3K | 2.56E-22 | 3.97E-01 | 0.94 | 0.695 | 8.59E-18 | Monocyte |
| PPP1CA | 2.66E-22 | 3.77E-01 | 0.828 | 0.459 | 8.92E-18 | Monocyte |
| SSR3 | 4.34E-22 | 2.53E-01 | 0.568 | 0.259 | 1.46E-17 | Monocyte |
| PPP1CB | 8.86E-22 | 3.22E-01 | 0.825 | 0.455 | 2.97E-17 | Monocyte |
| PPP1R15B | 2.02E-21 | 2.58E-01 | 0.538 | 0.245 | 6.77E-17 | Monocyte |
| INSIG1 | 2.42E-21 | 2.62E-01 | 0.568 | 0.271 | 8.13E-17 | Monocyte |
| WTAP | 4.54E-21 | 2.97E-01 | 0.686 | 0.341 | 1.52E-16 | Monocyte |
| EHD1 | 5.83E-21 | 3.69E-01 | 0.571 | 0.277 | 1.95E-16 | Monocyte |
| MIDN | 1.98E-20 | 3.61E-01 | 0.749 | 0.413 | 6.65E-16 | Monocyte |
| KDM6B | 2.63E-20 | 4.01E-01 | 0.831 | 0.528 | 8.82E-16 | Monocyte |
| NFAT5 | 3.18E-20 | 2.81E-01 | 0.45 | 0.2 | 1.07E-15 | Monocyte |
| REEP5 | 4.56E-20 | 2.52E-01 | 0.665 | 0.33 | 1.53E-15 | Monocyte |
| ATP5MC3 | 8.77E-20 | 3.16E-01 | 0.831 | 0.462 | 2.94E-15 | Monocyte |
| GNAI3 | 9.34E-20 | 2.58E-01 | 0.662 | 0.324 | 3.13E-15 | Monocyte |
| CSNK1A1 | 9.85E-20 | 2.78E-01 | 0.665 | 0.335 | 3.30E-15 | Monocyte |
| MBNL1 | 2.03E-19 | 3.27E-01 | 0.749 | 0.425 | 6.79E-15 | Monocyte |
| ERP29 | 3.43E-19 | 3.39E-01 | 0.798 | 0.425 | 1.15E-14 | Monocyte |
| VDAC2 | 5.05E-19 | 3.05E-01 | 0.743 | 0.397 | 1.69E-14 | Monocyte |
| ATP5PB | 6.01E-19 | 2.61E-01 | 0.595 | 0.295 | 2.01E-14 | Monocyte |
| ARL6IP4 | 6.10E-19 | 3.91E-01 | 0.858 | 0.512 | 2.05E-14 | Monocyte |
| TNFAIP8 | 6.16E-19 | 2.58E-01 | 0.408 | 0.182 | 2.07E-14 | Monocyte |
| ARL6IP5 | 6.57E-19 | 3.21E-01 | 0.885 | 0.552 | 2.20E-14 | Monocyte |
| ATP5F1C | 8.59E-19 | 2.66E-01 | 0.704 | 0.36 | 2.88E-14 | Monocyte |
| COX8A | 9.70E-19 | 3.60E-01 | 0.882 | 0.563 | 3.25E-14 | Monocyte |
| CCNL1 | 1.78E-18 | 3.94E-01 | 0.918 | 0.668 | 5.98E-14 | Monocyte |
| PCBP1 | 2.20E-18 | 3.79E-01 | 0.864 | 0.532 | 7.37E-14 | Monocyte |
| GNAS | 2.62E-18 | 2.79E-01 | 0.931 | 0.647 | 8.80E-14 | Monocyte |
| PSMB3 | 4.64E-18 | 3.19E-01 | 0.813 | 0.453 | 1.56E-13 | Monocyte |
| TUBA1A | 5.63E-18 | 4.05E-01 | 0.834 | 0.547 | 1.89E-13 | Monocyte |
| NFE2L2 | 6.46E-18 | 2.82E-01 | 0.725 | 0.391 | 2.16E-13 | Monocyte |
| MT-CYB | 7.91E-18 | 2.53E-01 | 0.991 | 0.978 | 2.65E-13 | Monocyte |
| FOSB | 8.56E-18 | 4.49E-01 | 0.955 | 0.748 | 2.87E-13 | Monocyte |
| AC020916.1 | 8.82E-18 | 3.88E-01 | 0.758 | 0.464 | 2.96E-13 | Monocyte |
| ELOB | 1.23E-17 | 3.58E-01 | 0.918 | 0.64 | 4.14E-13 | Monocyte |
| BRK1 | 1.50E-17 | 2.74E-01 | 0.822 | 0.445 | 5.03E-13 | Monocyte |
| HLA-E | 1.51E-17 | 2.59E-01 | 0.988 | 0.971 | 5.05E-13 | Monocyte |
| CEBPD | 2.24E-17 | 2.87E-01 | 0.668 | 0.363 | 7.52E-13 | Monocyte |
| ATP5MPL | 2.75E-17 | 2.80E-01 | 0.792 | 0.432 | 9.21E-13 | Monocyte |
| CHMP1B | 4.62E-17 | 4.95E-01 | 0.825 | 0.554 | 1.55E-12 | Monocyte |
| MYL12B | 1.21E-16 | 3.16E-01 | 0.964 | 0.752 | 4.06E-12 | Monocyte |
| ATP6V0E1 | 1.49E-16 | 2.85E-01 | 0.873 | 0.533 | 5.00E-12 | Monocyte |
| PNRC1 | 3.59E-16 | 3.47E-01 | 0.976 | 0.87 | 1.20E-11 | Monocyte |
| PSMB9 | 1.09E-15 | 3.42E-01 | 0.858 | 0.55 | 3.67E-11 | Monocyte |
| STAT3 | 2.20E-15 | 2.62E-01 | 0.713 | 0.4 | 7.37E-11 | Monocyte |
| YME1L1 | 2.35E-15 | 2.54E-01 | 0.671 | 0.378 | 7.88E-11 | Monocyte |
| COX5A | 9.50E-15 | 2.57E-01 | 0.773 | 0.447 | 3.19E-10 | Monocyte |
| UBE2B | 2.53E-14 | 2.50E-01 | 0.789 | 0.46 | 8.49E-10 | Monocyte |
| NDUFA4 | 6.85E-14 | 3.03E-01 | 0.918 | 0.655 | 2.30E-09 | Monocyte |
| SRSF5 | 1.12E-12 | 3.23E-01 | 0.958 | 0.857 | 3.74E-08 | Monocyte |
| COX7B | 1.13E-12 | 2.66E-01 | 0.879 | 0.611 | 3.78E-08 | Monocyte |
| TENT5A | 4.24E-12 | 2.56E-01 | 0.468 | 0.251 | 1.42E-07 | Monocyte |
| CCL3 | 1.27E-05 | 0.40684717 | 0.813 | 0.688 | 0.425945146 | Monocyte |
| CCL3L1 | 0.001689212 | 0.28534063 | 0.559 | 0.448 | 1 | Monocyte |
| C1QB | 0 | 7.01694518 | 0.891 | 0.01 | 0 | Macrophage |
| C1QA | 0 | 6.48971627 | 0.909 | 0.014 | 0 | Macrophage |
| C1QC | 0 | 5.94269692 | 0.884 | 0.004 | 0 | Macrophage |
| APOE | 0 | 5.40912843 | 0.88 | 0.024 | 0 | Macrophage |
| CST3 | 0 | 5.03264295 | 0.953 | 0.109 | 0 | Macrophage |
| SELENOP | 0 | 4.92069997 | 0.804 | 0.01 | 0 | Macrophage |
| CD5L | 0 | 4.90821239 | 0.601 | 0.001 | 0 | Macrophage |
| IFI27 | 0 | 4.41113915 | 0.587 | 0.032 | 0 | Macrophage |
| MARCO | 0 | 4.38816565 | 0.475 | 0.003 | 0 | Macrophage |
| CTSB | 0 | 4.22744559 | 0.721 | 0.086 | 0 | Macrophage |
| AIF1 | 0 | 4.12107051 | 0.736 | 0.039 | 0 | Macrophage |
| FCGRT | 0 | 4.09583275 | 0.699 | 0.055 | 0 | Macrophage |
| SLC40A1 | 0 | 4.05629947 | 0.656 | 0.007 | 0 | Macrophage |
| MS4A7 | 0 | 3.97777446 | 0.638 | 0.019 | 0 | Macrophage |
| MS4A6A | 0 | 3.95475931 | 0.634 | 0.029 | 0 | Macrophage |
| APOC1 | 0 | 3.90748798 | 0.529 | 0.028 | 0 | Macrophage |
| CFD | 0 | 3.61929683 | 0.496 | 0.021 | 0 | Macrophage |
| FOLR2 | 0 | 3.57196619 | 0.554 | 0.002 | 0 | Macrophage |
| CD68 | 0 | 3.55654546 | 0.58 | 0.028 | 0 | Macrophage |
| PDK4 | 0 | 3.43519526 | 0.431 | 0.004 | 0 | Macrophage |
| CD163 | 0 | 3.41164005 | 0.438 | 0.001 | 0 | Macrophage |
| LGMN | 0 | 2.97982497 | 0.391 | 0.019 | 0 | Macrophage |
| CPVL | 0 | 2.86512365 | 0.395 | 0.017 | 0 | Macrophage |
| CD14 | 0 | 2.7857474 | 0.319 | 0.007 | 0 | Macrophage |
| IGSF6 | 0 | 2.75909438 | 0.384 | 0.018 | 0 | Macrophage |
| CETP | 0 | 2.69150614 | 0.264 | 0.001 | 0 | Macrophage |
| MS4A4A | 0 | 2.59889404 | 0.373 | 0.006 | 0 | Macrophage |
| CXCL12 | 0 | 2.31E+00 | 0.272 | 0 | 0 | Macrophage |
| HNMT | 0 | 2.13E+00 | 0.275 | 0.005 | 0 | Macrophage |
| HLA-DQA1 | 8.60E-304 | 3.84E+00 | 0.71 | 0.092 | 2.88E-299 | Macrophage |
| CTSL | 1.02E-285 | 2.58E+00 | 0.315 | 0.016 | 3.43E-281 | Macrophage |
| CSF1R | 3.28E-265 | 2.36E+00 | 0.268 | 0.012 | 1.10E-260 | Macrophage |
| VCAM1 | 1.91E-262 | 2.55E+00 | 0.297 | 0.016 | 6.39E-258 | Macrophage |
| MARCKS | 3.48E-252 | 2.54E+00 | 0.351 | 0.023 | 1.17E-247 | Macrophage |
| CFP | 1.80E-243 | 2.90E+00 | 0.29 | 0.016 | 6.05E-239 | Macrophage |
| LY96 | 1.45E-231 | 2.50E+00 | 0.37 | 0.029 | 4.88E-227 | Macrophage |
| C1orf54 | 1.99E-230 | 1.85E+00 | 0.261 | 0.014 | 6.68E-226 | Macrophage |
| CYBB | 1.03E-214 | 2.11E+00 | 0.275 | 0.017 | 3.46E-210 | Macrophage |
| LIPA | 3.90E-209 | 3.54E+00 | 0.576 | 0.085 | 1.31E-204 | Macrophage |
| HLA-DRA | 2.49E-201 | 3.97E+00 | 0.909 | 0.286 | 8.34E-197 | Macrophage |
| FTL | 1.51E-166 | 5.22E+00 | 0.996 | 0.962 | 5.05E-162 | Macrophage |
| LYZ | 8.51E-160 | 2.65E+00 | 0.312 | 0.029 | 2.85E-155 | Macrophage |
| HMOX1 | 3.97E-151 | 3.19E+00 | 0.388 | 0.049 | 1.33E-146 | Macrophage |
| HLA-DRB1 | 2.60E-136 | 3.56E+00 | 0.888 | 0.451 | 8.71E-132 | Macrophage |
| CD74 | 2.05E-133 | 2.97E+00 | 0.96 | 0.772 | 6.88E-129 | Macrophage |
| FTH1 | 8.47E-128 | 2.15E+00 | 0.996 | 0.993 | 2.84E-123 | Macrophage |
| HLA-DPB1 | 5.64E-124 | 3.35E+00 | 0.826 | 0.403 | 1.89E-119 | Macrophage |
| GRN | 1.06E-117 | 2.78E+00 | 0.413 | 0.071 | 3.56E-113 | Macrophage |
| MAFB | 1.41E-111 | 2.08E+00 | 0.275 | 0.032 | 4.72E-107 | Macrophage |
| CREG1 | 5.08E-107 | 2.63E+00 | 0.417 | 0.078 | 1.70E-102 | Macrophage |
| NPC2 | 5.79E-107 | 3.23E+00 | 0.699 | 0.275 | 1.94E-102 | Macrophage |
| TIMP2 | 2.12E-97 | 1.99E+00 | 0.275 | 0.037 | 7.12E-93 | Macrophage |
| PLD3 | 1.73E-90 | 2.60E+00 | 0.435 | 0.099 | 5.81E-86 | Macrophage |
| TYROBP | 4.64E-88 | 1.91E+00 | 0.909 | 0.766 | 1.56E-83 | Macrophage |
| HLA-DPA1 | 2.33E-87 | 3.24E+00 | 0.746 | 0.409 | 7.80E-83 | Macrophage |
| BLVRB | 1.16E-86 | 3.02E+00 | 0.529 | 0.159 | 3.89E-82 | Macrophage |
| HLA-DQB1 | 9.11E-83 | 2.85E+00 | 0.493 | 0.14 | 3.06E-78 | Macrophage |
| CD63 | 1.55E-78 | 2.24E+00 | 0.833 | 0.725 | 5.21E-74 | Macrophage |
| TMSB10 | 5.69E-78 | 1.12E+00 | 0.989 | 0.988 | 1.91E-73 | Macrophage |
| TSPAN4 | 1.60E-76 | 1.85E+00 | 0.254 | 0.038 | 5.37E-72 | Macrophage |
| LST1 | 3.30E-73 | 2.06E+00 | 0.489 | 0.139 | 1.11E-68 | Macrophage |
| HLA-DMA | 7.84E-72 | 2.60E+00 | 0.467 | 0.143 | 2.63E-67 | Macrophage |
| CTSZ | 3.65E-70 | 2.74E+00 | 0.475 | 0.147 | 1.22E-65 | Macrophage |
| PRDX1 | 2.22E-68 | 2.75E+00 | 0.667 | 0.336 | 7.44E-64 | Macrophage |
| FCER1G | 5.74E-63 | 1.83E+00 | 0.837 | 0.686 | 1.93E-58 | Macrophage |
| CTSS | 7.44E-59 | 2.63E+00 | 0.638 | 0.358 | 2.49E-54 | Macrophage |
| TIMP1 | 1.80E-57 | 2.59E+00 | 0.496 | 0.187 | 6.04E-53 | Macrophage |
| S100A11 | 6.04E-56 | 2.31E+00 | 0.725 | 0.552 | 2.03E-51 | Macrophage |
| HLA-DMB | 5.37E-49 | 1.77E+00 | 0.254 | 0.056 | 1.80E-44 | Macrophage |
| ITM2B | 3.60E-43 | 1.74E+00 | 0.764 | 0.84 | 1.21E-38 | Macrophage |
| BASP1 | 1.85E-42 | 1.89E+00 | 0.264 | 0.066 | 6.20E-38 | Macrophage |
| LGALS3 | 6.54E-42 | 2.47E+00 | 0.355 | 0.119 | 2.19E-37 | Macrophage |
| RNASET2 | 1.23E-40 | 2.54E+00 | 0.518 | 0.28 | 4.12E-36 | Macrophage |
| BRI3 | 3.84E-39 | 2.29E+00 | 0.438 | 0.191 | 1.29E-34 | Macrophage |
| SAT1 | 2.94E-34 | 1.41E+00 | 0.779 | 0.837 | 9.85E-30 | Macrophage |
| TMSB4X | 1.10E-33 | 5.87E-01 | 0.989 | 0.995 | 3.69E-29 | Macrophage |
| PSAP | 7.90E-31 | 2.33E+00 | 0.551 | 0.389 | 2.65E-26 | Macrophage |
| ANXA5 | 1.35E-28 | 2.00E+00 | 0.37 | 0.164 | 4.52E-24 | Macrophage |
| VAMP8 | 1.38E-26 | 1.92E+00 | 0.594 | 0.493 | 4.62E-22 | Macrophage |
| B2M | 1.61E-26 | 4.02E-01 | 0.996 | 0.998 | 5.40E-22 | Macrophage |
| CTSD | 8.17E-26 | 1.94E+00 | 0.634 | 0.634 | 2.74E-21 | Macrophage |
| IFITM3 | 2.26E-25 | 1.93E+00 | 0.605 | 0.5 | 7.56E-21 | Macrophage |
| FCGR3A | 3.26E-24 | 1.68E+00 | 0.54 | 0.359 | 1.09E-19 | Macrophage |
| ATOX1 | 1.59E-23 | 1.88E+00 | 0.37 | 0.182 | 5.33E-19 | Macrophage |
| GLUL | 1.16E-19 | 1.86E+00 | 0.464 | 0.321 | 3.89E-15 | Macrophage |
| SERF2 | 5.14E-19 | 8.10E-01 | 0.793 | 0.962 | 1.72E-14 | Macrophage |
| FGL2 | 7.36E-18 | 1.57E+00 | 0.293 | 0.141 | 2.47E-13 | Macrophage |
| VAMP5 | 3.94E-16 | 2.01E+00 | 0.42 | 0.286 | 1.32E-11 | Macrophage |
| CTSC | 3.52E-15 | 1.65E+00 | 0.529 | 0.476 | 1.18E-10 | Macrophage |
| C1orf162 | 9.76E-15 | 1.85E+00 | 0.333 | 0.196 | 3.27E-10 | Macrophage |
| CEBPD | 1.29E-13 | 1.89E+00 | 0.464 | 0.366 | 4.32E-09 | Macrophage |
| ACTB | 2.07E-13 | 5.04E-01 | 0.942 | 0.99 | 6.94E-09 | Macrophage |
| TYMP | 8.46E-13 | 1.73E+00 | 0.359 | 0.243 | 2.84E-08 | Macrophage |
| CYBA | 3.01E-12 | 8.66E-01 | 0.75 | 0.909 | 1.01E-07 | Macrophage |
| MT2A | 3.73E-12 | 9.50E-01 | 0.725 | 0.758 | 1.25E-07 | Macrophage |
| CSTB | 4.54E-12 | 2.18E+00 | 0.428 | 0.337 | 1.52E-07 | Macrophage |
| PYCARD | 1.01E-11 | 1.77E+00 | 0.315 | 0.196 | 3.40E-07 | Macrophage |
| YBX1 | 2.13E-10 | 9.61E-01 | 0.634 | 0.839 | 7.15E-06 | Macrophage |
| ASAH1 | 2.45E-10 | 1.58E+00 | 0.337 | 0.235 | 8.22E-06 | Macrophage |
| CLTA | 3.78E-10 | 1.50E+00 | 0.326 | 0.213 | 1.27E-05 | Macrophage |
| YWHAB | 7.10E-10 | 3.16E-01 | 0.301 | 0.591 | 2.38E-05 | Macrophage |
| MGST3 | 7.40E-10 | 1.49E+00 | 0.304 | 0.191 | 2.48E-05 | Macrophage |
| ATP5F1E | 1.13E-09 | 7.04E-01 | 0.717 | 0.927 | 3.79E-05 | Macrophage |
| RNF213 | 2.12E-09 | 2.70E-01 | 0.178 | 0.394 | 7.11E-05 | Macrophage |
| EID1 | 3.21E-09 | 2.63E-01 | 0.203 | 0.421 | 0.000107719 | Macrophage |
| UFC1 | 4.16E-09 | 3.47E-01 | 0.214 | 0.449 | 0.000139625 | Macrophage |
| FABP5 | 5.75E-09 | 2.14E+00 | 0.33 | 0.227 | 0.000192852 | Macrophage |
| FAM96B | 5.97E-09 | 2.94E-01 | 0.178 | 0.373 | 0.000200285 | Macrophage |
| CD81 | 6.11E-09 | 1.59E+00 | 0.496 | 0.483 | 0.000204989 | Macrophage |
| NDUFB11 | 7.21E-09 | 2.72E-01 | 0.279 | 0.539 | 0.000241858 | Macrophage |
| COPS9 | 8.48E-09 | 3.28E-01 | 0.214 | 0.441 | 0.000284489 | Macrophage |
| WSB1 | 1.00E-08 | 4.02E-01 | 0.21 | 0.44 | 0.000335303 | Macrophage |
| TMEM230 | 1.03E-08 | 3.51E-01 | 0.17 | 0.365 | 0.000344114 | Macrophage |
| RTRAF | 1.08E-08 | 2.95E-01 | 0.199 | 0.407 | 0.000361528 | Macrophage |
| MZT2B | 1.10E-08 | 3.48E-01 | 0.228 | 0.471 | 0.000369588 | Macrophage |
| PRDX5 | 1.63E-08 | 3.67E-01 | 0.254 | 0.495 | 0.000547188 | Macrophage |
| RHOG | 2.57E-08 | 3.16E-01 | 0.221 | 0.457 | 0.000862802 | Macrophage |
| OAZ1 | 4.44E-08 | 7.34E-01 | 0.688 | 0.892 | 0.001490014 | Macrophage |
| ATP5F1B | 6.50E-08 | 3.59E-01 | 0.254 | 0.484 | 0.002180355 | Macrophage |
| CSNK2B | 1.05E-07 | 2.84E-01 | 0.196 | 0.392 | 0.00353466 | Macrophage |
| C4orf3 | 1.63E-07 | 4.36E-01 | 0.272 | 0.512 | 0.005465919 | Macrophage |
| NDUFB8 | 1.86E-07 | 4.40E-01 | 0.243 | 0.459 | 0.006232637 | Macrophage |
| BRK1 | 2.48E-07 | 3.38E-01 | 0.243 | 0.452 | 0.008310044 | Macrophage |
| RPS19BP1 | 2.58E-07 | 2.55E-01 | 0.167 | 0.335 | 0.008636746 | Macrophage |
| ARPC5 | 2.64E-07 | 1.60E+00 | 0.38 | 0.321 | 0.008851507 | Macrophage |
| MRPS21 | 2.69E-07 | 3.42E-01 | 0.141 | 0.303 | 0.009029427 | Macrophage |
| PSMA1 | 2.85E-07 | 3.15E-01 | 0.192 | 0.384 | 0.009542474 | Macrophage |
| OST4 | 3.41E-07 | 2.60E-01 | 0.409 | 0.768 | 0.011433463 | Macrophage |
| CHCHD10 | 3.87E-07 | 1.25E+00 | 0.257 | 0.167 | 0.012977098 | Macrophage |
| PSMB1 | 4.36E-07 | 2.92E-01 | 0.293 | 0.538 | 0.014608274 | Macrophage |
| KTN1 | 5.88E-07 | 2.71E-01 | 0.145 | 0.303 | 0.019730111 | Macrophage |
| TOP1 | 6.05E-07 | 4.18E-01 | 0.145 | 0.303 | 0.020285019 | Macrophage |
| HMGN3 | 6.17E-07 | 3.91E-01 | 0.178 | 0.358 | 0.020693977 | Macrophage |
| LSM3 | 7.57E-07 | 3.44E-01 | 0.167 | 0.334 | 0.025401034 | Macrophage |
| SELENOF | 7.73E-07 | 3.94E-01 | 0.163 | 0.327 | 0.025917712 | Macrophage |
| GSTO1 | 9.94E-07 | 1.44E+00 | 0.297 | 0.217 | 0.033333784 | Macrophage |
| JPT1 | 1.17E-06 | 2.97E-01 | 0.196 | 0.375 | 0.039078176 | Macrophage |
| COX14 | 1.38E-06 | 0.36709904 | 0.141 | 0.296 | 0.046245969 | Macrophage |
| MOB1A | 1.40E-06 | 0.29719005 | 0.185 | 0.347 | 0.047070998 | Macrophage |
| PPIA | 1.62E-06 | 1.00086125 | 0.62 | 0.81 | 0.054276781 | Macrophage |
| WASF2 | 1.67E-06 | 0.37424556 | 0.145 | 0.298 | 0.055932384 | Macrophage |
| LCP1 | 1.75E-06 | 0.43818121 | 0.322 | 0.588 | 0.058826521 | Macrophage |
| SPCS3 | 2.00E-06 | 0.40499562 | 0.167 | 0.325 | 0.066976442 | Macrophage |
| NDUFA3 | 2.09E-06 | 0.27153309 | 0.196 | 0.364 | 0.070060432 | Macrophage |
| PSMB6 | 2.38E-06 | 0.4912694 | 0.221 | 0.404 | 0.07997078 | Macrophage |
| AURKAIP1 | 2.68E-06 | 0.37978117 | 0.156 | 0.316 | 0.089923218 | Macrophage |
| PRELID1 | 2.90E-06 | 0.39702685 | 0.268 | 0.462 | 0.097397392 | Macrophage |
| COX6A1 | 3.18E-06 | 0.33210362 | 0.377 | 0.682 | 0.106531882 | Macrophage |
| VPS28 | 3.38E-06 | 0.3753587 | 0.228 | 0.422 | 0.11320338 | Macrophage |
| ARPC3 | 3.89E-06 | 0.95807114 | 0.591 | 0.799 | 0.130447373 | Macrophage |
| SNRPG | 3.90E-06 | 0.39052478 | 0.21 | 0.381 | 0.130853513 | Macrophage |
| IFNGR1 | 4.07E-06 | 0.34216078 | 0.21 | 0.385 | 0.136561185 | Macrophage |
| VAPA | 4.29E-06 | 0.44388487 | 0.261 | 0.473 | 0.143924485 | Macrophage |
| SUMO2 | 4.55E-06 | 0.36345318 | 0.413 | 0.75 | 0.152662946 | Macrophage |
| TRAM1 | 4.69E-06 | 0.2880082 | 0.167 | 0.316 | 0.157186192 | Macrophage |
| MTCH1 | 5.79E-06 | 0.26816384 | 0.13 | 0.266 | 0.194095363 | Macrophage |
| UQCRQ | 6.29E-06 | 5.07E-01 | 0.254 | 0.46 | 0.211095652 | Macrophage |
| IMP3 | 6.48E-06 | 3.08E-01 | 0.134 | 0.271 | 0.217464868 | Macrophage |
| NDUFS8 | 6.52E-06 | 3.60E-01 | 0.174 | 0.324 | 0.218786361 | Macrophage |
| SNRPD1 | 6.75E-06 | 2.83E-01 | 0.134 | 0.273 | 0.22625745 | Macrophage |
| TBCA | 9.66E-06 | 3.57E-01 | 0.228 | 0.403 | 0.32410406 | Macrophage |
| ATP5F1D | 9.73E-06 | 5.51E-01 | 0.29 | 0.524 | 0.326344155 | Macrophage |
| CAPZA2 | 1.25E-05 | 2.97E-01 | 0.152 | 0.288 | 0.419015458 | Macrophage |
| TMEM258 | 1.31E-05 | 3.06E-01 | 0.257 | 0.455 | 0.438656903 | Macrophage |
| NDUFA11 | 1.36E-05 | 4.46E-01 | 0.225 | 0.409 | 0.456011294 | Macrophage |
| COX7A2L | 1.58E-05 | 3.44E-01 | 0.185 | 0.335 | 0.528553747 | Macrophage |
| BAX | 1.70E-05 | 3.32E-01 | 0.199 | 0.36 | 0.568567812 | Macrophage |
| FIS1 | 1.85E-05 | 4.51E-01 | 0.196 | 0.351 | 0.61881968 | Macrophage |
| MRPL41 | 1.98E-05 | 3.11E-01 | 0.156 | 0.296 | 0.663937704 | Macrophage |
| RPN2 | 2.69E-05 | 3.40E-01 | 0.156 | 0.289 | 0.902612826 | Macrophage |
| PFDN5 | 2.77E-05 | 5.64E-01 | 0.67 | 0.917 | 0.929357158 | Macrophage |
| PSMA7 | 2.81E-05 | 4.35E-01 | 0.362 | 0.638 | 0.941248268 | Macrophage |
| WDR83OS | 2.92E-05 | 6.02E-01 | 0.29 | 0.505 | 0.977639759 | Macrophage |
| RAC1 | 3.27E-05 | 1.28E+00 | 0.464 | 0.525 | 1 | Macrophage |
| CSDE1 | 3.57E-05 | 4.93E-01 | 0.228 | 0.391 | 1 | Macrophage |
| ANXA2 | 4.21E-05 | 1.38E+00 | 0.275 | 0.212 | 1 | Macrophage |
| ACTR2 | 4.24E-05 | 4.14E-01 | 0.221 | 0.393 | 1 | Macrophage |
| PSMB9 | 4.26E-05 | 5.61E-01 | 0.319 | 0.557 | 1 | Macrophage |
| SSB | 4.43E-05 | 4.08E-01 | 0.178 | 0.315 | 1 | Macrophage |
| POMP | 4.59E-05 | 1.31E+00 | 0.489 | 0.556 | 1 | Macrophage |
| DAD1 | 4.70E-05 | 4.65E-01 | 0.236 | 0.407 | 1 | Macrophage |
| SSU72 | 4.80E-05 | 4.10E-01 | 0.17 | 0.305 | 1 | Macrophage |
| AP2S1 | 5.18E-05 | 1.31E+00 | 0.319 | 0.263 | 1 | Macrophage |
| SEC61G | 5.34E-05 | 5.89E-01 | 0.286 | 0.51 | 1 | Macrophage |
| OSTC | 5.50E-05 | 3.23E-01 | 0.156 | 0.28 | 1 | Macrophage |
| H2AFJ | 5.57E-05 | 3.45E-01 | 0.236 | 0.398 | 1 | Macrophage |
| GLIPR1 | 5.60E-05 | 2.70E-01 | 0.167 | 0.298 | 1 | Macrophage |
| NDUFA1 | 5.72E-05 | 5.46E-01 | 0.322 | 0.563 | 1 | Macrophage |
| ELOB | 5.77E-05 | 4.83E-01 | 0.373 | 0.646 | 1 | Macrophage |
| COX5A | 5.96E-05 | 5.70E-01 | 0.257 | 0.453 | 1 | Macrophage |
| DYNLRB1 | 6.02E-05 | 2.76E-01 | 0.185 | 0.325 | 1 | Macrophage |
| UQCR10 | 6.13E-05 | 1.46E+00 | 0.428 | 0.418 | 1 | Macrophage |
| BAG1 | 6.30E-05 | 2.68E-01 | 0.145 | 0.266 | 1 | Macrophage |
| NDUFB2 | 6.33E-05 | 4.19E-01 | 0.344 | 0.6 | 1 | Macrophage |
| DDT | 6.90E-05 | 4.25E-01 | 0.236 | 0.398 | 1 | Macrophage |
| MT-ND4L | 7.48E-05 | 4.69E-01 | 0.174 | 0.305 | 1 | Macrophage |
| PRNP | 8.13E-05 | 3.12E-01 | 0.17 | 0.305 | 1 | Macrophage |
| GMFG | 8.39E-05 | 5.31E-01 | 0.355 | 0.598 | 1 | Macrophage |
| MTPN | 8.89E-05 | 2.83E-01 | 0.141 | 0.256 | 1 | Macrophage |
| ATP5MPL | 9.53E-05 | 5.22E-01 | 0.25 | 0.439 | 1 | Macrophage |
| SCP2 | 9.77E-05 | 4.62E-01 | 0.199 | 0.338 | 1 | Macrophage |
| DBI | 0.000106947 | 3.58E-01 | 0.37 | 0.657 | 1 | Macrophage |
| IL10RA | 0.000107442 | 3.31E-01 | 0.152 | 0.274 | 1 | Macrophage |
| SELENOH | 0.000109468 | 3.60E-01 | 0.17 | 0.3 | 1 | Macrophage |
| UBE2L6 | 0.000115888 | 5.42E-01 | 0.203 | 0.357 | 1 | Macrophage |
| RTN4 | 0.000121215 | 5.84E-01 | 0.246 | 0.425 | 1 | Macrophage |
| MIEN1 | 0.000130368 | 4.19E-01 | 0.134 | 0.251 | 1 | Macrophage |
| ZNF706 | 0.000132064 | 3.00E-01 | 0.196 | 0.325 | 1 | Macrophage |
| NUCB1 | 0.00015193 | 3.90E-01 | 0.163 | 0.282 | 1 | Macrophage |
| EIF3D | 0.000152902 | 4.08E-01 | 0.196 | 0.334 | 1 | Macrophage |
| NDUFB4 | 0.000157534 | 4.60E-01 | 0.228 | 0.38 | 1 | Macrophage |
| NDUFC2 | 0.000170403 | 5.95E-01 | 0.196 | 0.334 | 1 | Macrophage |
| UBL5 | 0.000177851 | 5.09E-01 | 0.388 | 0.667 | 1 | Macrophage |
| COX17 | 0.000230528 | 4.42E-01 | 0.163 | 0.286 | 1 | Macrophage |
| RBX1 | 0.000247141 | 1.37E+00 | 0.304 | 0.251 | 1 | Macrophage |
| TPT1 | 0.0002636 | 3.09E-01 | 0.967 | 0.996 | 1 | Macrophage |
| FUNDC2 | 0.000310372 | 3.13E-01 | 0.159 | 0.273 | 1 | Macrophage |
| PSMA4 | 0.000314707 | 3.92E-01 | 0.188 | 0.312 | 1 | Macrophage |
| ISCU | 0.000333406 | 4.44E-01 | 0.181 | 0.302 | 1 | Macrophage |
| PSMB2 | 0.000352122 | 5.09E-01 | 0.17 | 0.284 | 1 | Macrophage |
| RAB5C | 0.000362298 | 4.42E-01 | 0.188 | 0.318 | 1 | Macrophage |
| PKM | 0.000381647 | 3.87E-01 | 0.192 | 0.323 | 1 | Macrophage |
| CAPZA1 | 0.000390936 | 3.90E-01 | 0.159 | 0.275 | 1 | Macrophage |
| DPP7 | 0.00045319 | 5.90E-01 | 0.207 | 0.344 | 1 | Macrophage |
| PSMA3 | 0.000492602 | 5.41E-01 | 0.141 | 0.25 | 1 | Macrophage |
| ATP6AP2 | 0.000528483 | 6.64E-01 | 0.199 | 0.329 | 1 | Macrophage |
| TNFSF10 | 0.000589499 | 1.52E+00 | 0.25 | 0.201 | 1 | Macrophage |
| ZNHIT1 | 0.000623248 | 4.51E-01 | 0.156 | 0.27 | 1 | Macrophage |
| YWHAE | 0.000742112 | 5.97E-01 | 0.181 | 0.305 | 1 | Macrophage |
| COX5B | 0.000749101 | 6.35E-01 | 0.333 | 0.566 | 1 | Macrophage |
| ERP44 | 0.000850077 | 4.35E-01 | 0.167 | 0.267 | 1 | Macrophage |
| ATP5PB | 0.000868787 | 2.81E-01 | 0.185 | 0.301 | 1 | Macrophage |
| MYDGF | 0.0008952 | 6.22E-01 | 0.236 | 0.379 | 1 | Macrophage |
| BCAP31 | 0.000898804 | 5.66E-01 | 0.214 | 0.356 | 1 | Macrophage |
| SMIM26 | 0.001080926 | 2.97E-01 | 0.174 | 0.277 | 1 | Macrophage |
| HCLS1 | 0.001098194 | 6.30E-01 | 0.217 | 0.35 | 1 | Macrophage |
| PLIN2 | 0.001183751 | 4.00E-01 | 0.246 | 0.408 | 1 | Macrophage |
| NDFIP1 | 0.001197536 | 4.08E-01 | 0.199 | 0.32 | 1 | Macrophage |
| PDIA6 | 0.001238281 | 5.49E-01 | 0.221 | 0.356 | 1 | Macrophage |
| OCIAD1 | 0.001239552 | 4.63E-01 | 0.156 | 0.254 | 1 | Macrophage |
| GNG5 | 0.001380081 | 1.43E+00 | 0.413 | 0.444 | 1 | Macrophage |
| SNX6 | 0.001808435 | 1.31E+00 | 0.297 | 0.263 | 1 | Macrophage |
| MRPL54 | 0.001921462 | 5.63E-01 | 0.217 | 0.332 | 1 | Macrophage |
| EVI2B | 0.001926749 | 4.05E-01 | 0.163 | 0.268 | 1 | Macrophage |
| ERP29 | 0.002032349 | 7.74E-01 | 0.264 | 0.432 | 1 | Macrophage |
| COX6C | 0.00211237 | 5.17E-01 | 0.409 | 0.68 | 1 | Macrophage |
| ATP5ME | 0.002124261 | 6.21E-01 | 0.268 | 0.428 | 1 | Macrophage |
| MRPL51 | 0.002298871 | 5.62E-01 | 0.156 | 0.257 | 1 | Macrophage |
| NDUFV2 | 0.002337541 | 4.47E-01 | 0.188 | 0.292 | 1 | Macrophage |
| HSP90B1 | 0.002451527 | 5.26E-01 | 0.391 | 0.626 | 1 | Macrophage |
| SEM1 | 0.00261558 | 5.16E-01 | 0.217 | 0.353 | 1 | Macrophage |
| RAB7A | 0.002906397 | 4.34E-01 | 0.217 | 0.349 | 1 | Macrophage |
| UQCR11 | 0.003330532 | 6.21E-01 | 0.355 | 0.583 | 1 | Macrophage |
| LAPTM5 | 0.003518758 | 5.10E-01 | 0.409 | 0.713 | 1 | Macrophage |
| REEP5 | 0.003892704 | 5.73E-01 | 0.221 | 0.336 | 1 | Macrophage |
| VPS29 | 0.004687102 | 6.32E-01 | 0.181 | 0.283 | 1 | Macrophage |
| ABRACL | 0.005824162 | 6.80E-01 | 0.236 | 0.368 | 1 | Macrophage |
| MT-ND3 | 0.006278068 | 4.98E-01 | 0.775 | 0.965 | 1 | Macrophage |
| NDUFS7 | 0.006883612 | 4.03E-01 | 0.174 | 0.262 | 1 | Macrophage |
| NAMPT | 0.007138638 | 5.06E-01 | 0.196 | 0.314 | 1 | Macrophage |
| VIM | 0.007665286 | 3.95E-01 | 0.721 | 0.849 | 1 | Macrophage |
| TUBA1B | 0.007779161 | 6.24E-01 | 0.409 | 0.693 | 1 | Macrophage |
| VMP1 | 0.007798668 | 5.34E-01 | 0.178 | 0.263 | 1 | Macrophage |
| SSBP1 | 0.007840976 | 5.97E-01 | 0.167 | 0.265 | 1 | Macrophage |
| RHOA | 0.009236032 | 5.59E-01 | 0.406 | 0.694 | 1 | Macrophage |
| CD79A | 0 | 4.13E+00 | 0.961 | 0.021 | 0 | B_cell |
| IGHM | 0 | 3.43E+00 | 0.836 | 0.023 | 0 | B_cell |
| HLA-DQA1 | 0 | 2.86E+00 | 0.895 | 0.09 | 0 | B_cell |
| MS4A1 | 0 | 2.65E+00 | 0.797 | 0.028 | 0 | B_cell |
| IGHD | 0 | 2.50E+00 | 0.621 | 0.001 | 0 | B_cell |
| PHACTR1 | 0 | 2.04E+00 | 0.547 | 0.02 | 0 | B_cell |
| TNFRSF13C | 0 | 1.90E+00 | 0.594 | 0.008 | 0 | B_cell |
| BANK1 | 0 | 1.87E+00 | 0.59 | 0.012 | 0 | B_cell |
| LINC00926 | 0 | 1.70E+00 | 0.492 | 0.003 | 0 | B_cell |
| CD24 | 0 | 1.37E+00 | 0.402 | 0.006 | 0 | B_cell |
| FCER2 | 0 | 1.34E+00 | 0.363 | 0.001 | 0 | B_cell |
| SPIB | 0 | 1.27E+00 | 0.375 | 0.006 | 0 | B_cell |
| CD22 | 0 | 1.19E+00 | 0.379 | 0.003 | 0 | B_cell |
| PKIG | 0 | 1.18E+00 | 0.379 | 0.018 | 0 | B_cell |
| VPREB3 | 0 | 1.11E+00 | 0.301 | 0.001 | 0 | B_cell |
| GNG7 | 0 | 1.08E+00 | 0.332 | 0.009 | 0 | B_cell |
| BCL11A | 0 | 1.06E+00 | 0.293 | 0.006 | 0 | B_cell |
| FCRL1 | 0 | 1.06E+00 | 0.301 | 0.001 | 0 | B_cell |
| LINC01857 | 0 | 9.33E-01 | 0.277 | 0.001 | 0 | B_cell |
| HLA-DQB1 | 1.93E-300 | 2.65E+00 | 0.887 | 0.136 | 6.47E-296 | B_cell |
| AFF3 | 2.97E-266 | 9.96E-01 | 0.293 | 0.014 | 9.96E-262 | B_cell |
| RALGPS2 | 1.24E-250 | 1.11E+00 | 0.309 | 0.017 | 4.17E-246 | B_cell |
| LY86 | 1.43E-242 | 8.75E-01 | 0.301 | 0.016 | 4.81E-238 | B_cell |
| TCF4 | 3.30E-242 | 1.26E+00 | 0.43 | 0.034 | 1.11E-237 | B_cell |
| HLA-DRA | 2.71E-236 | 3.53E+00 | 0.996 | 0.285 | 9.10E-232 | B_cell |
| LY9 | 1.41E-231 | 2.31E+00 | 0.617 | 0.079 | 4.74E-227 | B_cell |
| CCR7 | 2.62E-227 | 1.49E+00 | 0.445 | 0.039 | 8.79E-223 | B_cell |
| YBX3 | 5.67E-208 | 2.50E+00 | 0.664 | 0.101 | 1.90E-203 | B_cell |
| MEF2C | 3.27E-195 | 1.12E+00 | 0.371 | 0.031 | 1.10E-190 | B_cell |
| HLA-DMA | 3.01E-184 | 1.70E+00 | 0.746 | 0.14 | 1.01E-179 | B_cell |
| HLA-DRB1 | 2.88E-169 | 2.90E+00 | 0.996 | 0.45 | 9.66E-165 | B_cell |
| CD40 | 9.95E-165 | 8.76E-01 | 0.254 | 0.017 | 3.34E-160 | B_cell |
| HLA-DPA1 | 1.57E-161 | 2.07E+00 | 0.98 | 0.407 | 5.27E-157 | B_cell |
| HLA-DMB | 2.91E-148 | 1.25E+00 | 0.43 | 0.054 | 9.75E-144 | B_cell |
| HLA-DPB1 | 2.51E-145 | 2.06E+00 | 0.957 | 0.402 | 8.41E-141 | B_cell |
| CD74 | 1.26E-144 | 2.67E+00 | 1 | 0.772 | 4.22E-140 | B_cell |
| TSPAN13 | 7.40E-142 | 7.78E-01 | 0.266 | 0.022 | 2.48E-137 | B_cell |
| CD37 | 2.68E-111 | 1.78E+00 | 0.973 | 0.69 | 9.00E-107 | B_cell |
| KDM4B | 1.44E-107 | 9.90E-01 | 0.367 | 0.053 | 4.84E-103 | B_cell |
| ADAM28 | 9.89E-103 | 9.80E-01 | 0.352 | 0.05 | 3.32E-98 | B_cell |
| BASP1 | 1.12E-99 | 1.17E+00 | 0.398 | 0.065 | 3.76E-95 | B_cell |
| RPS8 | 2.45E-98 | 1.02E+00 | 1 | 0.986 | 8.21E-94 | B_cell |
| RPS23 | 4.63E-97 | 9.50E-01 | 1 | 0.991 | 1.55E-92 | B_cell |
| LAPTM5 | 2.51E-93 | 1.81E+00 | 0.961 | 0.707 | 8.42E-89 | B_cell |
| STAG3 | 2.87E-90 | 1.47E+00 | 0.305 | 0.044 | 9.62E-86 | B_cell |
| POU2F2 | 1.29E-87 | 9.49E-01 | 0.348 | 0.056 | 4.33E-83 | B_cell |
| RPL39 | 2.30E-79 | 7.40E-01 | 1 | 0.99 | 7.72E-75 | B_cell |
| RPL32 | 3.79E-78 | 7.95E-01 | 1 | 0.993 | 1.27E-73 | B_cell |
| RPL18A | 1.30E-76 | 9.95E-01 | 0.996 | 0.985 | 4.37E-72 | B_cell |
| CTSH | 3.08E-76 | 7.70E-01 | 0.297 | 0.047 | 1.03E-71 | B_cell |
| CD83 | 1.29E-75 | 1.72E+00 | 0.848 | 0.399 | 4.34E-71 | B_cell |
| RPL8 | 2.48E-75 | 8.38E-01 | 1 | 0.982 | 8.31E-71 | B_cell |
| RPL11 | 1.94E-74 | 7.16E-01 | 1 | 0.991 | 6.50E-70 | B_cell |
| SWAP70 | 3.32E-70 | 8.05E-01 | 0.277 | 0.045 | 1.11E-65 | B_cell |
| RPS12 | 2.96E-67 | 6.99E-01 | 1 | 0.994 | 9.94E-63 | B_cell |
| SMIM14 | 4.36E-67 | 9.88E-01 | 0.348 | 0.07 | 1.46E-62 | B_cell |
| CD55 | 3.00E-66 | 1.38E+00 | 0.832 | 0.396 | 1.01E-61 | B_cell |
| RPL12 | 1.18E-65 | 7.75E-01 | 0.996 | 0.985 | 3.95E-61 | B_cell |
| RPL18 | 8.65E-64 | 7.11E-01 | 1 | 0.983 | 2.90E-59 | B_cell |
| RPS11 | 8.04E-61 | 8.80E-01 | 1 | 0.942 | 2.70E-56 | B_cell |
| RPL34 | 1.04E-60 | 7.30E-01 | 1 | 0.991 | 3.49E-56 | B_cell |
| RPS5 | 3.27E-60 | 8.36E-01 | 0.992 | 0.961 | 1.10E-55 | B_cell |
| RPL30 | 7.75E-60 | 6.19E-01 | 1 | 0.991 | 2.60E-55 | B_cell |
| CD79B | 4.94E-59 | 1.10E+00 | 0.441 | 0.12 | 1.66E-54 | B_cell |
| TCOF1 | 7.27E-58 | 1.02E+00 | 0.375 | 0.092 | 2.44E-53 | B_cell |
| RPL13 | 8.46E-57 | 6.44E-01 | 1 | 0.994 | 2.84E-52 | B_cell |
| RPL19 | 2.62E-55 | 6.05E-01 | 1 | 0.993 | 8.80E-51 | B_cell |
| IFT57 | 5.10E-54 | 9.67E-01 | 0.395 | 0.103 | 1.71E-49 | B_cell |
| CD52 | 6.58E-54 | 1.04E+00 | 0.926 | 0.52 | 2.21E-49 | B_cell |
| SNX2 | 3.05E-53 | 8.19E-01 | 0.371 | 0.094 | 1.02E-48 | B_cell |
| RPS3A | 3.58E-53 | 5.82E-01 | 0.996 | 0.989 | 1.20E-48 | B_cell |
| RPS13 | 9.76E-52 | 6.48E-01 | 0.996 | 0.984 | 3.27E-47 | B_cell |
| RPS4X | 6.10E-50 | 5.95E-01 | 1 | 0.989 | 2.05E-45 | B_cell |
| RPL29 | 1.60E-48 | 6.08E-01 | 1 | 0.986 | 5.36E-44 | B_cell |
| RPS25 | 2.37E-48 | 6.80E-01 | 0.992 | 0.982 | 7.94E-44 | B_cell |
| TMEM123 | 2.53E-48 | 1.32E+00 | 0.629 | 0.275 | 8.50E-44 | B_cell |
| RPS21 | 1.57E-47 | 6.50E-01 | 0.996 | 0.961 | 5.25E-43 | B_cell |
| RNASET2 | 2.69E-47 | 9.36E-01 | 0.652 | 0.278 | 9.02E-43 | B_cell |
| ARID5B | 9.20E-47 | 9.61E-01 | 0.602 | 0.235 | 3.08E-42 | B_cell |
| RPS2 | 1.16E-46 | 6.52E-01 | 1 | 0.988 | 3.91E-42 | B_cell |
| RPS15 | 7.78E-46 | 5.34E-01 | 0.996 | 0.991 | 2.61E-41 | B_cell |
| LTB | 4.66E-44 | 8.49E-01 | 0.684 | 0.298 | 1.56E-39 | B_cell |
| ORAI2 | 9.40E-44 | 7.58E-01 | 0.297 | 0.073 | 3.15E-39 | B_cell |
| RPL7A | 3.48E-43 | 5.67E-01 | 1 | 0.982 | 1.17E-38 | B_cell |
| RPS28 | 1.04E-41 | 5.40E-01 | 1 | 0.989 | 3.47E-37 | B_cell |
| RPL26 | 7.01E-41 | 5.64E-01 | 1 | 0.986 | 2.35E-36 | B_cell |
| RPL22 | 8.30E-41 | 6.50E-01 | 0.992 | 0.938 | 2.78E-36 | B_cell |
| RPL37 | 4.83E-39 | 4.95E-01 | 1 | 0.985 | 1.62E-34 | B_cell |
| RPL9 | 6.60E-39 | 5.89E-01 | 1 | 0.98 | 2.21E-34 | B_cell |
| RPS16 | 2.14E-37 | 5.42E-01 | 1 | 0.976 | 7.18E-33 | B_cell |
| RPL28 | 2.78E-37 | 4.62E-01 | 1 | 0.994 | 9.34E-33 | B_cell |
| RPL10A | 4.35E-37 | 6.09E-01 | 1 | 0.967 | 1.46E-32 | B_cell |
| EZR | 5.89E-37 | 1.02E+00 | 0.887 | 0.673 | 1.97E-32 | B_cell |
| RPSA | 6.03E-37 | 6.44E-01 | 0.988 | 0.959 | 2.02E-32 | B_cell |
| RPS18 | 7.16E-37 | 6.75E-01 | 1 | 0.989 | 2.40E-32 | B_cell |
| EEF1B2 | 7.17E-37 | 7.15E-01 | 0.977 | 0.9 | 2.40E-32 | B_cell |
| RACK1 | 7.92E-37 | 5.88E-01 | 0.996 | 0.957 | 2.66E-32 | B_cell |
| ZFAS1 | 2.00E-36 | 8.59E-01 | 0.906 | 0.663 | 6.72E-32 | B_cell |
| NCF1 | 2.74E-36 | 7.07E-01 | 0.449 | 0.158 | 9.20E-32 | B_cell |
| FAU | 3.01E-36 | 4.65E-01 | 0.996 | 0.988 | 1.01E-31 | B_cell |
| RPS9 | 3.41E-36 | 6.65E-01 | 0.992 | 0.97 | 1.14E-31 | B_cell |
| LYN | 4.35E-35 | 8.82E-01 | 0.484 | 0.188 | 1.46E-30 | B_cell |
| ID3 | 4.73E-34 | 1.27E+00 | 0.363 | 0.121 | 1.59E-29 | B_cell |
| DRAM2 | 6.11E-34 | 7.11E-01 | 0.363 | 0.119 | 2.05E-29 | B_cell |
| RPLP0 | 1.05E-33 | 6.49E-01 | 0.992 | 0.917 | 3.53E-29 | B_cell |
| RPS19 | 2.91E-33 | 5.29E-01 | 0.996 | 0.992 | 9.77E-29 | B_cell |
| SMCHD1 | 3.24E-33 | 8.51E-01 | 0.719 | 0.395 | 1.09E-28 | B_cell |
| NFKBID | 6.55E-32 | 8.97E-01 | 0.527 | 0.233 | 2.20E-27 | B_cell |
| P2RX5 | 1.37E-31 | 7.68E-01 | 0.332 | 0.109 | 4.60E-27 | B_cell |
| BIRC3 | 1.83E-31 | 9.31E-01 | 0.66 | 0.342 | 6.13E-27 | B_cell |
| SYNGR2 | 2.31E-31 | 8.55E-01 | 0.418 | 0.163 | 7.74E-27 | B_cell |
| RPL35A | 2.32E-31 | 5.01E-01 | 0.992 | 0.985 | 7.78E-27 | B_cell |
| CHPT1 | 2.96E-31 | 7.32E-01 | 0.395 | 0.142 | 9.93E-27 | B_cell |
| RPL21 | 4.24E-31 | 6.71E-01 | 0.996 | 0.988 | 1.42E-26 | B_cell |
| RPL23 | 4.47E-31 | 6.58E-01 | 0.98 | 0.865 | 1.50E-26 | B_cell |
| RPL5 | 8.85E-31 | 5.25E-01 | 0.992 | 0.969 | 2.97E-26 | B_cell |
| RPS6 | 2.51E-30 | 6.31E-01 | 0.992 | 0.98 | 8.41E-26 | B_cell |
| RPL36 | 4.01E-30 | 5.37E-01 | 0.988 | 0.965 | 1.35E-25 | B_cell |
| SNX9 | 5.64E-30 | 7.41E-01 | 0.324 | 0.107 | 1.89E-25 | B_cell |
| PMEPA1 | 5.70E-30 | 8.07E-01 | 0.305 | 0.096 | 1.91E-25 | B_cell |
| CD72 | 3.56E-29 | 7.80E-01 | 0.352 | 0.126 | 1.19E-24 | B_cell |
| RPS27A | 3.67E-28 | 3.99E-01 | 1 | 0.993 | 1.23E-23 | B_cell |
| RPL13A | 6.40E-28 | 7.45E-01 | 0.996 | 0.977 | 2.15E-23 | B_cell |
| RPS27 | 6.97E-28 | 5.81E-01 | 1 | 0.992 | 2.34E-23 | B_cell |
| FOXP1 | 1.36E-27 | 9.09E-01 | 0.535 | 0.263 | 4.57E-23 | B_cell |
| RPL38 | 3.77E-26 | 5.94E-01 | 0.996 | 0.913 | 1.26E-21 | B_cell |
| CDKN1A | 6.15E-26 | 7.54E-01 | 0.453 | 0.199 | 2.06E-21 | B_cell |
| SELL | 1.45E-25 | 0.86053965 | 0.355 | 0.134 | 4.85E-21 | B_cell |
| MAP4K4 | 2.24E-25 | 0.58526982 | 0.266 | 0.085 | 7.51E-21 | B_cell |
| GGA2 | 2.97E-25 | 0.53662208 | 0.316 | 0.11 | 9.96E-21 | B_cell |
| TOR3A | 4.38E-25 | 0.68417236 | 0.262 | 0.085 | 1.47E-20 | B_cell |
| TMEM156 | 7.79E-25 | 0.77591106 | 0.309 | 0.112 | 2.61E-20 | B_cell |
| EEF1A1 | 1.64E-24 | 0.35329629 | 1 | 0.995 | 5.51E-20 | B_cell |
| ARRDC2 | 2.26E-24 | 0.82679113 | 0.445 | 0.202 | 7.58E-20 | B_cell |
| RPL10 | 2.45E-24 | 0.35323743 | 1 | 0.995 | 8.22E-20 | B_cell |
| RPL41 | 3.36E-24 | 0.41004688 | 0.996 | 0.997 | 1.13E-19 | B_cell |
| TMEM243 | 4.26E-24 | 0.72621557 | 0.527 | 0.262 | 1.43E-19 | B_cell |
| RPS7 | 4.50E-24 | 0.38392297 | 1 | 0.985 | 1.51E-19 | B_cell |
| FCMR | 1.05E-23 | 0.68949834 | 0.41 | 0.174 | 3.53E-19 | B_cell |
| CLEC2D | 1.21E-23 | 0.62029653 | 0.438 | 0.19 | 4.06E-19 | B_cell |
| OTUD1 | 4.77E-23 | 0.65108919 | 0.258 | 0.085 | 1.60E-18 | B_cell |
| RPLP2 | 4.58E-22 | 0.54041035 | 1 | 0.985 | 1.54E-17 | B_cell |
| LSM7 | 5.85E-22 | 0.93135273 | 0.641 | 0.404 | 1.96E-17 | B_cell |
| BCL2 | 2.05E-21 | 0.76275674 | 0.383 | 0.166 | 6.88E-17 | B_cell |
| RPL23A | 1.02E-20 | 0.5238124 | 0.988 | 0.979 | 3.42E-16 | B_cell |
| NOP53 | 1.03E-20 | 0.67414659 | 0.84 | 0.666 | 3.44E-16 | B_cell |
| SH3BP5 | 1.05E-20 | 0.66636147 | 0.34 | 0.144 | 3.52E-16 | B_cell |
| PRKCB | 1.44E-20 | 0.60271632 | 0.352 | 0.146 | 4.82E-16 | B_cell |
| RASGRP2 | 2.34E-20 | 0.54285956 | 0.293 | 0.11 | 7.83E-16 | B_cell |
| RPS10 | 3.42E-20 | 0.53822431 | 0.965 | 0.907 | 1.15E-15 | B_cell |
| SNHG7 | 5.23E-20 | 0.6714012 | 0.633 | 0.386 | 1.75E-15 | B_cell |
| IRF9 | 1.50E-19 | 0.7337712 | 0.469 | 0.239 | 5.04E-15 | B_cell |
| PRDM2 | 3.32E-19 | 0.61105178 | 0.453 | 0.225 | 1.11E-14 | B_cell |
| RPL7 | 3.55E-19 | 0.58284003 | 0.996 | 0.925 | 1.19E-14 | B_cell |
| MT-ND3 | 4.33E-19 | 0.41348124 | 0.984 | 0.963 | 1.45E-14 | B_cell |
| RCN2 | 4.63E-19 | 0.68828772 | 0.426 | 0.211 | 1.55E-14 | B_cell |
| TPD52 | 8.62E-19 | 0.50978199 | 0.289 | 0.114 | 2.89E-14 | B_cell |
| RPL4 | 1.18E-18 | 0.54595227 | 0.98 | 0.874 | 3.95E-14 | B_cell |
| RPS15A | 1.28E-18 | 0.30919494 | 0.996 | 0.991 | 4.31E-14 | B_cell |
| SP110 | 2.26E-18 | 0.62047601 | 0.441 | 0.218 | 7.56E-14 | B_cell |
| RPS20 | 3.10E-18 | 0.57319069 | 0.988 | 0.928 | 1.04E-13 | B_cell |
| RPL15 | 9.75E-18 | 0.42221053 | 1 | 0.984 | 3.27E-13 | B_cell |
| RPL36A | 9.87E-18 | 0.60053639 | 0.859 | 0.689 | 3.31E-13 | B_cell |
| EIF3E | 1.39E-17 | 0.52698339 | 0.781 | 0.559 | 4.65E-13 | B_cell |
| NACA | 2.06E-17 | 0.40099584 | 0.98 | 0.963 | 6.92E-13 | B_cell |
| PTPN1 | 5.65E-17 | 0.60021341 | 0.434 | 0.223 | 1.89E-12 | B_cell |
| TAF1D | 5.77E-17 | 0.65600576 | 0.637 | 0.425 | 1.94E-12 | B_cell |
| RPL14 | 8.59E-17 | 0.34246451 | 0.996 | 0.982 | 2.88E-12 | B_cell |
| ZNF791 | 1.15E-16 | 0.61904689 | 0.316 | 0.142 | 3.86E-12 | B_cell |
| ODC1 | 1.51E-16 | 0.56254007 | 0.543 | 0.319 | 5.06E-12 | B_cell |
| DDX21 | 1.85E-16 | 0.71811765 | 0.562 | 0.35 | 6.21E-12 | B_cell |
| UBA52 | 2.67E-16 | 0.3446231 | 0.992 | 0.969 | 8.94E-12 | B_cell |
| RCSD1 | 4.58E-16 | 0.53960744 | 0.309 | 0.137 | 1.54E-11 | B_cell |
| TRIM38 | 9.11E-16 | 0.52367513 | 0.316 | 0.141 | 3.05E-11 | B_cell |
| RPL3 | 9.51E-16 | 0.43382305 | 1 | 0.981 | 3.19E-11 | B_cell |
| RPL24 | 1.90E-15 | 0.32174569 | 0.992 | 0.973 | 6.36E-11 | B_cell |
| PARP14 | 2.04E-15 | 0.54629663 | 0.301 | 0.133 | 6.83E-11 | B_cell |
| MT-ND4 | 2.20E-15 | 0.30909348 | 0.992 | 0.983 | 7.36E-11 | B_cell |
| EBLN3P | 3.30E-15 | 0.46401891 | 0.25 | 0.101 | 1.11E-10 | B_cell |
| SNX3 | 5.02E-15 | 0.5796202 | 0.602 | 0.39 | 1.68E-10 | B_cell |
| EEF2 | 9.81E-15 | 0.52690956 | 0.883 | 0.791 | 3.29E-10 | B_cell |
| NGLY1 | 1.22E-14 | 0.51760995 | 0.289 | 0.13 | 4.09E-10 | B_cell |
| AL121944.1 | 2.07E-14 | 0.54378017 | 0.328 | 0.156 | 6.94E-10 | B_cell |
| OSER1 | 3.01E-14 | 0.55694248 | 0.363 | 0.182 | 1.01E-09 | B_cell |
| HERPUD1 | 3.55E-14 | 0.66602383 | 0.781 | 0.645 | 1.19E-09 | B_cell |
| EIF1B | 4.16E-14 | 0.55886482 | 0.527 | 0.322 | 1.40E-09 | B_cell |
| IRF8 | 8.51E-14 | 0.47615947 | 0.609 | 0.376 | 2.85E-09 | B_cell |
| RPS14 | 1.48E-13 | 0.29763299 | 0.996 | 0.99 | 4.96E-09 | B_cell |
| GABPB1-AS1 | 1.86E-13 | 0.58723568 | 0.332 | 0.166 | 6.22E-09 | B_cell |
| RPL37A | 2.15E-13 | 0.34369325 | 0.992 | 0.967 | 7.22E-09 | B_cell |
| WDR74 | 2.63E-13 | 0.57393537 | 0.371 | 0.197 | 8.82E-09 | B_cell |
| RPL35 | 2.66E-13 | 0.4206011 | 0.977 | 0.949 | 8.91E-09 | B_cell |
| ZNRD1 | 7.24E-13 | 1.01567672 | 0.348 | 0.187 | 2.43E-08 | B_cell |
| SNHG8 | 3.19E-12 | 0.58943329 | 0.707 | 0.534 | 1.07E-07 | B_cell |
| PARP1 | 4.72E-12 | 0.39304995 | 0.312 | 0.156 | 1.58E-07 | B_cell |
| USP12 | 1.36E-11 | 0.4458184 | 0.301 | 0.151 | 4.57E-07 | B_cell |
| ATF7IP | 2.59E-11 | 0.55281043 | 0.32 | 0.17 | 8.68E-07 | B_cell |
| PPM1K | 2.99E-11 | 0.43433532 | 0.301 | 0.151 | 1.00E-06 | B_cell |
| CMTM6 | 6.59E-11 | 0.45805951 | 0.512 | 0.329 | 2.21E-06 | B_cell |
| SMAP2 | 8.14E-11 | 0.54118704 | 0.672 | 0.51 | 2.73E-06 | B_cell |
| RPS29 | 1.28E-10 | 0.40819233 | 0.996 | 0.975 | 4.29E-06 | B_cell |
| FAM3C | 1.34E-10 | 0.51903872 | 0.336 | 0.184 | 4.51E-06 | B_cell |
| KLF2 | 1.54E-10 | 0.55197036 | 0.852 | 0.659 | 5.17E-06 | B_cell |
| PRDX1 | 1.72E-10 | 0.49414201 | 0.523 | 0.338 | 5.76E-06 | B_cell |
| SP140 | 1.76E-10 | 0.38457288 | 0.305 | 0.157 | 5.89E-06 | B_cell |
| SLC25A6 | 1.88E-10 | 0.44160345 | 0.859 | 0.744 | 6.31E-06 | B_cell |
| SAT1 | 3.23E-10 | 0.37477334 | 0.902 | 0.836 | 1.08E-05 | B_cell |
| RNMT | 5.28E-10 | 0.52255521 | 0.512 | 0.347 | 1.77E-05 | B_cell |
| MTPN | 8.71E-10 | 0.40575507 | 0.418 | 0.253 | 2.92E-05 | B_cell |
| ERP29 | 1.73E-09 | 0.43747331 | 0.59 | 0.428 | 5.80E-05 | B_cell |
| SNU13 | 2.11E-09 | 0.44227422 | 0.645 | 0.462 | 7.07E-05 | B_cell |
| GPR183 | 5.00E-09 | 0.30409838 | 0.277 | 0.146 | 0.00016762 | B_cell |
| HINT1 | 5.29E-09 | 0.31450614 | 0.875 | 0.772 | 0.000177481 | B_cell |
| APLP2 | 6.74E-09 | 0.40459167 | 0.34 | 0.199 | 0.000226194 | B_cell |
| NAP1L1 | 8.49E-09 | 0.31370038 | 0.754 | 0.561 | 0.000284765 | B_cell |
| EIF2S3 | 9.21E-09 | 0.41735877 | 0.367 | 0.223 | 0.000308771 | B_cell |
| PFDN5 | 9.47E-09 | 0.28962611 | 0.961 | 0.913 | 0.000317613 | B_cell |
| MARCKSL1 | 1.05E-08 | 0.49149327 | 0.559 | 0.387 | 0.000351223 | B_cell |
| RILPL2 | 1.36E-08 | 0.4214153 | 0.453 | 0.29 | 0.00045542 | B_cell |
| MYCBP2 | 1.46E-08 | 0.40726534 | 0.398 | 0.244 | 0.00048849 | B_cell |
| SGK1 | 1.49E-08 | 0.46858787 | 0.273 | 0.15 | 0.000499177 | B_cell |
| SEC62 | 1.64E-08 | 0.39093103 | 0.551 | 0.393 | 0.000550156 | B_cell |
| HSPA4 | 1.76E-08 | 0.60795317 | 0.355 | 0.222 | 0.000590228 | B_cell |
| SMDT1 | 1.88E-08 | 0.37549648 | 0.605 | 0.438 | 0.000630028 | B_cell |
| SYPL1 | 2.61E-08 | 0.40755121 | 0.285 | 0.16 | 0.000875963 | B_cell |
| ATP2B1 | 2.69E-08 | 0.34153309 | 0.371 | 0.225 | 0.000901244 | B_cell |
| TUBA1A | 3.03E-08 | 0.41138449 | 0.699 | 0.549 | 0.001017634 | B_cell |
| NSD3 | 3.67E-08 | 0.33046865 | 0.457 | 0.299 | 0.001231384 | B_cell |
| UPF2 | 5.77E-08 | 0.34361321 | 0.254 | 0.137 | 0.001935794 | B_cell |
| ARID1B | 5.90E-08 | 0.42143212 | 0.391 | 0.25 | 0.001978291 | B_cell |
| MDM4 | 6.87E-08 | 0.3516139 | 0.309 | 0.177 | 0.002304999 | B_cell |
| RASGEF1B | 9.32E-08 | 0.43141636 | 0.344 | 0.212 | 0.003125461 | B_cell |
| NR4A1 | 1.22E-07 | 0.4664435 | 0.535 | 0.375 | 0.00408681 | B_cell |
| SF1 | 1.48E-07 | 0.39465681 | 0.68 | 0.546 | 0.004970986 | B_cell |
| CREBRF | 1.58E-07 | 0.38412841 | 0.348 | 0.216 | 0.005310163 | B_cell |
| HMGA1 | 1.83E-07 | 0.33019865 | 0.328 | 0.196 | 0.00614136 | B_cell |
| RPL27A | 1.98E-07 | 0.35119143 | 0.973 | 0.89 | 0.006645777 | B_cell |
| ALG13 | 1.99E-07 | 0.62871101 | 0.34 | 0.217 | 0.006675195 | B_cell |
| CCNI | 2.54E-07 | 0.3510291 | 0.84 | 0.707 | 0.008511655 | B_cell |
| CTSZ | 2.95E-07 | 0.29452532 | 0.262 | 0.15 | 0.009895816 | B_cell |
| USP8 | 3.21E-07 | 0.34345044 | 0.262 | 0.149 | 0.010763421 | B_cell |
| C6orf48 | 3.64E-07 | 0.37047181 | 0.527 | 0.368 | 0.01219759 | B_cell |
| CHCHD10 | 3.78E-07 | 0.39775329 | 0.281 | 0.167 | 0.012690293 | B_cell |
| MKNK2 | 4.01E-07 | 0.31349345 | 0.465 | 0.321 | 0.013449777 | B_cell |
| CD69 | 4.44E-07 | 0.26708754 | 0.934 | 0.858 | 0.014896273 | B_cell |
| ANKRD11 | 4.63E-07 | 0.49906814 | 0.457 | 0.325 | 0.015539145 | B_cell |
| ANKRD12 | 5.05E-07 | 0.42593251 | 0.57 | 0.438 | 0.016949424 | B_cell |
| RPL27 | 5.20E-07 | 0.30170385 | 0.977 | 0.931 | 0.017434878 | B_cell |
| C7orf50 | 6.66E-07 | 0.42411569 | 0.277 | 0.168 | 0.022339157 | B_cell |
| RBM38 | 7.46E-07 | 0.59613077 | 0.539 | 0.418 | 0.025016489 | B_cell |
| TNRC6B | 9.83E-07 | 0.34644058 | 0.398 | 0.26 | 0.032969803 | B_cell |
| IL4R | 1.08E-06 | 0.48237715 | 0.25 | 0.15 | 0.036189729 | B_cell |
| IMPDH2 | 1.24E-06 | 0.29154677 | 0.254 | 0.145 | 0.041622195 | B_cell |
| ISG20 | 1.26E-06 | 0.26141653 | 0.879 | 0.738 | 0.042183958 | B_cell |
| STX5 | 1.37E-06 | 0.32033876 | 0.344 | 0.215 | 0.045870145 | B_cell |
| BOD1L1 | 1.52E-06 | 0.40323935 | 0.344 | 0.223 | 0.050873524 | B_cell |
| LIMD2 | 1.68E-06 | 0.30038142 | 0.637 | 0.482 | 0.05647343 | B_cell |
| RGS16 | 1.73E-06 | 0.49239753 | 0.273 | 0.167 | 0.058101732 | B_cell |
| SERP1 | 3.09E-06 | 0.29970509 | 0.82 | 0.693 | 0.10348026 | B_cell |
| EPB41L4A-AS1 | 3.79E-06 | 0.29797376 | 0.348 | 0.232 | 0.127026487 | B_cell |
| NSA2 | 3.97E-06 | 0.28545678 | 0.48 | 0.349 | 0.133210201 | B_cell |
| PRNP | 4.36E-06 | 0.25177302 | 0.441 | 0.302 | 0.146332508 | B_cell |
| SIAH2 | 4.88E-06 | 0.42283199 | 0.387 | 0.271 | 0.163588335 | B_cell |
| MT-ND4L | 5.40E-06 | 0.30349393 | 0.43 | 0.302 | 0.181231822 | B_cell |
| ZNF331 | 6.52E-06 | 0.53540076 | 0.68 | 0.576 | 0.21867787 | B_cell |
| LAMTOR5 | 1.40E-05 | 0.28688094 | 0.477 | 0.339 | 0.468912144 | B_cell |
| NR1H2 | 1.60E-05 | 0.29081604 | 0.344 | 0.228 | 0.535815287 | B_cell |
| UBALD2 | 1.62E-05 | 0.47194135 | 0.551 | 0.453 | 0.544650226 | B_cell |
| MAPK1IP1L | 1.68E-05 | 0.2871324 | 0.465 | 0.327 | 0.563850029 | B_cell |
| EMP3 | 1.72E-05 | 0.40201987 | 0.695 | 0.59 | 0.57758561 | B_cell |
| SLBP | 2.01E-05 | 0.37120826 | 0.422 | 0.305 | 0.675085297 | B_cell |
| ADD3 | 3.88E-05 | 0.2635606 | 0.293 | 0.192 | 1 | B_cell |
| TAF7 | 4.80E-05 | 0.2645076 | 0.617 | 0.499 | 1 | B_cell |
| TTC3 | 5.27E-05 | 0.40357156 | 0.273 | 0.181 | 1 | B_cell |
| ATP6V1F | 5.29E-05 | 0.33134991 | 0.477 | 0.375 | 1 | B_cell |
| TOMM20 | 5.81E-05 | 3.12E-01 | 0.555 | 0.426 | 1 | B_cell |
| GRASP | 6.06E-05 | 4.38E-01 | 0.438 | 0.336 | 1 | B_cell |
| JUNB | 7.18E-05 | 2.52E-01 | 0.98 | 0.921 | 1 | B_cell |
| AFF4 | 7.41E-05 | 2.51E-01 | 0.32 | 0.217 | 1 | B_cell |
| YBX1 | 8.57E-05 | 2.69E-01 | 0.891 | 0.836 | 1 | B_cell |
| SNHG15 | 9.98E-05 | 3.15E-01 | 0.438 | 0.327 | 1 | B_cell |
| EVI2B | 0.000100798 | 2.67E-01 | 0.375 | 0.266 | 1 | B_cell |
| TNFAIP8 | 0.000129111 | 3.69E-01 | 0.273 | 0.185 | 1 | B_cell |
| TCEA1 | 0.000133989 | 2.52E-01 | 0.344 | 0.245 | 1 | B_cell |
| C1QBP | 0.000159434 | 2.56E-01 | 0.32 | 0.225 | 1 | B_cell |
| PPIG | 0.000162925 | 2.75E-01 | 0.426 | 0.308 | 1 | B_cell |
| GRB2 | 0.00018677 | 2.70E-01 | 0.391 | 0.291 | 1 | B_cell |
| KLF3 | 0.000232431 | 3.62E-01 | 0.352 | 0.257 | 1 | B_cell |
| SNRPG | 0.00026909 | 2.62E-01 | 0.477 | 0.378 | 1 | B_cell |
| CHRAC1 | 0.000275109 | 2.54E-01 | 0.254 | 0.17 | 1 | B_cell |
| WDR45B | 0.000293595 | 3.63E-01 | 0.262 | 0.181 | 1 | B_cell |
| ZBTB20 | 0.000294727 | 2.77E-01 | 0.285 | 0.194 | 1 | B_cell |
| RPS17 | 0.000297212 | 4.46E-01 | 0.602 | 0.515 | 1 | B_cell |
| RAB11FIP1 | 0.000346964 | 2.95E-01 | 0.426 | 0.326 | 1 | B_cell |
| SRRM2 | 0.000356515 | 2.63E-01 | 0.66 | 0.59 | 1 | B_cell |
| RHOH | 0.000401243 | 3.08E-01 | 0.52 | 0.414 | 1 | B_cell |
| SNAP23 | 0.000428918 | 3.07E-01 | 0.25 | 0.17 | 1 | B_cell |
| CKS2 | 0.000437242 | 2.65E-01 | 0.32 | 0.225 | 1 | B_cell |
| PPP4R3A | 0.00051752 | 2.80E-01 | 0.277 | 0.192 | 1 | B_cell |
| RPL22L1 | 0.000647117 | 3.23E-01 | 0.605 | 0.519 | 1 | B_cell |
| ANKRD44 | 0.000667885 | 3.07E-01 | 0.336 | 0.247 | 1 | B_cell |
| APEX1 | 0.000687923 | 3.12E-01 | 0.254 | 0.178 | 1 | B_cell |
| JMJD1C | 0.000738193 | 2.82E-01 | 0.438 | 0.343 | 1 | B_cell |
| HIST1H1C | 0.000994664 | 5.03E-01 | 0.352 | 0.267 | 1 | B_cell |
| SMG1 | 0.001130165 | 3.53E-01 | 0.277 | 0.196 | 1 | B_cell |
| NUDT4 | 0.002523602 | 3.38E-01 | 0.25 | 0.179 | 1 | B_cell |
| IRF1 | 0.004004829 | 2.82E-01 | 0.84 | 0.824 | 1 | B_cell |
| DAZAP2 | 0.004789912 | 2.52E-01 | 0.598 | 0.512 | 1 | B_cell |
| RGS2 | 0.006283041 | 6.68E-01 | 0.547 | 0.5 | 1 | B_cell |
| APOA2 | 0 | 9.30E+00 | 1 | 0.041 | 0 | Hepatocytes |
| ALB | 0 | 9.25E+00 | 1 | 0.042 | 0 | Hepatocytes |
| APOA1 | 0 | 8.56E+00 | 1 | 0.03 | 0 | Hepatocytes |
| APOC3 | 0 | 7.12E+00 | 0.981 | 0.014 | 0 | Hepatocytes |
| APOC1 | 0 | 7.04E+00 | 1 | 0.029 | 0 | Hepatocytes |
| AMBP | 0 | 6.82E+00 | 1 | 0.006 | 0 | Hepatocytes |
| SERPINA1 | 0 | 6.72E+00 | 1 | 0.04 | 0 | Hepatocytes |
| TTR | 0 | 6.25E+00 | 0.963 | 0.006 | 0 | Hepatocytes |
| APOE | 0 | 6.10E+00 | 0.991 | 0.029 | 0 | Hepatocytes |
| FABP1 | 0 | 6.03E+00 | 0.972 | 0.004 | 0 | Hepatocytes |
| VTN | 0 | 5.93E+00 | 1 | 0.004 | 0 | Hepatocytes |
| APOH | 0 | 5.91E+00 | 1 | 0.005 | 0 | Hepatocytes |
| RBP4 | 0 | 5.91E+00 | 0.991 | 0.006 | 0 | Hepatocytes |
| ORM1 | 0 | 5.89E+00 | 0.925 | 0.004 | 0 | Hepatocytes |
| FGG | 0 | 5.53E+00 | 0.907 | 0.005 | 0 | Hepatocytes |
| FGB | 0 | 5.41E+00 | 0.916 | 0.004 | 0 | Hepatocytes |
| FGA | 0 | 5.05E+00 | 0.888 | 0.004 | 0 | Hepatocytes |
| TF | 0 | 4.96E+00 | 0.935 | 0.003 | 0 | Hepatocytes |
| RARRES2 | 0 | 4.89E+00 | 0.907 | 0.004 | 0 | Hepatocytes |
| CYP2E1 | 0 | 4.85E+00 | 0.776 | 0.006 | 0 | Hepatocytes |
| IFI27 | 0 | 4.70E+00 | 0.916 | 0.035 | 0 | Hepatocytes |
| ORM2 | 0 | 4.39E+00 | 0.888 | 0.001 | 0 | Hepatocytes |
| KNG1 | 0 | 4.02E+00 | 0.841 | 0.001 | 0 | Hepatocytes |
| CFHR1 | 0 | 4.02E+00 | 0.822 | 0.001 | 0 | Hepatocytes |
| AHSG | 0 | 3.98E+00 | 0.897 | 0.001 | 0 | Hepatocytes |
| AGT | 0 | 3.93E+00 | 0.832 | 0.002 | 0 | Hepatocytes |
| GC | 0 | 3.89E+00 | 0.841 | 0.001 | 0 | Hepatocytes |
| CLU | 0 | 3.82E+00 | 0.879 | 0.035 | 0 | Hepatocytes |
| PPP1R1A | 0 | 3.79E+00 | 0.794 | 0.001 | 0 | Hepatocytes |
| EPHX1 | 0 | 3.78E+00 | 0.822 | 0.008 | 0 | Hepatocytes |
| SELENOP | 0 | 3.67E+00 | 0.86 | 0.015 | 0 | Hepatocytes |
| GPC3 | 0 | 3.55E+00 | 0.766 | 0.001 | 0 | Hepatocytes |
| CYP2D6 | 0 | 3.53E+00 | 0.804 | 0.003 | 0 | Hepatocytes |
| TMEM176B | 0 | 3.52E+00 | 0.832 | 0.009 | 0 | Hepatocytes |
| TMEM176A | 0 | 3.51E+00 | 0.794 | 0.004 | 0 | Hepatocytes |
| ANG | 0 | 3.47E+00 | 0.813 | 0.003 | 0 | Hepatocytes |
| F2 | 0 | 3.45E+00 | 0.841 | 0.001 | 0 | Hepatocytes |
| IGF2.1 | 0 | 3.43E+00 | 0.738 | 0.001 | 0 | Hepatocytes |
| GSTA1 | 0 | 3.30E+00 | 0.458 | 0.001 | 0 | Hepatocytes |
| HPX | 0 | 3.19E+00 | 0.757 | 0.001 | 0 | Hepatocytes |
| AGXT | 0 | 3.12E+00 | 0.72 | 0.001 | 0 | Hepatocytes |
| ANGPTL8 | 0 | 3.09E+00 | 0.626 | 0 | 0 | Hepatocytes |
| F10 | 0 | 2.98E+00 | 0.72 | 0 | 0 | Hepatocytes |
| SERPINC1 | 0 | 2.93E+00 | 0.645 | 0.001 | 0 | Hepatocytes |
| FGL1 | 0 | 2.93E+00 | 0.439 | 0.003 | 0 | Hepatocytes |
| HSD17B6 | 0 | 2.91E+00 | 0.757 | 0.001 | 0 | Hepatocytes |
| ALDOB | 0 | 2.85E+00 | 0.673 | 0.001 | 0 | Hepatocytes |
| SERPINF1 | 0 | 2.79E+00 | 0.701 | 0.008 | 0 | Hepatocytes |
| AZGP1 | 0 | 2.74E+00 | 0.645 | 0.001 | 0 | Hepatocytes |
| CYP3A7 | 0 | 2.72E+00 | 0.533 | 0 | 0 | Hepatocytes |
| ANGPTL3 | 0 | 2.66E+00 | 0.607 | 0 | 0 | Hepatocytes |
| C3 | 0 | 2.62E+00 | 0.551 | 0.004 | 0 | Hepatocytes |
| ITIH2 | 0 | 2.60E+00 | 0.598 | 0.001 | 0 | Hepatocytes |
| SULT2A1 | 0 | 2.59E+00 | 0.57 | 0 | 0 | Hepatocytes |
| MGST1 | 0 | 2.58E+00 | 0.514 | 0.003 | 0 | Hepatocytes |
| KHK | 0 | 2.56E+00 | 0.654 | 0.008 | 0 | Hepatocytes |
| ASGR1 | 0 | 2.53E+00 | 0.692 | 0.005 | 0 | Hepatocytes |
| SERPINA7 | 0 | 2.50E+00 | 0.598 | 0 | 0 | Hepatocytes |
| SPP2 | 0 | 2.38E+00 | 0.57 | 0 | 0 | Hepatocytes |
| NUPR1 | 0 | 2.37E+00 | 0.533 | 0.002 | 0 | Hepatocytes |
| KRT18 | 0 | 2.35E+00 | 0.598 | 0.009 | 0 | Hepatocytes |
| SERPIND1 | 0 | 2.35E+00 | 0.579 | 0 | 0 | Hepatocytes |
| APCS | 0 | 2.35E+00 | 0.262 | 0.001 | 0 | Hepatocytes |
| PEG10 | 0 | 2.34E+00 | 0.514 | 0.004 | 0 | Hepatocytes |
| PGLYRP2 | 0 | 2.32E+00 | 0.542 | 0.006 | 0 | Hepatocytes |
| SERPINA6 | 0 | 2.32E+00 | 0.551 | 0 | 0 | Hepatocytes |
| APOM | 0 | 2.23E+00 | 0.486 | 0.009 | 0 | Hepatocytes |
| GGH | 0 | 2.22E+00 | 0.598 | 0.017 | 0 | Hepatocytes |
| LEAP2 | 0 | 2.21E+00 | 0.561 | 0.016 | 0 | Hepatocytes |
| LGALS4 | 0 | 2.19E+00 | 0.505 | 0.002 | 0 | Hepatocytes |
| APOC2 | 0 | 2.15E+00 | 0.598 | 0.002 | 0 | Hepatocytes |
| UGT2B4 | 0 | 2.15E+00 | 0.495 | 0 | 0 | Hepatocytes |
| SERPINA5 | 0 | 2.15E+00 | 0.551 | 0 | 0 | Hepatocytes |
| GATM | 0 | 2.14E+00 | 0.561 | 0.003 | 0 | Hepatocytes |
| NPW | 0 | 2.13E+00 | 0.374 | 0.002 | 0 | Hepatocytes |
| ASGR2 | 0 | 2.13E+00 | 0.533 | 0.001 | 0 | Hepatocytes |
| C8G | 0 | 2.12E+00 | 0.542 | 0.004 | 0 | Hepatocytes |
| C4BPB | 0 | 2.11E+00 | 0.514 | 0.001 | 0 | Hepatocytes |
| C2 | 0 | 2.10E+00 | 0.523 | 0.005 | 0 | Hepatocytes |
| EPCAM | 0 | 2.09E+00 | 0.467 | 0.004 | 0 | Hepatocytes |
| ITIH3 | 0 | 2.07E+00 | 0.486 | 0.001 | 0 | Hepatocytes |
| SDC2 | 0 | 2.07E+00 | 0.523 | 0.002 | 0 | Hepatocytes |
| SLC2A2 | 0 | 2.04E+00 | 0.551 | 0 | 0 | Hepatocytes |
| CES1 | 0 | 2.02E+00 | 0.505 | 0.013 | 0 | Hepatocytes |
| CD24 | 0 | 2.00E+00 | 0.467 | 0.008 | 0 | Hepatocytes |
| ITIH1 | 0 | 1.99E+00 | 0.486 | 0 | 0 | Hepatocytes |
| APOB | 0 | 1.98E+00 | 0.486 | 0.001 | 0 | Hepatocytes |
| DEFB1 | 0 | 1.98E+00 | 0.271 | 0.001 | 0 | Hepatocytes |
| PLG | 0 | 1.96E+00 | 0.542 | 0.001 | 0 | Hepatocytes |
| ALDH2 | 0 | 1.90E+00 | 0.495 | 0.012 | 0 | Hepatocytes |
| SLPI | 0 | 1.90E+00 | 0.449 | 0.001 | 0 | Hepatocytes |
| ADH1A | 0 | 1.84E+00 | 0.505 | 0 | 0 | Hepatocytes |
| SERPINA4 | 0 | 1.83E+00 | 0.514 | 0 | 0 | Hepatocytes |
| TM4SF4 | 0 | 1.81E+00 | 0.477 | 0.001 | 0 | Hepatocytes |
| SERPING1 | 0 | 1.78E+00 | 0.505 | 0.005 | 0 | Hepatocytes |
| PTGR1 | 0 | 1.78E+00 | 0.43 | 0.002 | 0 | Hepatocytes |
| ADH4 | 0 | 1.75E+00 | 0.346 | 0 | 0 | Hepatocytes |
| TST | 0 | 1.75E+00 | 0.505 | 0.009 | 0 | Hepatocytes |
| S100A16 | 0 | 1.73E+00 | 0.467 | 0 | 0 | Hepatocytes |
| TM4SF5 | 0 | 1.72E+00 | 0.467 | 0 | 0 | Hepatocytes |
| C4BPA | 0 | 1.71E+00 | 0.383 | 0.001 | 0 | Hepatocytes |
| SLC22A7 | 0 | 1.71E+00 | 0.439 | 0 | 0 | Hepatocytes |
| C1S | 0 | 1.69E+00 | 0.439 | 0.001 | 0 | Hepatocytes |
| KRT8 | 0 | 1.69E+00 | 0.449 | 0.002 | 0 | Hepatocytes |
| FTCD | 0 | 1.67E+00 | 0.439 | 0 | 0 | Hepatocytes |
| NAT8 | 0 | 1.67E+00 | 0.439 | 0 | 0 | Hepatocytes |
| SAA4 | 0 | 1.67E+00 | 0.402 | 0 | 0 | Hepatocytes |
| BAAT | 0 | 1.66E+00 | 0.383 | 0.001 | 0 | Hepatocytes |
| CYP2C9 | 0 | 1.64E+00 | 0.308 | 0 | 0 | Hepatocytes |
| CPB2 | 0 | 1.63E+00 | 0.467 | 0 | 0 | Hepatocytes |
| C1R | 0 | 1.62E+00 | 0.458 | 0.007 | 0 | Hepatocytes |
| PROC | 0 | 1.57E+00 | 0.477 | 0.003 | 0 | Hepatocytes |
| ARG1 | 0 | 1.56E+00 | 0.327 | 0.002 | 0 | Hepatocytes |
| ADH1B | 0 | 1.56E+00 | 0.383 | 0.001 | 0 | Hepatocytes |
| ALDH1A1 | 0 | 1.53E+00 | 0.411 | 0.002 | 0 | Hepatocytes |
| FN1 | 0 | 1.50E+00 | 0.318 | 0.001 | 0 | Hepatocytes |
| CYP27A1 | 0 | 1.47E+00 | 0.355 | 0 | 0 | Hepatocytes |
| PON3 | 0 | 1.42E+00 | 0.355 | 0.004 | 0 | Hepatocytes |
| TSPAN8 | 0 | 1.42E+00 | 0.299 | 0 | 0 | Hepatocytes |
| GRTP1 | 0 | 1.40E+00 | 0.29 | 0.001 | 0 | Hepatocytes |
| HRG | 0 | 1.38E+00 | 0.327 | 0.001 | 0 | Hepatocytes |
| SERPINF2 | 0 | 1.38E+00 | 0.346 | 0.003 | 0 | Hepatocytes |
| RAB13 | 0 | 1.37E+00 | 0.336 | 0.007 | 0 | Hepatocytes |
| TDO2 | 0 | 1.35E+00 | 0.262 | 0 | 0 | Hepatocytes |
| AADAC | 0 | 1.35E+00 | 0.364 | 0 | 0 | Hepatocytes |
| GJB1 | 0 | 1.32E+00 | 0.346 | 0 | 0 | Hepatocytes |
| LECT2 | 0 | 1.28E+00 | 0.346 | 0 | 0 | Hepatocytes |
| PON1 | 0 | 1.27E+00 | 0.336 | 0.001 | 0 | Hepatocytes |
| TDGF1 | 0 | 1.22E+00 | 0.29 | 0 | 0 | Hepatocytes |
| SLC38A3 | 0 | 1.17E+00 | 0.29 | 0 | 0 | Hepatocytes |
| DPYS | 0 | 1.17E+00 | 0.318 | 0 | 0 | Hepatocytes |
| HPN | 0 | 1.16E+00 | 0.336 | 0.002 | 0 | Hepatocytes |
| GNMT | 0 | 1.15E+00 | 0.271 | 0.002 | 0 | Hepatocytes |
| HAO1 | 0 | 1.13E+00 | 0.327 | 0 | 0 | Hepatocytes |
| A1CF | 0 | 1.13E+00 | 0.28 | 0 | 0 | Hepatocytes |
| HJV | 0 | 1.12E+00 | 0.271 | 0 | 0 | Hepatocytes |
| MST1 | 0 | 1.10E+00 | 0.299 | 0.003 | 0 | Hepatocytes |
| GOLT1A | 0 | 1.08E+00 | 0.308 | 0 | 0 | Hepatocytes |
| RBP1 | 0 | 1.07E+00 | 0.262 | 0 | 0 | Hepatocytes |
| CALD1 | 0 | 1.06E+00 | 0.336 | 0.002 | 0 | Hepatocytes |
| UPB1 | 0 | 1.05E+00 | 0.308 | 0.002 | 0 | Hepatocytes |
| PPIC | 0 | 1.04E+00 | 0.262 | 0.001 | 0 | Hepatocytes |
| PRAP1 | 0 | 9.81E-01 | 0.28 | 0 | 0 | Hepatocytes |
| SELENBP1 | 0 | 9.48E-01 | 0.252 | 0.002 | 0 | Hepatocytes |
| LRG1 | 0 | 9.43E-01 | 0.28 | 0.001 | 0 | Hepatocytes |
| TFPI | 0 | 9.42E-01 | 0.252 | 0.003 | 0 | Hepatocytes |
| C8B | 0 | 9.19E-01 | 0.28 | 0 | 0 | Hepatocytes |
| SGCE | 0 | 8.74E-01 | 0.28 | 0.002 | 0 | Hepatocytes |
| A2M | 1.98E-298 | 1.04E+00 | 0.28 | 0.005 | 6.65E-294 | Hepatocytes |
| MDK | 2.63E-295 | 1.81E+00 | 0.346 | 0.008 | 8.82E-291 | Hepatocytes |
| H1F0 | 3.06E-295 | 1.13E+00 | 0.308 | 0.006 | 1.02E-290 | Hepatocytes |
| FAM213A | 3.08E-289 | 1.57E+00 | 0.439 | 0.013 | 1.03E-284 | Hepatocytes |
| SERINC2 | 8.87E-286 | 1.05E+00 | 0.271 | 0.005 | 2.97E-281 | Hepatocytes |
| SORD | 2.35E-284 | 1.46E+00 | 0.383 | 0.01 | 7.89E-280 | Hepatocytes |
| CFH | 1.31E-278 | 2.37E+00 | 0.598 | 0.027 | 4.41E-274 | Hepatocytes |
| NR2F6 | 7.72E-276 | 1.19E+00 | 0.308 | 0.006 | 2.59E-271 | Hepatocytes |
| PDZK1 | 6.11E-273 | 1.36E+00 | 0.374 | 0.01 | 2.05E-268 | Hepatocytes |
| HP | 4.12E-271 | 4.41E+00 | 0.262 | 0.005 | 1.38E-266 | Hepatocytes |
| FXYD1 | 3.78E-263 | 2.77E+00 | 0.673 | 0.037 | 1.27E-258 | Hepatocytes |
| SLC9A3R2 | 2.11E-256 | 1.05E+00 | 0.327 | 0.008 | 7.09E-252 | Hepatocytes |
| TFR2 | 1.29E-255 | 8.34E-01 | 0.262 | 0.005 | 4.32E-251 | Hepatocytes |
| FMO5 | 6.85E-237 | 9.56E-01 | 0.262 | 0.005 | 2.30E-232 | Hepatocytes |
| RIDA | 8.09E-234 | 1.77E+00 | 0.486 | 0.02 | 2.71E-229 | Hepatocytes |
| ANPEP | 5.29E-218 | 1.06E+00 | 0.262 | 0.006 | 1.77E-213 | Hepatocytes |
| GAMT | 1.99E-210 | 2.51E+00 | 0.645 | 0.043 | 6.69E-206 | Hepatocytes |
| TM7SF2 | 1.81E-204 | 1.54E+00 | 0.477 | 0.023 | 6.06E-200 | Hepatocytes |
| BEX3 | 5.11E-204 | 2.04E+00 | 0.579 | 0.034 | 1.71E-199 | Hepatocytes |
| TCEA3 | 2.26E-195 | 1.14E+00 | 0.299 | 0.009 | 7.58E-191 | Hepatocytes |
| CD302 | 5.03E-188 | 1.08E+00 | 0.318 | 0.011 | 1.69E-183 | Hepatocytes |
| SLC37A4 | 6.21E-173 | 1.31E+00 | 0.318 | 0.012 | 2.08E-168 | Hepatocytes |
| PXMP2 | 4.73E-168 | 1.39E+00 | 0.43 | 0.022 | 1.59E-163 | Hepatocytes |
| PCK2 | 7.58E-165 | 1.08E+00 | 0.364 | 0.016 | 2.54E-160 | Hepatocytes |
| ATF5 | 4.78E-164 | 3.40E+00 | 0.785 | 0.086 | 1.60E-159 | Hepatocytes |
| MSRB2 | 2.75E-158 | 1.75E+00 | 0.458 | 0.027 | 9.23E-154 | Hepatocytes |
| SCCPDH | 4.03E-149 | 1.80E+00 | 0.598 | 0.05 | 1.35E-144 | Hepatocytes |
| ACAT1 | 1.20E-143 | 1.74E+00 | 0.523 | 0.04 | 4.02E-139 | Hepatocytes |
| GALK1 | 2.68E-143 | 1.76E+00 | 0.467 | 0.032 | 9.00E-139 | Hepatocytes |
| CDO1 | 8.15E-141 | 1.34E+00 | 0.467 | 0.03 | 2.73E-136 | Hepatocytes |
| PTMS | 2.73E-136 | 2.87E+00 | 0.804 | 0.107 | 9.15E-132 | Hepatocytes |
| TPM1 | 5.82E-135 | 1.37E+00 | 0.355 | 0.019 | 1.95E-130 | Hepatocytes |
| CYB5A | 2.22E-129 | 3.69E+00 | 0.897 | 0.158 | 7.46E-125 | Hepatocytes |
| MGST2 | 6.08E-129 | 2.08E+00 | 0.57 | 0.053 | 2.04E-124 | Hepatocytes |
| PGRMC1 | 1.21E-125 | 2.32E+00 | 0.636 | 0.069 | 4.04E-121 | Hepatocytes |
| PPP1R16A | 1.25E-121 | 1.48E+00 | 0.383 | 0.025 | 4.20E-117 | Hepatocytes |
| LAPTM4B | 4.09E-114 | 1.04E+00 | 0.327 | 0.019 | 1.37E-109 | Hepatocytes |
| SCD | 1.50E-113 | 1.88E+00 | 0.43 | 0.033 | 5.03E-109 | Hepatocytes |
| GCSH | 2.86E-113 | 1.39E+00 | 0.402 | 0.029 | 9.58E-109 | Hepatocytes |
| PCBD1 | 3.86E-111 | 2.13E+00 | 0.617 | 0.072 | 1.29E-106 | Hepatocytes |
| A1BG | 1.33E-105 | 2.75E+00 | 0.738 | 0.116 | 4.45E-101 | Hepatocytes |
| TMEM220 | 8.60E-105 | 1.16E+00 | 0.364 | 0.025 | 2.88E-100 | Hepatocytes |
| FCGRT | 1.04E-102 | 1.71E+00 | 0.551 | 0.06 | 3.49E-98 | Hepatocytes |
| FAH | 1.88E-102 | 8.08E-01 | 0.262 | 0.013 | 6.29E-98 | Hepatocytes |
| QPRT | 3.62E-102 | 1.01E+00 | 0.29 | 0.017 | 1.22E-97 | Hepatocytes |
| SMCO4 | 1.70E-96 | 1.13E+00 | 0.364 | 0.028 | 5.70E-92 | Hepatocytes |
| CST3 | 1.53E-91 | 1.42E+00 | 0.729 | 0.116 | 5.12E-87 | Hepatocytes |
| CBR1 | 7.79E-91 | 1.87E+00 | 0.533 | 0.063 | 2.61E-86 | Hepatocytes |
| BRI3 | 9.73E-90 | 2.72E+00 | 0.841 | 0.191 | 3.26E-85 | Hepatocytes |
| AAMDC | 9.13E-88 | 1.11E+00 | 0.355 | 0.028 | 3.06E-83 | Hepatocytes |
| COPRS | 2.35E-85 | 9.60E-01 | 0.318 | 0.024 | 7.89E-81 | Hepatocytes |
| IGFBP4 | 9.86E-78 | 1.42E+00 | 0.43 | 0.046 | 3.31E-73 | Hepatocytes |
| CEBPA | 9.90E-78 | 1.25E+00 | 0.374 | 0.036 | 3.32E-73 | Hepatocytes |
| PON2 | 3.62E-76 | 1.23E+00 | 0.374 | 0.036 | 1.22E-71 | Hepatocytes |
| ECI2 | 5.36E-76 | 1.66E+00 | 0.542 | 0.077 | 1.80E-71 | Hepatocytes |
| CMTM8 | 1.05E-75 | 1.13E+00 | 0.29 | 0.022 | 3.53E-71 | Hepatocytes |
| ACSL4 | 1.49E-73 | 1.35E+00 | 0.393 | 0.042 | 5.00E-69 | Hepatocytes |
| IFITM3 | 1.82E-71 | 3.36E+00 | 0.981 | 0.499 | 6.11E-67 | Hepatocytes |
| MTHFS | 7.19E-70 | 1.21E+00 | 0.421 | 0.049 | 2.41E-65 | Hepatocytes |
| THOP1 | 1.19E-68 | 9.88E-01 | 0.299 | 0.026 | 4.00E-64 | Hepatocytes |
| AIG1 | 2.38E-68 | 1.75E+00 | 0.514 | 0.077 | 7.97E-64 | Hepatocytes |
| CDKN2A | 6.00E-67 | 1.15E+00 | 0.393 | 0.044 | 2.01E-62 | Hepatocytes |
| DNPH1 | 7.06E-66 | 1.96E+00 | 0.542 | 0.09 | 2.37E-61 | Hepatocytes |
| MTCH2 | 1.15E-65 | 1.56E+00 | 0.495 | 0.074 | 3.87E-61 | Hepatocytes |
| FTL | 1.61E-64 | 3.01E+00 | 1 | 0.963 | 5.41E-60 | Hepatocytes |
| PAICS | 5.76E-64 | 8.00E-01 | 0.299 | 0.027 | 1.93E-59 | Hepatocytes |
| PCYT2 | 1.35E-63 | 9.12E-01 | 0.299 | 0.027 | 4.51E-59 | Hepatocytes |
| MPC2 | 3.00E-63 | 2.67E+00 | 0.86 | 0.335 | 1.01E-58 | Hepatocytes |
| ETFB | 8.05E-62 | 2.33E+00 | 0.701 | 0.181 | 2.70E-57 | Hepatocytes |
| PEBP1 | 1.33E-60 | 2.65E+00 | 0.869 | 0.354 | 4.47E-56 | Hepatocytes |
| NDUFC1 | 3.23E-60 | 1.93E+00 | 0.561 | 0.103 | 1.08E-55 | Hepatocytes |
| ECHS1 | 8.03E-59 | 1.94E+00 | 0.673 | 0.163 | 2.69E-54 | Hepatocytes |
| G0S2 | 1.19E-58 | 1.18E+00 | 0.28 | 0.026 | 3.98E-54 | Hepatocytes |
| PHYH | 3.56E-55 | 1.80E+00 | 0.533 | 0.099 | 1.19E-50 | Hepatocytes |
| ECI1 | 5.92E-55 | 1.26E+00 | 0.421 | 0.061 | 1.99E-50 | Hepatocytes |
| STARD10 | 7.46E-55 | 1.62E+00 | 0.551 | 0.107 | 2.50E-50 | Hepatocytes |
| LGALS3BP | 1.59E-54 | 1.12E+00 | 0.393 | 0.054 | 5.32E-50 | Hepatocytes |
| FUOM | 8.50E-54 | 1.36E+00 | 0.421 | 0.063 | 2.85E-49 | Hepatocytes |
| PDLIM1 | 1.24E-53 | 1.37E+00 | 0.467 | 0.076 | 4.15E-49 | Hepatocytes |
| PRDX3 | 3.68E-53 | 1.28E+00 | 0.43 | 0.066 | 1.23E-48 | Hepatocytes |
| UGDH | 1.27E-51 | 9.19E-01 | 0.262 | 0.026 | 4.24E-47 | Hepatocytes |
| PECR | 5.22E-51 | 7.98E-01 | 0.262 | 0.026 | 1.75E-46 | Hepatocytes |
| FBP1 | 1.13E-48 | 1.25E+00 | 0.364 | 0.051 | 3.79E-44 | Hepatocytes |
| CAMK2N1 | 1.50E-46 | 7.57E-01 | 0.271 | 0.03 | 5.03E-42 | Hepatocytes |
| DCXR | 2.41E-46 | 2.13E+00 | 0.766 | 0.28 | 8.10E-42 | Hepatocytes |
| LPCAT3 | 1.68E-44 | 1.00E+00 | 0.299 | 0.038 | 5.64E-40 | Hepatocytes |
| TCEAL4 | 2.18E-43 | 1.03E+00 | 0.355 | 0.054 | 7.30E-39 | Hepatocytes |
| ATP5PF | 2.18E-43 | 2.19E+00 | 0.822 | 0.402 | 7.32E-39 | Hepatocytes |
| LSR | 4.99E-43 | 1.45E+00 | 0.393 | 0.067 | 1.67E-38 | Hepatocytes |
| SEC11C | 8.27E-43 | 2.12E+00 | 0.71 | 0.249 | 2.77E-38 | Hepatocytes |
| TMEM14C | 8.44E-43 | 1.51E+00 | 0.523 | 0.116 | 2.83E-38 | Hepatocytes |
| PSMB5 | 5.04E-42 | 1.49E+00 | 0.43 | 0.081 | 1.69E-37 | Hepatocytes |
| MPST | 5.35E-42 | 1.68E+00 | 0.533 | 0.125 | 1.80E-37 | Hepatocytes |
| GRHPR | 1.26E-41 | 1.36E+00 | 0.486 | 0.101 | 4.21E-37 | Hepatocytes |
| ADI1 | 1.58E-40 | 1.84E+00 | 0.542 | 0.136 | 5.29E-36 | Hepatocytes |
| COA3 | 1.03E-39 | 1.28E+00 | 0.439 | 0.086 | 3.47E-35 | Hepatocytes |
| ACOT13 | 1.60E-39 | 9.12E-01 | 0.28 | 0.038 | 5.37E-35 | Hepatocytes |
| PSMG1 | 4.30E-39 | 8.76E-01 | 0.262 | 0.033 | 1.44E-34 | Hepatocytes |
| PEMT | 4.54E-39 | 1.08E+00 | 0.271 | 0.035 | 1.52E-34 | Hepatocytes |
| TXN | 1.42E-38 | 2.33E+00 | 0.813 | 0.409 | 4.75E-34 | Hepatocytes |
| GLRX5 | 4.43E-38 | 1.52E+00 | 0.589 | 0.163 | 1.48E-33 | Hepatocytes |
| PRDX4 | 5.89E-38 | 1.25E+00 | 0.43 | 0.085 | 1.98E-33 | Hepatocytes |
| BNIP3 | 1.40E-37 | 1.14E+00 | 0.336 | 0.056 | 4.70E-33 | Hepatocytes |
| SPATS2L | 1.90E-36 | 1.51E+00 | 0.486 | 0.116 | 6.36E-32 | Hepatocytes |
| ANXA4 | 1.91E-36 | 1.18E+00 | 0.383 | 0.071 | 6.39E-32 | Hepatocytes |
| ARL4D | 2.18E-36 | 1.10E+00 | 0.336 | 0.055 | 7.31E-32 | Hepatocytes |
| HEBP1 | 1.60E-35 | 9.16E-01 | 0.271 | 0.038 | 5.36E-31 | Hepatocytes |
| SCOC | 1.86E-35 | 9.17E-01 | 0.29 | 0.043 | 6.24E-31 | Hepatocytes |
| NENF | 9.51E-35 | 1.25E+00 | 0.477 | 0.114 | 3.19E-30 | Hepatocytes |
| AKR1C3 | 2.51E-34 | 1.56E+00 | 0.467 | 0.109 | 8.40E-30 | Hepatocytes |
| GLO1 | 2.25E-33 | 1.30E+00 | 0.514 | 0.135 | 7.53E-29 | Hepatocytes |
| CRYL1 | 7.98E-33 | 8.84E-01 | 0.308 | 0.052 | 2.68E-28 | Hepatocytes |
| NAA20 | 1.51E-32 | 1.31E+00 | 0.421 | 0.096 | 5.05E-28 | Hepatocytes |
| FTH1 | 2.15E-32 | 1.00E+00 | 1 | 0.993 | 7.22E-28 | Hepatocytes |
| ACAT2 | 1.21E-31 | 1.42E+00 | 0.421 | 0.097 | 4.07E-27 | Hepatocytes |
| TMEM205 | 2.09E-31 | 1.32E+00 | 0.421 | 0.097 | 7.02E-27 | Hepatocytes |
| CHCHD10 | 2.12E-31 | 1.50E+00 | 0.551 | 0.166 | 7.11E-27 | Hepatocytes |
| NDUFC2 | 3.33E-31 | 1.82E+00 | 0.71 | 0.331 | 1.12E-26 | Hepatocytes |
| FH | 3.83E-31 | 9.26E-01 | 0.271 | 0.043 | 1.29E-26 | Hepatocytes |
| TMEM38B | 7.27E-31 | 8.59E-01 | 0.252 | 0.038 | 2.44E-26 | Hepatocytes |
| HSDL2 | 1.18E-30 | 5.93E-01 | 0.271 | 0.042 | 3.97E-26 | Hepatocytes |
| DDT | 8.10E-30 | 1.86E+00 | 0.757 | 0.394 | 2.72E-25 | Hepatocytes |
| ERG28 | 9.29E-29 | 1.29E+00 | 0.402 | 0.096 | 3.12E-24 | Hepatocytes |
| IGFBP2 | 1.30E-28 | 1.33E+00 | 0.336 | 0.068 | 4.37E-24 | Hepatocytes |
| CLTA | 6.98E-28 | 1.32E+00 | 0.598 | 0.212 | 2.34E-23 | Hepatocytes |
| CISD3 | 1.14E-27 | 1.28E+00 | 0.467 | 0.126 | 3.82E-23 | Hepatocytes |
| MGMT | 1.77E-27 | 1.37E+00 | 0.514 | 0.156 | 5.93E-23 | Hepatocytes |
| UQCC2 | 5.14E-27 | 1.02E+00 | 0.421 | 0.105 | 1.72E-22 | Hepatocytes |
| CERS2 | 7.13E-27 | 1.20E+00 | 0.43 | 0.115 | 2.39E-22 | Hepatocytes |
| COA6 | 9.12E-27 | 9.93E-01 | 0.355 | 0.078 | 3.06E-22 | Hepatocytes |
| SIGMAR1 | 1.16E-26 | 8.73E-01 | 0.271 | 0.048 | 3.89E-22 | Hepatocytes |
| NIPSNAP1 | 1.38E-26 | 7.08E-01 | 0.262 | 0.045 | 4.64E-22 | Hepatocytes |
| PRDX6 | 1.93E-26 | 1.53E+00 | 0.785 | 0.488 | 6.48E-22 | Hepatocytes |
| TMEM14A | 4.02E-26 | 8.35E-01 | 0.271 | 0.049 | 1.35E-21 | Hepatocytes |
| ADH5 | 1.51E-25 | 1.05E+00 | 0.421 | 0.109 | 5.06E-21 | Hepatocytes |
| DHRS4L2 | 2.75E-25 | 8.12E-01 | 0.336 | 0.074 | 9.21E-21 | Hepatocytes |
| GTF2H5 | 4.51E-25 | 1.25E+00 | 0.402 | 0.103 | 1.51E-20 | Hepatocytes |
| CHPT1 | 6.33E-25 | 1.17E+00 | 0.477 | 0.143 | 2.12E-20 | Hepatocytes |
| FAM200B | 6.53E-25 | 1.18E+00 | 0.411 | 0.108 | 2.19E-20 | Hepatocytes |
| SLC2A4RG | 7.44E-25 | 1.05E+00 | 0.402 | 0.101 | 2.50E-20 | Hepatocytes |
| TCEAL8 | 2.54E-24 | 9.85E-01 | 0.374 | 0.093 | 8.52E-20 | Hepatocytes |
| CD151 | 1.10E-23 | 9.86E-01 | 0.402 | 0.106 | 3.70E-19 | Hepatocytes |
| PDCD5 | 1.18E-23 | 1.19E+00 | 0.467 | 0.145 | 3.96E-19 | Hepatocytes |
| NDUFS6 | 1.26E-23 | 1.46E+00 | 0.654 | 0.305 | 4.23E-19 | Hepatocytes |
| GSTO1 | 1.71E-23 | 1.45E+00 | 0.561 | 0.216 | 5.75E-19 | Hepatocytes |
| SEM1 | 2.03E-23 | 1.74E+00 | 0.673 | 0.35 | 6.82E-19 | Hepatocytes |
| PRDX2 | 2.60E-23 | 1.41E+00 | 0.598 | 0.252 | 8.71E-19 | Hepatocytes |
| MRPL14 | 4.54E-23 | 1.30E+00 | 0.514 | 0.184 | 1.52E-18 | Hepatocytes |
| POLD2 | 4.68E-23 | 9.08E-01 | 0.299 | 0.064 | 1.57E-18 | Hepatocytes |
| LAMTOR2 | 5.92E-23 | 1.13E+00 | 0.505 | 0.17 | 1.99E-18 | Hepatocytes |
| P4HB | 6.09E-23 | 1.50E+00 | 0.72 | 0.4 | 2.04E-18 | Hepatocytes |
| ATP5ME | 3.34E-22 | 1.57E+00 | 0.729 | 0.425 | 1.12E-17 | Hepatocytes |
| DBI | 4.19E-22 | 1.53E+00 | 0.822 | 0.653 | 1.40E-17 | Hepatocytes |
| GLUD1 | 4.31E-22 | 1.45E+00 | 0.551 | 0.216 | 1.45E-17 | Hepatocytes |
| MRPS33 | 7.22E-22 | 1.33E+00 | 0.364 | 0.098 | 2.42E-17 | Hepatocytes |
| ATP5MC1 | 8.04E-22 | 1.22E+00 | 0.523 | 0.186 | 2.70E-17 | Hepatocytes |
| ISOC2 | 8.35E-22 | 1.00E+00 | 0.28 | 0.06 | 2.80E-17 | Hepatocytes |
| ANAPC11 | 1.01E-21 | 1.41E+00 | 0.645 | 0.312 | 3.38E-17 | Hepatocytes |
| PERP | 1.10E-21 | 9.36E-01 | 0.308 | 0.071 | 3.68E-17 | Hepatocytes |
| IFI6 | 1.20E-21 | 1.39E+00 | 0.673 | 0.356 | 4.01E-17 | Hepatocytes |
| MRPL2 | 1.46E-21 | 7.72E-01 | 0.308 | 0.07 | 4.88E-17 | Hepatocytes |
| LAMP2 | 3.19E-21 | 8.07E-01 | 0.364 | 0.094 | 1.07E-16 | Hepatocytes |
| COMT | 3.72E-21 | 1.18E+00 | 0.393 | 0.117 | 1.25E-16 | Hepatocytes |
| POLR2F | 4.69E-21 | 1.33E+00 | 0.561 | 0.217 | 1.57E-16 | Hepatocytes |
| GPX4 | 6.85E-21 | 1.33E+00 | 0.785 | 0.593 | 2.30E-16 | Hepatocytes |
| ETFRF1 | 1.91E-20 | 0.88484986 | 0.355 | 0.093 | 6.39E-16 | Hepatocytes |
| MSMO1 | 1.91E-20 | 0.95678432 | 0.29 | 0.068 | 6.40E-16 | Hepatocytes |
| NDUFV3 | 2.14E-20 | 0.77539739 | 0.28 | 0.063 | 7.18E-16 | Hepatocytes |
| ARPC1A | 2.25E-20 | 0.65089672 | 0.262 | 0.055 | 7.56E-16 | Hepatocytes |
| FKBP2 | 1.14E-19 | 1.20732963 | 0.589 | 0.251 | 3.83E-15 | Hepatocytes |
| AGPAT2 | 1.95E-19 | 1.12981524 | 0.318 | 0.084 | 6.55E-15 | Hepatocytes |
| SDHC | 2.12E-19 | 0.9514894 | 0.458 | 0.155 | 7.12E-15 | Hepatocytes |
| HINT2 | 1.01E-18 | 1.05862163 | 0.411 | 0.13 | 3.38E-14 | Hepatocytes |
| FDPS | 1.24E-18 | 1.56485609 | 0.523 | 0.221 | 4.17E-14 | Hepatocytes |
| SQLE | 2.15E-18 | 1.00220643 | 0.346 | 0.1 | 7.20E-14 | Hepatocytes |
| HINT1 | 2.63E-18 | 0.944532 | 0.86 | 0.773 | 8.84E-14 | Hepatocytes |
| HACD3 | 5.63E-18 | 7.92E-01 | 0.271 | 0.066 | 1.89E-13 | Hepatocytes |
| ATP5PD | 8.13E-18 | 1.43E+00 | 0.617 | 0.327 | 2.73E-13 | Hepatocytes |
| MRPL27 | 9.23E-18 | 8.38E-01 | 0.308 | 0.083 | 3.10E-13 | Hepatocytes |
| EBP | 1.66E-17 | 1.23E+00 | 0.579 | 0.276 | 5.55E-13 | Hepatocytes |
| ERH | 1.67E-17 | 1.42E+00 | 0.579 | 0.274 | 5.61E-13 | Hepatocytes |
| CFL2 | 2.00E-17 | 8.13E-01 | 0.299 | 0.078 | 6.72E-13 | Hepatocytes |
| ATOX1 | 3.21E-17 | 1.21E+00 | 0.477 | 0.183 | 1.08E-12 | Hepatocytes |
| NDUFB1 | 3.54E-17 | 1.23E+00 | 0.589 | 0.276 | 1.19E-12 | Hepatocytes |
| BST2 | 3.67E-17 | 1.17E+00 | 0.766 | 0.564 | 1.23E-12 | Hepatocytes |
| NDUFB7 | 3.96E-17 | 1.26E+00 | 0.607 | 0.295 | 1.33E-12 | Hepatocytes |
| EBAG9 | 4.08E-17 | 6.62E-01 | 0.252 | 0.059 | 1.37E-12 | Hepatocytes |
| TMEM14B | 6.73E-17 | 1.35E+00 | 0.505 | 0.219 | 2.26E-12 | Hepatocytes |
| MRPL13 | 7.90E-17 | 8.05E-01 | 0.29 | 0.076 | 2.65E-12 | Hepatocytes |
| NDUFB4 | 1.49E-16 | 1.25E+00 | 0.645 | 0.377 | 4.99E-12 | Hepatocytes |
| COX6C | 1.53E-16 | 1.17635687 | 0.804 | 0.677 | 5.15E-12 | Hepatocytes |
| CAT | 2.18E-16 | 0.8685238 | 0.383 | 0.125 | 7.31E-12 | Hepatocytes |
| SEC61B | 2.20E-16 | 1.25268356 | 0.804 | 0.595 | 7.36E-12 | Hepatocytes |
| PPIB | 2.44E-16 | 1.23168469 | 0.794 | 0.652 | 8.18E-12 | Hepatocytes |
| HEBP2 | 4.29E-16 | 0.97145721 | 0.467 | 0.172 | 1.44E-11 | Hepatocytes |
| HADHB | 4.36E-16 | 0.79105072 | 0.383 | 0.126 | 1.46E-11 | Hepatocytes |
| PEPD | 4.37E-16 | 0.98603247 | 0.355 | 0.113 | 1.46E-11 | Hepatocytes |
| YIPF3 | 4.40E-16 | 0.89014295 | 0.355 | 0.109 | 1.48E-11 | Hepatocytes |
| OAS1 | 5.24E-16 | 0.97456413 | 0.29 | 0.08 | 1.76E-11 | Hepatocytes |
| NUCKS1 | 6.23E-16 | 1.30612843 | 0.617 | 0.333 | 2.09E-11 | Hepatocytes |
| CNIH4 | 9.14E-16 | 1.13093307 | 0.327 | 0.101 | 3.06E-11 | Hepatocytes |
| HSPB1 | 2.10E-15 | 0.66920183 | 0.804 | 0.461 | 7.03E-11 | Hepatocytes |
| DHRS7 | 4.46E-15 | 1.01889539 | 0.72 | 0.464 | 1.50E-10 | Hepatocytes |
| MPLKIP | 5.73E-15 | 0.86335601 | 0.355 | 0.116 | 1.92E-10 | Hepatocytes |
| NDUFB11 | 5.93E-15 | 1.09812436 | 0.738 | 0.535 | 1.99E-10 | Hepatocytes |
| MRPS26 | 6.96E-15 | 0.63128843 | 0.318 | 0.094 | 2.33E-10 | Hepatocytes |
| MRPS18B | 7.41E-15 | 0.53776373 | 0.271 | 0.073 | 2.49E-10 | Hepatocytes |
| CPQ | 1.25E-14 | 0.8062365 | 0.28 | 0.079 | 4.20E-10 | Hepatocytes |
| UQCRQ | 1.29E-14 | 1.28247779 | 0.673 | 0.456 | 4.34E-10 | Hepatocytes |
| SNF8 | 1.83E-14 | 0.90963679 | 0.458 | 0.186 | 6.14E-10 | Hepatocytes |
| RNF5 | 2.11E-14 | 0.85298533 | 0.393 | 0.146 | 7.07E-10 | Hepatocytes |
| PQLC1 | 2.53E-14 | 0.75886576 | 0.271 | 0.076 | 8.47E-10 | Hepatocytes |
| METTL5 | 2.64E-14 | 0.94128686 | 0.346 | 0.114 | 8.85E-10 | Hepatocytes |
| ACBD6 | 3.15E-14 | 0.65112646 | 0.299 | 0.087 | 1.06E-09 | Hepatocytes |
| TMEM106C | 3.19E-14 | 0.94502571 | 0.29 | 0.086 | 1.07E-09 | Hepatocytes |
| COA4 | 3.64E-14 | 0.84028969 | 0.346 | 0.117 | 1.22E-09 | Hepatocytes |
| ATP5MD | 4.08E-14 | 1.24096365 | 0.701 | 0.469 | 1.37E-09 | Hepatocytes |
| NDUFB3 | 4.74E-14 | 0.99829145 | 0.421 | 0.162 | 1.59E-09 | Hepatocytes |
| POR | 5.34E-14 | 0.76813917 | 0.336 | 0.113 | 1.79E-09 | Hepatocytes |
| ECH1 | 6.41E-14 | 1.15300854 | 0.598 | 0.345 | 2.15E-09 | Hepatocytes |
| PSMF1 | 7.42E-14 | 0.80718773 | 0.458 | 0.183 | 2.49E-09 | Hepatocytes |
| CREG1 | 7.64E-14 | 0.74842487 | 0.28 | 0.081 | 2.56E-09 | Hepatocytes |
| NDUFV2 | 9.22E-14 | 1.21005703 | 0.57 | 0.289 | 3.09E-09 | Hepatocytes |
| BOLA3 | 9.80E-14 | 0.87657617 | 0.299 | 0.093 | 3.29E-09 | Hepatocytes |
| COX5B | 9.86E-14 | 0.88977051 | 0.757 | 0.563 | 3.31E-09 | Hepatocytes |
| GRINA | 1.02E-13 | 0.62922502 | 0.28 | 0.081 | 3.41E-09 | Hepatocytes |
| GCHFR | 1.75E-13 | 0.91995209 | 0.374 | 0.139 | 5.86E-09 | Hepatocytes |
| ATP5MC3 | 1.79E-13 | 1.04018224 | 0.701 | 0.466 | 6.00E-09 | Hepatocytes |
| NME1 | 1.80E-13 | 0.90434275 | 0.28 | 0.087 | 6.04E-09 | Hepatocytes |
| MRPL41 | 2.17E-13 | 1.0543195 | 0.57 | 0.293 | 7.26E-09 | Hepatocytes |
| SPCS2 | 2.76E-13 | 1.19705393 | 0.645 | 0.425 | 9.25E-09 | Hepatocytes |
| LSM6 | 5.19E-13 | 0.72523893 | 0.318 | 0.103 | 1.74E-08 | Hepatocytes |
| MRPL34 | 5.41E-13 | 0.69919328 | 0.383 | 0.139 | 1.81E-08 | Hepatocytes |
| PDIA6 | 6.02E-13 | 1.04503761 | 0.607 | 0.353 | 2.02E-08 | Hepatocytes |
| CTSZ | 9.67E-13 | 0.80094125 | 0.393 | 0.15 | 3.24E-08 | Hepatocytes |
| GADD45GIP1 | 1.02E-12 | 1.07510444 | 0.57 | 0.291 | 3.44E-08 | Hepatocytes |
| PMVK | 1.18E-12 | 0.6871857 | 0.327 | 0.111 | 3.97E-08 | Hepatocytes |
| BLVRB | 1.28E-12 | 0.7555899 | 0.411 | 0.162 | 4.29E-08 | Hepatocytes |
| PHB | 1.66E-12 | 0.99808294 | 0.495 | 0.238 | 5.56E-08 | Hepatocytes |
| SMIM19 | 1.75E-12 | 0.87208536 | 0.374 | 0.141 | 5.86E-08 | Hepatocytes |
| EBPL | 4.39E-12 | 0.86034909 | 0.299 | 0.103 | 1.47E-07 | Hepatocytes |
| SMIM26 | 4.44E-12 | 1.07542116 | 0.533 | 0.275 | 1.49E-07 | Hepatocytes |
| HIGD1A | 4.78E-12 | 0.80239708 | 0.308 | 0.105 | 1.60E-07 | Hepatocytes |
| SRP9 | 6.13E-12 | 0.8161522 | 0.523 | 0.258 | 2.06E-07 | Hepatocytes |
| YIF1A | 6.64E-12 | 0.65929951 | 0.29 | 0.094 | 2.23E-07 | Hepatocytes |
| NUDT5 | 9.58E-12 | 0.91627676 | 0.318 | 0.112 | 3.21E-07 | Hepatocytes |
| SIVA1 | 1.24E-11 | 1.14833405 | 0.542 | 0.284 | 4.16E-07 | Hepatocytes |
| CHID1 | 1.55E-11 | 0.64773396 | 0.262 | 0.083 | 5.21E-07 | Hepatocytes |
| NAXE | 1.81E-11 | 0.64523959 | 0.364 | 0.137 | 6.07E-07 | Hepatocytes |
| MGST3 | 2.01E-11 | 0.93953583 | 0.421 | 0.192 | 6.75E-07 | Hepatocytes |
| MRPL12 | 2.10E-11 | 0.66398745 | 0.28 | 0.093 | 7.04E-07 | Hepatocytes |
| MYDGF | 2.44E-11 | 1.08798428 | 0.636 | 0.376 | 8.17E-07 | Hepatocytes |
| STOML2 | 2.64E-11 | 0.85788873 | 0.327 | 0.123 | 8.86E-07 | Hepatocytes |
| UQCR10 | 3.45E-11 | 1.17406565 | 0.645 | 0.417 | 1.16E-06 | Hepatocytes |
| SNRPC | 3.49E-11 | 1.06866288 | 0.458 | 0.229 | 1.17E-06 | Hepatocytes |
| TMED2 | 4.07E-11 | 0.91643101 | 0.579 | 0.312 | 1.36E-06 | Hepatocytes |
| DECR1 | 4.37E-11 | 0.74057421 | 0.383 | 0.161 | 1.47E-06 | Hepatocytes |
| JAGN1 | 6.91E-11 | 0.61382295 | 0.252 | 0.08 | 2.32E-06 | Hepatocytes |
| GAPDH | 7.13E-11 | 0.67472959 | 0.963 | 0.93 | 2.39E-06 | Hepatocytes |
| SNRPE | 7.30E-11 | 0.94284077 | 0.477 | 0.237 | 2.45E-06 | Hepatocytes |
| FAM96A | 1.18E-10 | 0.4593834 | 0.271 | 0.089 | 3.95E-06 | Hepatocytes |
| OCIAD1 | 1.90E-10 | 0.91657484 | 0.486 | 0.252 | 6.37E-06 | Hepatocytes |
| MRPL36 | 2.48E-10 | 0.73045793 | 0.28 | 0.099 | 8.33E-06 | Hepatocytes |
| VKORC1 | 2.78E-10 | 0.69906905 | 0.336 | 0.13 | 9.32E-06 | Hepatocytes |
| PPP1R14B | 3.22E-10 | 0.79950109 | 0.336 | 0.139 | 1.08E-05 | Hepatocytes |
| DYNLL1 | 3.31E-10 | 0.80672115 | 0.757 | 0.584 | 1.11E-05 | Hepatocytes |
| TALDO1 | 3.34E-10 | 0.8729375 | 0.458 | 0.22 | 1.12E-05 | Hepatocytes |
| VEGFB | 3.37E-10 | 0.58723284 | 0.29 | 0.105 | 1.13E-05 | Hepatocytes |
| STAU1 | 3.41E-10 | 0.82343964 | 0.439 | 0.196 | 1.14E-05 | Hepatocytes |
| RDH11 | 3.52E-10 | 0.60840995 | 0.271 | 0.092 | 1.18E-05 | Hepatocytes |
| DHCR7 | 3.53E-10 | 0.67138474 | 0.271 | 0.092 | 1.18E-05 | Hepatocytes |
| MRPL40 | 3.64E-10 | 0.59104449 | 0.29 | 0.104 | 1.22E-05 | Hepatocytes |
| NDUFA11 | 4.08E-10 | 0.93300397 | 0.607 | 0.406 | 1.37E-05 | Hepatocytes |
| TIMM13 | 4.24E-10 | 0.80348138 | 0.421 | 0.19 | 1.42E-05 | Hepatocytes |
| DDAH2 | 5.06E-10 | 0.7652707 | 0.308 | 0.116 | 1.70E-05 | Hepatocytes |
| KDELR1 | 5.30E-10 | 0.74258778 | 0.402 | 0.18 | 1.78E-05 | Hepatocytes |
| PHPT1 | 6.10E-10 | 0.95639836 | 0.561 | 0.34 | 2.05E-05 | Hepatocytes |
| HSD17B11 | 1.03E-09 | 0.82242805 | 0.458 | 0.218 | 3.45E-05 | Hepatocytes |
| CEBPD | 1.19E-09 | 0.9621093 | 0.589 | 0.367 | 4.01E-05 | Hepatocytes |
| AKR1A1 | 1.54E-09 | 0.94864285 | 0.327 | 0.138 | 5.16E-05 | Hepatocytes |
| PAIP1 | 1.98E-09 | 0.77703507 | 0.252 | 0.086 | 6.63E-05 | Hepatocytes |
| MRPL55 | 2.11E-09 | 0.66224343 | 0.346 | 0.145 | 7.06E-05 | Hepatocytes |
| MRPL57 | 2.29E-09 | 0.89011656 | 0.533 | 0.294 | 7.67E-05 | Hepatocytes |
| GNG5 | 2.42E-09 | 0.92249087 | 0.645 | 0.442 | 8.12E-05 | Hepatocytes |
| MRPL51 | 2.45E-09 | 0.8944051 | 0.477 | 0.255 | 8.22E-05 | Hepatocytes |
| DUSP23 | 3.72E-09 | 0.50471818 | 0.271 | 0.099 | 0.000124859 | Hepatocytes |
| ZNHIT1 | 3.78E-09 | 0.72051824 | 0.505 | 0.267 | 0.000126797 | Hepatocytes |
| KRTCAP2 | 3.87E-09 | 0.81103546 | 0.449 | 0.224 | 0.000129922 | Hepatocytes |
| GLUL | 4.86E-09 | 0.87606174 | 0.551 | 0.322 | 0.000162868 | Hepatocytes |
| PRDX1 | 4.87E-09 | 0.68978226 | 0.561 | 0.339 | 0.000163168 | Hepatocytes |
| RNF181 | 5.27E-09 | 0.79278229 | 0.467 | 0.243 | 0.000176621 | Hepatocytes |
| NDUFA12 | 6.56E-09 | 0.94124763 | 0.542 | 0.324 | 0.000219987 | Hepatocytes |
| PIGC | 7.22E-09 | 0.46653388 | 0.262 | 0.094 | 0.000242265 | Hepatocytes |
| ALKBH7 | 8.63E-09 | 0.71709239 | 0.43 | 0.212 | 0.00028935 | Hepatocytes |
| ATP1B1 | 9.23E-09 | 0.68363685 | 0.318 | 0.134 | 0.000309391 | Hepatocytes |
| SSR1 | 9.54E-09 | 0.67971203 | 0.364 | 0.16 | 0.000319918 | Hepatocytes |
| PSMD4 | 9.89E-09 | 1.06417277 | 0.411 | 0.206 | 0.000331768 | Hepatocytes |
| MPC1 | 1.10E-08 | 0.87138357 | 0.318 | 0.137 | 0.000368159 | Hepatocytes |
| ATP5F1C | 1.26E-08 | 0.94757959 | 0.579 | 0.363 | 0.000422681 | Hepatocytes |
| NDUFAF3 | 1.36E-08 | 0.82643616 | 0.402 | 0.195 | 0.000455979 | Hepatocytes |
| CTSB | 1.52E-08 | 0.67139736 | 0.252 | 0.092 | 0.000509734 | Hepatocytes |
| RBCK1 | 1.54E-08 | 0.95972386 | 0.439 | 0.226 | 0.000514901 | Hepatocytes |
| FAM162A | 1.84E-08 | 0.62992957 | 0.299 | 0.119 | 0.000616851 | Hepatocytes |
| CSTB | 2.06E-08 | 1.05390168 | 0.57 | 0.337 | 0.000691494 | Hepatocytes |
| DAD1 | 2.21E-08 | 0.88030219 | 0.598 | 0.405 | 0.00074052 | Hepatocytes |
| NDUFA6 | 2.37E-08 | 0.82341453 | 0.589 | 0.376 | 0.000794018 | Hepatocytes |
| UBL5 | 2.48E-08 | 0.77561017 | 0.766 | 0.664 | 0.000831712 | Hepatocytes |
| SLIRP | 2.90E-08 | 0.74011589 | 0.374 | 0.175 | 0.000972039 | Hepatocytes |
| TMBIM6 | 3.84E-08 | 0.79129769 | 0.748 | 0.702 | 0.001289426 | Hepatocytes |
| ATP5MC2 | 4.29E-08 | 0.6519623 | 0.813 | 0.753 | 0.001437918 | Hepatocytes |
| DDRGK1 | 5.36E-08 | 0.72468544 | 0.374 | 0.185 | 0.001797671 | Hepatocytes |
| POLR2J | 5.43E-08 | 0.92089748 | 0.458 | 0.24 | 0.00182143 | Hepatocytes |
| ATXN10 | 6.19E-08 | 0.4731345 | 0.29 | 0.117 | 0.002075012 | Hepatocytes |
| RAB4A | 7.31E-08 | 0.60725478 | 0.336 | 0.15 | 0.002450897 | Hepatocytes |
| SSR3 | 8.21E-08 | 0.86051028 | 0.467 | 0.262 | 0.002753306 | Hepatocytes |
| PIN4 | 8.50E-08 | 0.75053239 | 0.29 | 0.12 | 0.002850684 | Hepatocytes |
| TMCO1 | 8.94E-08 | 0.73886275 | 0.449 | 0.255 | 0.002998794 | Hepatocytes |
| TRAM1 | 1.04E-07 | 0.93162825 | 0.514 | 0.313 | 0.003477844 | Hepatocytes |
| IER3IP1 | 1.23E-07 | 0.70568388 | 0.318 | 0.142 | 0.00411111 | Hepatocytes |
| RPS27L | 1.35E-07 | 0.84769116 | 0.542 | 0.342 | 0.00453973 | Hepatocytes |
| MX1 | 1.38E-07 | 0.59308128 | 0.318 | 0.141 | 0.004642726 | Hepatocytes |
| SEC61G | 1.60E-07 | 0.78738309 | 0.673 | 0.507 | 0.005360403 | Hepatocytes |
| NDUFS3 | 1.61E-07 | 0.60070603 | 0.318 | 0.144 | 0.005401328 | Hepatocytes |
| COX6B1 | 1.71E-07 | 0.76030942 | 0.72 | 0.605 | 0.005722985 | Hepatocytes |
| PPIF | 1.85E-07 | 0.54975658 | 0.29 | 0.124 | 0.006188503 | Hepatocytes |
| PSMA3 | 2.39E-07 | 0.89215842 | 0.439 | 0.248 | 0.008022012 | Hepatocytes |
| MRPS21 | 2.47E-07 | 0.78796671 | 0.505 | 0.301 | 0.008292449 | Hepatocytes |
| TCEA1 | 3.12E-07 | 0.82127336 | 0.43 | 0.245 | 0.01047512 | Hepatocytes |
| CNPY2 | 3.29E-07 | 0.63113035 | 0.411 | 0.214 | 0.011043426 | Hepatocytes |
| FKBP3 | 4.11E-07 | 0.55386449 | 0.29 | 0.125 | 0.013788223 | Hepatocytes |
| CHMP2A | 4.50E-07 | 0.65624952 | 0.458 | 0.248 | 0.015099464 | Hepatocytes |
| NOL7 | 4.58E-07 | 0.61073687 | 0.421 | 0.225 | 0.015371755 | Hepatocytes |
| UBE2K | 5.06E-07 | 0.71741919 | 0.346 | 0.167 | 0.016983807 | Hepatocytes |
| CD59 | 6.02E-07 | 0.49621222 | 0.28 | 0.119 | 0.020189133 | Hepatocytes |
| VPS29 | 6.08E-07 | 0.92073579 | 0.486 | 0.281 | 0.020394306 | Hepatocytes |
| ATP5MF | 6.13E-07 | 0.78018535 | 0.598 | 0.449 | 0.020564316 | Hepatocytes |
| CYC1 | 6.61E-07 | 0.70946251 | 0.43 | 0.236 | 0.022170258 | Hepatocytes |
| CNIH1 | 6.64E-07 | 0.58192717 | 0.374 | 0.185 | 0.022280377 | Hepatocytes |
| VDAC3 | 7.60E-07 | 0.68607601 | 0.364 | 0.179 | 0.025494582 | Hepatocytes |
| C1orf43 | 8.33E-07 | 0.8421372 | 0.411 | 0.229 | 0.027952809 | Hepatocytes |
| LAP3 | 8.48E-07 | 0.71079191 | 0.355 | 0.185 | 0.028432563 | Hepatocytes |
| COX20 | 9.93E-07 | 0.77816779 | 0.449 | 0.253 | 0.033315823 | Hepatocytes |
| DPM3 | 1.07E-06 | 0.81793261 | 0.308 | 0.151 | 0.035901413 | Hepatocytes |
| TIMM8B | 1.14E-06 | 0.77176689 | 0.336 | 0.168 | 0.038096504 | Hepatocytes |
| NDUFA4 | 1.14E-06 | 0.83391079 | 0.71 | 0.659 | 0.038163237 | Hepatocytes |
| PDIA4 | 1.22E-06 | 0.48500315 | 0.299 | 0.136 | 0.040843911 | Hepatocytes |
| SRP72 | 1.48E-06 | 0.87939534 | 0.411 | 0.232 | 0.049689366 | Hepatocytes |
| CD63 | 1.53E-06 | 0.493863 | 0.766 | 0.726 | 0.051167806 | Hepatocytes |
| GNAS | 1.59E-06 | 0.57542465 | 0.738 | 0.65 | 0.053466469 | Hepatocytes |
| PRDX5 | 1.60E-06 | 0.77425614 | 0.636 | 0.492 | 0.053770276 | Hepatocytes |
| ROMO1 | 1.64E-06 | 0.76808376 | 0.514 | 0.341 | 0.054908842 | Hepatocytes |
| SERF2 | 1.65E-06 | 0.48656644 | 0.925 | 0.96 | 0.05546808 | Hepatocytes |
| ACAA1 | 1.73E-06 | 0.69482519 | 0.318 | 0.157 | 0.058015887 | Hepatocytes |
| SAT2 | 2.18E-06 | 0.81245636 | 0.411 | 0.24 | 0.073196751 | Hepatocytes |
| ATP5MPL | 2.60E-06 | 0.7667012 | 0.598 | 0.436 | 0.087138854 | Hepatocytes |
| CYB5R3 | 2.76E-06 | 0.7102697 | 0.262 | 0.117 | 0.092643382 | Hepatocytes |
| RANBP1 | 2.80E-06 | 0.91328864 | 0.421 | 0.249 | 0.094001774 | Hepatocytes |
| TMEM258 | 2.84E-06 | 0.83661383 | 0.579 | 0.452 | 0.095245788 | Hepatocytes |
| SOD1 | 3.18E-06 | 0.58995666 | 0.729 | 0.628 | 0.106499816 | Hepatocytes |
| SUCLG1 | 3.68E-06 | 0.57953607 | 0.299 | 0.142 | 0.123315866 | Hepatocytes |
| UQCRC1 | 4.01E-06 | 0.65789367 | 0.299 | 0.143 | 0.134597453 | Hepatocytes |
| NDUFS7 | 4.50E-06 | 0.68852963 | 0.458 | 0.261 | 0.151061613 | Hepatocytes |
| TFG | 5.07E-06 | 0.54670067 | 0.271 | 0.128 | 0.169955933 | Hepatocytes |
| SDHD | 5.19E-06 | 0.54169319 | 0.336 | 0.173 | 0.174098514 | Hepatocytes |
| HSBP1 | 6.88E-06 | 0.52547259 | 0.29 | 0.143 | 0.230576 | Hepatocytes |
| AP2S1 | 7.21E-06 | 0.66980672 | 0.439 | 0.263 | 0.241786494 | Hepatocytes |
| NPC2 | 7.96E-06 | 0.51971548 | 0.458 | 0.279 | 0.266815415 | Hepatocytes |
| CCDC167 | 9.19E-06 | 0.66381372 | 0.374 | 0.21 | 0.308258681 | Hepatocytes |
| SLC35B1 | 1.14E-05 | 0.57096666 | 0.28 | 0.137 | 0.382190908 | Hepatocytes |
| UGP2 | 1.16E-05 | 0.64164034 | 0.458 | 0.293 | 0.389501095 | Hepatocytes |
| CSNK2B | 1.39E-05 | 0.64461476 | 0.551 | 0.389 | 0.466651108 | Hepatocytes |
| SNRPD3 | 1.52E-05 | 0.7462526 | 0.467 | 0.304 | 0.510315642 | Hepatocytes |
| POLR2I | 1.52E-05 | 0.68140473 | 0.28 | 0.14 | 0.511307042 | Hepatocytes |
| JTB | 1.58E-05 | 0.54382967 | 0.673 | 0.529 | 0.528639769 | Hepatocytes |
| PPA1 | 2.04E-05 | 0.67933735 | 0.383 | 0.214 | 0.68509399 | Hepatocytes |
| RPLP0 | 2.44E-05 | 0.62061634 | 0.869 | 0.918 | 0.817872298 | Hepatocytes |
| COX5A | 2.57E-05 | 0.60705301 | 0.598 | 0.45 | 0.86095928 | Hepatocytes |
| ACAA2 | 2.90E-05 | 0.5147333 | 0.299 | 0.156 | 0.971773631 | Hepatocytes |
| CFDP1 | 2.99E-05 | 0.39513788 | 0.262 | 0.123 | 1 | Hepatocytes |
| COX17 | 3.13E-05 | 0.68054378 | 0.43 | 0.284 | 1 | Hepatocytes |
| EDF1 | 4.65E-05 | 0.55936118 | 0.757 | 0.708 | 1 | Hepatocytes |
| MRPL22 | 4.79E-05 | 0.43836276 | 0.28 | 0.14 | 1 | Hepatocytes |
| OLA1 | 4.87E-05 | 0.36675046 | 0.327 | 0.168 | 1 | Hepatocytes |
| MRPS18C | 5.22E-05 | 0.56418203 | 0.271 | 0.135 | 1 | Hepatocytes |
| SF3B6 | 6.42E-05 | 1.02442601 | 0.533 | 0.387 | 1 | Hepatocytes |
| NDUFS8 | 7.05E-05 | 0.66020943 | 0.495 | 0.322 | 1 | Hepatocytes |
| SNHG25 | 8.14E-05 | 0.46982512 | 0.262 | 0.133 | 1 | Hepatocytes |
| DSTN | 8.37E-05 | 0.47004338 | 0.486 | 0.302 | 1 | Hepatocytes |
| SNRPD1 | 8.76E-05 | 0.55338337 | 0.43 | 0.271 | 1 | Hepatocytes |
| NDUFB6 | 9.64E-05 | 0.58017461 | 0.402 | 0.24 | 1 | Hepatocytes |
| SPCS1 | 0.000101713 | 0.75153092 | 0.579 | 0.466 | 1 | Hepatocytes |
| SEPHS2 | 0.000104002 | 0.63780914 | 0.271 | 0.143 | 1 | Hepatocytes |
| SRSF9 | 0.000113166 | 0.50479757 | 0.523 | 0.354 | 1 | Hepatocytes |
| PUF60 | 0.000117978 | 0.6455978 | 0.411 | 0.265 | 1 | Hepatocytes |
| UQCRB | 0.000134245 | 0.50751336 | 0.794 | 0.794 | 1 | Hepatocytes |
| SELENOH | 0.000145747 | 0.55684709 | 0.458 | 0.298 | 1 | Hepatocytes |
| PFDN2 | 0.000150529 | 0.47779041 | 0.458 | 0.285 | 1 | Hepatocytes |
| BAD | 0.000153303 | 0.49991645 | 0.28 | 0.147 | 1 | Hepatocytes |
| SURF1 | 0.00015908 | 0.33729171 | 0.29 | 0.152 | 1 | Hepatocytes |
| SSBP1 | 0.000159849 | 0.57958723 | 0.411 | 0.263 | 1 | Hepatocytes |
| RAD23B | 0.00016164 | 0.57446322 | 0.28 | 0.147 | 1 | Hepatocytes |
| HSPB11 | 0.000173401 | 0.53429399 | 0.29 | 0.157 | 1 | Hepatocytes |
| UQCR11 | 0.000181366 | 0.73741551 | 0.636 | 0.58 | 1 | Hepatocytes |
| LRPAP1 | 0.000195158 | 0.54957481 | 0.486 | 0.324 | 1 | Hepatocytes |
| NDUFB9 | 0.000201322 | 0.58498128 | 0.467 | 0.31 | 1 | Hepatocytes |
| NDUFB2 | 0.000211794 | 0.69507741 | 0.692 | 0.596 | 1 | Hepatocytes |
| ENY2 | 0.000214356 | 0.76443773 | 0.589 | 0.461 | 1 | Hepatocytes |
| RBX1 | 0.000233666 | 0.57403924 | 0.393 | 0.251 | 1 | Hepatocytes |
| DYNLT1 | 0.000258043 | 0.57710075 | 0.327 | 0.194 | 1 | Hepatocytes |
| HM13 | 0.000265834 | 0.67174729 | 0.355 | 0.215 | 1 | Hepatocytes |
| RPN1 | 0.000335199 | 0.41860047 | 0.29 | 0.154 | 1 | Hepatocytes |
| NDUFB5 | 0.000347803 | 0.45187434 | 0.318 | 0.183 | 1 | Hepatocytes |
| COX8A | 0.000349643 | 0.51264947 | 0.645 | 0.567 | 1 | Hepatocytes |
| KDELR2 | 0.000489182 | 0.49093486 | 0.449 | 0.295 | 1 | Hepatocytes |
| DYNLRB1 | 0.000513472 | 0.60658647 | 0.467 | 0.323 | 1 | Hepatocytes |
| GID8 | 0.000529613 | 0.41395884 | 0.29 | 0.158 | 1 | Hepatocytes |
| TXNL4A | 0.00069627 | 0.43639252 | 0.374 | 0.232 | 1 | Hepatocytes |
| MZT2B | 0.000723646 | 0.68266672 | 0.579 | 0.468 | 1 | Hepatocytes |
| DNAJC3 | 0.000728115 | 0.39127976 | 0.336 | 0.201 | 1 | Hepatocytes |
| HAX1 | 0.000734606 | 0.52463741 | 0.355 | 0.22 | 1 | Hepatocytes |
| COPS5 | 0.000796733 | 0.61525757 | 0.28 | 0.167 | 1 | Hepatocytes |
| EIF6 | 0.000802023 | 0.67763297 | 0.411 | 0.264 | 1 | Hepatocytes |
| TMEM230 | 0.000825369 | 0.7027229 | 0.467 | 0.362 | 1 | Hepatocytes |
| EIF2S2 | 0.000834472 | 0.5710508 | 0.383 | 0.245 | 1 | Hepatocytes |
| TMBIM4 | 0.000963624 | 0.42609487 | 0.43 | 0.291 | 1 | Hepatocytes |
| H3F3A | 0.001076686 | 0.43696643 | 0.907 | 0.942 | 1 | Hepatocytes |
| UBE2M | 0.001106553 | 0.46856168 | 0.355 | 0.22 | 1 | Hepatocytes |
| GDI2 | 0.001178187 | 0.49300919 | 0.43 | 0.298 | 1 | Hepatocytes |
| MDH1 | 0.001231627 | 0.57667408 | 0.336 | 0.201 | 1 | Hepatocytes |
| ATP5PO | 0.001527424 | 0.72142345 | 0.514 | 0.414 | 1 | Hepatocytes |
| TECR | 0.001556927 | 0.59194753 | 0.43 | 0.295 | 1 | Hepatocytes |
| SNX3 | 0.001631178 | 0.59495757 | 0.505 | 0.392 | 1 | Hepatocytes |
| SNRPG | 0.001760275 | 0.64226355 | 0.495 | 0.379 | 1 | Hepatocytes |
| LSM5 | 0.001777446 | 0.49668768 | 0.318 | 0.198 | 1 | Hepatocytes |
| UAP1 | 0.001784681 | 0.40578175 | 0.327 | 0.195 | 1 | Hepatocytes |
| SYPL1 | 0.001873456 | 0.43537647 | 0.271 | 0.161 | 1 | Hepatocytes |
| NDUFB8 | 0.002047394 | 0.4531574 | 0.579 | 0.456 | 1 | Hepatocytes |
| ATP5F1D | 0.002102358 | 0.58402453 | 0.617 | 0.521 | 1 | Hepatocytes |
| LAPTM4A | 0.002137439 | 0.57682529 | 0.467 | 0.363 | 1 | Hepatocytes |
| LMAN1 | 0.002196423 | 0.57289174 | 0.271 | 0.166 | 1 | Hepatocytes |
| ARL6IP4 | 0.002347589 | 0.48257194 | 0.607 | 0.517 | 1 | Hepatocytes |
| CARHSP1 | 0.002537247 | 0.4304547 | 0.327 | 0.204 | 1 | Hepatocytes |
| VDAC1 | 0.002702587 | 0.55901155 | 0.449 | 0.324 | 1 | Hepatocytes |
| PSMB7 | 0.002785134 | 0.49687949 | 0.346 | 0.229 | 1 | Hepatocytes |
| TBCA | 0.002793482 | 0.44230462 | 0.523 | 0.4 | 1 | Hepatocytes |
| NDUFAB1 | 0.00283164 | 0.40611257 | 0.29 | 0.177 | 1 | Hepatocytes |
| C19orf70 | 0.002849594 | 0.54822928 | 0.336 | 0.222 | 1 | Hepatocytes |
| TIMM17B | 0.002897328 | 0.38161503 | 0.252 | 0.147 | 1 | Hepatocytes |
| MRPL52 | 0.003986731 | 0.56101115 | 0.327 | 0.212 | 1 | Hepatocytes |
| VTI1B | 0.004548493 | 0.37976887 | 0.262 | 0.156 | 1 | Hepatocytes |
| TMEM141 | 0.004701791 | 0.39887498 | 0.271 | 0.169 | 1 | Hepatocytes |
| APLP2 | 0.004786467 | 0.41285059 | 0.308 | 0.2 | 1 | Hepatocytes |
| C8orf59 | 0.004810331 | 0.46146052 | 0.439 | 0.311 | 1 | Hepatocytes |
| MRFAP1 | 0.00515941 | 0.5508807 | 0.439 | 0.326 | 1 | Hepatocytes |
| TMEM183A | 0.005504515 | 0.26886747 | 0.271 | 0.16 | 1 | Hepatocytes |
| DGCR6L | 0.005627319 | 0.41116277 | 0.29 | 0.184 | 1 | Hepatocytes |
| PTGES3 | 0.005701832 | 0.26083861 | 0.645 | 0.552 | 1 | Hepatocytes |
| UBXN4 | 0.005732413 | 0.58047076 | 0.393 | 0.291 | 1 | Hepatocytes |
| TMEM179B | 0.005736399 | 0.37075488 | 0.252 | 0.148 | 1 | Hepatocytes |
| UQCRFS1 | 0.005851364 | 0.45949917 | 0.458 | 0.337 | 1 | Hepatocytes |
| PPIA | 0.005901635 | 0.37723613 | 0.794 | 0.808 | 1 | Hepatocytes |
| MRPL20 | 0.006204928 | 0.3741833 | 0.364 | 0.238 | 1 | Hepatocytes |
| RPA3 | 0.006252399 | 0.46685692 | 0.252 | 0.157 | 1 | Hepatocytes |
| HDGF | 0.006357986 | 0.31574128 | 0.28 | 0.173 | 1 | Hepatocytes |
| LRRC59 | 0.006393676 | 0.34172602 | 0.262 | 0.163 | 1 | Hepatocytes |
| COX7A2L | 0.006767114 | 0.3923228 | 0.449 | 0.333 | 1 | Hepatocytes |
| TXN2 | 0.007488024 | 0.35353984 | 0.29 | 0.19 | 1 | Hepatocytes |
| OSTC | 0.007693996 | 0.45889558 | 0.383 | 0.278 | 1 | Hepatocytes |
| ZNF706 | 0.008020566 | 0.43757056 | 0.43 | 0.323 | 1 | Hepatocytes |
| APEX1 | 0.008033374 | 0.64772552 | 0.271 | 0.178 | 1 | Hepatocytes |
| TUBB2A | 0.008450732 | 0.51569418 | 0.262 | 0.172 | 1 | Hepatocytes |
| TXNL1 | 0.00845282 | 0.34667147 | 0.346 | 0.238 | 1 | Hepatocytes |
| PSMC3 | 0.008923309 | 0.33120872 | 0.327 | 0.222 | 1 | Hepatocytes |
| APH1A | 0.008928743 | 0.56296759 | 0.336 | 0.233 | 1 | Hepatocytes |
| C19orf24 | 0.009100341 | 0.35790927 | 0.308 | 0.201 | 1 | Hepatocytes |
| RHOB | 0.009389847 | 0.34920444 | 0.449 | 0.34 | 1 | Hepatocytes |
| COPS6 | 0.009913949 | 0.49144643 | 0.355 | 0.253 | 1 | Hepatocytes |
| TMEM70 | 0.0099905 | 0.70460826 | 0.252 | 0.173 | 1 | Hepatocytes |
| HBB | 0 | 12.4152095 | 1 | 0.01 | 0 | Erythroblast |
| HBA2 | 0 | 9.17596951 | 0.985 | 0.016 | 0 | Erythroblast |
| HBA1 | 0 | 8.74083934 | 1 | 0.032 | 0 | Erythroblast |
| HBD | 0 | 8.02087679 | 0.985 | 0 | 0 | Erythroblast |
| HBM | 0 | 5.74986884 | 0.758 | 0 | 0 | Erythroblast |
| AHSP | 0 | 5.62036726 | 0.848 | 0.002 | 0 | Erythroblast |
| CA1 | 0 | 5.09511043 | 0.621 | 0 | 0 | Erythroblast |
| ALAS2 | 0 | 4.48044414 | 0.712 | 0 | 0 | Erythroblast |
| GYPA | 0 | 3.03133441 | 0.348 | 0 | 0 | Erythroblast |
| SELENBP1 | 0 | 2.72157749 | 0.318 | 0.002 | 0 | Erythroblast |
| GYPB | 0 | 2.71407516 | 0.303 | 0 | 0 | Erythroblast |
| SNCA | 1.77E-260 | 3.12163747 | 0.379 | 0.007 | 5.93E-256 | Erythroblast |
| HEMGN | 3.49E-253 | 2.56865598 | 0.273 | 0.003 | 1.17E-248 | Erythroblast |
| HMBS | 1.27E-78 | 2.70889471 | 0.288 | 0.014 | 4.25E-74 | Erythroblast |
| SLC25A37 | 2.18E-48 | 3.19454013 | 0.47 | 0.062 | 7.33E-44 | Erythroblast |
| BLVRB | 3.57E-36 | 3.58150472 | 0.636 | 0.161 | 1.20E-31 | Erythroblast |
| EIF1AY | 1.07E-28 | 2.94303336 | 0.485 | 0.108 | 3.59E-24 | Erythroblast |
| SLC25A39 | 2.15E-17 | 2.88897784 | 0.545 | 0.226 | 7.20E-13 | Erythroblast |
| LGALS3 | 9.75E-17 | 2.77750517 | 0.409 | 0.121 | 3.27E-12 | Erythroblast |
| GLRX5 | 9.99E-17 | 2.77355526 | 0.47 | 0.164 | 3.35E-12 | Erythroblast |
| PRDX2 | 6.89E-12 | 3.08633376 | 0.5 | 0.253 | 2.31E-07 | Erythroblast |
| UROD | 1.91E-11 | 2.10628149 | 0.258 | 0.067 | 6.42E-07 | Erythroblast |
| GYPC | 3.07E-09 | 1.70226758 | 0.652 | 0.637 | 0.00010299 | Erythroblast |
| BNIP3L | 2.49E-08 | 2.27403707 | 0.364 | 0.166 | 0.000834981 | Erythroblast |
| YBX3 | 3.87E-08 | 2.19186006 | 0.288 | 0.107 | 0.001296722 | Erythroblast |
| BSG | 1.88E-06 | 2.046267 | 0.545 | 0.495 | 0.063166156 | Erythroblast |
| BPGM | 1.77E-05 | 1.84470078 | 0.288 | 0.142 | 0.592836985 | Erythroblast |
| PRDX6 | 0.000231069 | 0.25287646 | 0.167 | 0.49 | 1 | Erythroblast |
| SNRPD1 | 0.000453958 | 0.30861456 | 0.061 | 0.272 | 1 | Erythroblast |
| TUBA1B | 0.000746364 | 0.85718846 | 0.273 | 0.691 | 1 | Erythroblast |
| SNX3 | 0.001140017 | 0.35778096 | 0.136 | 0.393 | 1 | Erythroblast |
| FBXO7 | 0.001406767 | 1.98544705 | 0.333 | 0.242 | 1 | Erythroblast |
| UBB | 0.001873164 | 0.60362876 | 0.864 | 0.978 | 1 | Erythroblast |
| HIST1H4C | 0.002350281 | 0.92699268 | 0.121 | 0.346 | 1 | Erythroblast |
| HMGB2 | 0.002832095 | 0.43373993 | 0.242 | 0.566 | 1 | Erythroblast |
| FIS1 | 0.005876363 | 0.54700448 | 0.136 | 0.35 | 1 | Erythroblast |
|  |  |  |  |  |  |  |
