# Supplementary material for: Combining bulk and single-cell RNA-sequencing data to develop an NK cell-related prognostic signature for hepatocellular carcinoma based on an integrated machine learning framework
Source: Eur J Med Res. 2023 Aug 30;28:306. doi: 10.1186/s40001-023-01300-6 (PMC10466881; doi:10.1186/s40001-023-01300-6)
Supplement: Supplementary file 10 — Additional file 10. The 245 survival-related NK cell markers selected by WGCNA. [file 40001_2023_1300_MOESM10_ESM.docx]

Additional file 10. The 245 survival-related NK cell markers selected by WGCNA.

| **No.** | **Gene Name** |
| --- | --- |
| 1 | GNLY |
| 2 | GZMB |
| 3 | CTSW |
| 4 | NKG7 |
| 5 | KLRB1 |
| 6 | CCL3 |
| 7 | PRF1 |
| 8 | GZMA |
| 9 | CD247 |
| 10 | TRDC |
| 11 | ALOX5AP |
| 12 | CST7 |
| 13 | HOPX |
| 14 | CCL5 |
| 15 | GZMM |
| 16 | CCL4 |
| 17 | GSTP1 |
| 18 | ADGRE5 |
| 19 | LITAF |
| 20 | HCST |
| 21 | PLAC8 |
| 22 | PFN1 |
| 23 | TYROBP |
| 24 | HLA-A |
| 25 | FCER1G |
| 26 | HLA-C |
| 27 | LAT2 |
| 28 | CDC42SE1 |
| 29 | ITGB2 |
| 30 | CD63 |
| 31 | TAGLN2 |
| 32 | GNG2 |
| 33 | SRSF2 |
| 34 | GZMH |
| 35 | CFL1 |
| 36 | ARHGDIB |
| 37 | IL2RB |
| 38 | NFKB1 |
| 39 | EIF5A |
| 40 | EFHD2 |
| 41 | LSP1 |
| 42 | BIN2 |
| 43 | IL2RG |
| 44 | CHST12 |
| 45 | PDIA3 |
| 46 | RPS17 |
| 47 | CD69 |
| 48 | SRGN |
| 49 | CD53 |
| 50 | TGFB1 |
| 51 | TMSB4X |
| 52 | VPS37B |
| 53 | TLE1 |
| 54 | HSPA5 |
| 55 | SLC16A3 |
| 56 | RAC2 |
| 57 | AOAH |
| 58 | LDHA |
| 59 | APOBEC3G |
| 60 | ANXA1 |
| 61 | IRF8 |
| 62 | SLC15A4 |
| 63 | CLIC1 |
| 64 | BHLHE40 |
| 65 | BZW1 |
| 66 | MAPK1 |
| 67 | RELB |
| 68 | NINJ1 |
| 69 | NCR3 |
| 70 | BSG |
| 71 | GZMK |
| 72 | C12orf75 |
| 73 | CALR |
| 74 | XCL1 |
| 75 | OSTF1 |
| 76 | GPR65 |
| 77 | HNRNPA1 |
| 78 | SYTL3 |
| 79 | GYPC |
| 80 | FOSL2 |
| 81 | SLA2 |
| 82 | TAPBP |
| 83 | HLA-E |
| 84 | RBM39 |
| 85 | AKNA |
| 86 | CORO1A |
| 87 | ARPC2 |
| 88 | RAP1B |
| 89 | PTMA |
| 90 | WIPF1 |
| 91 | YPEL5 |
| 92 | LCP1 |
| 93 | METRNL |
| 94 | SNRPB |
| 95 | EVL |
| 96 | SH2D2A |
| 97 | ITGB7 |
| 98 | ANKRD28 |
| 99 | PPP1CA |
| 100 | FCGR3A |
| 101 | TSEN54 |
| 102 | ACTG1 |
| 103 | GLIPR2 |
| 104 | HMGN3 |
| 105 | HNRNPC |
| 106 | ABHD17A |
| 107 | UPP1 |
| 108 | HNRNPK |
| 109 | CHCHD2 |
| 110 | IGKC |
| 111 | MYADM |
| 112 | BIRC2 |
| 113 | ATP1B3 |
| 114 | IRF1 |
| 115 | THAP2 |
| 116 | AC245297.3 |
| 117 | RAB8B |
| 118 | KMT2E |
| 119 | RBM3 |
| 120 | ICAM1 |
| 121 | ARHGAP9 |
| 122 | SERBP1 |
| 123 | SIRT2 |
| 124 | PSME2 |
| 125 | UBE2L3 |
| 126 | FMNL1 |
| 127 | TPST2 |
| 128 | POLR2G |
| 129 | ISG20 |
| 130 | INPP5D |
| 131 | STARD3NL |
| 132 | PCBP2 |
| 133 | CMTM3 |
| 134 | PPIB |
| 135 | PHF20 |
| 136 | TES |
| 137 | HSH2D |
| 138 | MSN |
| 139 | RALY |
| 140 | TPM4 |
| 141 | NFKB2 |
| 142 | GPSM3 |
| 143 | NFKBIZ |
| 144 | AC004687.1 |
| 145 | MYO1F |
| 146 | ENO1 |
| 147 | CCND2 |
| 148 | CLDND1 |
| 149 | ARHGDIA |
| 150 | JAK1 |
| 151 | BRAF |
| 152 | UBE2D3 |
| 153 | CD164 |
| 154 | CHMP1B |
| 155 | JMJD6 |
| 156 | SKIL |
| 157 | ZAP70 |
| 158 | CSNK1D |
| 159 | OFD1 |
| 160 | PTPN7 |
| 161 | CD96 |
| 162 | CAPZB |
| 163 | TAP1 |
| 164 | TRAPPC1 |
| 165 | NFE2L2 |
| 166 | OTULIN |
| 167 | EIF3I |
| 168 | BUD31 |
| 169 | ARID4B |
| 170 | RPL17 |
| 171 | HNRNPM |
| 172 | KDM6B |
| 173 | RASSF5 |
| 174 | KDELR2 |
| 175 | RHOG |
| 176 | ARF4 |
| 177 | ELOVL5 |
| 178 | MYL12A |
| 179 | CAP1 |
| 180 | SPPL2A |
| 181 | PRKAR1A |
| 182 | BAZ1A |
| 183 | ILF2 |
| 184 | ARPC5L |
| 185 | PRMT2 |
| 186 | FAM118A |
| 187 | LSM3 |
| 188 | OPTN |
| 189 | TMED10 |
| 190 | TMED2 |
| 191 | LINC00623 |
| 192 | RSRP1 |
| 193 | LMO4 |
| 194 | FLNA |
| 195 | RNPS1 |
| 196 | RNF115 |
| 197 | PIP4K2A |
| 198 | UBE2I |
| 199 | CDKN2D |
| 200 | SURF4 |
| 201 | PDCD4 |
| 202 | ZEB2 |
| 203 | AP2M1 |
| 204 | ARHGAP45 |
| 205 | HES4 |
| 206 | MAZ |
| 207 | RASSF1 |
| 208 | SPN |
| 209 | ACTN4 |
| 210 | SELENOT |
| 211 | STRAP |
| 212 | SAR1A |
| 213 | MBP |
| 214 | PPP1R18 |
| 215 | IER3 |
| 216 | TLN1 |
| 217 | FNIP1 |
| 218 | CPNE1 |
| 219 | PIM2 |
| 220 | RALGAPA1 |
| 221 | MED10 |
| 222 | EIF4H |
| 223 | SLC9A3R1 |
| 224 | METTL9 |
| 225 | KLF3 |
| 226 | PSMC4 |
| 227 | CASP4 |
| 228 | CXCR4 |
| 229 | HNRNPUL1 |
| 230 | RNF166 |
| 231 | GATA3 |
| 232 | SDF4 |
| 233 | SH3GLB1 |
| 234 | NXT1 |
| 235 | RNF168 |
| 236 | DSTN |
| 237 | SEC14L1 |
| 238 | PGK1 |
| 239 | GGNBP2 |
| 240 | CNOT2 |
| 241 | HIPK1 |
| 242 | DDX27 |
| 243 | YME1L1 |
| 244 | MAPRE2 |
| 245 | PPP1R15A |
|  |  |
